# Sodium Borates: Expanding the Electrolyte Selection for Sodium‐Ion Batteries

**DOI:** 10.1002/anie.202202133

**Published:** 2022-05-03

**Authors:** Darren M. C. Ould, Svetlana Menkin, Holly E. Smith, Victor Riesgo‐Gonzalez, Erlendur Jónsson, Christopher A. O'Keefe, Fazlil Coowar, Jerry Barker, Andrew D. Bond, Clare P. Grey, Dominic S. Wright

**Affiliations:** ^1^ Yusuf Hamied Department of Chemistry University of Cambridge Lensfield Road Cambridge CB2 1EW UK; ^2^ The Faraday Institution Quad One, Harwell Science and Innovation Campus Didcot UK; ^3^ Faradion Limited, The Innovation Centre 217 Portobello Sheffield S1 4DP UK

**Keywords:** Batteries, Electrolytes, Main Group Synthesis, SEI, Sodium-Ion Batteries

## Abstract

Sodium‐ion batteries (SIBs) are a promising grid‐level storage technology due to the abundance and low cost of sodium. The development of new electrolytes for SIBs is imperative since it impacts battery life and capacity. Currently, sodium hexafluorophosphate (NaPF_6_) is used as the benchmark salt, but is highly hygroscopic and generates toxic HF. This work describes the synthesis of a series of sodium borate salts, with electrochemical studies revealing that Na[B(hfip)_4_]⋅DME (hfip=hexafluoroisopropyloxy, O^i^Pr^F^) and Na[B(pp)_2_] (pp=perfluorinated pinacolato, O_2_C_2_(CF_3_)_4_) have excellent electrochemical performance. The [B(pp)_2_]^−^ anion also exhibits a high tolerance to air and water. Both electrolytes give more stable electrode‐electrolyte interfaces than conventionally used NaPF_6_, as demonstrated by impedance spectroscopy and cyclic voltammetry. Furthermore, they give greater cycling stability and comparable capacity to NaPF_6_ for SIBs, as shown in commercial pouch cells.

## Graphical abstract

Sodium‐ion batteries (SIBs) are a promising technology for grid‐level storage, but require electrolytes specifically optimized for them. This work showcases the synthesis of a series of sodium borate salts that can act as electrolytes for SIBs. Electrochemical studies reveal Na[B(hfip)_4_]⋅DME (hfip=hexafluoroisopropyloxy, O^i^Pr^F^) and Na[B(pp)_2_] (pp=perfluorinated pinacolato, O_2_C_2_(CF_3_)_4_) are excellent alternatives to conventionally used NaPF_6_.

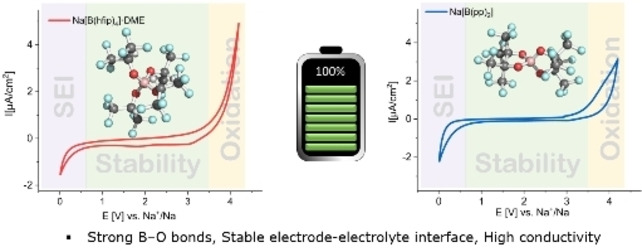

## Introduction

The ambition to create a world energy supply based on renewable energy sources requires suitable technology for large‐scale grid storage. This global drive towards electrification will not only affect energy production but will impact transportation, given the large greenhouse gas emissions these sectors currently produce.[^1^, ^2^] While lithium‐ion batteries (LIBs) currently lead the way in battery technology, the relative low abundance and high cost of lithium, limitations on the transition metals used in the cathode, as well as ever increasing demand for LIBs means that alternative battery technologies are urgently required.[^3^, ^4^, ^5^] Given the wider abundance and lower cost of sodium, sodium‐ion batteries (SIBs) are increasingly being viewed as an attractive candidate for applications where the size and weight of the battery are less of a concern, as the inherently lower energy density of SIBs is offset by reduced cell cost.^[6]^ The sustainability of SIBs is further improved as they allow cobalt‐free cathodes to be used,^[7]^ and aluminium current collectors can be employed at the anode, in contrast to copper used in LIBs.^[8]^

Adopted from the LIB field, which typically uses lithium hexafluorophosphate as the anion of choice, the current benchmark electrolyte for SIBs is sodium hexafluorophosphate (NaPF_6_) in a carbonate solvent mixture.[^9^, ^10^] While NaPF_6_ offers high ionic conductivity, its high susceptibility to undergo hydrolysis is problematic, as toxic HF is formed as well as NaF and POF_3_ (which goes on to further react with water).[^11^, ^12^] The presence of NaF causes solubility difficulties as highlighted in our previous work,^[13]^ whereas the ability to form HF and POF_3_ poses significant safety concerns due to their high toxicity; a recent report showing high levels of these gases are produced in LIB fires.^[14]^ Furthermore, the presence of PF_6_^−^ and its toxic breakdown products add extra challenges to battery recycling.

Other sodium electrolyte salts may be used instead of NaPF_6_, but alternatives suffer from either safety concerns, poor electrochemical performance or high cost.[^10^, ^15^, ^16^] NaClO_4_ is a popular choice and is widely studied in the literature, but the ClO_4_^−^ anion is a strong oxidant (potential explosive) so its use is unsuitable for commercial applications. Additional electrolyte salts include NaTFSI (TFSI=bis(trifluoromethylsulfonyl)imide) and NaFSI (FSI=bis(fluorosulfonyl)imide), which are attractive due to their high thermal stabilities and non‐toxicity, but cannot be used as single salts as they are corrosive towards the aluminium current collector.^[17]^

As the electrolyte remains underexplored in SIBs, it would be desirable to design an electrolyte salt that is much less hygroscopic than NaPF_6_, not strongly oxidizing like NaClO_4_, and has decomposition products that have reduced toxicity. The electrolyte salt should also offer high energy density, high conductivity and a wide electrochemical stability window (ESW). With that in mind, we have turned to borate anions, which offer a number of attractive features as electrolyte salts. Sodium borates can be synthesized from cheap and commercially available starting materials, contain strong B−O bonds to aid chemical stability and have tunable steric and electronic properties by tailoring the ligands coordinated to the central boron heteroatom.

While limited, the literature contains examples where borate anions have been used as electrolyte salts in battery systems. Arguably the most familiar is bis(oxalato)borate (BOB), which has been well‐studied as an electrolyte salt and additive in LIBs.[^18^, ^19^, ^20^, ^21^, ^22^, ^23^] The sodium analogue (NaBOB) is known and has recently been shown to behave as a non‐flammable electrolyte when dissolved in trimethyl phosphate (TMP) solvent.^[24]^ However, NaBOB suffers from low solubility in traditionally used carbonate solvents, hence sodium difluoro(oxalato)borate (NaDFOB) is used as a more soluble alternative.^[25]^

**Figure 1:**
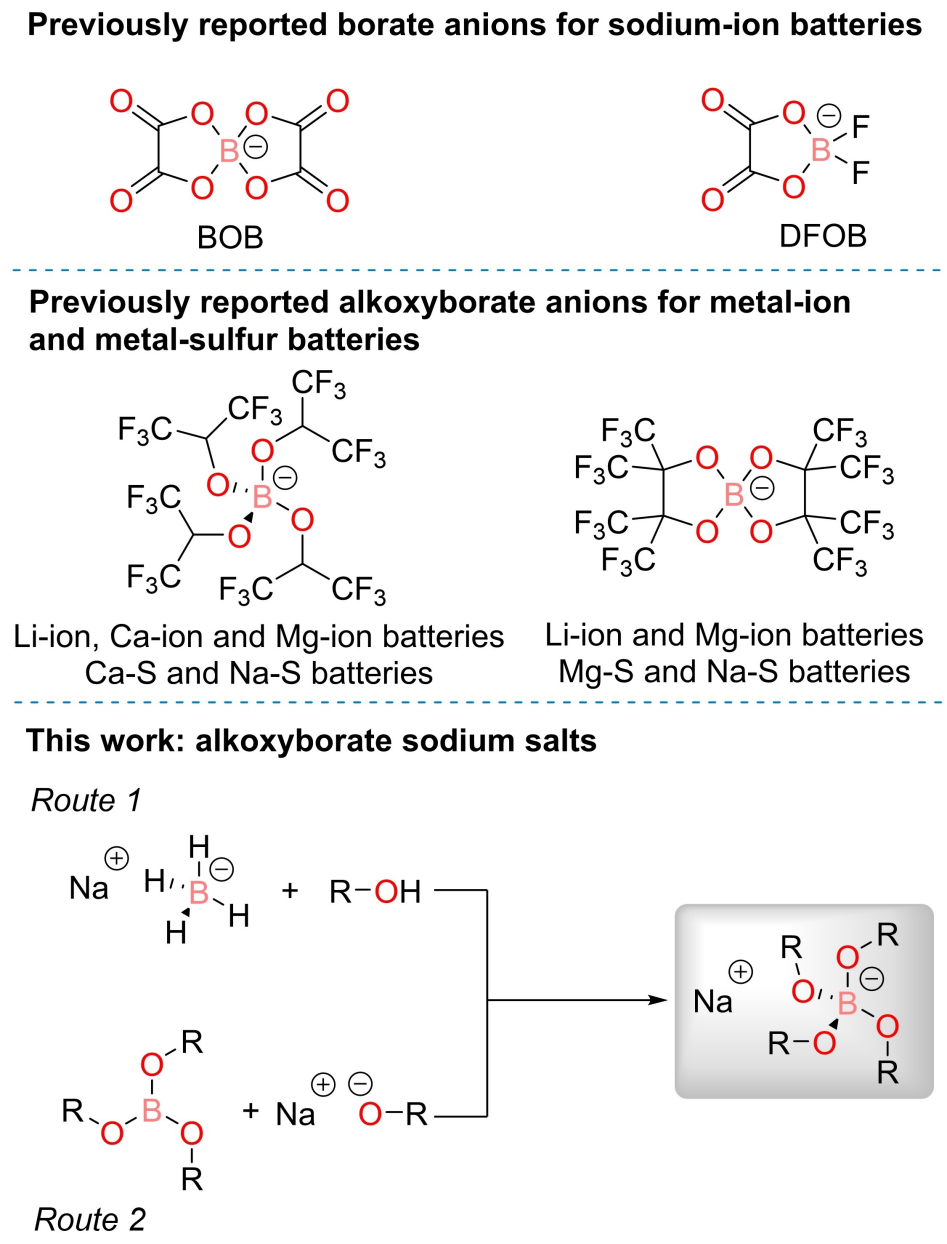
Top: Previously reported borate anions for sodium‐ion batteries.[^24^, ^25^] Middle: Previously reported fluorinated alkoxyborate anions in metal‐ion and metal‐sulfur batteries.[^33^, ^34^, ^35^, ^36^, ^37^, ^38^, ^39^, ^40^, ^41^, ^42^, ^43^, ^44^] Bottom: General scheme of the synthetic routes to prepare a wide range of borate anions, as illustrated in this work.

Away from BOB derived anions, the LIB field has previously looked at using organoborates that feature a fused aromatic ring in the backbone.[^26^, ^27^, ^28^, ^29^, ^30^] These anions have varying degrees of fluorination, with an increase in fluorination giving a greater ESW.^[31]^ More recently, the lithium‐ion, magnesium‐ion and calcium‐ion battery fields have produced fluorinated alkoxyborate complexes to serve as weakly coordinated anions (Figure 1). Inspired by the aluminate analogue,^[32]^ Mg[B(hfip)_4_]_2_ (hfip=hexafluoroisopropyloxy, O^i^Pr^F^) has been used in magnesium‐ion batteries, where high anodic stability, high ionic conductivity and high Coulombic efficiency of magnesium deposition were found.^[33]^ A detailed study of the interactions present in the Mg[B(hfip)_4_]_2_ electrolyte has revealed the fine balance between ligand stabilization and electron‐withdrawing effects.^[34]^

Additionally, the B(hfip)_4_^−^ anion has been used in LIBs, calcium‐ion batteries, calcium‐sulfur batteries and magnesium‐sulfur batteries, with high stability and reversibility again observed.[^35^, ^36^, ^37^, ^38^] Alternatively, the B(pp)_2_^−^ (pp=perfluorinated pinacolato, O_2_C_2_(CF_3_)_4_) anion has been investigated for use in LIBs and magnesium‐ion batteries. Li[B(pp)_2_] was shown to offer excellent conductivity and electrochemical stability,[^39^, ^40^] whereas Mg[B(pp)_2_]_2_ enabled reversible magnesium deposition and gave an anodic stability of 4.0 V vs. Mg.[^41^, ^42^] Of interest, both the B(hfip)_4_^−^ and B(pp)_2_^−^ anions have very recently been studied in sodium‐sulfur batteries, but importantly this study did not look at sodium‐ion application or SEI stability.[^43^, ^44^]

This paper describes the synthesis of a series of sodium borate salts from cheap and readily available starting materials, creating a scalable method for electrolyte production. Once synthesized, the chemical, thermal and electrochemical stability of these salts was evaluated to determine their suitability to act as electrolyte salts for SIBs. The results of these studies show that the electrolyte salts Na[B(hfip)_4_]⋅DME (**1 a**) and Na[B(pp)_2_] (**1 b′**) give greater cycling stability and comparable capacity to NaPF_6_, while the latter also shows high tolerance to air and water. This greater chemical stability allows for convenient handling, transport and storage of the salt, which is an important requirement for use in a commercial battery. Moreover, this will aid the vital transition of moving from LIBs to the more sustainable SIBs.

## Results and Discussion

Since sodium borohydride is an ideal starting material that can be purchased at low cost and in high‐grade, without the need of further purification, it was used to prepare the sodium salts of the fluorinated borate anions. Synthesis proceeded by reacting sodium borohydride with a small excess of chosen fluorinated alcohol in 1,2‐dimethoxyethane (DME) solvent.^[45]^ The resulting solution is heated to reflux for 6 hours, after which the solution is cooled and left to stir at 50 °C for 16 hours. Removal of the solvent gives the desired sodium borate product, which is purified by precipitating the salt out of a concentrated DME solution using pentane.

**Scheme 1:**
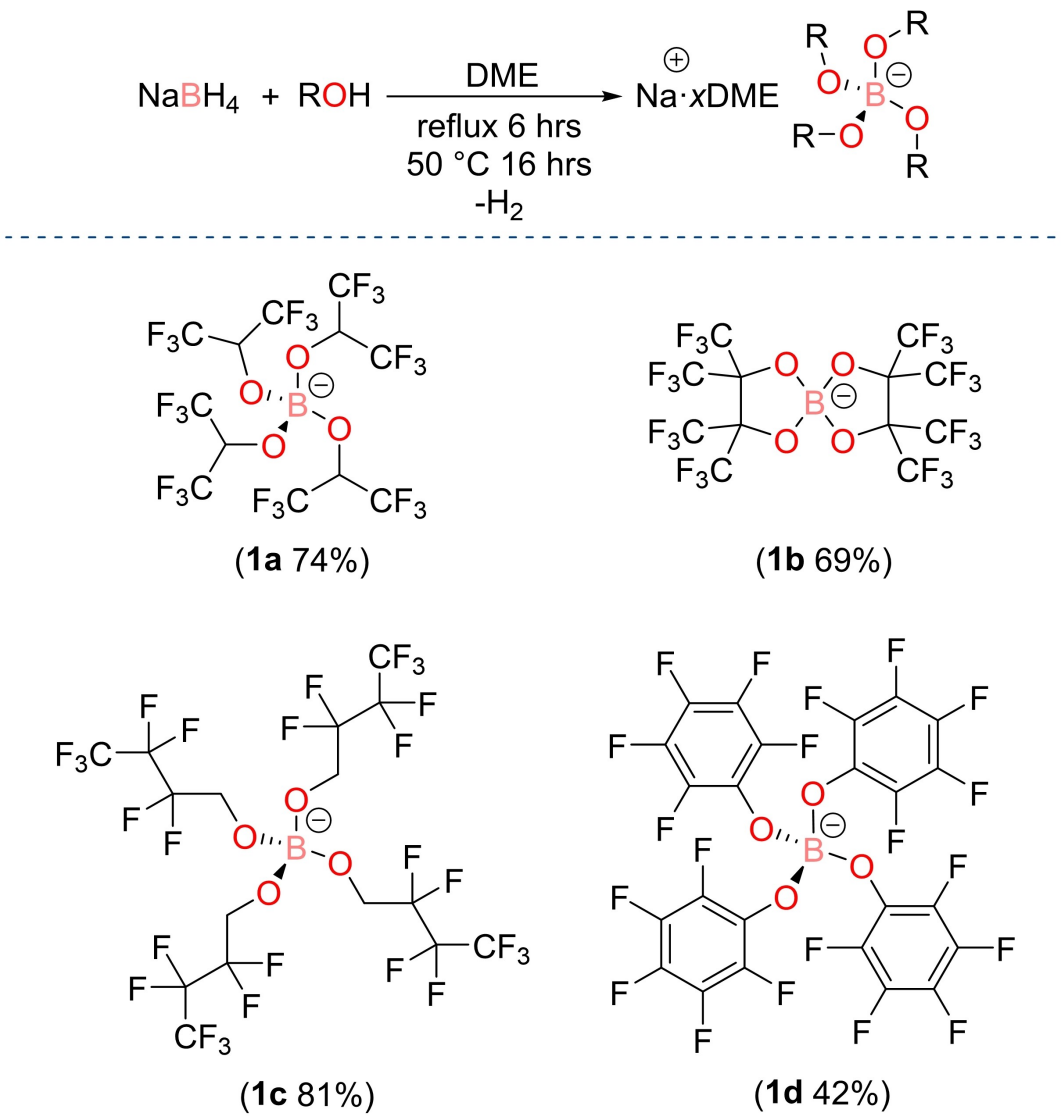
Overall reaction scheme (top) used to synthesize Na[B(hfip)_4_]⋅DME (**1 a**), Na[B(pp)_2_]⋅3 DME (**1 b**), Na[B(OCH_2_(CF_2_)_2_CF_3_)_4_] (**1 c**) and Na[B(OPh^F^)_4_]⋅3 DME (**1 d**). Note Na[B(OCH_2_(CF_2_)_2_CF_3_)_4_] (**1 c**) does not form as a DME adduct, where DME=1,2‐dimethoxyethane.

The first salt synthesized using this protocol was sodium tetrakis(hexafluoroisopropyloxy)borate, Na[B(hfip)_4_]⋅DME (**1 a**), which was isolated in good yield (74 %, Scheme 1). Inspection of the ^11^B solution NMR spectrum in CD_3_CN solvent showed a sharp signal at 1.7 ppm, which is indicative of a four‐coordinate borate, and complete consumption of NaBH_4_. The ^1^H NMR spectrum showed the expected broad singlet resonance at 4.72 ppm, but also two signals at 3.46 and 3.29 ppm. The latter two signals correspond to DME, meaning DME solvates the sodium cation and when dried at 90 °C under vacuum, stoichiometric solvent coordination results.

**Figure 2:**
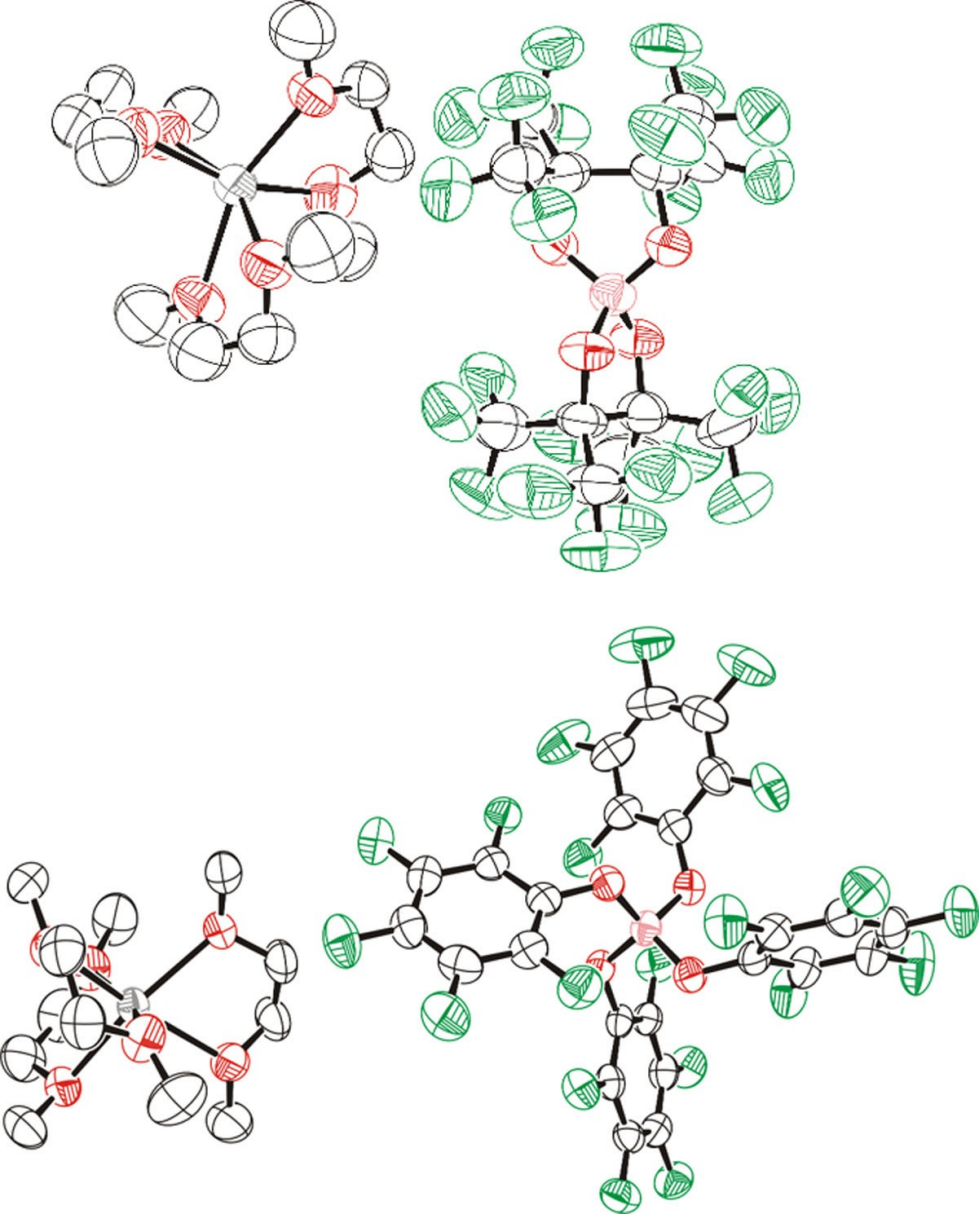
Solid‐state structures of sodium borates Na[B(pp)_2_]⋅3 DME (**1 b**) (top) and Na[B(OPh^F^)_4_]⋅3 DME (**1 d**) (bottom).^[49]^ Pink: boron; red: oxygen; green: fluorine; grey: sodium. Displacement ellipsoids drawn at 50 % probability and H‐atoms omitted. Disorder of the DME ligands in Na[B(pp)_2_]⋅3 DME is also omitted for clarity.

The synthetic protocol of adding a fluorinated alcohol to sodium borohydride was extended to produce a range of borate anions that have varying steric and electronic properties. Using this method, sodium bis(perfluorinated pinacolato)borate, Na[B(pp)_2_]⋅3 DME (**1 b**), was produced, which again was isolated in good yield (69 %). Like **1 a**, inspection of the ^1^H NMR spectrum showed DME solvent signals. However, compared to **1 a**, the ^11^B NMR spectrum revealed a more downfield chemical shift of 11.4 ppm, consistent with previous findings for this anion.[^39^, ^41^] Single crystals suitable for X‐ray diffraction measurements were grown by hot recrystallisation from DME; the resulting solid‐state structure revealed three DME solvent molecules coordinate to the sodium cation (Figure 2). These solvent molecules are labile, as observed by a lower level of DME solvation by elemental analysis and for **1 b** the DME could be fully removed by heating under vacuum at 140 °C for 48 hours. This gives unsolvated Na[B(pp)_2_] (**1 b′**). No DME signals are observed in the ^1^H NMR spectrum for **1 b′** (Figure S10.1.9).

The fluorinated alcohol perfluoropropyl carbinol was used to give the product sodium tetrakis(2,2,3,3,4,4,4‐heptafluorobutoxy)borate, Na[B(OCH_2_(CF_2_)_2_CF_3_)_4_] (**1 c**), in excellent yield (81 %). Unlike **1 a** and **1 b**, no DME was detected in the ^1^H NMR spectrum, with just the CH_2_ signals observed at 3.88 ppm. The last fluorinated salt to be produced was sodium tetrakis(perfluorophenoxy)borate, Na[B(OPh^F^)_4_]⋅3 DME (**1 d**), which was isolated in a 42 % yield (the lower yield is on account of multiple recrystallisation steps required). Like **1 b**, single‐crystal X‐ray diffraction showed that three DME molecules solvate the Na cation (Figure 2). Again, these solvent molecules are labile, as less DME was observed in elemental analysis results.

**Scheme 2:**
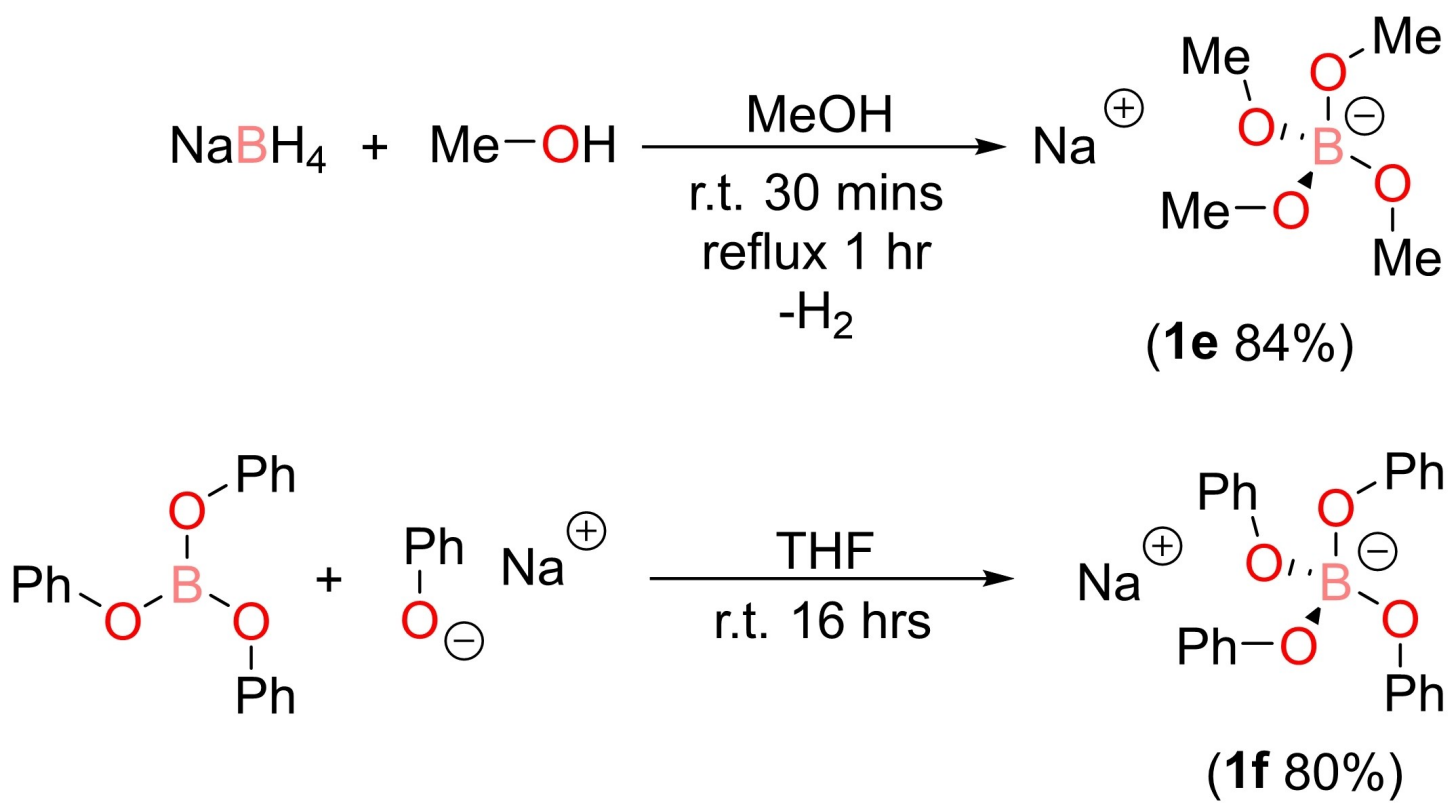
Top: Synthesis of Na[B(OMe)_4_] (**1 e**). Bottom: Synthesis of Na[B(OPh)_4_] (**1 f**).

Having produced a series of fluorinated sodium borate salts, two non‐fluorinated borate anions were synthesized. This was firstly to compare the effect of the strong electron withdrawing nature of the fluorine group, but secondly non‐fluorinated electrolytes are desirable due to potential toxicity and corrosivity of some fluorinated compounds.[^46^, ^47^] In contrast to the procedures used to synthesize the fluorinated borate anions, sodium tetramethoxyborate, Na[B(OMe)_4_] (**1 e**), was prepared by adding sodium borohydride to a large excess of methanol and heating to reflux for one hour (Scheme 2, top). This reaction is vigorous, with the loss of dihydrogen immediately observed.

Initial attempts to synthesize sodium tetraphenoxyborate, Na[B(OPh)_4_], from the addition of sodium borohydride with phenol gave a ^11^B NMR spectrum that contained multiple signals, suggesting incomplete product conversion and thus required a different synthetic protocol. Instead, by adding sodium phenoxide to triphenyl borate in THF solvent and leaving to stir at ambient temperature overnight, Na[B(OPh)_4_] (**1 f**) was isolated in an excellent yield of 80 % (Scheme 2, bottom).^[48]^

Additionally, the synthesis of the non‐fluorinated salt Na[B(O^i^Pr)_4_] (**1 g**) was attempted using the same method to produce **1 f**. However, the inherent low solubility of **1 g** made purification difficult and consequently this salt was not explored as an electrolyte salt for SIBs (see Supporting Information for synthetic details).

With a series of sodium borate salts in hand, the chemical stability with respect to air and moisture was first probed. An electrolyte salt with a high tolerance for air and water is desirable as it allows for easy handling, transport and storage. Moreover, it has been proposed that decomposition of carbonate solvents during battery operation can generate water, which can in turn decompose the electrolyte salt.^[50]^ Assessing air stability, salts **1 a**–**1 f** were left open to air in uncapped vials for 24 hours and 48 hours. For water stability, the addition of 1 equiv, 5 equiv and then 10 equiv of water to NMR samples of the salts in CD_3_CN was performed, being left for 24 hours in each case. Multinuclear NMR spectroscopy was used to assess whether degradation had taken place, where liberation of the alcohol and formation of Na[B(OH)_4_] were likely products of complete hydrolysis.

The results of the chemical stability experiments clearly showed Na[B(pp)_2_]⋅3 DME (**1 b**) to be the most stable salt with respect to air and water, with no signs of decomposition in the ^1^H, ^11^B or ^19^F NMR spectra. However, decomposition of the [B(pp)_2_]^−^ anion could be forced by using D_2_O alone as the NMR solvent (Figures S10.3.7–S10.3.9). The remaining borate salts all showed varying signs of decomposition/hydrolysis in these experiments. Markedly, both Na[B(OPh^F^)_4_]⋅3 DME (**1 d**) and Na[B(OPh)_4_] (**1 f**) were highly sensitive to both air and moisture, with signs of decomposition present after exposure to air for 24 hours (see Supporting Information S2).

**Figure 3:**
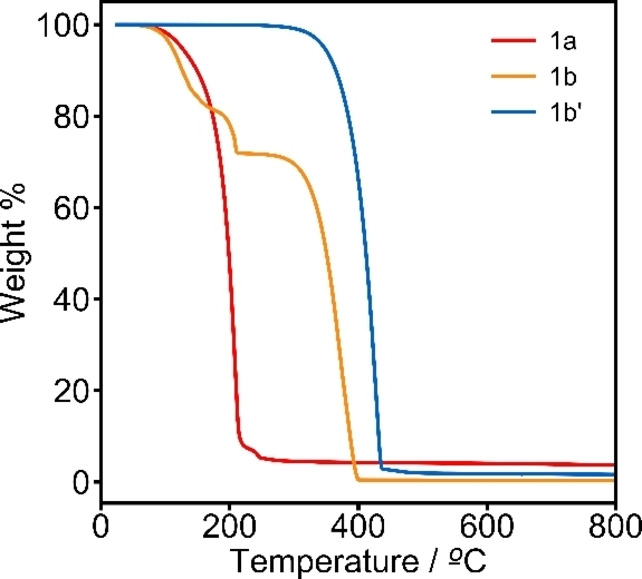
TGA curves for salts Na[B(hfip)_4_]⋅DME (**1 a**) (red), Na[B(pp)_2_]⋅3 DME (**1 b**) (orange) and Na[B(pp)_2_] (**1 b′**) (blue). Heating rate of 10 °C min^‐1^ and under a nitrogen flow.

Thermogravimetric analysis (TGA) of salts **1 a**–**1 f** was performed to understand the thermal stability of the electrolyte salts; all studied salts are thermally stable during battery operating temperatures. From the series of synthesized salts, Na[B(hfip)_4_]⋅DME (**1 a**) was the first to thermally decompose, with an onset temperature of 166 °C (Figure S4.2.2). In contrast, Na[B(pp)_2_]⋅3 DME (**1 b**) and Na[B(pp)_2_] (**1 b′**) exhibit much higher thermal stabilities, with onset temperatures of 328 °C and 370 °C respectively (Figure 3). The initial mass loss of 24 % at an onset temperature of 171 °C in Na[B(pp)_2_]⋅3 DME is assigned to the removal of DME solvent. Salts **1 c**–**1 f** all have onset temperatures between the values of Na[B(hfip)_4_]⋅DME and Na[B(pp)_2_] (see Supporting Information S4).

Having investigated chemical and thermal stability, electrochemical measurements on the synthesized sodium borate salts were then performed in order to ascertain their suitability to act as electrolyte salts for SIBs. Conductivity measurements, electrochemical impedance spectroscopy (EIS), cyclic voltammetry (CV) and galvanostatic cycling of Na‐ion battery cells were all performed in a binary ethylene carbonate:diethyl carbonate (EC:DEC) 1 : 1 v/v solvent system.

**Figure 4:**
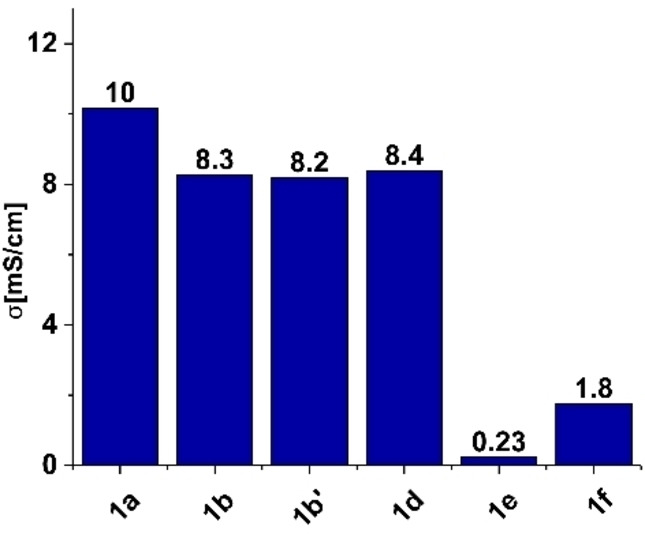
Electrolyte conductivity of 1 M sodium borate electrolytes (EC:DEC 1 : 1 v/v). The concentration of electrolyte **1 e** is 0.5 M due to lower solubility. Cell constant (K) is 15.5 cm^−1^ at *T*=30–35 °C using impedance spectroscopy with a frequency range of 1 MHz–1 Hz and a 10 mV amplitude.

Conductivity measurements were first undertaken as a method of screening borate salts **1 a**–**1 f** (Figure 4), where maximum conductivity was found using a 1 M solution of Na[B(hfip)_4_]⋅DME (**1 a**), giving a value of 10 mS cm^−1^. This compares to values of 8.3 and 8.2 mS cm^−1^ for 1 M solutions of Na[B(pp)_2_]⋅3 DME (**1 b**) and Na[B(pp)_2_] (**1 b′**) respectively. The phenoxy derived borate salts Na[B(OPh^F^)_4_]⋅3 DME (**1 d**) and Na[B(OPh)_4_] (**1 f**) gave conductivity values of 8.4 and 1.8 mS cm^−1^ respectively. The values for Na[B(OPh^F^)_4_]⋅3 DME and Na[B(OPh)_4_] highlight the effect of fluorination in these salts, given the much larger conductivity of the former. Note, the use of three DME molecules solvating the sodium cation in **1 b** and **1 d** was determined from their solid‐state structure (for further discussion on DME solvation see Supporting Information 5.7).

For Na[B(OMe)_4_] (**1 e**), the solubility in EC:DEC (1 : 1 v/v) was low, where only a 0.5 M solution could be prepared and a corresponding conductivity of 0.23 mS cm^−1^ was recorded. Lastly, during conductivity measurements it was found that Na[B(OCH_2_(CF_2_)_2_CF_3_)_4_] (**1 c**) is poorly soluble in EC:DEC (1 : 1 v/v) solvent, as even a 0.25 M solution could not be prepared, despite leaving for 48 hours. Hence, conductivity and further electrochemical measurements were not recorded for this salt.

**Figure 5:**
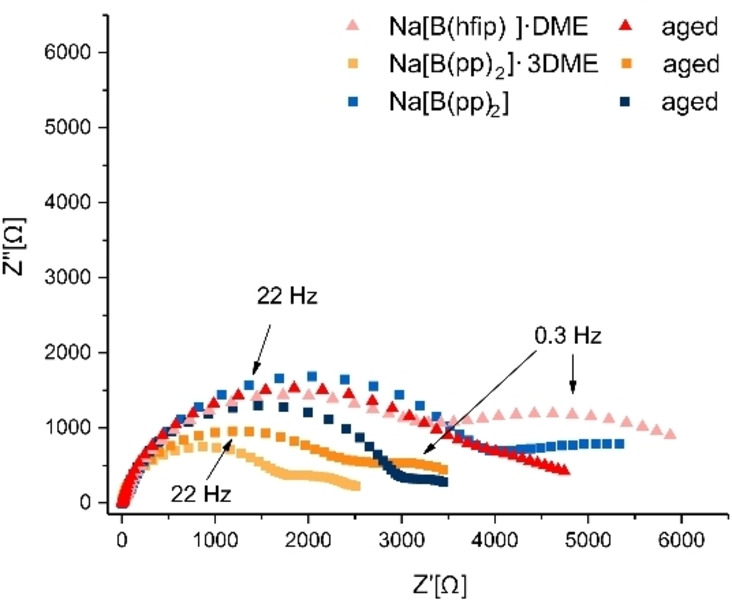
EIS Nyquist plots of fresh and aged Na[B(hfip)_4_]⋅DME (**1 a**) (red), Na[B(pp)_2_]⋅3 DME (**1 b**) (orange) and Na[B(pp)_2_] (**1b′**) (blue) using impedance spectroscopy with a frequency range of 1 MHz–0.1 Hz and a 10 mV amplitude. The frequencies at the semicircles’ maxima are indicated for Na[B(hfip)_4_]⋅DME and Na[B(pp)_2_]⋅3 DME aged. High frequency semicircle (left): maximum at 22 Hz, low frequency semicircle (right): maximum at 0.3 Hz (see Supporting Information for circle fitting details).

The impedance evolution of the native solid‐electrolyte interphase (SEI) was then measured in Na−Na symmetric cells using EIS measurements (Figures 5 and S5.5.1–S5.5.4). Impedance spectra were recorded for the fresh cells and 80 hours after cell assembly. Nyquist plots of fresh and aged Na[B(hfip)_4_]⋅DME (**1 a**), Na[B(pp)_2_]⋅3 DME (**1 b**) and Na[B(pp)_2_] (**1 b′**) are presented in Figure 5.

**Figure 6:**
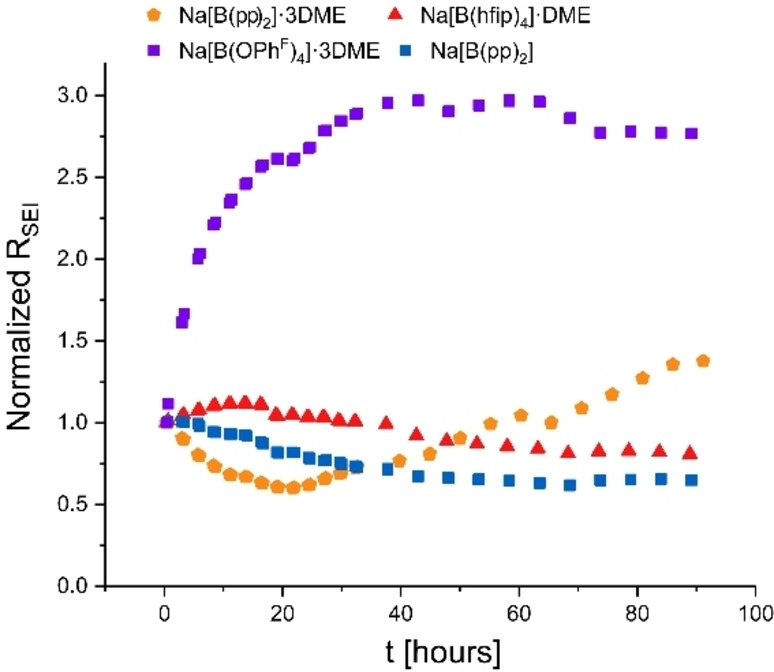
Evolution of the normalized R_SEI_ at 0.1 Hz vs. time. Na[B(hfip)_4_]⋅DME (**1 a**) (red), Na[B(pp)_2_]⋅3 DME (**1 b**) (orange), Na[B(pp)_2_] (**1 b′**) (blue), and Na[B(OPh^F^)_4_]⋅3 DME (**1 d**) (purple).

The EIS spectra are composed of two semicircles, with maxima at 22 Hz and 0.3 Hz; these are typical maximum semicircle frequencies for the unstable and porous Na‐SEI.^[51]^ The higher‐frequency semicircle, fitted with a capacitance on the order of μF, is typically attributed to the ionic transport though the interface, while the lower frequency semicircle, fitted with a capacitance on the order of nF, is attributed to ion transport in the grain boundaries of the SEI (see Supporting Information for the EIS fitting parameters). It is assumed that the main contribution to the low‐frequency impedance is the sodium SEI. Thus, the evolution of the low‐frequency (0.1 Hz) impedance (denoted R_SEI_) is attributed to the ageing, reorganization and dissolution of the SEI during rest (Figure 6). The R_SEI_ for freshly assembled cells is between 2500–8000 Ω, equivalent to the conductivity of 0.2–0.5 μS cm^−1^.

The interface stabilization rate is similar for Na[B(pp)_2_] (**1 b′**) and Na[B(hfip)_4_]⋅DME (**1 a**) electrolytes; their impedance after 80 hours of storage stabilizes around 5000 Ω (0.2 μS cm^−1^). However, the interface stabilization is slower for Na[B(pp)_2_]⋅3 DME (**1 b**) (not stabilized during 80 hour experiment), with its impedance around 2500 Ω (0.5μS cm^−1^) after 90 hours. The SEI impedance decreased for Na[B(hfip)_4_]⋅DME and Na[B(pp)_2_] cells, but increased for Na[B(pp)_2_]⋅3 DME cells with respect to storage time (Figures 6, S5.5.1–S5.5.3). We postulate the decrease in impedance for Na[B(pp)_2_]⋅3 DME in the first 20 hours is due to SEI chemical dissolution, while the increase in impedance after approximately 20 hours is a result of partial electrolyte consumption and formation of a thicker SEI. This decreases the solubility of the SEI, however doesn't impede the constant SEI formation which results in cell degradation.

Additionally, EIS measurements were performed on the phenoxy borate salts Na[B(OPh^F^)_4_]⋅3 DME (**1 d**) and Na[B(OPh)_4_] (**1 f**). The SEI impedance for the electrolytes grows rapidly after cell assembly and stabilizes at approximately threefold (Figure 6) and tenfold (Figure S5.5.4) the initial impedance, respectively, after 38 hours. This striking difference in the growth rate of the impedance of the SEI demonstrates the commonly‐accepted superiority of the SEI formed in fluorinated electrolyte solutions. In addition, these vastly different SEI evolution trends suggest that the anion has a major effect of SEI solubility and degradation mechanism.

**Figure 7:**
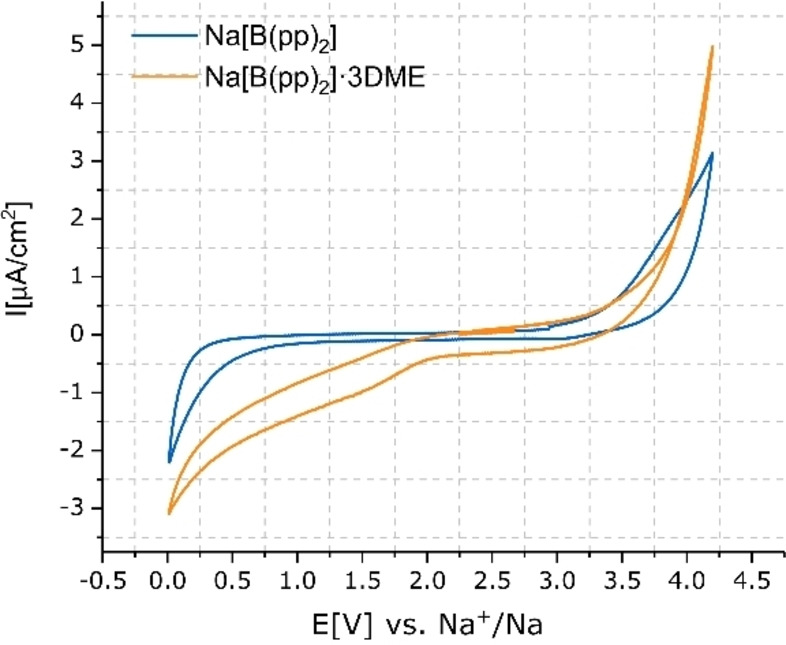
Cyclic voltammetry of 1 M Na[B(pp)_2_]⋅3 DME (**1 b**) and Na[B(pp)_2_] (**1 b′**) (EC:DEC 1 : 1 v/v) in three‐electrode cell (WE‐ aluminum, CE‐ sodium metal, RE‐ sodium metal). 3rd cycle, measured at 5 mV s^−1^ between 0.01 V and 4.2 V.

**Figure 8:**
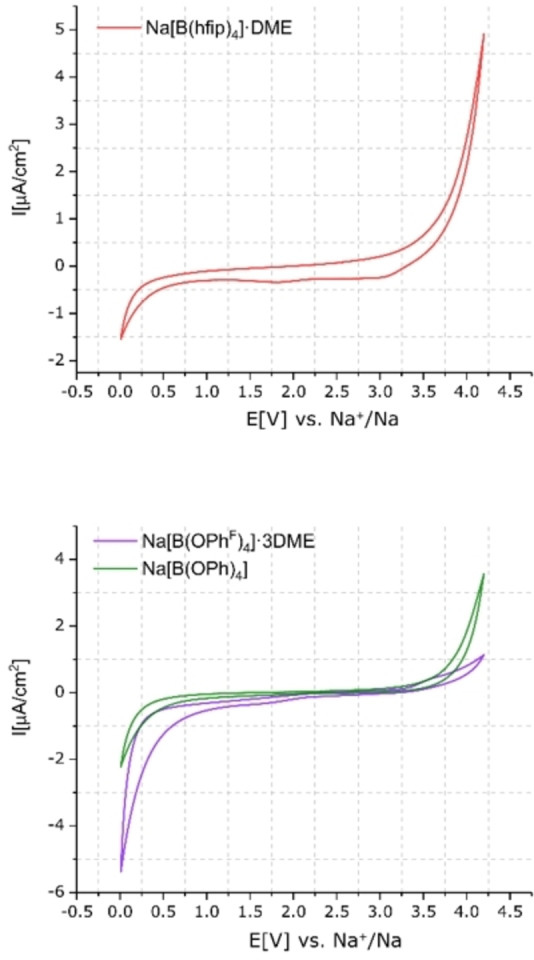
Cyclic voltammetry of Na[B(hfip)_4_]⋅DME (**1 a**) (top) and Na[B(OPh^F^)_4_]⋅3 DME (**1 d**) and Na[B(OPh)_4_] (**1 f**) (bottom) (EC:DEC 1 : 1 v/v) in three‐electrode cell (WE‐ aluminum, CE‐ sodium metal, RE‐ sodium metal). 3rd cycle, measured at 5 mV s^−1^ between 0.01 V and 4.2 V.

The results from both conductivity and EIS demonstrated that, with the exception of Na[B(OCH_2_(CF_2_)_2_CF_3_)_4_] (**1 c**) and Na[B(OMe)_4_] (**1 e**), which exhibit low solubility and conductivity, all the borate salts showed potential to act as electrolytes for SIBs. Consequently, these salts were further investigated and the ESW of Na[B(hfip)_4_]⋅DME (**1 a**), Na[B(pp)_2_]⋅3 DME (**1 b**), Na[B(pp)_2_] (**1 b′**), Na[B(OPh^F^)_4_]⋅3 DME (**1 d**) and Na[B(OPh)_4_] (**1 f**) were determined using CV. Electrolyte solutions at 1 M concentration in EC:DEC (1 : 1 v/v) were tested in three‐electrode cells using aluminium as the working electrode (WE); sodium metal was used as the counter (CE) and quasi‐reference electrode (RE) (Figures 7 and 8). The current density of both oxidation (>4 V) and reduction (<1 V) waves decrease with the cycle number (shown for Na[B(pp)_2_] in Figure S5.5.6).

The CV measurements of electrolytes Na[B(hfip)_4_]⋅DME (**1 a**) (Figure 8, top), Na[B(pp)_2_]⋅3 DME (**1 b**) and Na[B(pp)_2_] (**1 b′**) (Figure 7) vs. aluminium showed that the measured oxidation currents are approximately two times lower for the electrolyte solution of Na[B(pp)_2_] compared to Na[B(hfip)_4_]⋅DME and Na[B(pp)_2_]⋅3 DME. The estimated oxidation threshold (assumed in this work to be at the potential at which the current is a quarter of the maximum oxidation current at 4.2 V) is approximately 3.7 V vs. Na^+^/Na for electrolytes Na[B(hfip)_4_]⋅DME, Na[B(pp)_2_]⋅3 DME and Na[B(pp)_2_]. For the phenoxy borate salts Na[B(OPh^F^)_4_]⋅3 DME (**1 d**) and Na[B(OPh)_4_] (**1 f**) (Figure 8, bottom) the estimated oxidation threshold is lower, at 3.45 V vs. Na^+^/Na. While the oxidation potential is solely a property of the electrolyte (solvent, salt, additives and impurities), the magnitude of the oxidation current can be attributed to both the extent of the oxidation reaction and the quality of the passivation of the Al current collector (WE).

The slope of the oxidation wave is generally attributed to the conductivity of the electrolyte. Notably, the oxidation waves are sharp for Na[B(hfip)_4_]⋅DME (**1 a**) and Na[B(pp)_2_]⋅3 DME (**1 b**), moderate for Na[B(pp)_2_] (**1 b′**), and less steep for Na[B(OPh^F^)_4_]⋅3 DME (**1 d**) and Na[B(OPh)_4_] (**1 f**). This trend is consistent with that observed for the SEI conductivity but not with the bulk electrolyte conductivity, demonstrating the stronger dependence between passivation and current collector reactivity.

Comparing the electrochemical stability of the different salts, Na[B(pp)_2_] (**1 b′**) was found to be the most stable versus aluminum current collectors, compared to Na[B(hfip)_4_]⋅DME (**1 a**) and Na[B(pp)_2_]⋅3 DME (**1 b**), suggesting that the presence of DME has a detrimental effect. To explore this effect, the CV plots for Na[B(pp)_2_]⋅3 DME and Na[B(pp)_2_] in EC:DEC (1 : 1 v/v) were compared with the plot for 1 M Na[B(pp)_2_] in DME solvent (Figure S5.5.5). A distinct oxidation peak around 3.6 V was observed for the DME‐based Na[B(pp)_2_] electrolyte. The same peak was not found in the CV of Na[B(hfip)_4_]⋅DME, and appeared as a minimal peak in the CV plot of Na[B(OPh^F^)_4_]⋅3 DME (**1 d**), possibly due to less DME content in the former and lower conductivity for the latter.

The magnitude of the oxidation current in the CV plot is correlated to the oxidation reaction rate. A higher rate of electrolyte oxidation will result in extensive electrolyte consumption and battery degradation, hence shorter battery life. The tested sodium borate electrolytes gave approximately an order of magnitude lower oxidation current peaks compared to NaPF_6_ at the same concentration and same solvent system (EC:DEC 1 : 1 v/v), which cannot be explained by the ratio of the bulk conductivities alone.^[13]^

To investigate the findings observed from the conductivity and CV measurements, density functional theory (DFT) calculations were employed to assess the ion‐pair dissociation energies and oxidation potentials of salts **1 a**–**1 g**. Ion‐pair dissociation energies were calculated by automatically generating a large number of ion‐pairs using SECIL.^[52]^ In each case, the initial optimization pass was done with xtb version 6.4.0.[^53^, ^54^] Selected ion‐pairs (on basis of having distinct structures and energies) were then optimized at B3LYP‐D/def2‐TZVPP level of theory within ORCA;[^55^, ^56^, ^57^, ^58^, ^59^] the ion‐pair dissociation energy was calculated from the final energetic values (full details in experimental). Oxidation potentials were calculated by taking the final anion structure and doing a single point energy calculation on the corresponding oxidized system. All calculations are in the gas phase.

A lower ion‐pair dissociation energy means that the solvation of an ion‐pair into an electrolyte should be easier, assuming similar solvation energies for the anions.[^60^, ^61^] The results from Table 1 show that the fluorinated borate salts have lower dissociation energies (with a bare Na^+^ cation) than their non‐fluorinated analogues ([B(hfip)_4_]^−^ vs. [B(O^i^Pr)_4_]^−^ and [B(OPh^F^)_4_]^−^ vs. [B(OPh)_4_]^−^), thus suggesting that the fluorinated versions will have greater solubility and less ion‐pairing in solution. This prediction is consistent with our experimental preparation of electrolyte solution of the salts. The [B(pp)_2_]^−^ anion was found to have the lowest dissociation energy, at 448 kJ mol^−1^, although the difference in dissociation energies between [B(pp)_2_]^−^ and the anions [B(hfip)_4_]^−^ (472 kJ mol^−1^) and [B(OPh^F^)_4_]^−^ (461 kJ mol^−1^) is small. Notably, the [B(OMe)_4_]^−^ anion has the highest ion‐pair dissociation energy and has markedly poorer bulk conductivity.

**Table 1:** Ion‐pair dissociation energy (reported as Gibbs free energy) and oxidation potential (V vs. Na^+^/Na) for sodium borate salts.

Anion	Dissociation energy [kJ mol^−1^]	Oxidation Potential vs. Na^+^/Na^V^
[B(hfip)_4_]^−^	472	4.79
[B(pp)_2_]^−^	448	4.63
[B(OCH_2_(CF_2_)_2_CF_3_)_4_]^−^	484	4.36
[B(OPh^F^)_4_]^−^	461	3.40
[B(OMe)_4_]^−^	579	2.06
[B(OPh)_4_]^−^	496	2.18
[B(O^i^Pr)_4_]^−^	560	2.09

The predicted oxidation potentials vs. Na^+^/Na of sodium borate salts, calculated by subtracting 1.73 V from the absolute potential values, are presented in Table 1. Comparing the calculated and experimental oxidation potentials, the calculated potentials are notably higher for [B(hfip)_4_] and [B(pp)_2_]^−^, similar for [B(OPh^F^)_4_]^−^ and lower for [B(OPh)_4_]^−^, with respect to the experimental values from CV. These trends reflect faster oxidation kinetics on the aluminium metal‐electrolyte interface and the apparent improved stability in the non‐fluorinated poorer conducting electrolytes.

From the calculated oxidation potentials, the effect of fluorination is clear. This is shown by the higher DFT calculated potentials (referenced against Na^+^/Na) for the anions [B(hfip)_4_]^−^ and [B(OPh^F^)_4_]^−^, compared to their respective non‐fluorinated analogues [B(O^i^Pr)_4_]^−^ and [B(OPh)_4_]^−^. Thus, having a higher level of fluorination increases oxidative stability. Additionally, the fluoroalkyl‐derived anions all have predicted higher oxidative stability than the perfluorophenyl borate anion. The non‐fluorinated anion [B(OMe)_4_]^−^ has the lowest predicted oxidative stability, at 2.06 V vs. Na^+^/Na.

After CV measurements, cycling of 1 M electrolyte solutions of Na[B(hfip)_4_]⋅DME (**1 a**), Na[B(pp)_2_]⋅3 DME (**1 b**), Na[B(pp)_2_] (**1 b′**), Na[B(OPh^F^)_4_]⋅3 DME (**1 d**) and Na[B(OPh)_4_] (**1 f**) in sodium‐ion battery coin cells was undertaken. The anode active material was a commercially available hard carbon and Na_(0.79±0.05)_[Ni_(0.27±0.05)_Mn_(0.42±0.05)_Mg_(0.15±0.05)_Ti_(0.17±0.05)_]O_(2±0.05)_ cathode active material was sourced from Haldor‐Topsoe A/S, synthesized according to Faradion's specification for a mixed phase O3/P2 layered oxide (Faradion Ltd).[^62^, ^63^]

**Figure 9:**
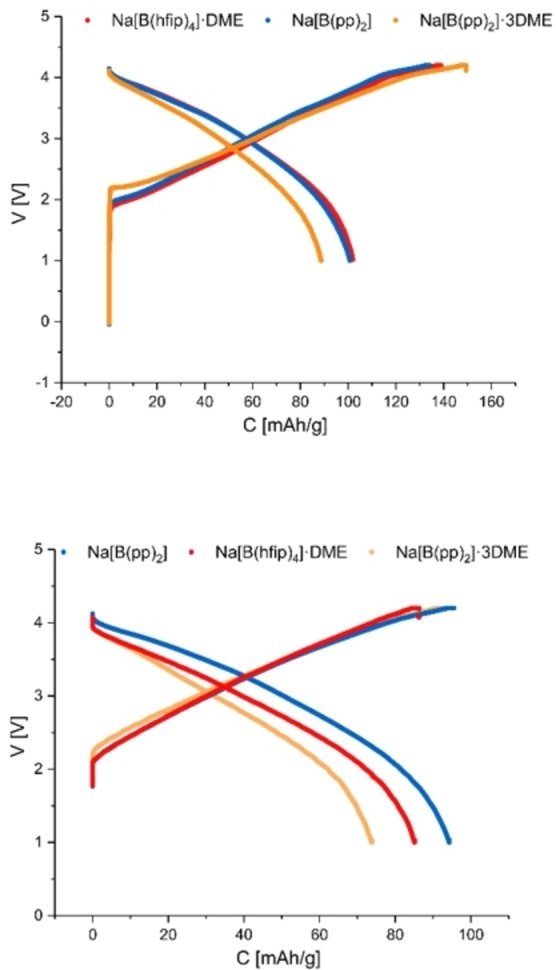
Top: Cell voltage (V) vs. gravimetric specific capacity (C), collected from first cycle. Bottom: Cell voltage (V) vs. gravimetric specific capacity, collected from 10^th^ cycle. Approximate constant current rate of C/5 for charge and discharge using cell voltage limits of 1.0 and 4.2 V. Electrolyte is 1 M Na[B(hfip)_4_]⋅DME (**1 a**), Na[B(pp)_2_]⋅3 DME (**1 b**) and Na[B(pp)_2_] (**1 b′**) salts in EC:DEC (1 : 1 v/v), measured in coin cells.

The cells were cycled for ten cycles at a rate of C/5. The discharge capacities of the cells in the 1^st^ and 10^th^ cycle were compared under the same conditions (full details in experimental), which showed the capacity of the Na[B(pp)_2_] (**1 b′**) cell is similar to that of Na[B(hfip)_4_]⋅DME (**1 a**), while the cell containing Na[B(pp)_2_]⋅3 DME (**1 b**) has 10 % less capacity (Figures 9 top and bottom and 10). Moreover, degradation between cycle three and ten is significantly reduced for Na[B(hfip)_4_]⋅DME and Na[B(pp)_2_], compared to Na[B(pp)_2_]⋅3 DME. Notably, the charge curve for Na[B(pp)_2_]⋅3 DME has a distinctive feature around 2.3 V, possibly indicating increased degradation and cell polarization.

**Figure 10:**
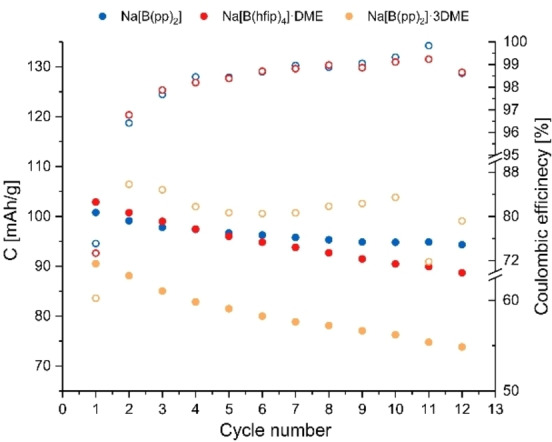
Discharge gravimetric capacity (filled circles) and efficiency (non‐filled circles) vs. cycle number collected from the first 12 cycles at an approximate constant current rate of C/5 for charge and discharge using cell voltage limits of 1.0 and 4.2 V. Electrolyte is 1 M Na[B(hfip)_4_]⋅DME (**1 a**), Na[B(pp)_2_]⋅3 DME (**1 b**) and Na[B(pp)_2_] (**1 b′**) salts in EC:DEC (1 : 1 v/v), measured in coin cells.

Formation cycles and early cycle life of Na[B(hfip)_4_]⋅DME (**1 a**), Na[B(pp)_2_]⋅3 DME (**1 b**) and Na[B(pp)_2_] (**1 b′**) cells are shown in Figure 10. The degradation rate of Na[B(pp)_2_]⋅3 DME and Na[B(hfip)_4_]⋅DME is similar, while the initial reversible capacity is lower by approximately 10 % for Na[B(pp)_2_]⋅3 DME. The degradation rate of Na[B(pp)_2_] is notably reduced compared to Na[B(pp)_2_]⋅3 DME and Na[B(hfip)_4_]⋅DME, suggesting the presence of DME solvent is detrimental to the overall cell performance.

To further explain the difference in degradation rate between Na[B(hfip)_4_]⋅DME (**1 a**), Na[B(pp)_2_]⋅3 DME (**1 b**) and Na[B(pp)_2_] (**1 b′**), solution‐state NMR spectroscopy was used to investigate changes in the electrolyte solutions upon cycling. Cycled coin cells were opened in an inert atmosphere and the electrolyte was extracted from the separator using DMSO‐*d*_6_ as the NMR solvent. Integration of the signals relative to the EC signal in the ^1^H NMR spectra revealed a decrease in the amount of DEC for all electrolyte solutions and a decrease in the amount of DME for Na[B(hfip)_4_]⋅DME and Na[B(pp)_2_]⋅3 DME. The intensity of the DME signals are reduced by approximately 65 % and 56 % for Na[B(hfip)_4_]⋅DME and Na[B(pp)_2_]⋅3 DME, respectively. The loss of DEC is likely a result of evaporation during sample preparation, while the loss of the DME molecules indicates that they are consumed upon cycling. The consumption of DME could result in a more poorly passivating SEI which leads to capacity fade over time or its oxidation on the cathode‐electrolyte interface. Since DME undergoes oxidation at approximately 3.2 V vs. Na^+^/Na (as shown in Figure S5.5.5), its oxidation is likely to occur during the Na‐ion cell cycling.

The cycling performance of sodium‐ion coin cells made using Na[B(OPh^F^)_4_]⋅3 DME (**1 d**) and Na[B(OPh)_4_] (**1 f**) based electrolytes was noticeably poorer than those containing Na[B(hfip)_4_]⋅DME (**1 a**), Na[B(pp)_2_]⋅3 DME (**1 b**) and Na[B(pp)_2_] (**1 b′**). The irreversible capacity measured for Na[B(OPh)_4_] electrolyte was 80 %, compared to approximately 30 % for all other measured cells with fluorinated salts. Cells using Na[B(OPh^F^)_4_]⋅3 DME failed to charge beyond 3.9 V, possibly due to a parasitic reaction that resulted in a potential plateau that continued for over five hours (Figure S5.5.7).

Cycle life for electrolytes Na[B(hfip)_4_]⋅DME (**1 a**), Na[B(pp)_2_]⋅3 DME (**1 b**) and Na[B(pp)_2_] (**1 b′**) were investigated using commercial 2‐electrode pouch cells prepared by Faradion Limited, UK. 1 M electrolyte solutions of these salts were prepared in an organic solvent blend of EC, DEC and propylene carbonate (PC) (1 : 2 : 1 wt/wt). The same anode and cathode materials as used for the coin cells were employed. The results of this showed that all three electrolytes were stable throughout the performed 90 cycles at a rate of C/5. Electrolytes Na[B(hfip)_4_]⋅DME and Na[B(pp)_2_] showed minimal capacity loss during these cycles. On the other hand, Na[B(pp)_2_]⋅3 DME exhibited much higher capacity loss as well as cycling at a lower capacity (Figure S7.2.2). The difference in cycling performance between Na[B(pp)_2_] and Na[B(pp)_2_]⋅3 DME is consistent with observations from coin cells and again highlights the detrimental effect of DME.

Comparing the cycle life of Na[B(hfip)_4_]⋅DME (**1 a**) and Na[B(pp)_2_] (**1 b′**) to 1 M NaPF_6_ (EC:DEC:PC, 1 : 2 : 1 wt/wt) in 2‐electrode pouch cells, Na[B(hfip)_4_]⋅DME and Na[B(pp)_2_] exhibited greater cycling stability, with NaPF_6_ having a higher drop‐off in capacity (Figure S7.2.2). The initial cycle capacities of Na[B(hfip)_4_]⋅DME and Na[B(pp)_2_] (**1 b′**) were comparable to NaPF_6_, albeit slightly higher for NaPF_6_. Further electrochemical comparisons to NaPF_6_ are given in Supporting Information S5.8.

Lastly, to evaluate the suitability of borate salts Na[B(hfip)_4_]⋅DME (**1 a**), Na[B(pp)_2_]⋅3 DME (**1 b**) and Na[B(pp)_2_] (**1 b′**) further as electrolytes for SIBs, differential scanning calorimetry (DSC) was employed to determine their freezing point temperature in EC:DEC (1 : 1 v/v) at 1 M concentration. This is important for electrolyte characterization because it dictates the lowest battery operating temperature. For this, a 20 μL electrolyte sample of **1 a**–**1 b′** was cooled from 25 °C to −40 °C at a rate of 5 °C min^−1^ under nitrogen (Figure S4.3.2), with the DSC trace of EC:DEC (1 : 1 v/v) solvent showing the solvent freezes at −12.4 °C. Addition of the salts **1 a**–**1 b′** suppresses this temperature, with electrolyte solution Na[B(hfip)_4_]⋅DME giving an exothermic peak at −29.5 °C in the DSC trace, corresponding to the solution freezing, whereas Na[B(pp)_2_]⋅3 DME and Na[B(pp)_2_] electrolyte solutions had exothermic peaks at −36.7 °C and −27.1 °C, respectively.

Having employed multiple techniques to investigate the chemical, thermal and electrochemical properties of the seven synthesized sodium borate salts, an evaluation can be made regarding their suitability to act as electrolyte salts for SIBs. Firstly, the low solubility of Na[B(OCH_2_(CF_2_)_2_CF_3_)_4_] (**1 c**) prevented electrochemical measurements, whilst the low solubility and low conductivity of Na[B(OMe)_4_] (**1 e**) rules it out as an effective electrolyte salt. From the remaining salts, the phenoxy‐derived borates Na[B(OPh^F^)_4_]⋅3 DME (**1 d**) and Na[B(OPh)_4_] (**1 f**) have the lowest tolerance to air and water, though Na[B(OPh^F^)_4_]⋅3 DME does exhibit good bulk conductivity (8.4 mS cm^−1^). In addition, both Na[B(OPh^F^)_4_]⋅3 DME and Na[B(OPh)_4_] gave poor cycling performance, with the former failing to charge beyond 3.9 V and the latter giving a high irreversible capacity.

Na[B(hfip)_4_]⋅DME (**1 a**) demonstrates excellent electrochemical stability, having the highest bulk conductivity of 10 mS cm^−1^ in EC:DEC (1 : 1 v/v) solvent. The cycling performance of Na[B(hfip)_4_]⋅DME is comparable to Na[B(pp)_2_] (**1 b′**), which also exhibits high electrochemical stability. Furthermore, 2‐electrode commercial pouch cell results showed both electrolytes give greater cycling stability compared to 1 M NaPF_6_ and cycle at comparable capacity (Figure S7.2.2). The improved SEI stability in DME‐free Na[B(pp)_2_] electrolyte is key for its superior cyclability. This greater stability is demonstrated via three electrochemical methods (EIS, CV and chronoamperometry). Notably, we find that the [B(pp)_2_]^−^ anion does not undergo hydrolysis or decomposition when exposed to air for two days or addition of ten equivalents of water in CD_3_CN solution. Comparison of the electrochemical performance of Na[B(pp)_2_] to Na[B(pp)_2_]⋅3 DME (**1 b**) shows that the presence of DME solvation is detrimental to the overall cells’ performance.

In addition, this study has looked at comparing the role of fluorination in electrolyte salts, by synthesizing both fluorinated and non‐fluorinated analogues. DFT calculations show the ion‐pair dissociation energies are higher for the non‐fluorinated borate salts, which is in agreement with our experimental observations of lower solubility and lower conductivity for these complexes compared to their fluorinated counterparts.

Lastly, we have found using CV experiments that sodium undergoes plating and stripping in Na[B(hfip)_4_]⋅DME (**1 a**) and Na[B(pp)_2_] (**1 b′**) (see Supporting Information S5.9). As a consequence, “anode‐free” sodium metal batteries are now the focus of our future research efforts.

## Conclusion

A series of sodium borate salts with varying steric and electronic properties have been prepared. Synthesis involved either reacting sodium borohydride with a fluorinated alcohol in 1,2‐dimethoxyethane solvent, or the addition of a tricoordinate borate to the corresponding sodium alkoxide in THF solvent. A range of experimental and theoretical techniques was employed to evaluate how suitable the sodium borate complexes are to act as electrolyte salts, with Na[B(hfip)_4_]⋅DME (**1 a**) and Na[B(pp)_2_] (**1 b′**) being found to have superior electrochemistry. In addition, the [B(pp)_2_]^−^ anion exhibits high chemical stability and was found to be bench stable when left open to air for two days, allowing for convenient handling, transport and storage. Lastly, commercial pouch cells cycled with Na[B(hfip)_4_]⋅DME and Na[B(pp)_2_] with standard, commercial electrode materials shows comparable capacity and greater cycling stability than cells prepared with the conventionally used but toxic NaPF_6_ salt. Overall, these two borate salts have multiple significant advantages over NaPF_6_ based electrolyte for use in commercially relevant SIBs.

## Conflict of interest

The authors declare no conflict of interest.

1

## Supporting information

As a service to our authors and readers, this journal provides supporting information supplied by the authors. Such materials are peer reviewed and may be re‐organized for online delivery, but are not copy‐edited or typeset. Technical support issues arising from supporting information (other than missing files) should be addressed to the authors.

Supporting Information

Supporting Information

Supporting Information

## Data Availability

The data that support the findings of this study are available in the Supporting Information of this article.
