# Supplementary material for: Sodium Borates: Expanding the Electrolyte Selection for Sodium‐Ion Batteries
Source: Angew Chem Int Ed Engl. 2022 May 3;61(32):e202202133. doi: 10.1002/anie.202202133 (PMC9401571; doi:10.1002/anie.202202133)

Angewandte Chemie
Eine Zeitschrift der Gesellschaft Deutscher Chemiker

Supporting Information

Sodium Borates: Expanding the Electrolyte Selection for Sodium-Ion Batteries

D. M. C. Ould, S. Menkin, H. E. Smith, V. Riesgo-Gonzalez, E. Jónsson, C. A. O'Keefe, F. Coowar, J. Barker, A. D. Bond, C. P. Grey, D. S. Wright**

Supplementary Information

Contents

S1 Experimental procedure and compound characterisation. 2
S2 Stability of sodium borate to air and water. 8
S3 Single crystal X-ray diffraction. 11
S4 Thermal stability plots and analysis. 15
S5 Electrochemistry of sodium borate salts. 20
S6 Solution-state NMR spectroscopy of electrolytes after cycling. 31
S7 Commercial 2- and 3-elctrode pouch cell cycling. 35
S8 Density functional theory (DFT). 39
S9 References. 40
S10 NMR spectra. 41

S1 Experimental procedure and compound characterisation.

S1.1 General experimental.

Unless stated otherwise, all reactions were carried out using standard Schlenk (under a dinitrogen atmosphere) and glove box (under argon; Saffron, Alpha model) techniques. Dimethoxyethane (DME) and methanol solvents were dried over 4 Å activated molecular sieves for 24 hours and stored in an ampoule fitted with a Teflon valve under a dinitrogen atmosphere. THF was collected freshly distilled over sodium wire/benzophenone and pentane was collected freshly distilled over calcium hydride. Both solvents were then stored over activated 4 Å molecular sieves in an ampoule fitted with a Teflon valve under a dinitrogen atmosphere. Deuterated solvents were dried over 4 Å activated molecular sieves and stored in an argon filled glovebox. Starting materials were purchased from commercial suppliers. Alcohols were dried over 4 Å activated molecular sieves for 24 hours and stored over molecular sieves in an ampoule fitted with a Teflon valve under a dinitrogen atmosphere. $NaPF_6$ was prepared using a literature procedure.[1]

^{1}H, $^{13}C\{^{1}H\}$, ^{19}F, $^{19}F\{^{1}H\}$ and ^{11}B solution-state NMR spectra were recorded at 298 K on a Bruker 400 MHz AVIII HD Smart Probe spectrometer. Chemical shifts are expressed as parts per million (ppm, δ) and are referenced to either CD_3CN (1.95/118.26 ppm) or $(CD_3)_2SO$ (2.50/39.52 ppm) as internal standards. Multinuclear NMR spectra were referenced to $BF_3{\cdot}Et_2O/CDCl_3$ (^{11}B) and $CFCl_3$ (^{19}F). The description of signals includes s = singlet, d = doublet, dd = doublet of doublets, t = triplet, tt= triplet of triplets, q = quartet, quin = quintet, m = multiplet, ov = overlapping and br = broad. All coupling constants are absolute values and are expressed in Hertz (Hz). IR-Spectra were measured on a Shimadzu IR Affinity-1 photospectrometer. The description of signals includes s = strong, m = medium, w = weak, vw = very weak, sh = shoulder, and br = broad. High-resolution mass spectra (HRMS) were collected by the School of Chemistry in University of Cambridge using a Waters Xevo G2-S QTOF mass spectrometer in negative mode. Elemental microanalytical data were obtained from the University of Cambridge, Department of Chemistry microanalytical service, using a PerkinElmer 240 Elemental Analyser.

Ethylene carbonate: diethyl carbonate (EC:DEC 1:1 v/v) was prepared by weighing a known amount of EC and translating this to a volume using the density 1.321 g/mL. The equal volume of DEC was added; gentle heating to 50 °C was required to fully dissolve EC. The 1:1 by volume ratio was confirmed by integrating the EC and DEC signals in the ^{1}H NMR spectrum. The prepared solvent was degassed using freeze-pump-thaw degas technique and dried over 4 Å activated molecular sieves to ca. 10 ppm water (determined by Karl-Fischer titration).

S1.2 Synthesis of sodium borate compounds.

General Synthetic procedure 1:

A Schlenk tube was charged with sodium borohydride (1.0 equiv) and 1,2-dimethoxyethane (DME, ca. 20 mL). The solution was cooled to 0 °C and the mono substituted fluorinated alcohol (4.3 equiv) or diol (2.3 equiv) was added dropwise. The evolution of dihydrogen was immediately observed with vigorous effervescence. The reaction was warmed to room temperature, then heated to reflux for six hours. After this the reaction was cooled to 50 °C and left to stir for 16 hours. The reaction was left to cool to ambient temperature and the solvent level was reduced to approximately one third *in vacuo* (until precipitation starts to occur). Pentane (40 mL) was added, which precipitated the product out, and the resulting precipitate was cooled to −30 °C for 3 hours. The precipitate was isolated by removing the solvent by syringe. This purification procedure was repeated, with DME (ca. 4 mL) added to dissolve the sodium borate product, followed by pentane (40 mL) to precipitate the product. After solvent removal by syringe, the product was then washed with pentane (3 × 10 mL). Drying *in vacuo* afforded the sodium borate salts **1a**–**1d** as a white powder.

Synthesis of sodium tetrakis(hexafluoroisopropyloxy)borate, Na[B(hfip)$_4$]·DME (**1a**):

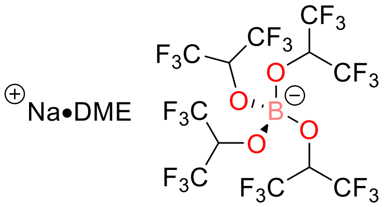

Compound **1a** was synthesised according to general procedure 1 using sodium borohydride (407 mg, 10.8 mmol, 1.0 equiv) and 1,1,1,3,3,3-hexafluoro-2-propanol (4.9 mL, 46.3 mmol, 4.3 equiv). At this stage ^{1}H NMR spectroscopy shows that the formula of the product is Na[B(hfip)$_4$]·3DME. The product was further dried by heating to 90 °C *in vacuo* for ca. two hours and the ^{1}H NMR spectrum and elemental analysis indicates that the formula is Na[B(hfip)$_4$]·DME (consistent with the previously reported solid-state characterisation[2]). Note: the product is prone to subliming at higher temperatures *in vacuo*. Yield: 6.3 g, 8.0 mmol, 74% (based on Na[B(hfip)$_4$]·DME). Spectroscopic values in agreement with literature.[2]

^{1}H NMR (400 MHz, CD_3CN, 295 K) δ/ppm: 4.72 (br s, 4H, OC–H), 3.46 (s, 4H, DME–CH_2), 3.29 (s, 6H, DME–CH_3). **^{13}C{^{1}H} NMR** (101 MHz, CD_3CN, 295 K) δ/ppm: 128.1–119.6 (m, CF_3), 72.3 (s, DME–CH_2), 71.1–69.3 (m, CH), 58.9 (s, DME–CH_3). **^{11}B NMR** (128 MHz, CD_3CN, 295 K) δ/ppm: 1.7 (quin, $^3J_{BH}$ = 3.0 Hz). **^{19}F{^{1}H} NMR** (376 MHz, CD_3CN, 295 K) δ/ppm: −75.4 (s). **HRMS** (ASAP$^-$) *m/z* calculated for [M]$^-$ [$C_{12}H_4BO_4F_{24}$]$^-$: 678.9819 found: 678.9813. **IR** ν_{max} (cm^{-1}): 2947 (vw), 1457 (vw), 1366 (w), 1285 (w), 1208 (m), 1179 (s), 1167 (s), 1135 (sh), 1094 (s), 1077 (m), 1034 (m), 1007 (m), 928 (m), 889 (m), 880 (s), 743 (m) and 686 (s). **Anal**. Calcd for $C_{16}H_{14}BF_{24}NaO_6$ (Na[B(hfip)$_4$]·DME): C, 24.26; H, 1.78; N, 0.00. Found: C, 24.76; H, 2.00; N, 0.00.

Synthesis of sodium bis(perfluorinated pinacolato)borate, Na[B(pp)$_2$]·3DME (**1b**):

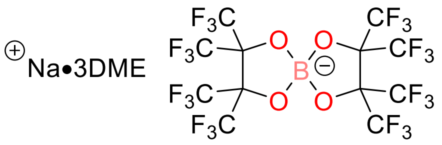

Compound **1b** was synthesised according to general procedure 1 using sodium borohydride (250 mg, 6.61 mmol, 1.0 equiv) and hexafluoro-2,3-bis(trifluoromethyl)-2,3-butanediol (5.1 g, 15.2 mmol, 2.3 equiv). Drying *in vacuo* for ca. 30 minutes results in some loss of the DME solvation, with elemental analysis indicating two DME molecules remain per formula unit. However, the solid-state structure showed three DME solvent molecules coordinate to the sodium cation. Yield: 4.4 g, 4.6 mmol, 69% (based on Na[B(pp)$_2$]·3DME). Note: Heating **1b** to 140 °C *in vacuo* for 48 hours removed the DME solvent, as detected by ^{1}H NMR spectroscopy. Spectroscopic values of the anion in agreement with literature.[3]

^{1}H NMR (400 MHz, CD_3CN, 295 K) δ/ppm: 3.46 (s, 4H, DME–CH_2), 3.29 (s, 6H, DME–CH_3). **^{13}C{^{1}H} NMR** (101 MHz, $(CD_3)_2SO$, 295 K) δ/ppm: 126.0–117.2 (m, CF_3), 85.2 (br s, O<u>C</u>(CF_3)$_2$), 71.2 (s, DME–CH_2), 58.0 (s, DME–CH_3). **^{11}B NMR** (128 MHz, CD_3CN, 295 K) δ/ppm: 11.4 (s). **^{19}F NMR** (376 MHz, CD_3CN, 295 K) δ/ppm: −70.4 (s). **HRMS** (ASAP$^-$) *m/z* calculated for $[M]^-$ $[C_{12}BO_4F_{24}]^-$: 674.9506 found: 674.9492. **IR** ν_{max} (cm^{-1}): 2946 (vw), 1476 (w), 1277 (w), 1262 (w), 1219 (m), 1193 (m), 1154 (w), 1123 (w), 1067 (w), 1026 (w), 975 (w), 930 (w), 859 (w), 742 (w), 721 (w) and 680 (w). **Anal.** Calcd for $C_{20}H_{20}BF_{24}NaO_8$ (Na[B(pp)$_2$]·2DME): C, 27.36; H, 2.30; N, 0.00. Found: C, 27.31; H, 2.53; N, 0.00.

Synthesis of sodium tetrakis(2,2,3,3,4,4,4-heptafluorobutoxy)borate, Na[B(OCH$_2$(CF$_2$)$_2$CF$_3$)$_4$] (**1c**):

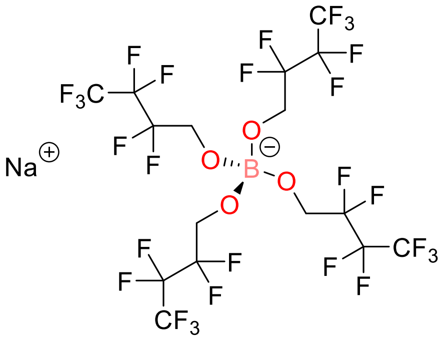

Compound **1c** was synthesised according to general procedure 1 using sodium borohydride (130 mg, 3.43 mmol, 1.0 equiv) and perfluoropropyl carbinol (1.9 mL, 14.8 mmol, 4.3 equiv). Yield: 2.3 g, 2.8 mmol, 81%.

^{1}H NMR (400 MHz, CD_3CN, 295 K) δ/ppm: 3.88 (t, $^3J_{FH}$ = 15.9 Hz, 4H, CH_2). **^{13}C{^{1}H} NMR** (101 MHz, $(CD_3)_2SO$, 295 K) δ/ppm: 122.3–105.8 (m, <u>C</u>F$_2$<u>C</u>F$_2$<u>C</u>F$_3$), 59.2 (t, $^2J_{FC}$ = 25.0 Hz, CH_2). **^{11}B NMR** (128 MHz, CD_3CN, 295 K) δ/ppm: 2.3 (s). **^{19}F{^{1}H} NMR** (376 MHz, CD_3CN, 295 K) δ/ppm: −82.0 (t, $^3J_{FF}$ = 9.3 Hz, 3F, CF_3), −122.4 (q, $^3J_{FF}$ = 9.3 Hz, 2F, CF_2), −128.6 (s, 2F, CF_2). **HRMS** (ASAP$^-$) *m/z* calculated for $[M]^-$ $[C_{16}H_8BO_4F_{28}]^-$: 807.0069 found: 807.0074. **IR** ν_{max} (cm^{-1}): 2927 (vw), 2884 (vw), 1463 (vw), 1411 (vw), 1345 (w), 1223 (s), 1170 (s), 1150 (s), 1120 (s), 1092 (s), 1053 (m), 1005 (s), 973 (s), 911 (s), 796 (w), 761 (m), 743 (m) and 669 (w). **Anal.** Calcd for $C_{16}H_8BF_{28}NaO_4$ (Na[B(OCH$_2$(CF$_2$)$_2$CF$_3$)$_4$]): C, 23.15; H, 0.97; N, 0.00. Found: C, 23.49; H, 0.89; N, 0.00.

Synthesis of sodium tetrakis(perfluorophenoxy)borate, Na[B(OPh^F)$_4$]·3DME (**1d**):

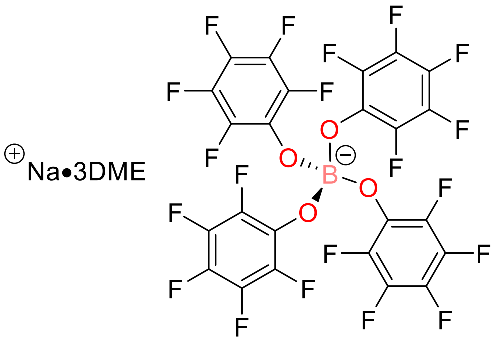

Compound **1d** was synthesised according to general procedure 1 using sodium borohydride (130 mg, 3.44 mmol, 1.0 equiv) and pentafluorophenol (1.6 mL, 15.3 mmol, 4.3 equiv). Note: due to pentafluorophenol being a solid at room temperature, the alcohol was gently warmed to 45 °C in a J. Youngs ampoule to melt the solid. Addition of the alcohol to sodium borohydride gave a yellow solution. Purification of the product required 2 × hot recrystallisation from DME solvent (ca. 3 mL). Drying *in vacuo* for ca. 30 minutes results in some loss of the DME solvation, with elemental analysis indicating one DME molecule remains per formula unit. However, the solid-state structure showed three DME solvent molecules coordinate to the sodium cation. Yield: 1.5 g, 1.4 mmol, 42% (based on Na[B(OPh^F)$_4$]·3DME). Spectroscopic values of the anion in agreement with literature.[4]

^{1}H NMR (400 MHz, CD_3CN, 295 K) δ/ppm: 3.46 (s, 4H, DME–CH_2), 3.29 (s, 6H, DME–CH_3). **^{13}C{^{1}H} NMR** (101 MHz, $(CD_3)_2SO$, 295 K) δ/ppm: 142.8–132.7 (m, Ar–F), 71.1 (s, DME–CH_2), 58.0 (s, DME–CH_3). **^{11}B NMR** (128 MHz, CD_3CN, 295 K) δ/ppm: 1.2 (s). **^{19}F{^{1}H} NMR** (376 MHz, CD_3CN, 295 K) δ/ppm: −158.8 (d, $^3J_{FF}$ = 20.0 Hz, 8F, Ar–F), −169.3 (ov dd, $^3J_{FF}$ = 20.0 Hz, 8F, Ar–F), −172.5 (ov tt, $^3J_{FF}$ = 20.0 Hz, 4F, Ar–F). **HRMS** (ASAP$^-$) *m/z* calculated for [M]$^-$ [$C_{24}BO_4F_{20}$]$^-$: 742.9570 found: 742.9557. **IR** ν_{max} (cm^{-1}): 1524 (s), 1505 (s), 1476 (m), 1320 (w), 1161 (m), 1023 (sh), 988 (s), 957 (m), 926 (s) and 792 (w). **Anal.** Calcd for $C_{28}H_{10}BF_{20}NaO_6$ (Na[B(OPh^F)$_4$]·DME): C, 39.28; H, 1.18; N, 0.00. Found: C, 39.14; H, 1.13; N, 0.00.

Synthesis of sodium tetramethoxyborate, Na[B(OMe)$_4$] (**1e**):

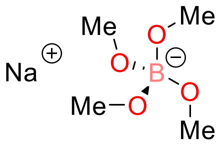

A Schlenk tube was charged with sodium borohydride (511 mg, 13.5 mmol, 1.0 equiv) and methanol (35 mL, 0.86 mol, 64 equiv) was added. The sodium borohydride quickly dissolved and the evolution of dihydrogen was observed. The reaction was stirred at room temperature for 30 minutes, after which a condenser was fitted and then heated to reflux for one hour. Subsequently, the solution was cooled to ambient temperature and the excess methanol was removed *in vacuo*. Sodium tetraphenoxyborate was isolated as a white powder. Yield: 1.8 g, 11 mmol, 84%. Spectroscopic values in agreement with literature.[5]

^{1}H NMR (400 MHz, CD_3CN, 295 K) δ/ppm: 3.26 (s, 12H, CH_3). **^{13}C{^{1}H} NMR** (101 MHz, CD_3CN, 295 K) δ/ppm: 49.3 (s, CH_3). **^{11}B NMR** (128 MHz, CD_3CN, 295 K) δ/ppm: 3.0 (s). **IR** ν_{max} (cm^{-1}): 2933 (w), 2819 (w), 1446 (vw), 1192 (w), 1060 (m), 984 (m), 951 (m) and 724 (vw). **Anal**. Calcd for $C_4H_{12}BNaO_4$ (Na[B(OMe)$_4$]): C, 30.42; H, 7.66; N, 0.00. Found: C, 30.27; H, 7.65; N, 0.00.

Synthesis of sodium tetraphenoxyborate, Na[B(OPh)₄] (**1f**):

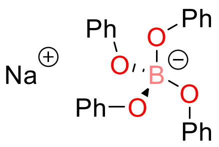

Sodium phenoxide (233 mg, 2.01 mmol, 1.0 equiv) dissolved in THF (15 mL) in a Schlenk tube was added dropwise to a stirred solution of triphenylborate (582 mg, 2.01 mmol, 1.0 equiv) dissolved in THF (10 mL). The reaction was left to stir at ambient temperature for 16 hours, after which the solvent was reduced *in vacuo* to approximately one third. Pentane (20 mL) was added, which precipitated the product out, and the resulting white powder was washed with pentane (3 × 10 mL). Further drying *in vacuo* afforded the product sodium tetraphenoxyborate as a white powder. Yield: 652 mg, 1.61 mmol, 80%. Spectroscopic values in agreement with literature.[6]

^{1}H NMR (400 MHz, CD_3CN, 295 K) δ/ppm: 7.06–7.01 (m, 16H, Ar–H), 6.65–6.62 (m, 4H, Ar–H). **^{13}C{^{1}H} NMR** (101 MHz, CD_3CN, 295 K) δ/ppm: 158.7 (s), 129.4 (s), 119.9 (s), 119.0 (s). **^{11}B NMR** (128 MHz, CD_3CN, 295 K) δ/ppm: 2.5 (s). **IR** ν_{max} (cm^{-1}): 1594 (w), 1491 (m), 1230 (m), 1162 (w), 1024 (w), 982 (sh), 956 (m), 930 (m), 759 (m), 717 (w) and 691 (m). **Anal**. Calcd for $C_{24}H_{20}BNaO_4$ (Na[B(OPh)$_4$]): C, 70.96; H, 4.96; N, 0.00. Found: C, 71.10; H, 5.08; N, 0.00.

Attempted synthesis of sodium tetraisopropoxyborate, Na[B(O^{i}Pr)$_4$] (**1g**).

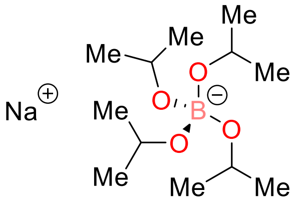

The synthesis of sodium tetraisopropoxyborate, Na[B(O^{i}Pr)$_4$] (**1g**), was attempted following the same procedure used for sodium tetraphenoxyborate, where the product Na[B(O^{i}Pr)$_4$] immediately precipitated out of solution after the addition of sodium isopropoxide with triisopropyl borate. However, ^{1}H NMR spectroscopy revealed unreacted starting material present, even after purification attempts using isopropanol (the crude mixture is soluble in isopropanol). Analysis of the ^{1}H NMR spectrum showed unreacted starting material present, as two sets of doublets at 1.03 and 0.97 ppm were observed. Due to the close proximity of these signals reliable integration could not be performed. Furthermore, crude **1g** proved to have very low solubility, as even a 0.25 M electrolyte solution in EC:DEC (1:1 v/v) could not be prepared.

Sodium isopropoxide (504 mg, 6.14 mmol, 1 equiv) dissolved in THF (10 mL) was added dropwise to triisopropyl borate (1.4 mL, 6.14 mmol, 1 equiv) in THF (10 mL). Immediately a white precipitate formed and the resulting suspension was heated to 50 °C for 16 hours. The suspension was cooled to ambient temperature and the solvent was removed *in vacuo*. Dry isopropanol (15 mL) was then added and heated to reflux, which dissolved the title compound. Upon cooling, sodium tetraisopropoxyborate began to precipitate out of solution, and further precipitation occurred after cooling to −30 °C. The solvent was removed by syringe and the resulting white powder was washed

with pentane (3 × 5 mL). Drying *in vacuo* afforded the crude product sodium tetraisopropoxyborate as a white powder.

S2 Stability of sodium borate to air and water.

S2.1 Stability to air.

To determine the air stability of the synthesised sodium borate salts **1a**–**f**, a vial containing 0.1 mmol of the salt was left exposed to air for 24 hours. Another vial also containing 0.1 mmol of the salt was left exposed for 48 hours. ^{1}H, ^{11}B and where applicable ^{19}F NMR spectra were recorded in CD_3CN solvent to detect any decomposition. Due to low solubility of the decomposition products as well as some of the salts (particularly non-fluorinated salts), the experiment was repeated using d^6-DMSO as the NMR solvent to confirm findings. Key NMR spectra are shown in Section 10.

The NMR spectra of $Na[B(hfip)_4]\cdot DME$ (**1a**) showed decomposition had occurred after both 24 hours and 48 hours, with new signals appearing in both the ^{1}H and ^{19}F NMR spectra. Note the vial contains significant amount of insoluble white powder. No changes were detected in the ^{11}B NMR spectrum, with the white powder being too insoluble in both CD_3CN and d^6-DMSO.

The NMR spectra of $Na[B(pp)_2]\cdot 3DME$ (**1b**) showed no change in appearance after 24 hours and 48 hours. Note the vial did not contain insoluble material as seen in other cases. The sodium borate salt **1b** appears to be the most resistant to decomposition in air. The same experiment on $Na[B(pp)_2]$ (**1b'**) gave an unchanged ^{11}B spectrum and ^{19}F NMR spectrum.

The NMR spectra of $Na[B(OCH_2(CF_2)_2CF_3)_4]$ (**1c**) showed little decomposition after 24 hours and 48 hours, with the ^{19}F NMR spectrum remaining largely unchanged. The ^{1}H NMR spectrum shows a very low intensity triplet, suggesting liberation of the alcohol. Note the vial did contain insoluble white powder, but the solubility of the borate salt **1c** is lower in CD_3CN than **1a** and **1b**. No changes were detected in the ^{11}B NMR spectrum. Repeating the experiment but using d^6-DMSO as the solvent gave the same findings and did not leave behind any material.

The NMR spectra of $Na[B(OPh^F)_4]\cdot 3DME$ (**1d**) showed decomposition after 24 hours and 48 hours, as observed by the appearance of a new broad signal in the ^{1}H NMR spectrum. This is likely to be the liberated alcohol OH signal. Note the vial did contain insoluble white powder. No changes were detected in the ^{11}B NMR spectrum when using CD_3CN solvent due to low solubility. Repeating using d^6-DMSO as the solvent showed a broad signal at 19.7 ppm as the dominant signal in the ^{11}B NMR spectrum after 48 hours, indicating significant decomposition has occurred.

The very low solubility of $Na[B(OMe)_4]$ (**1e**) made determining its air stability difficult. The ^{1}H NMR spectrum after 24 hours suggested some decomposition had occurred, but after 48 hours only the solvent signal was observed. The decreased solubility from 24 to 48 hours (meaning a ^{1}H NMR

spectrum could not be obtained) indicates decomposition but this could not be verified by NMR spectroscopy. The solubility was not improved when using d^6-DMSO as the NMR solvent which gave the same findings.

The NMR spectra of Na[B(OPh)$_4$] (**1f**) looked to show decomposition after 24 hours, as observed by a new broad resonance at *ca*. 5.5 ppm in the ^{1}H NMR spectrum, likely to be from the OH group on phenol. In addition, the aromatic region shows multiple new signals, giving further evidence of liberation of phenol.

S2.2 Stability to water.

To determine the water stability of the synthesised sodium borate salts **1a**–**f**, a 0.1 mmol scale NMR experiment in CD_3CN (0.4 ml) solvent was undertaken. Initially 1 equiv of water was added and the ^{1}H, ^{11}B and ^{19}F where applicable NMR spectra were recorded after 1 hour. After this multi-nuclear NMR spectra were recorded after 1 day. At this point the level of water was increased to 5 equiv, with the NMR spectra recorded the following day. Following this, 5 more equivalents of water were added to the NMR tube, giving a total of 10 equiv, with the NMR spectra being recorded the following day. Key NMR spectra are shown in Section 10.

For Na[B(hfip)$_4$]·DME (**1a**), throughout the NMR experiment the appearance of the ^{1}H, ^{11}B and ^{19}F NMR spectra did not greatly change. With 5 equiv of water present a white precipitate had formed in the NMR tube. Repeating the experiment in d^6-DMSO also did not show changes in the appearance of the NMR spectra but the solution had turned pale yellow after 1 day with 1 equiv of water present, suggesting sensitivity to water.

For Na[B(pp)$_2$]·3DME (**1b**), throughout the NMR experiment the appearance of the NMR spectra did not change. Unlike **1a**, **1b** did not show a white precipitate in the NMR tube. The sodium borate salt **1b** appears to be the most resistant to hydrolysis. The same experiment on Na[B(pp)$_2$] (**1b’**) gave an unchanged ^{11}B spectrum and ^{19}F NMR spectrum after 10 equiv of water were added.

For Na[B(OCH$_2$(CF$_2$)$_2$CF$_3$)$_4$] (**1c**), the appearance of the ^{1}H NMR spectrum started to change after 1 day with 1 equiv of water present. Liberation of the alcohol looked to take place, with a new triplet resonance forming. After 10 equiv of water were added the ^{11}B NMR spectrum only gave a weak signal, which with the white precipitate in the NMR tube present suggests almost complete hydrolysis had occurred.

For Na[B(OPh^F)$_4$]·3DME (**1d**), the appearance of the ^{1}H NMR spectrum started to change after 1 day with 1 equiv of water present. Liberation of the alcohol looked to take place, with a new resonance in the ^{1}H NMR spectrum appearing. With 5 equiv of water present the ^{11}B NMR spectrum showed

complete loss of the starting sodium borate **1d** and instead a new signal at *ca*. 20 ppm was observed. This new signal in the ^{11}B NMR spectrum increased in intensity with 10 equiv of water present. The sodium borate salt **1d** is very susceptible to hydrolysis.

For $Na[B(OMe)_4]$ (**1e**), the ^{1}H NMR spectrum does not show a change in chemical shift or formation of a new signal throughout the experiment. The ^{11}B NMR spectrum when using d^6-DMSO as the NMR solvent showed a reduction in the intensity of the signal, but no new signal appeared. This perhaps suggests some hydrolysis has occurred but the product is not detected using NMR spectroscopy.

For $Na[B(OPh)_4]$ (**1f**), the ^{11}B NMR spectrum after 1 hour with 1 equiv of water present starts to show the appearance of a new low intensity signal at *ca*. 11 ppm. This signal grows in intensity and when 5 equiv of water are added complete loss of **1f** has taken place, with only the signal at *ca*. 11 ppm present. The ^{1}H NMR spectrum shows multiple signals in the aromatic region with 1 equiv of water added after 1 day, with a new singlet resonance also observed. This is likely to be from the OH group of the liberated alcohol. The sodium borate salt **1f** is very susceptible to hydrolysis.

S3 Single crystal X-ray diffraction.

S3.1 Single crystal X-ray diffraction experimental.

Crystals suitable for single crystal X-ray diffraction were grown by hot recrystallisation from DME solvent and slowly cooled to ambient temperature under a dinitrogen atmosphere.

X-ray crystallographic data were collected using a Bruker D8-QUEST diffractometer equipped with an Incoatec IµS Cu microsource (Cu Kα, λ= 1.5418 Å) and a PHOTON-III detector. The temperature was held at either 180(2) or 220(2) K using an Oxford Cryosystems N_2 cryostat. Data collection, integration and reduction were undertaken using the APEX4 software suite. Multi-scan corrections were applied using SADABS (**1d**) or TWINABS (**1b**). Structures were solved using SHELXT[7] and refined using SHELXL.[8]

$Na[B(pp)_2]\cdot 3DME$ (**1b**): The diffraction pattern was indexed and integrated as two components, related by 180 degree rotation around the reciprocal lattice vector (-1 0 1). The data set used for refinement comprised all single reflections for the strongest component, plus all overlaid reflections, using the HKLF-5 format. The diffracted intensity dropped off quite rapidly as a function of resolution, with I/σ falling below 2.0 around 1.0 Å. The data for refinement are truncated to 0.95 Å resolution, with overall < 50% observed at I/σ > 2.0. The precision of the structure is limited accordingly. Several crystals were examined at 180(2) and 220(2) K, and the reported data set is the best obtained. The DME ligands in the $[Na(DME)_3]^+$ cation are modelled as disordered over two components, with restraints applied to the geometry and displacement parameters. The approximate spherical disorder of the resulting $[Na(DME)_3]^+$ cation may contribute to the rapid drop off in the diffracted intensity at higher diffraction angles.

$Na[B(OPh^F)_4]\cdot 3DME$ (**1d**): The diffraction pattern was indexed and integrated as a single component, but subsequent analysis using the TWINROTMAT module in PLATON[9] identified a plausible (non-merohedral) twin law, which was used to construct a data set in the HKLF-5 format. Refinement then proceeded without difficulty to yield a well-resolved structure.

CCDC 2132618 and 2132619 contain the supplementary crystallographic data for this paper. These data can be obtained free of charge via www.ccdc.cam.ac.uk/data_request/CIF or by emailing data_request@ccdc.cam.ac.uk, or by contacting The Cambridge Crystallographic Data Centre, 12 Union Road, Cambridge, CB2 1EZ, UK; fax: +44 1223 336033.

S3.2 Solid-state structures.

Figure S3.2.1 Solid-state structure of Na[B(pp)$_2$]·3DME (**1b**). Pink: Boron, Red: Oxygen, Green: Fluorine, Grey: Sodium. Displacement ellipsoids drawn at 50% probability and H-atoms omitted. Disorder of the DME ligands in the [Na(DME)$_3$]$^+$ cation is also omitted for clarity.

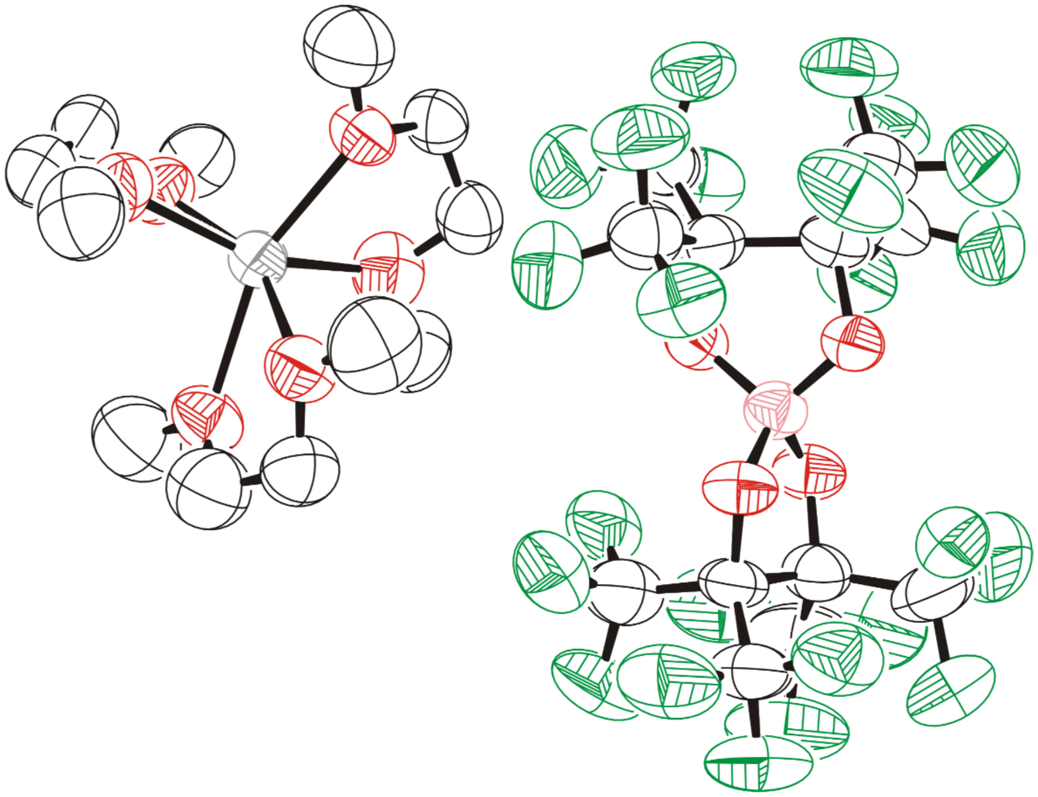

Figure S3.2.2 Solid-state structure of Na[B(OPh^F)$_4$]·3DME (**1d**). Pink: Boron, Red: Oxygen, Green: Fluorine, Grey: Sodium. Displacement ellipsoids drawn at 50% probability and H-atoms omitted.

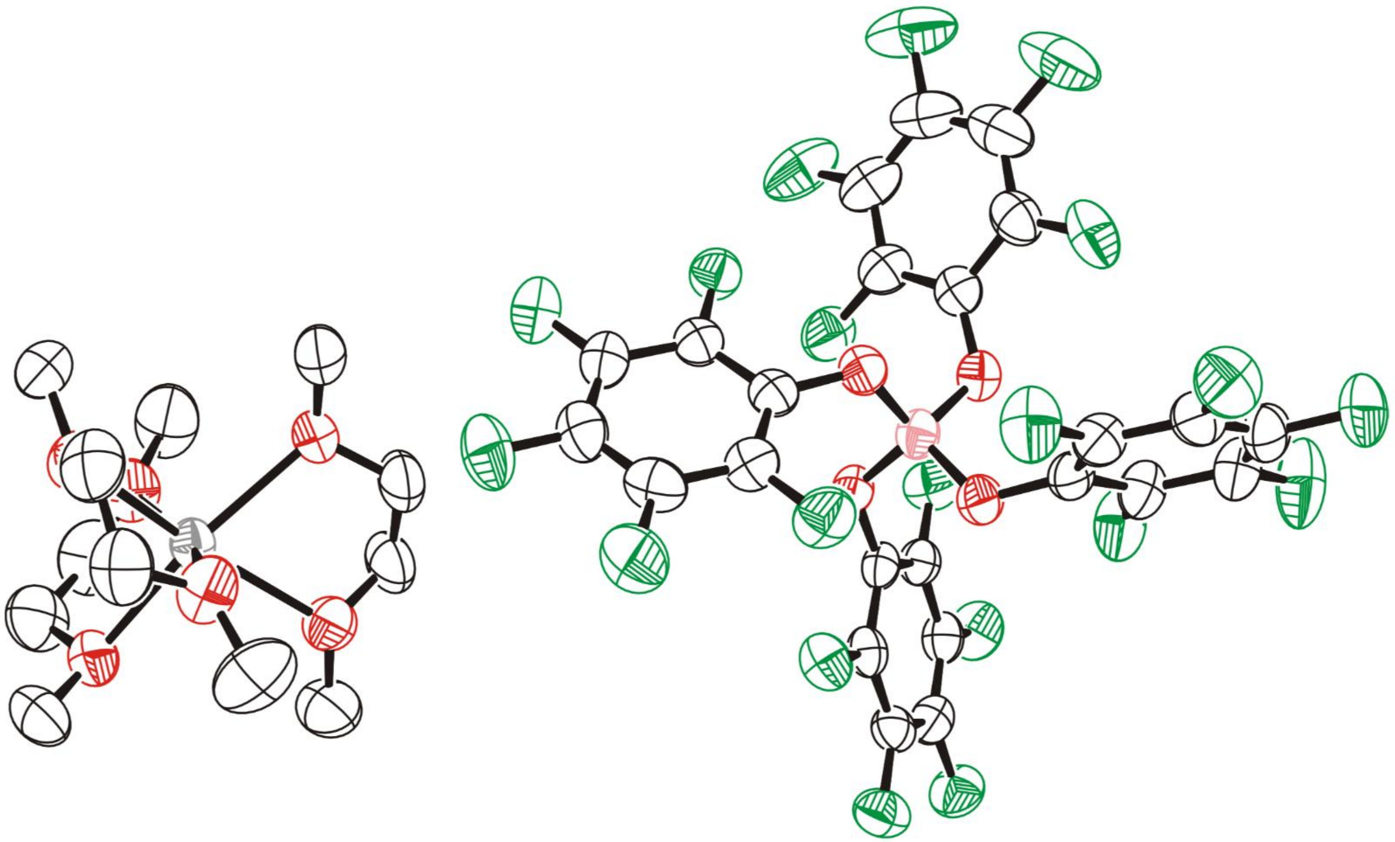

S3.3 Refinement data.

Table S3.3.1 Refinement data for Na[B(pp)$_2$]·3DME (**1b**) and Na[B(OPh^F)$_4$]·3DME (**1d**).

	**1b**	**1d**
CCDC number	2132618	2132619
Cambridge data number	DW_B2_0306	DW_B2_0301
Moiety formula	$[C_{12}H_{30}NaO_6]^+$,$[C_{12}BF_{24}O_4]^-$	$[C_{12}H_{30}NaO_6]^+$,$[C_{24}BF_{20}O_4]^-$
Empirical formula	$C_{24}H_{30}BF_{24}NaO_{10}$	$C_{36}H_{30}BF_{20}NaO_{10}$
Formula weight	968.28	1036.40
Temperature / K	220(2)	180(2)
Crystal system	monoclinic	triclinic
Space group	$P2_1/n$	P -1
a / Å	14.2078(10)	13.6662(6)
b / Å	17.7391(11)	13.9076(6)
c / Å	15.9073(10)	14.2892(7)
α / degrees	90	102.019(2)
β / degrees	101.777(4)	113.427(2)
γ / degrees	90	108.579(2)
Unit-cell volume / Å^3	3924.8(4)	2179.97(18)
Z	4	2
Calc. density / g cm^{-3}	1.639	1.579
F(000)	1944	1044
Radiation type	CuKα	CuKα
Absorption coefficient / mm^{-1}	1.870	1.600
Crystal size / mm^3	0.18 x 0.16 x 0.04	0.14 x 0.14 x 0.12
2-θ range / degrees	7.55-109.33	7.27-133.24
Completeness to max 2-θ	0.474	0.990
No. of reflections measured	43314	32239
No. of independent reflections	4665	7617
R(int)	0.1169	0.0756
No. parameters / restraints	678 / 270	620 / 0
Final R1 values (I > 2σ(I))	0.0973	0.0552
Final wR(F^2) values (all data)	0.2907	0.1752
Goodness-of-fit on F^2	1.020	1.041
Largest difference peak & hole / e angstrom^{-3}	0.370, -0.338	0.268, -0.361

S4 Thermal stability plots and analysis.

S4.1 Thermal stability general experimental.

Thermogravimetric analysis (TGA): TGA data was recorded with a Mettler Toledo TGA / DSC 2 Star^{ed} system equipped with a Huber minichiller. A few milligrams of sample were taken out of the argon-filled glovebox and immediately transferred to the TGA heating chamber to minimise air exposure. All the measurements were performed form 25 °C to 800 °C with a heating rate of 10 °C min^{-1} and under nitrogen flow. The decomposition temperature is obtained from the onset temperatures of the TGA curves. Inflection points of the TGA curves are also given.

Differential scanning calorimetry (DSC): Measurements were carried out using a Mettler Toledo DSC822e equipped with a Haake EK90/MT chiller and a TS0801R0 sample robot. The samples were loaded into 40 µL aluminium crucibles inside an argon-filled glovebox. For the melting point (T_m) measurements, 15–28 mg of sample was loaded. The samples were heated from 25 °C to a temperature (200 °C for **1a** and 240 °C for **1b** and **1b'**) at a rate of 10 °C min^{-1}. To determine the freezing point of the electrolyte solutions, a sample of 20 µL of electrolyte solution was used and the samples were cooled from 25 °C to −40 °C. The electrolyte solutions were then heated back up to +40 °C with a rate of 5 °C min^{-1} under nitrogen to determine the melting point temperature. After that, they were cooled down again to −40 °C to determine the freezing point. Three repeats of each measurement were carried out to test for reproducibility.

S4.2 Thermogravimetric analysis plots.

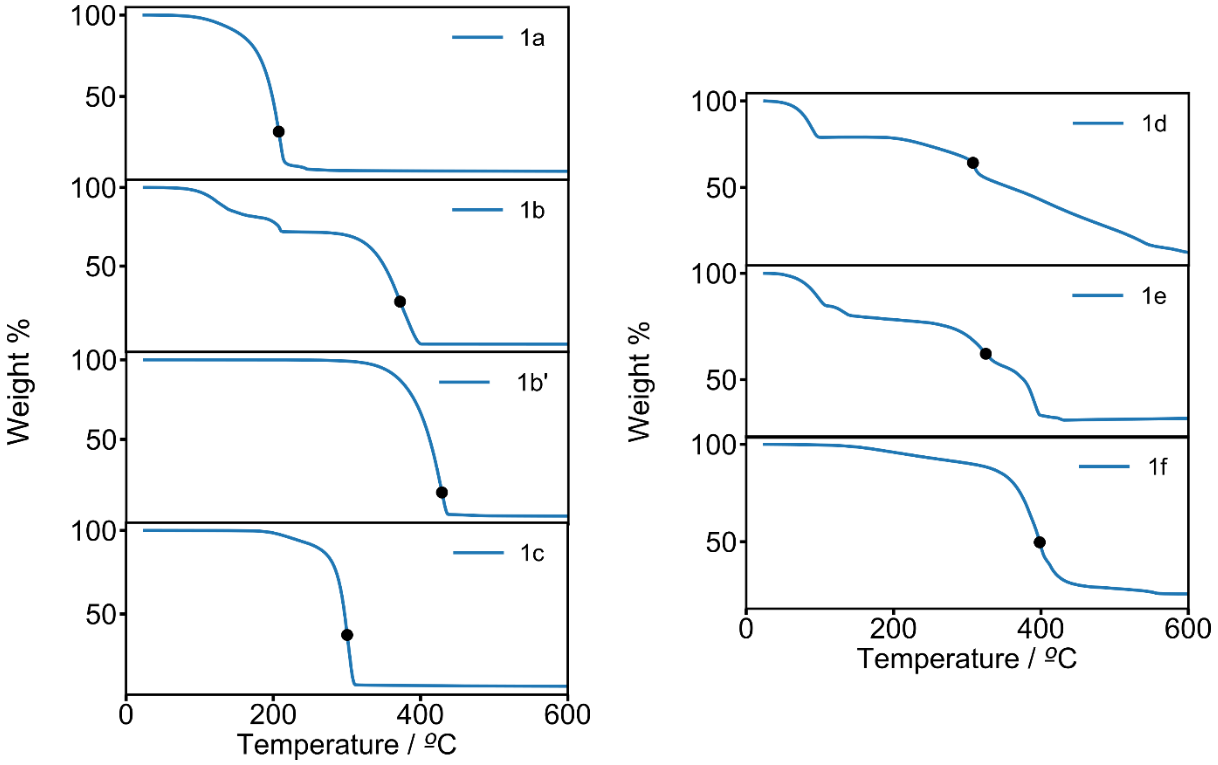

Figure S4.2.1 TGA curves for the sodium borate salts measured at a rate of 10 °C min^{-1}. The black dots correspond to the inflection points. Where: **1a** = $Na[B(hfip)_4]·DME$, **1b** = $Na[B(pp)_2]·3DME$, **1b’** = $Na[B(pp)_2]$, **1c** = $Na[B(OCH_2(CF_2)_2CF_3)_4]$, **1d** = $Na[B(OPh^F)_4]·3DME$, **1e** = $Na[B(OMe)_4]$, **1f** = $Na[B(OPh)_4]$.

Table S4.2.1 Inflection points (T_i) as determined by TGA.

**Compound**	**T_i / °C**
$Na[B(hfip)_4]·DME$ (**1a**)	208
$Na[B(pp)_2]·3DME$ (**1b**)	372
$Na[B(pp)_2]$ (**1b’**)	429
$Na[B(OCH_2(CF_2)_2CF_3)_4]$ (**1c**)	300
$Na[B(OPh^F)_4]·3DME$ (**1d**)	307
$Na[B(OMe)_4]$ (**1e**)	325
$Na[B(OPh)_4]$ (**1f**)	398

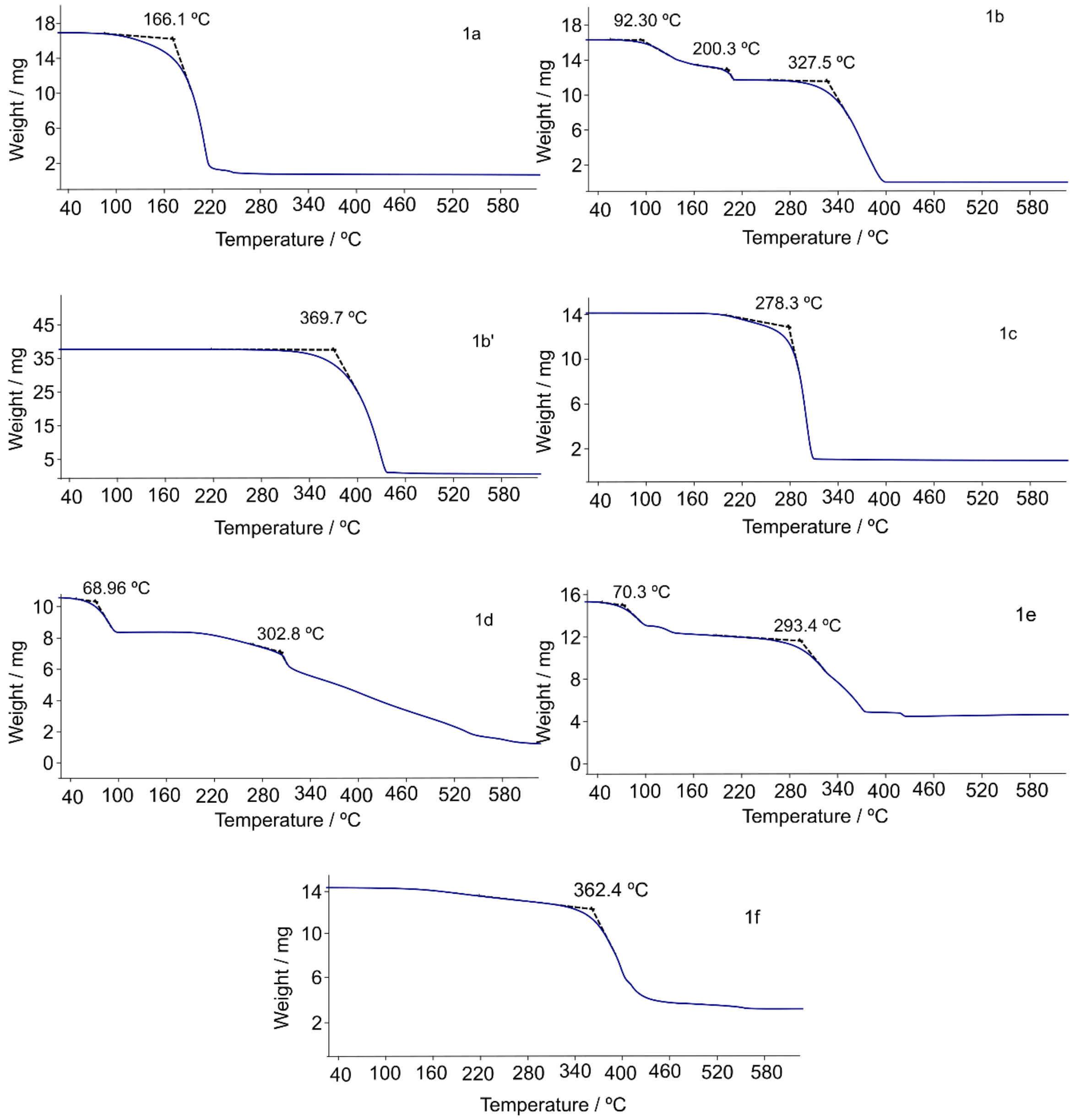

Figure S4.2.2 TGA curves for the sodium borate salts measured at a rate of 10 °C min^{-1}. The onset temperatures for the decomposition step(s) are shown for each of the salts. Where: **1a** = Na[B(hfip)$_4$]·DME, **1b** = Na[B(pp)$_2$]·3DME, **1b’** = Na[B(pp)$_2$], **1c** = Na[B(OCH$_2$(CF$_2$)$_2$CF$_3$)$_4$], **1d** = Na[B(OPh^F)$_4$]·3DME, **1e** = Na[B(OMe)$_4$], **1f** = Na[B(OPh)$_4$].

S4.3 Differential scanning calorimetry.

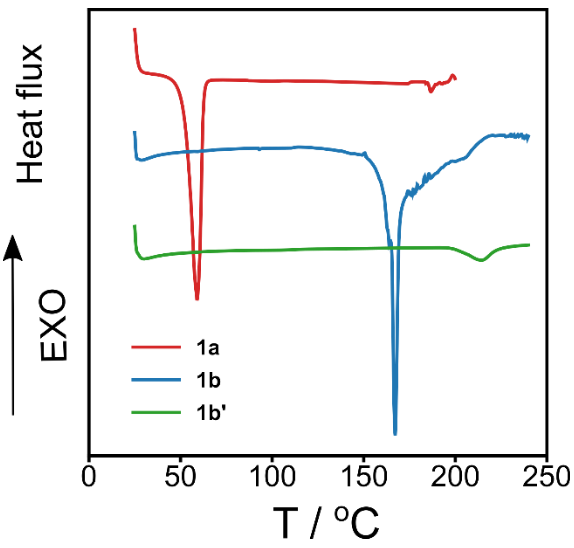

Figure S4.3.1 DSC curves for the sodium borate salts Na[B(hfip)$_4$]·DME (**1a**), Na[B(pp)$_2$]·3DME (**1b**) and Na[B(pp)$_2$] (**1b'**) measured at a rate of 10 °C min^{-1}. There is an endothermic event taking place at 58 °C for **1a**, 166 °C for **1b** and at 214 °C for **1b'**. This is assigned as the melting point for each of these compounds. The melting points follow the same trend as the decomposition temperatures measured by TGA, with **1b'** showing the highest melting point, followed by **1b** and **1a** having a much lower melting point compared to the other two.

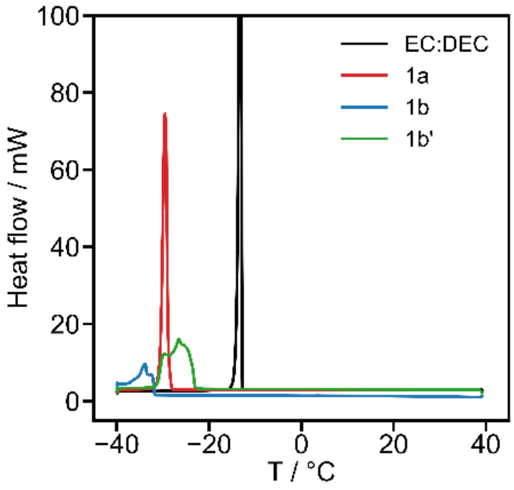

Figure S4.3.2 DSC cooling trace of electrolyte solutions Na[B(hfip)$_4$]·DME (**1a**), Na[B(pp)$_2$]·3DME (**1b**) and Na[B(pp)$_2$] (**1b'**) in EC:DEC (1:1 v/v). The electrolyte solution cooled to −40 °C from room temperature was heated to 40 °C and then cooled back down to −40 °C at a rate of 5 °C min^{-1}.

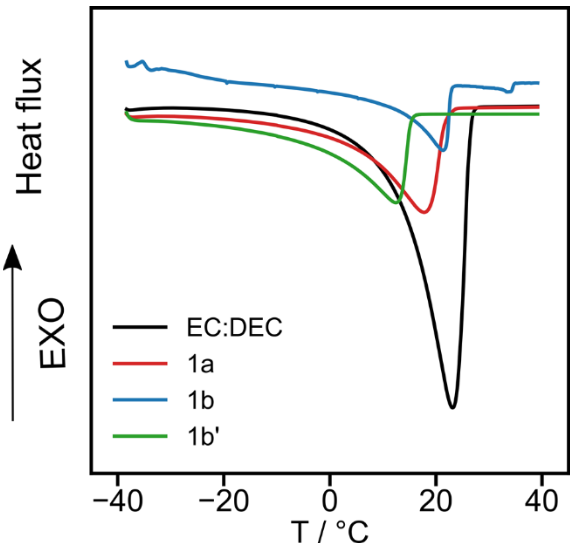

Figure S4.3.3 DSC heating traces of electrolyte solutions Na[B(hfip)$_4$]·DME (**1a**), Na[B(pp)$_2$]·3DME (**1b**) and Na[B(pp)$_2$] (**1b'**) in EC:DEC (1:1 v/v) measured at 5 °C min^{-1}. The EC:DEC (1:1 v/v) solvent was measured for comparison. The electrolyte solution cooled to −40 °C from room temperature was heated to 40 °C and then cooled back down to −40 °C at a rate of 5 °C min^{-1}. An endothermic peak is seen for all the samples and it is attributed to the melting of the frozen electrolyte solution. As expected, the salts lower the melting point. The melting temperatures are listed in Table S4.3.1.

Table S4.3.1 Freezing point temperature and melting point temperature (T_m) of electrolyte solutions as determined by DSC. Values calculated from three repeat measurements to give average temperature and standard deviation.

	**Freezing point / °C**		**T_m / °C**	
	**Average**	**Standard deviation**	**Average**	**Standard deviation**
EC:DEC (1:1 v/v)	-12.4	0.3	23.3	0.3
Na[B(hfip)$_4$]·DME (**1a**)	-29.5	0.3	18.5	0.4
Na[B(pp)$_2$]·3DME (**1b**)	-36.7	3	21.5	0.3
Na[B(pp)$_2$] (**1b'**)	-27.1	1	12.4	0.3

Note: A large hysteresis effect is observed in the freezing point and melting point temperatures during the DSC electrolyte experiments. To look at this, we took approximately 2 mL of EC:DEC (1:1 v/v) in a vial and placed it in a freezer at −30 °C for 18 hours. This froze the solvent. The vial was then placed in a fumehood at 24 °C (temperature measured using a temperature probe attached to a stirrer hotplate). After 2.5 hours the solvent had not completely melted and instead contained solid material suspended in solution. We hypothesise this is solid EC (lit. mp is 35–38 °C, value from Sigma-Aldrich online) suspended in DEC (lit. mp is −43 °C, value from Sigma-Aldrich online). These observations are consistent with the preparation of the EC:DEC (1:1 v/v) solvent mixture, where gentle heating is required to dissolve EC in DEC to make the binary carbonate solvent.

S5 Electrochemistry of sodium borate salts.

S5.1 Conductivity measurement experimental.

Solution conductivity measurements were made in a TSC 1600 Closed cell from RHD instruments. Electrolyte solutions were prepared by dissolving the respective sodium borate salts in an organic solvent blend of ethylene carbonate and diethyl carbonate (EC:DEC 1:1 v/v) at 1 M concentration, with the exception of $Na[B(OMe)_4]$ which was used at 0.5 M due to low solubility. A sample volume of 1 mL was used for all electrolytes, and the cell was filled and measured inside an argon-filled glovebox. Impedance spectra were measured using a PalmSens4 potentiostat, with an applied voltage amplitude of 10 mV and frequencies between 1 MHz and 1 Hz. The temperature of the measurements was between 30 °C and 35 °C. The impedance spectra were fitted using the equivalence circuit Q+Q/R, and the solution conductivity was found by taking the reciprocal of the R component, multiplied by the cell constant.

The cell constant was determined using aqueous potassium chloride solutions at concentrations of 0.001 M, 0.01 M, 0.02 M and 0.05 M. The solutions were prepared by diluting a 3 M stock solution with Millipore water. 1 mL of each solution was filled into the cell, and impedance was measured at 1 kHz. The solution conductivity was taken as the reciprocal of the real component of the impedance. This was plotted against the specific conductivity for the different concentrations, as shown in Figure S5.1.1. This procedure was repeated on different days when room temperature was approximately 25 °C and 20 °C, hence different specific conductivities for the two measurements.[10] The cell constant was then calculated as the average of the gradients of the two linear trendlines, as 15.5±0.2 cm^{-1}.

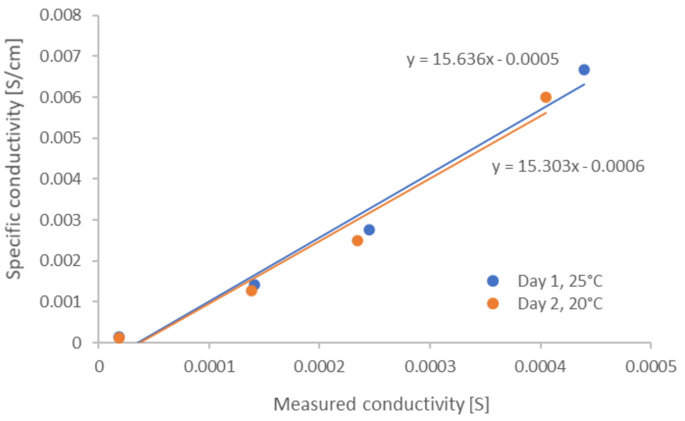

Figure S.5.1.1 Graph to determine cell constant for the TSC 1600 Closed cell from RHD instruments, using aqueous potassium chloride solutions between 0.001 M and 0.05 M. 1kHz, 10 mV amplitude.

S5.2 Impedance spectroscopy measurements of the SEI.

The conductivity and ageing of the native solid electrolyte interphase (SEI) were measured in Na-Na symmetric coin cells using Potentiostatic electrochemical impedance spectroscopy (PEIS) measurements between 1 MHz and 0.1 Hz (Figures 5, 6, and S5.5.1–S5.5.4). Consecutive impedance spectra were recorded for 90 hours after cell assembly, and Nyquist plots accumulated during the first 90 hours from cell assembly. It is assumed that the main contribution to the low-frequency impedance is the sodium SEI; thus, the evolution of the low-frequency impedance is attributed to the ageing, reorganisation, and dissolution of the SEI during rest.

Due to the dynamic nature of the sodium SEI, equivalent circuit fitting is not suitable for the analysis of the Nyquist plots. Thus, the EIS plots were analyzed using the fit circle function (EC-lab software). The frequency at the maximum and capacitance are reported. Capacitance in the range of 10^{-6}–10^{-8} F is typical for the SEI, while 10^{-9}–10^{-10} F is typical for grain boundaries.[11] Since no other source of grain boundary conduction is found in the symmetric sodium cells, the latter component is assigned to the grain boundaries in the SEI.

S5.3 Cyclic voltammetry measurements of the studied electrolytes.

The studied electrolytes' electrochemical stability window (ESW) was determined using cyclic voltammetry (CV). Mixtures of 1 M electrolyte in EC:DEC (1:1 v/v) were tested in three-electrode cells (RHD, surface cell) using aluminium as working electrodes (WE); sodium metal was used as the counter and quasi-reference electrode (Figures 7, 8, S5.5.5 and S5.5.6). The RHD surface cell is a "beaker style" cell that uses an excess of electrolyte (600 µl) and doesn't use a separator. Each CV experiment comprised of fifteen consecutive CV scans, five at each of the following rates: 5, 1 and 10 mV/sec.

S5.4 Coin cells assembly.

For all coin cells prepared, 75 µL of electrolyte was used. Celgard 3500 poly ethylene-poly propylene was used as the separator. Symmetric sodium metal electrode cells (2-electrode) were assembled in coin cells with 2 cm^2 electrodes. The oil on sodium was removed by washing with heptane and rolled to expose the non-oxidated surface. For Na-ion battery cells, 2-electrode single-layer coin cells with 1.13 cm^2 cathode area were assembled with a geometrically over-sized hard carbon anode (2 cm^2). Celgard 3500 poly ethylene-poly propylene was used as the separator.

For electrochemical battery tests, the Na-ion coin cells were tested at room temperature with a battery cycler (MPG 200, Biologic Instruments). The formation protocol consists of 5 charge-discharge cycles at a rate of C/5 between a voltage limit of 4.2–1.0 V. For cycle life tests, a C/5 charge/discharge (constant current- constant voltage, CCCV profile) rate was used. The C-rates was based on a nominal specific capacity of 130 $mAhg^{-1}$ for the cathode. The capacity of the coin cells was around 1 mAh.

Impedance measurements were done using Biologic Instruments VSP-300 in the frequency range 1 MHz–1 Hz at 10 mV amplitude.

S5.5 Electrochemistry analysis plots.

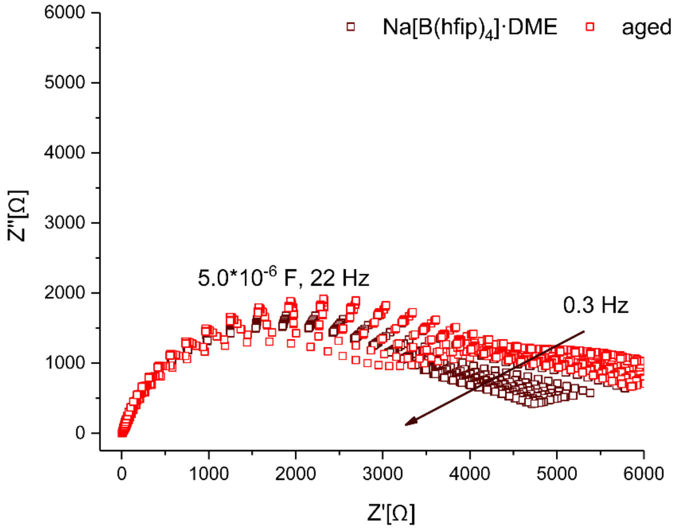

Figure S5.5.1 Consecutive EIS Nyquist plots of $Na[B(hfip)_4]$·DME using impedance spectroscopy 1 MHz–1 Hz 10 mV amplitude. High-frequency semicircle (left side): maximum at 22 Hz and capacitance of 5.0 × 10^{-6} F.

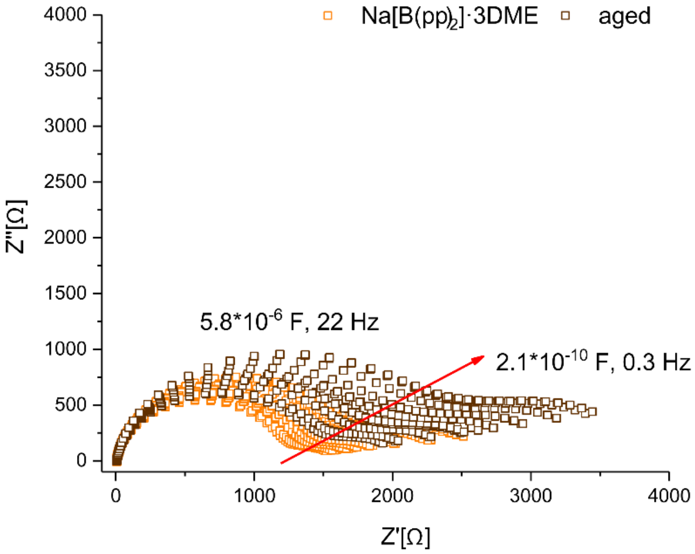

Figure S5.5.2 Consecutive EIS Nyquist plots of $Na[B(pp)_2]$·3DME using impedance spectroscopy 1 MHz–1 Hz 10 mV amplitude. High-frequency semicircle (left): maximum at 22 Hz and capacitance of 5.8 × 10^{-6} F, low frequency semicircle: maximum at 0.35 Hz and capacitance of 2.1 × 10^{-10}.

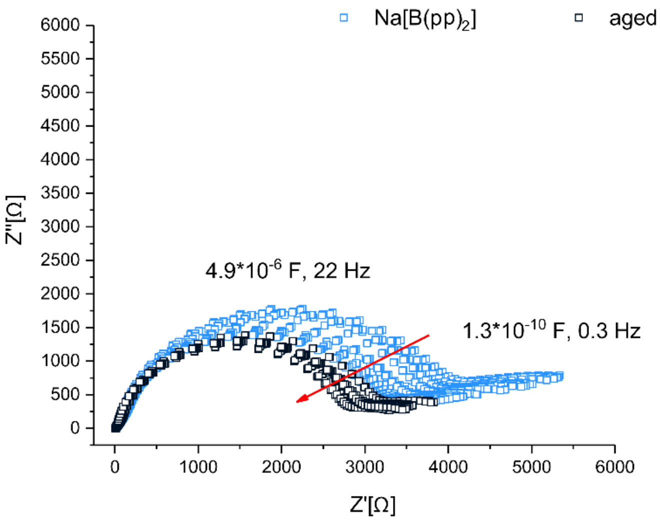

Figure S5.5.3 Consecutive EIS Nyquist plots of Na[B(pp)$_2$] using impedance spectroscopy 1 MHz–1 Hz 10 mV amplitude. High frequency semicircle (left): maximum at 22 Hz and capacitance of 4.9 × 10^{-6} F, low frequency semicircle: maximum at 0.35 Hz and capacitance of 1.3 × 10^{-10}

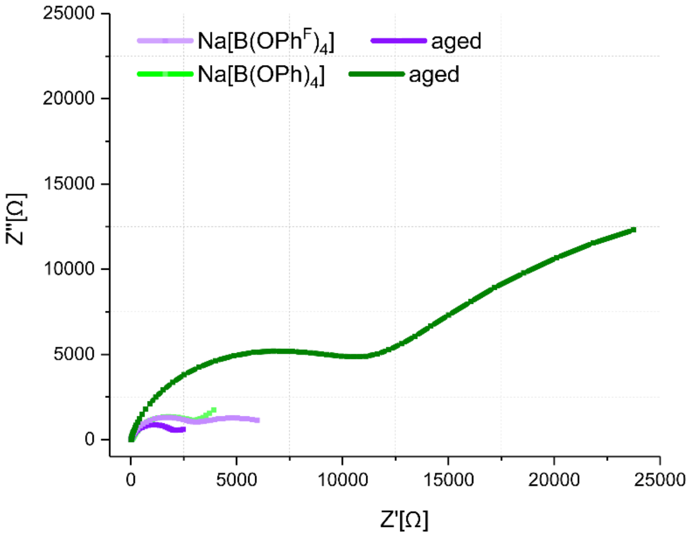

Figure S5.5.4 Impedance spectroscopy Nyquist plot of Na–Na symmetric cell of 1 M Na[B(OPh^F)$_4$] and Na[B(OPh)$_4$] in EC:DEC (1:1) 1 MHz–0.1 Hz 10 mV amplitude.

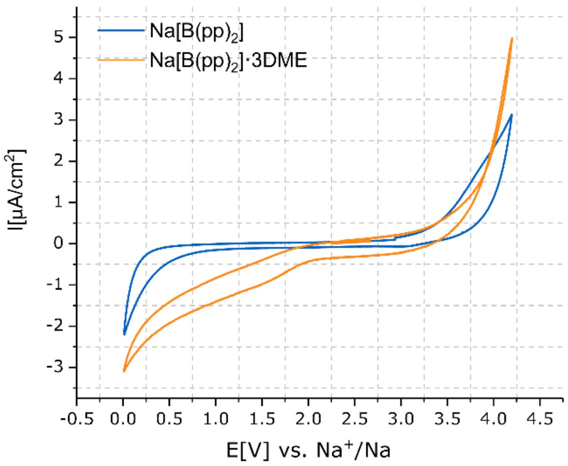

Figure S5.5.5 Cyclic voltammetry of Na[B(pp)$_2$] and Na[B(pp)$_2$]·3DME in EC:DEC (1:1 v/v) and Na[B(pp)$_2$] in DME solvent. Working electrode (WE) is aluminium, counter electrode (CE) and reference electrode (RE) are Na metal. 5mV/sec, first cycle.

In the CV experiment in this study, the WE undergoes both anodic and cathodic polarization; the first scans are the most informative for the evaluation of the electrochemical window. The following electrolyte scans were used mainly to validate that the formed SEI is stable and that there is no significant degradation of the passivation layer. All the CV scans for Na[B(pp)$_2$] (**1b'**) in EC:DEC (1:1 v/v) are presented in Figure S5.2.6. Notably, while the oxidation and reduction currents decrease with cycle number, the reduction peaks at approximately 3 V and 1.8 V were measured at 1 mV/sec (Figure S5.2.6), despite the passivation of the WE at the first five cycles. While we note that a slower scanning rate facilitates a better resolution of the CV peaks, the appearance of surface oxide reduction peaks (such as the peak at 3 V) suggests that the passivation of the electrode is not complete after five cycles. A similar trend was observed for Na[B(hfip)$_4$]·DME and Na[B(pp)$_2$]·3DME electrolytes. This trend is evidence for the increased solubility and reactivity of the Na SEI.

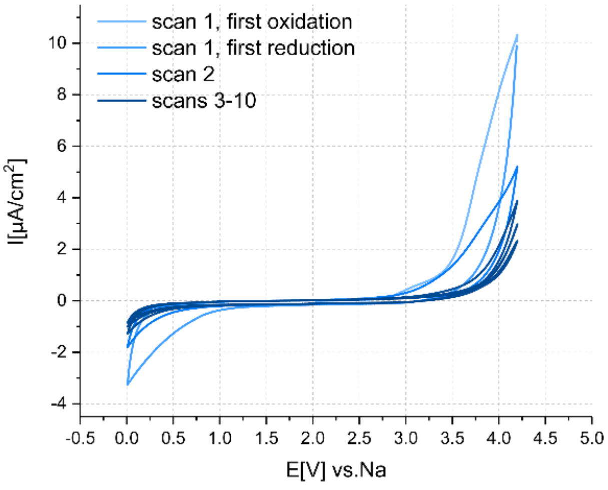

Figure S5.5.6 Cyclic voltammetry of $Na[B(pp)_2]$ in EC:DEC (1:1 v/v). WE is aluminium, CE and RE are Na metal. 5 mV/sec.

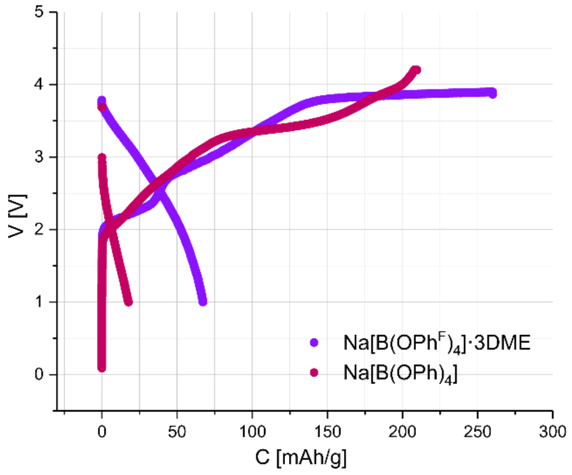

Figure S5.5.7 Cell voltage (V) vs. cumulative specific capacity. Collected from the 1st cycle at an approximate constant current rate of C/5 for charge and discharge using cell voltage limits of 1.0 and 4.2 V. Electrolyte is 1 M $Na[B(OPh^F)_4]\cdot 3DME$ and $Na[B(OPh)_4]$ salts in EC:DEC (1:1 v/v).

S5.6 Water content of electrolytes.

The sodium borate salts were synthesized and stored under anhydrous conditions. However, due to the hygroscopic nature of the sodium salts, the main contribution to water concentration in the studied electrolytes is the salt. It was found that different anions result in different typical water content in the electrolyte solution, regardless of reagent/solvent drying. Water content in the studied electrolytes was determined *via* Metrohm 899 Coulometer Karl Fischer titration. The typical water content in 1 M (0.5 M for **1e**) electrolytes are presented in Table S5.3.1.

Table S5.6.1 Typical water content in studied electrolyte solutions.

Compound	Water content/ ppm
$Na[B(hfip)_4]\cdot DME$ (**1a**)	50-150
$Na[B(pp)_2]\cdot 3DME$ (**1b**)	<50
$Na[B(pp)_2]$ (**1b'**)	
$Na[B(OCH_2(CF_2)_2CF_3)_4]$ (**1c**)	NA
$Na[B(OPh^F)_4]\cdot 3DME$ (**1d**)	>80
$Na[B(OMe)_4]$ (**1e**)	
$Na[B(OPh)_4]$ (**1f**)	

S5.7 Preparation of electrolytes.

Electrolytes were prepared by dissolving the required mass of sodium borate salt in EC:DEC (1:1 v/v) solvent to make a 1 M concentration solution (0.5 M concentration for $Na[B(OMe)_4]$ due to low solubility of the salt). Electrolytes were stored in an aluminium bottle in an argon filled glovebox.

During the characterisation of salts $Na[B(pp)_2]\cdot x$DME and $Na[B(OPh^F)_4]\cdot x$DME (x = number of DME molecules), single crystal X-ray diffraction found three DME solvent molecules solvate the sodium cation. However, inspection of the elemental analysis (EA) results found lower levels of DME present for both salts. For $Na[B(pp)_2]\cdot x$DME, two DME molecules were found by EA; one DME molecule was found by EA for $Na[B(OPh^F)_4]\cdot x$DME. This suggests the DME solvation is labile and can be partially removed while drying under vacuum. Nevertheless, for both salts the molecular weight was calculated assuming three DME molecules, and resulting electrochemical analysis assumed the presence of three DME molecules. Table S5.4.1 shows how the electrolyte concentration would change with different values of DME, assuming the same mass of salt was used. Note, for $Na[B(hfip)_4]\cdot$DME the one DME solvent molecule was determined by ^{1}H NMR spectroscopy by integrating the DME signals to the fluorinated isopropyl group proton.

Table S5.7.1 Electrolyte concentrations for the salts $Na[B(pp)_2]\cdot$xDME and $Na[B(OPh^F)_4]\cdot x$DME in EC:DEC (1:1 v/v), where x = 1, 2 and 3. The mass of salt used was constant in all cases (the amount needed for 1.0 M solution assuming 3 DME molecules), hence the difference in concentration for different amounts of DME present.

Salt	Electrolyte concentration with 3 molecules of DME (M)	Electrolyte concentration with 2 molecules of DME (M)	Electrolyte concentration with 1 molecule of DME (M)
$Na[B(pp)_2]\cdot x$DME	1.0	1.1	1.2
$Na[B(OPh^F)_4]\cdot x$DME	1.0	1.1	1.2

S5.8 Comparison of borate and $NaPF_6$ electrolytes.

The work in this manuscript details the use of sodium borate electrolyte salts as alternatives to $NaPF_6$ for use in sodium-ion batteries, with Na[B(hfip)$_4$]·DME (**1a**) and Na[B(pp)$_2$] (**1b'**) found to be the highest performing synthesised salts. Therefore, it is appropriate to compare their use to the current benchmark standard, $NaPF_6$. Note, the electrolytes are all at 1 M concentration in EC:DEC (1:1 v/v) solvent; the $NaPF_6$ was prepared as detailed in our previous work.[1]

The maximum bulk electrolyte conductivity of 1 M $NaPF_6$ (EC:DEC 1:1 v/v) is 19 mS/cm.[1] In contrast, the maximum bulk conductivity of Na[B(hfip)$_4$]·DME and Na[B(pp)$_2$] was found to be 10 mS/cm and 8 mS/cm respectively. It is important to note that in contrast to $NaPF_6$, the borates electrolyte concentration and solvent composition were not optimised in this study. Moreover, this work has shown the stability and conductivity of the interface (SEI) has a greater effect on battery performance compared to bulk conductivity.

The native SEI stability, electrochemical stability *vs*. Al and sodium-ion cell capacity were then compared in cells containing 1 M Na[B(pp)$_2$] and $NaPF_6$ electrolytes (Figures S5.8.1–S5.8.3). The estimated oxidation potential of 1 M $NaPF_6$ is significantly lower (*ca*. 3 V *vs*. Na^+/Na) compared to 3.7 V *vs*. Na^+/Na for Na[B(pp)$_2$]. The impedance of the native SEI on Na metal in $NaPF_6$ was approximately double the value for Na[B(pp)$_2$], demonstrating a similar stabilisation rate. Finally, the capacity and the sodiation curves for the third cycle seem to be comparable for both electrolytes.

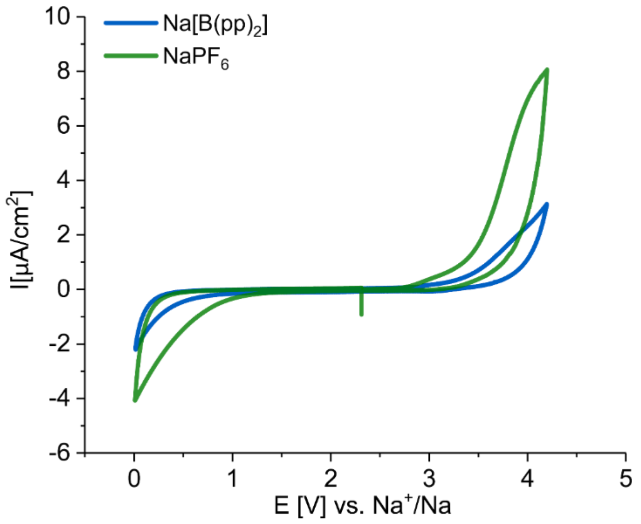

Figure S5.8.1 Cyclic voltammetry of 1 M Na[B(pp)$_2$] (**1b'**) (EC:DEC 1:1 v/v) and 1 M $NaPF_6$ (EC:DEC 1:1 v/v) electrolytes in three-electrode cell (WE- aluminum, CE- sodium metal, RE- sodium metal). 3rd cycle, measured at 5 mV/s between 0.01 V and 4.2 V.

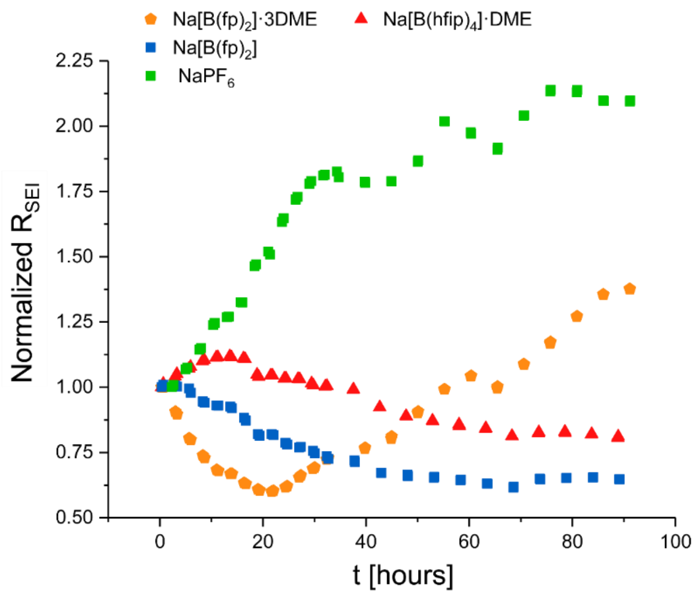

Figure S5.8.2 Evolution of the normalized R_{SEI} at 0.1 Hz *vs*. time. Na[B(hfip)$_4$]·DME (**1a**) (red), Na[B(pp)$_2$]·3DME (**1b**) (orange), Na[B(pp)$_2$] (**1b'**) (blue), and $NaPF_6$ (green). All electrolytes are 1 M concentration and in EC:DEC (1:1 v/v) solvent.

Comparing the evolution of the low-frequency (0.1 Hz) impedance (denoted R_{SEI}) of 1 M $NaPF_6$, 1 M Na[B(hfip)$_4$]·DME (**1a**) and 1 M Na[B(pp)$_2$] (**1b'**) in EC:DEC (1:1 v/v), it can be seen that the impedance of Na[B(hfip)$_4$]·DME and Na[B(pp)$_2$] stabilizes after 80 hours of storage, whereas for $NaPF_6$ the SEI impedance increases with respect to storage time. As SEI chemical dissolution is the most likely degradation mechanism in Na symmetric cells, the inner layer of the SEI in Na[B(pp)$_2$] and Na[B(hfip)$_4$] electrolytes is likely to be composed of less soluble electrolyte reduction products compared to $NaPF_6$.

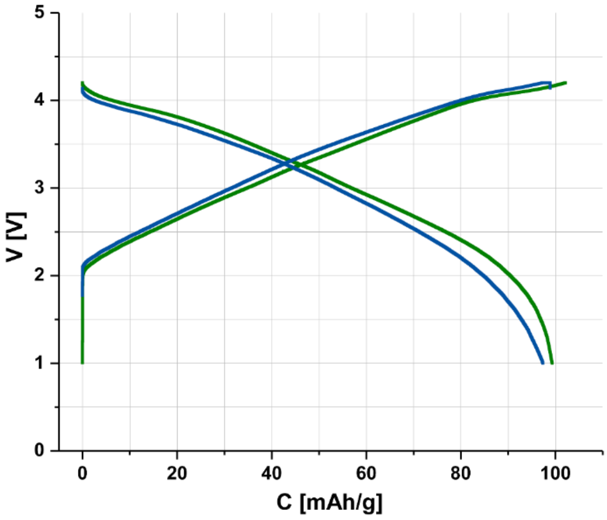

Figure S5.8.3 Charge and discharge curves of the third cycle of cells based on 1 M $NaPF_6$ (green) and 1 M $Na[B(pp)_2]$ (blue) salts (dissolved in EC:DEC 1:1 v/v).The significant similarity of both charge and discharge curves shows that the sodiation mechanism of the commercial hard carbon anode in the studied borate electrolytes is similar to that in $NaPF_6$.

S5.9 Sodium metal deposition experiments.

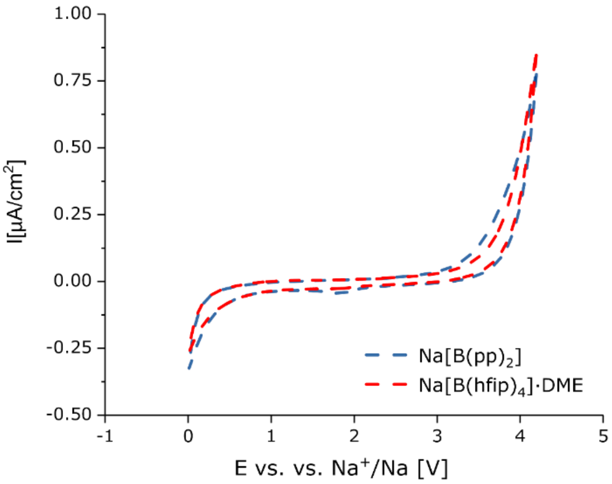

Figure S5.9.1 Cyclic voltammetry of 1 M $Na[B(hfip)_4]\cdot DME$ (**1a**) and 1 M $Na[B(pp)_2]$ (**1b'**) (EC:DEC 1:1 v/v) electrolytes in three-electrode cell (WE- aluminum, CE- sodium metal, RE- sodium metal). 3rd cycle, measured at 5 mV/s between 0.01 V and 4.2 V.

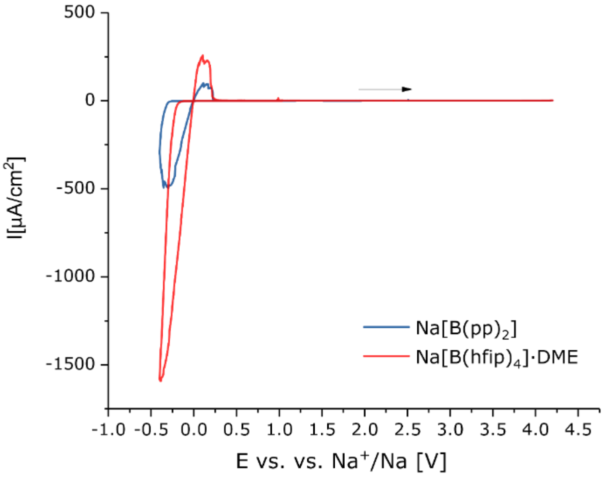

Figure S5.9.2 Cyclic voltammetry of 1 M $Na[B(hfip)_4]$·DME (**1a**) and 1 M $Na[B(pp)_2]$ (**1b'**) (EC:DEC 1:1 v/v) electrolytes in three-electrode cell (WE- aluminum, CE- sodium metal, RE- sodium metal). 3rd cycle, measured at 5 mV/s between −0.4 V and 4.2 V.

Figure S5.9.1 shows the electrochemical stability of 1 M $Na[B(hfip)_4]$·DME (**1a**) and 1 M $Na[B(pp)_2]$ (**1b'**) (EC:DEC 1:1 v/v) to validate the previous stability measurements (Figures 7 and Figure 8 top) before plating and stripping measurements were undertaken. To compare Na plating overpotentials in the borate electrolytes, we conducted CV scans between 4.2 V and −0.4 V *vs*. Na^+/Na (Figure S5.9.2). For both electrolytes, a metal reduction peak around −0.4 V and a metal oxidation oxidation peak around 0.2V were observed.

For both electrolytes, sodium metal plating and stripping peaks are not symmetrical. This is to be expected due to the non-optimised electrolyte conditions used for this experiment. Nevertheless, these preliminary results indicate that both electrolytes are promising candidates for Na metal and “anode-free” batteries.

S6 Solution-state NMR spectroscopy of electrolytes after cycling.

S6.1 Experimental Details.

NMR samples were prepared by opening cycled coin cells and removing the Celgard separators. The separators were put into vials and soaked with 0.7 mL of DMSO-d_6 for five minutes. The solutions were then pipetted into 5 mm out diameter NMR tubes fitted with J-Young taps. NMR experiments were conducted on a Bruker Avance III HD spectrometer equipped with an 11.7 T magnet (v_0^{1H} = 500 MHz) using a BBO probe. A Bloch decay pulse sequence was used with optimized pulse lengths. ^{1}H chemical shifts were referenced using the residual proton peak of the DMSO-d_6 solvent at 2.50 ppm.

S6.2 NMR spectra.

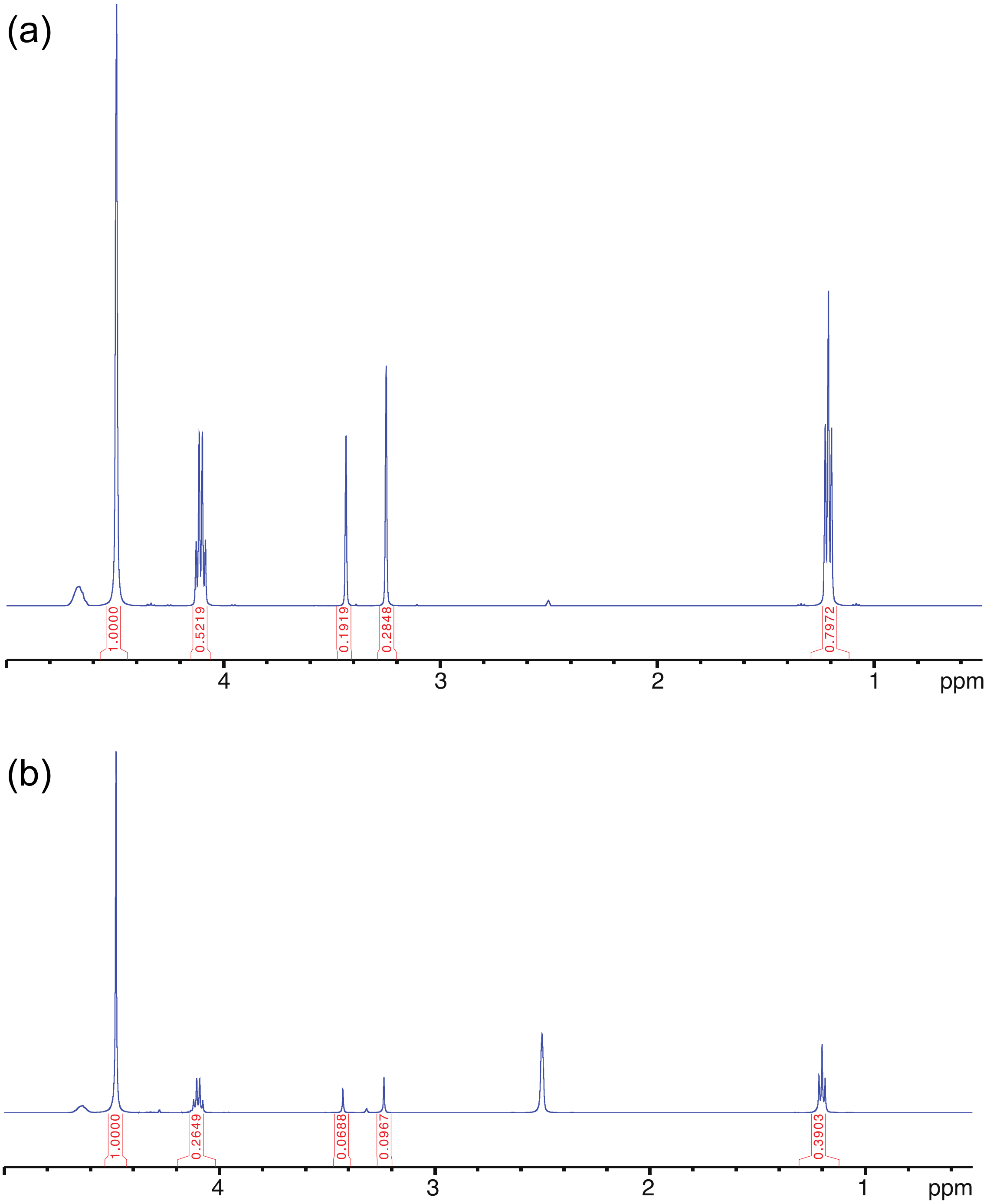

Figure S6.2.1 ^{1}H NMR spectra of (a) pristine and (b) cycled 1 M $Na[B(hfip)_4]$•DME in EC:DEC (1:1 v/v%). Integrals are shown relative to the EC signal.

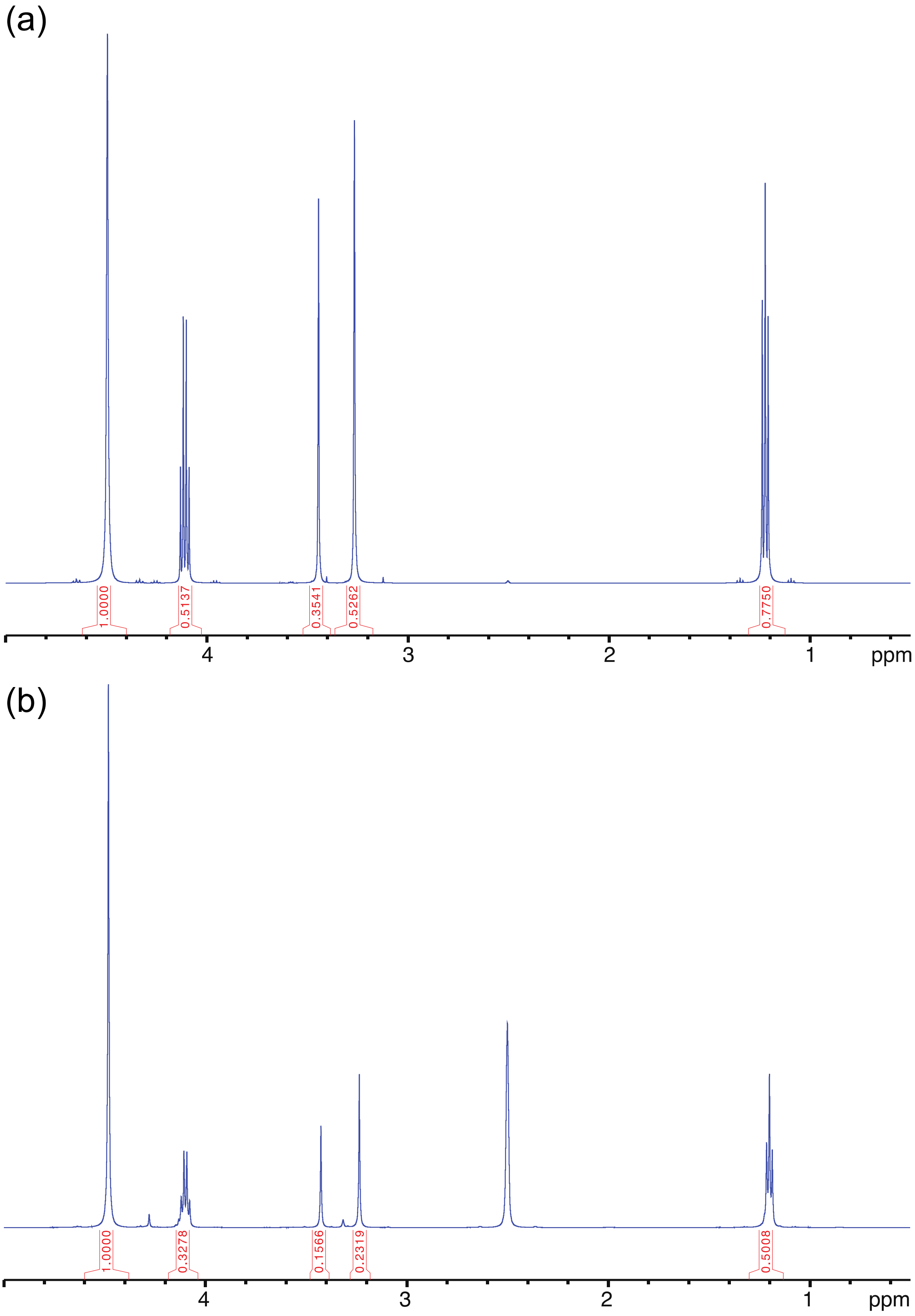

Figure S6.2.2 ^{1}H NMR spectra of (a) pristine and (b) cycled 1 M $Na[B(pp)_2]$•3DME in EC:DEC (1:1 v/v%). Integrals are shown relative to the EC signal.

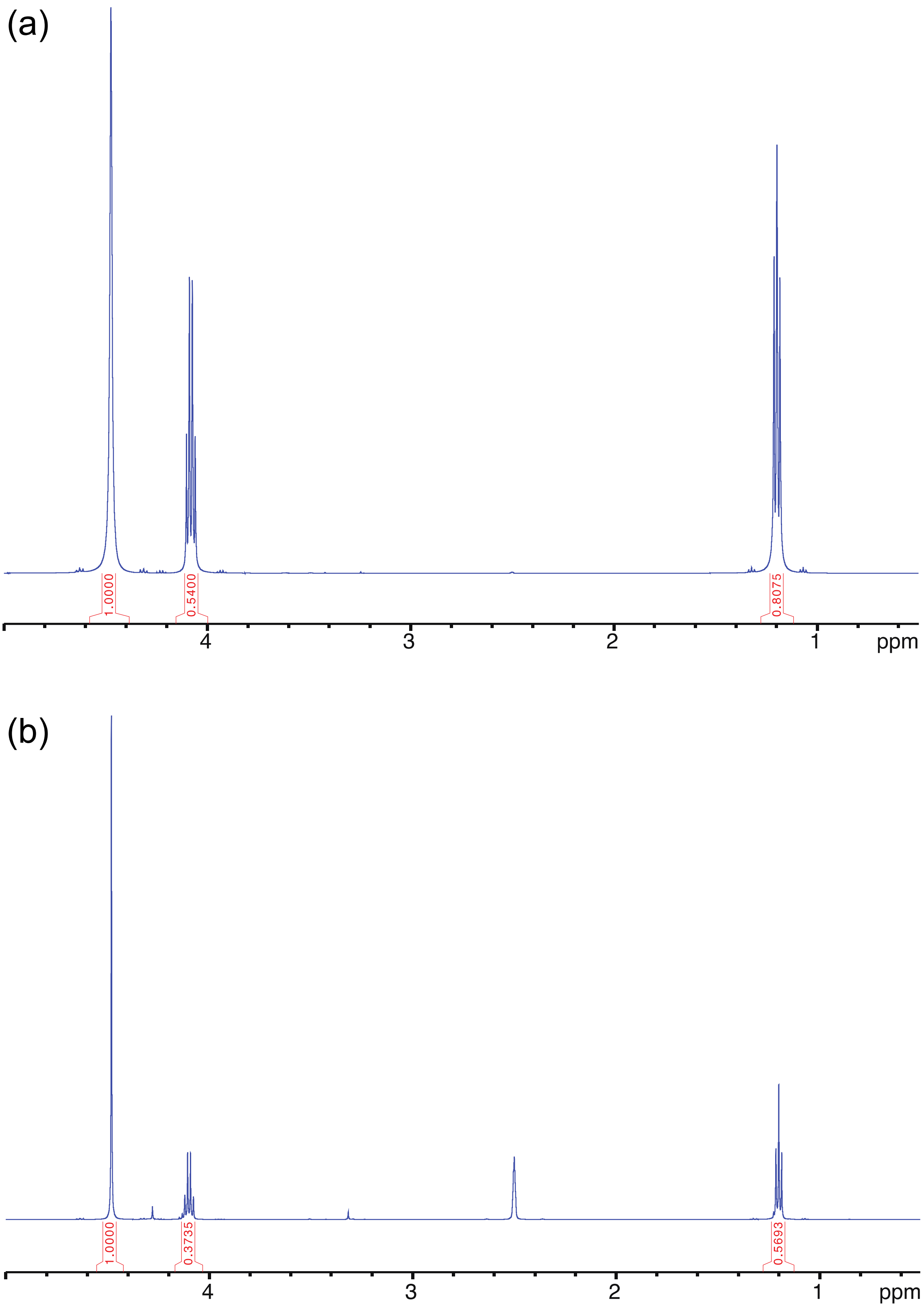

Figure S6.2.3 ^{1}H NMR spectra of (a) pristine and (b) cycled 1 M Na[B(pp)$_2$] in EC:DEC (1:1 v/v%). Integrals are shown relative to the EC signal.

S7 Commercial 2- and 3-elctrode pouch cell cycling.

S7.1 Experimental

Composite cathode

Cathode slurry was fabricated by dispersing Faradion's proprietary layered oxide cathode material and carbon conductive additives in a polymer solution of PVDF in N-methyl pyrrolidinone (NMP) using a Thinky ARE 310 mixer. The resulting slurry was coated onto carbon-coated aluminium foil using a 100 mm width adjustable Doctor-blade from RK Print, UK. The wet coating was dried initially at 80 °C for about 15 minutes in ambient air and thereafter transferred to a vacuum oven and dried at 120 °C for a further 12 hours. The cathode was calendered to a porosity of 35%.

Composite anode

Anode slurry was fabricated in a similar way as the cathode slurry by dispersing commercial hard carbon and carbon conductive additive in a polymer solution of PVDF in NMP. The anode slurry was also coated onto carbon-coated aluminium foil. The wet coating was dried at 80 °C for about 15 minutes in ambient air and thereafter transferred to a vacuum oven and dried at 120 °C for a further 12 hours. The anode was calendered to a porosity of 40%.

Pouch cells fabrication

2-electrode single-layer pouch cells with 4 cm^2 cathode area (20 × 20 mm^2) were manufactured with a geometrically over-sized hard carbon anode (22 × 22 mm^2). Whatman Glass microfiber filter (GF/A) (26 × 26 mm^2, glass microfiber filter CAT No. 1820–150) was used as the separator.

For 3-electrode single layer pouch cells, 2 glass-fibre separators were used. A piece of sodium metal pressed onto aluminium tab was used as a reference-electrode (RE).

Electrolyte solutions at 1 M concentration of 3 different sodium borate salts, $Na[B(hfip)_4]\cdot DME$ (**1a**), $Na[B(pp)_2]\cdot 3DME$ (**1b**) and $Na[B(pp)_2]$ (**1b'**) were prepared by dissolving the respective salts in an organic solvent blend of ethylene carbonate, diethyl carbonate and propylene carbonate (1:2:1 wt/wt).

Electrochemical tests

The pouch cells were tested at 30 °C in a temperature-controlled chamber (Memmert, Germany) with a battery cycler (Model 4600 Battery test system, Maccor, USA). The formation protocol consists of 5 charge-discharge cycles at a rate of C/10 between a voltage limit of 4.2–1 V. The 3-electrode cell was charged and discharged at a rate of C/10.

For cycle life tests in 2-electrode cells, constant current charge at a rate of C/5 was applied to 4.2 V. Constant voltage charge at 4.2 V was applied until the current was < 0.02C. Constant current discharge at C/5 was applied to 1 V.

The C-rates was based on a nominal specific capacity of 130 $mAhg^{-1}$ for the cathode. The capacity of the pouch cells was around 10 mAh.

S7.2 Results and Discussion.

3-electrode cell in Figure S7.2.1a and Figure S7.2.1b depicts the voltage profile of a 3-electrode cell of the second cycle for the two borate salts $Na[B(pp)_2]$ and $Na[B(hfip)_4]\cdot DME$.

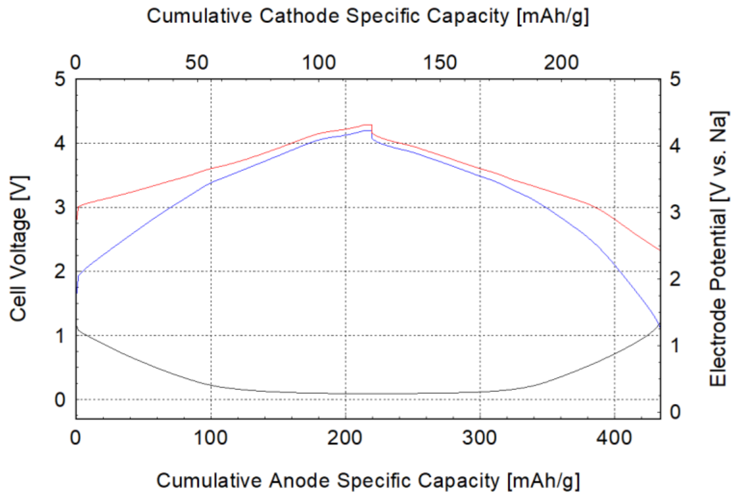

Figure S7.2.1a Voltage profile for 1 M $Na[B(pp)_2]$. The cell voltage trace is in blue, the cathode voltage profile versus a Na metal reference electrode in red and the anode voltage profile in black.

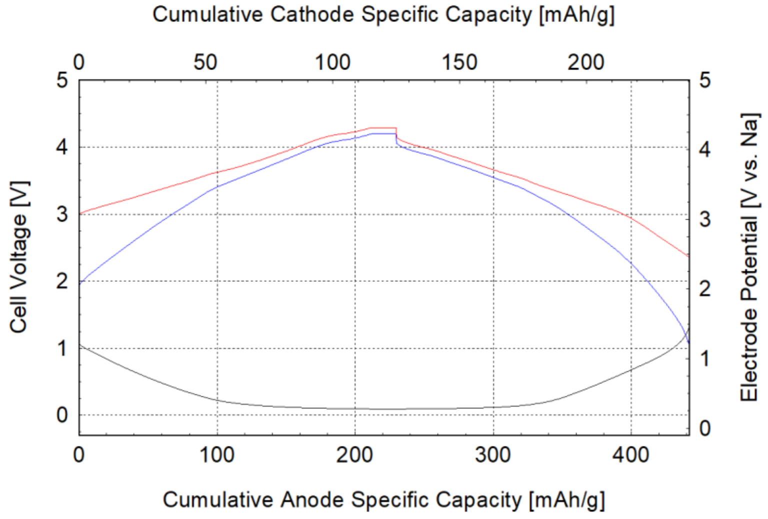

Figure S7.2.1b Voltage profile for 1 M $Na[B(hfip)_4]\cdot DME$. The cell voltage trace is in blue, the cathode voltage profile versus a Na metal reference electrode in red and the anode voltage profile in black.

The voltage profiles for both electrolyte systems are similar. In both cases, on top of charge, the cathode voltage was around 4.25 V and the anode voltage remains well above 0 V vs. Na/Na^+, indicating that both systems are well balanced and sodium plating is not likely to occur at the anode surface. On discharge, the cathode voltage was 2.4 V and the anode voltage was around 1.15 V.

2-electrode cell

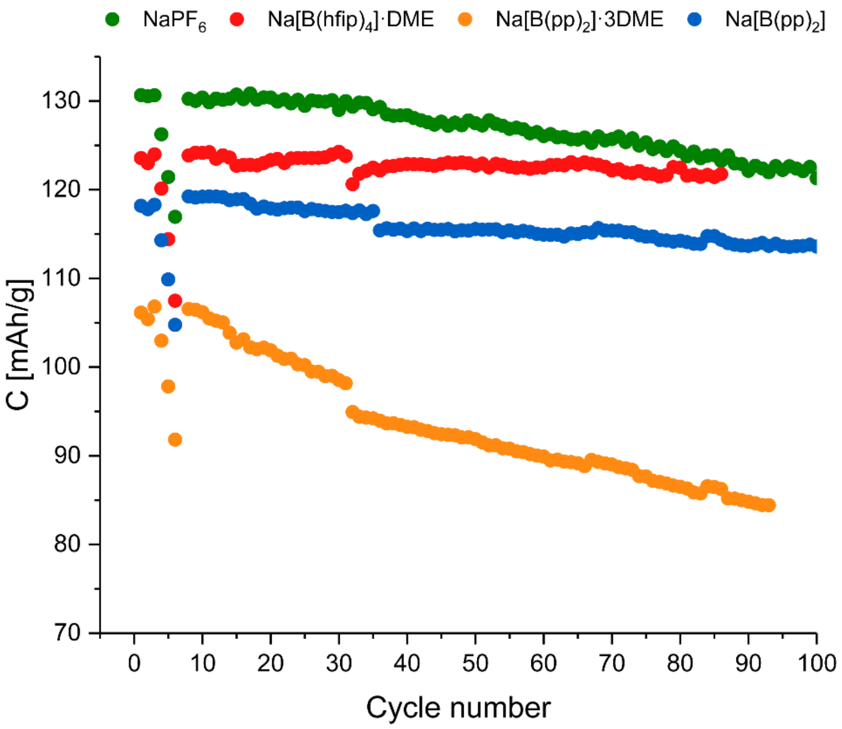

Figure S7.2.2 Capacity vs. cycle number of electrolytes Na[B(hfip)$_4$]·DME (**1a**), Na[B(pp)$_2$]·3DME (**1b**), Na[B(pp)$_2$] (**1b'**) and NaPF$_6$ in EC:DEC:PC (1:2:1 wt/wt). Voltage range 1.0–4.2 V and rate C/5.

Note, the apparent discrepancy between the degradation rate of Na[B(hfip)$_4$]·DME based electrolyte in coin cells and pouch cells can be explained by the significantly larger electrolyte/electrode volume ratio in coin cells, which may increase the solubility of the SEI.

S8 Density functional theory (DFT).

S8.1 DFT experimental.

For each anion, a large number of ion-pairs with Na^+ were generated automatically using SECIL.[12] In each case, the initial optimisation pass was done with xtb version 6.4.0.[13,14] Selected (on basis of having distinct structures and energies) ion-pairs, along with the anions, were then optimised at the B3LYP-D/def2-TZVPP level of theory[15–17] with ORCA version 4.2.1.[18] In each case, the dispersion-corrected B3LYP functional was used with the def2-TZVPP basis set. As the RIJCOSX approximation was used, the def2/J auxillary basis set was used.[19] In each case the frequencies were inspected to ensure that no imaginary frequencies were found. The full list of options used for a standard calculation is: B3LYP def2-TZVPP def2/J TightSCF RIJCOSX D3BJ Freq GridX4 Grid4 FinalGrid5 TightOpt

The ion-pair dissociation energy was calculated from the final energetic values (ΔE_d = E(Na)+E(An) − E(IP)). Oxidation potentials were calculated by taking the final anion structure and doing a single point energy calculation on the corresponding oxidised system. All calculations were done in the gas phase.

S8.2 DFT Results.

Table S8.2.1 Ion-pair dissociation energies of the Na-salts studied and their corresponding oxidation potentials. The dissociation energies are reported both as electronic, ΔE_d, and Gibb free energies, ΔG_d.

Anion	ΔE_d (kJ mol^{-1})	ΔG_d (kJ mol^{-1})	Oxidation potential (eV)	Oxidation potential vs. Na^+/Na (V)
$[B(hfip)_4]^-$	504	472	6.52	4.79
$[B(pp)_2]^-$	480	448	6.36	4.63
$[B(OCH_2(CF2)_2CF_3)_4]^-$	521	484	6.09	4.36
$[B(OPh^F)_4]^-$	495	461	5.13	3.40
$[B(OMe)_4]^-$	614	579	3.79	2.06
$[B(OPh)_4]^-$	530	496	3.91	2.18
$[B(O^iPr)_4]^-$	594	560	3.82	2.09

S9 References.

[1] D. M. C. Ould, S. Menkin, C. A. O'Keefe, F. Coowar, J. Barker, C. P. Grey, D. S. Wright, *Angew. Chem. Int. Ed.* **2021**, *60*, 24882–24887.

[2] S. Bulut, P. Klose, I. Krossing, *Dalton Trans.* **2011**, *40*, 8114–8124.

[3] J. Luo, Y. Bi, L. Zhang, X. Zhang, T. L. Liu, *Angew. Chem. Int. Ed.* **2019**, *58*, 6967–6971.

[4] E. N. Keyzer, J. Lee, Z. Liu, A. D. Bond, D. S. Wright, C. P. Grey, *J. Mater. Chem. A* **2019**, *7*, 2677–2685.

[5] A. G. Campaña, N. Fuentes, E. Gómez-Bengoa, C. Mateo, J. E. Oltra, A. M. Echavarren, J. M. Cuevra, *J. Org. Chem*. **2007**, *72*, 8127–8130.

[6] I. M. Malkowsky, R. Fröhlich, U. Griesbach, H. Pütter, S. R. Waldvogel, *Eur. J. Inorg. Chem.* **2006**, 1690–1697.

[7] G. M. Sheldrick, *Acta Cryst.* **2015**, *A71*, 3–8.

[8] G. M. Sheldrick, *Acta Cryst*. **2015**, *C71*, 3–8.

[9] A. L. Spek, *Acta Cryst*. **2009**, *D65*, 148–155.

[10] R. H. Shreiner, K. W. Pratt, Primary Standards and Standard Reference Materials for Electrolytic Conductivity, *NIST Special Publication* 260–142, **2004**.

[11] J. T. S. Irvine, D. C. Sinclair, A. R. West, *Adv. Mater.* **1990**, *2*, 132–138.

[12] K. Angenendt, P. Johansson, *J. Phys. Chem. C* **2010**, *114*, 20577–20582.

[13] C. Bannwarth, E. Caldeweyher, S. Ehlert, A. Hansen, P. Pracht, J. Seibert, S. Spicher, S. Grimme, *WIREs Comput*. *Mol*. *Sci*. **2021**, *11*, e1493.

[14] C. Bannwarth, S. Ehlert, S. Grimme, *J. Chem. Theory Comput.* **2019**, *15*, 1652–1671.

[15] S. Grimme, S. Ehrlich, L. Goerigk, *J. Comput. Chem.* **2011**, *32*, 1456–1465.

[16] S. Grimme, J. Antony, S. Ehrlich, H. Krieg, *J. Chem. Phys.* **2010**, *132*, 154104.

[17] F. Weigend, R. Ahlrichs, *Phys. Chem. Chem. Phys.* **2005**, *7*, 3297–3305.

[18] F. Neese, *WIREs Comput Mol Sci* **2018**, *8*, e1327.

[19] F. Weigend, *Phys. Chem. Chem. Phys.* **2006**, *8*, 1057–1065.

S10 NMR spectra.

S10.1 NMR spectra of sodium borate complexes.

Figure S10.1.1 ^{1}H NMR (400 MHz, CD_3CN, 295 K) spectrum of Na[B(hfip)$_4$]·DME (**1a**).

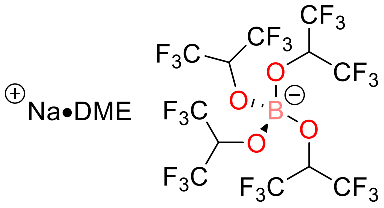

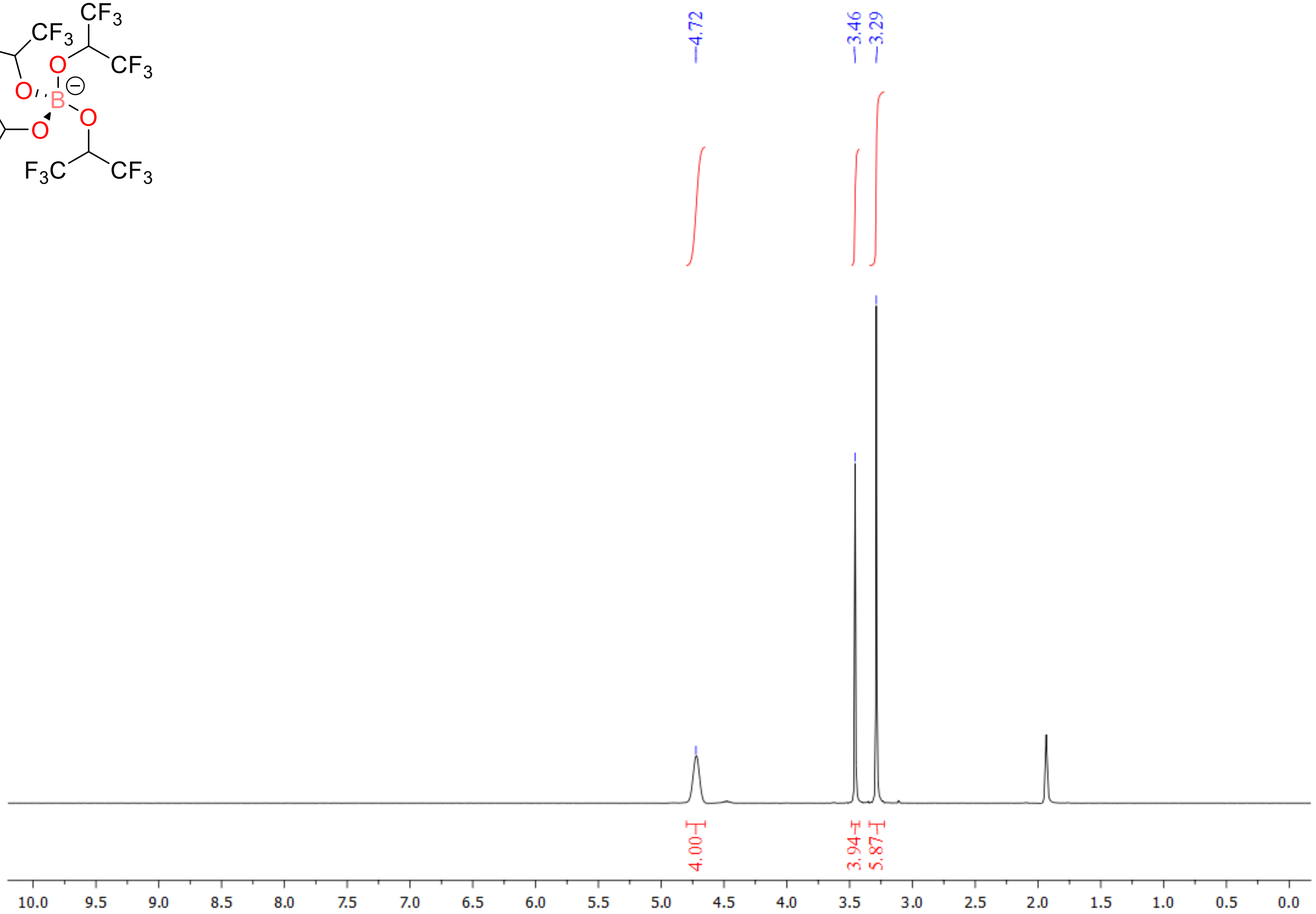

Figure S10.1.2 $^{13}C\{^1H\}$ NMR (101 MHz, CD_3CN, 295 K) spectrum of Na[B(hfip)$_4$]·DME (**1a**).

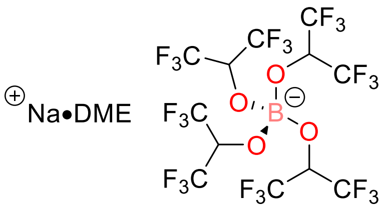

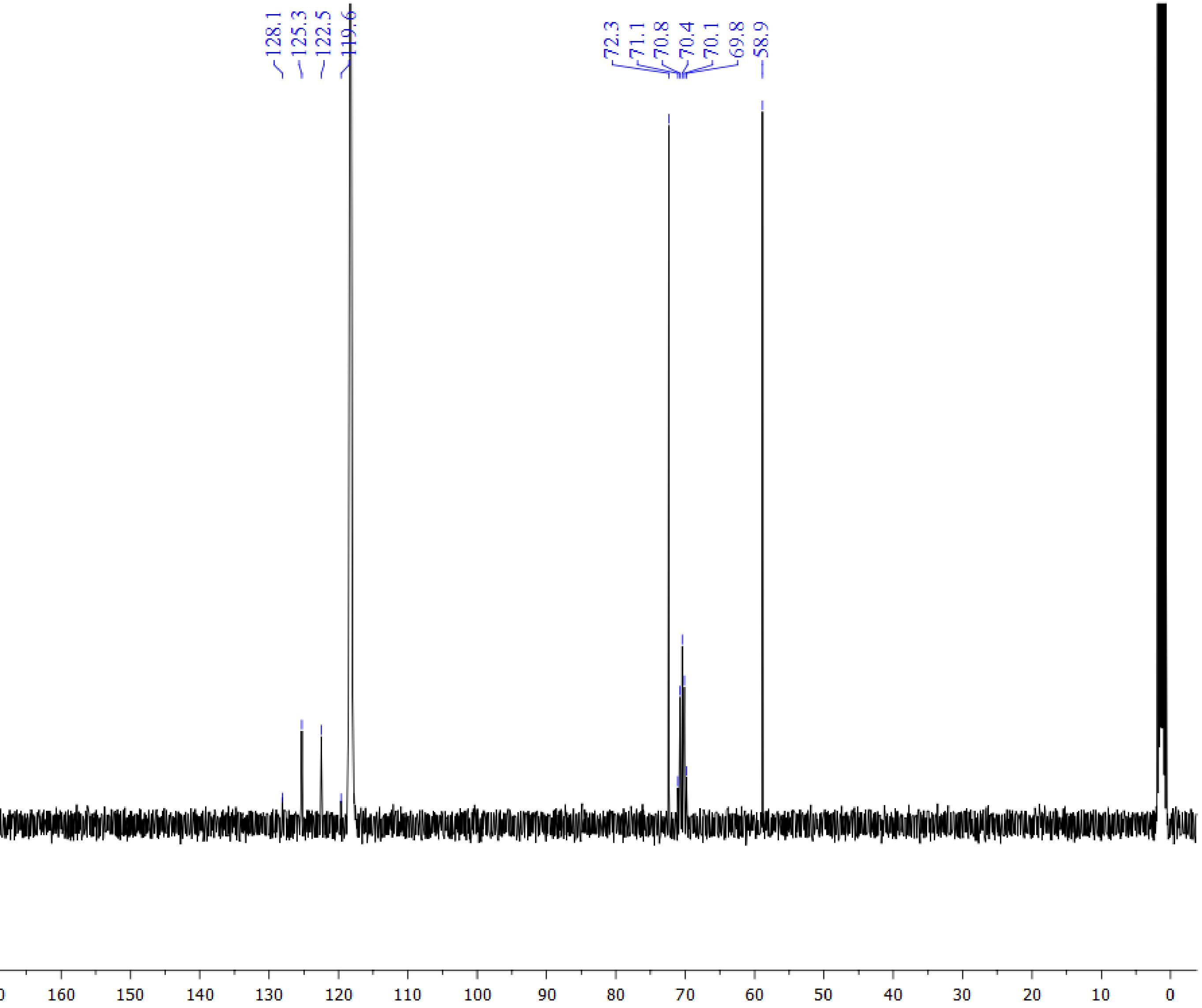

Figure S10.1.3 ^{11}B NMR (128 MHz, CD_3CN, 295 K) spectrum of Na[B(hfip)$_4$]·DME (**1a**).

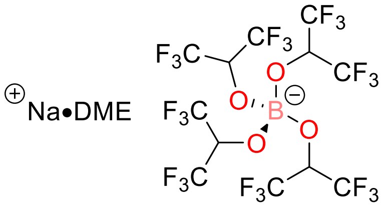

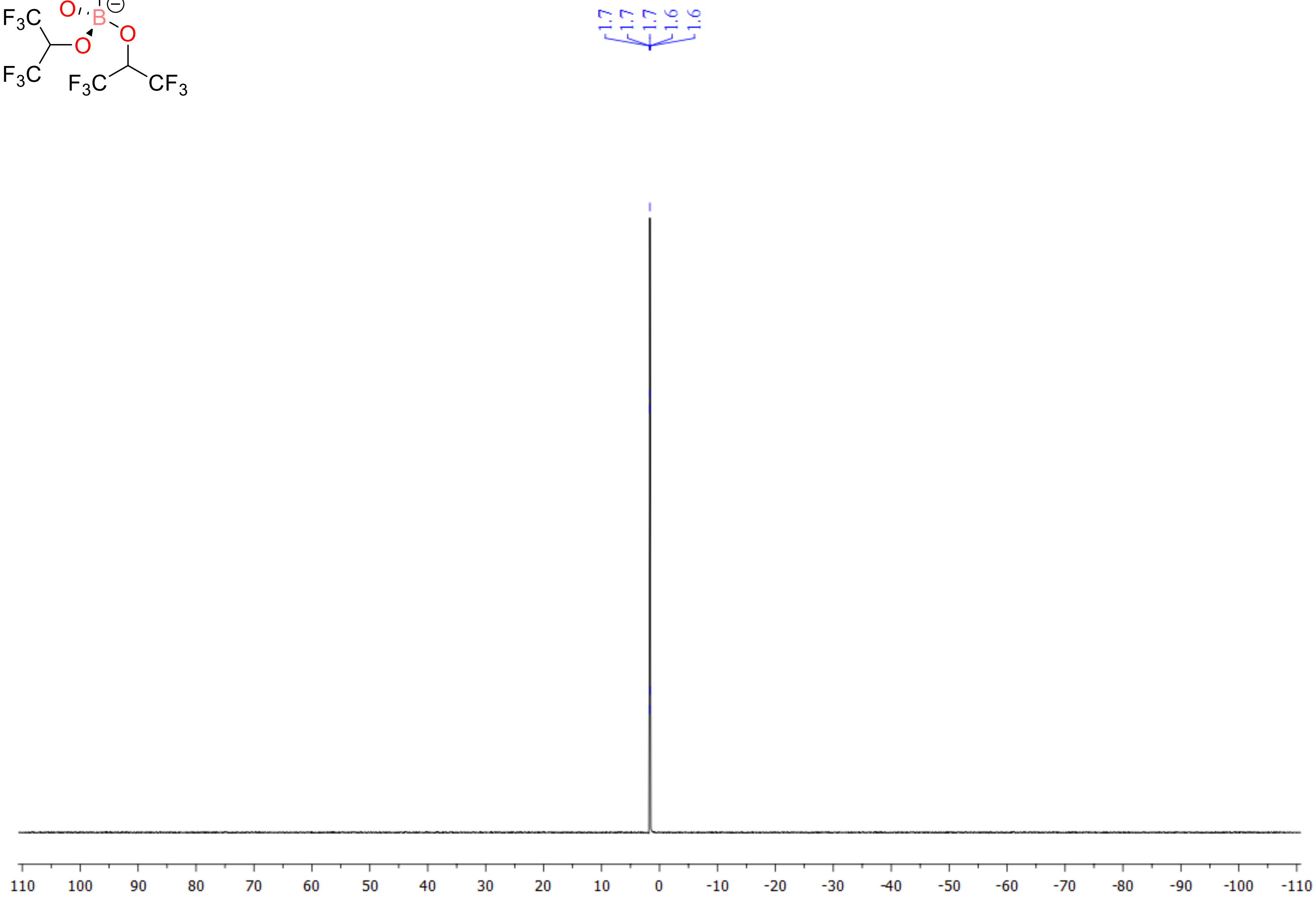

Figure S10.1.4 $^{19}F\{^{1}H\}$ NMR (376 MHz, CD_3CN, 295 K) spectrum of Na[B(hfip)$_4$]·DME (**1a**).

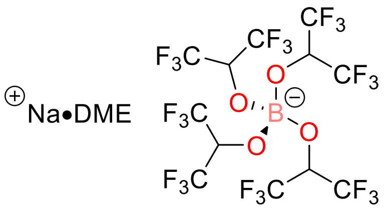

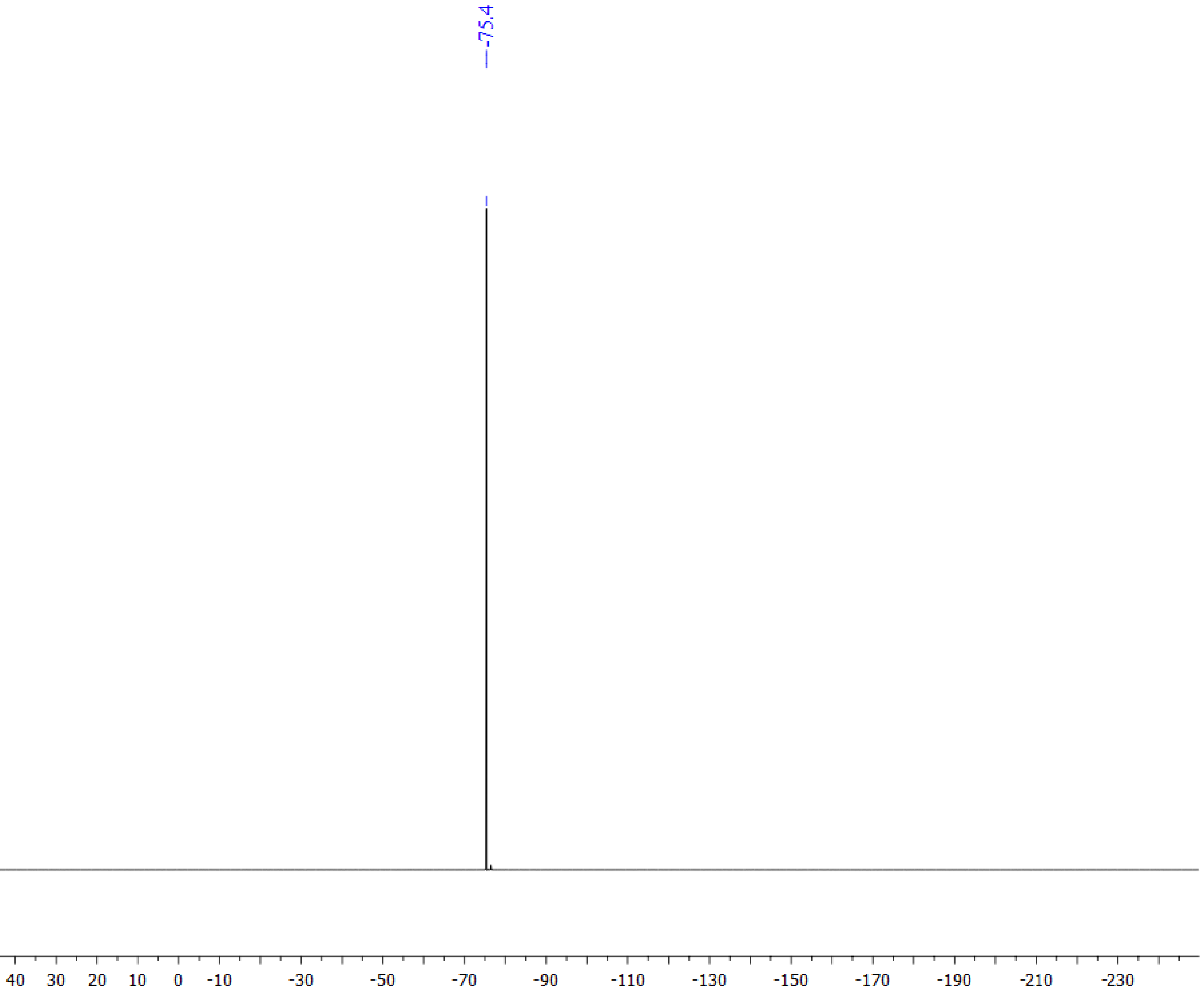

Figure S10.1.5 ^{1}H NMR (400 MHz, CD_3CN, 295 K) spectrum of Na[B(pp)$_2$]·3DME (**1b**).

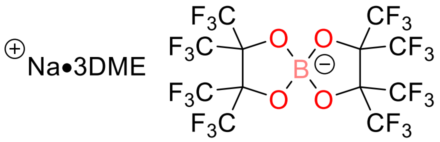

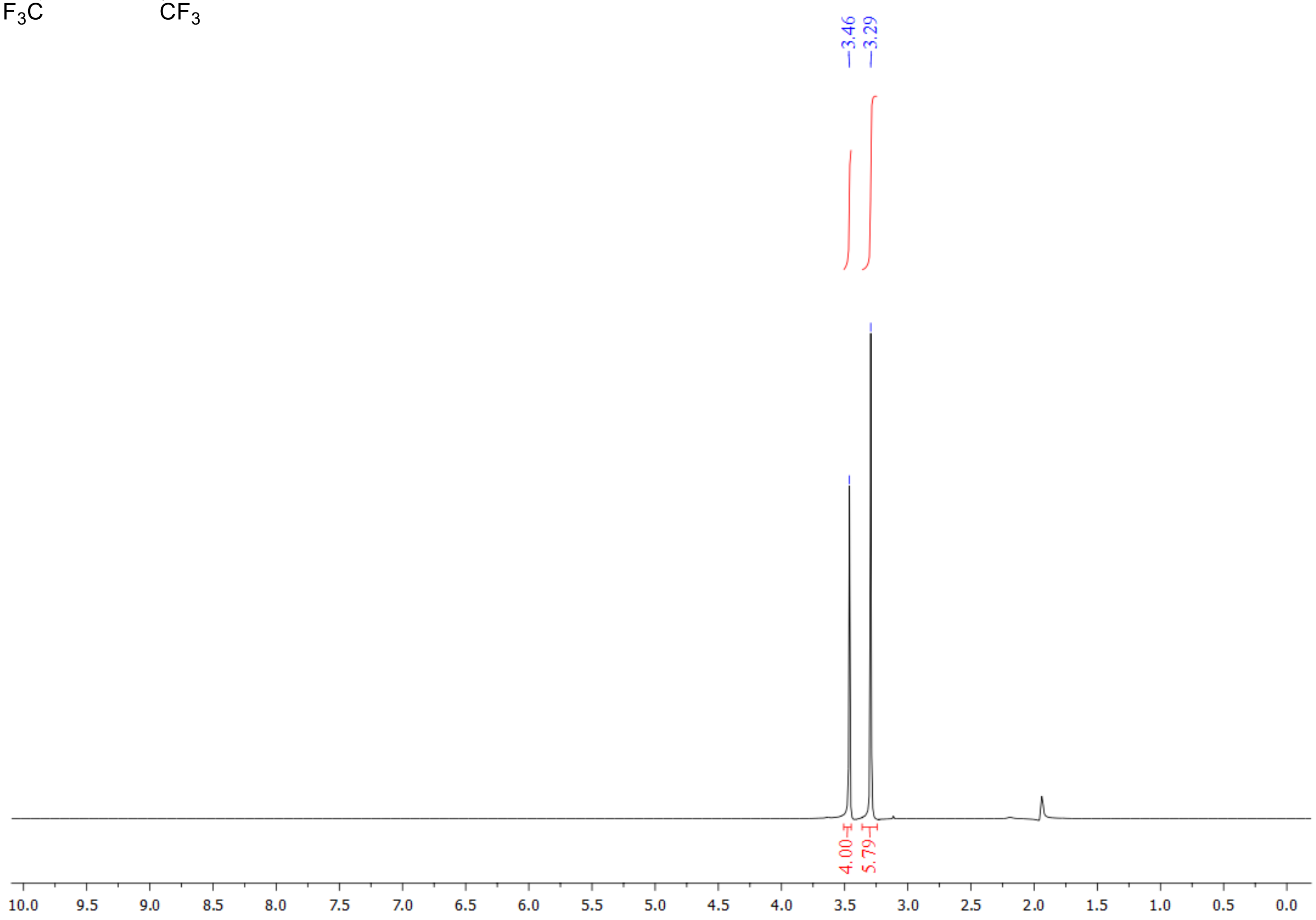

Figure S10.1.6 $^{13}C\{^1H\}$ NMR (101 MHz, $(CD_3)_2SO$, 295 K) spectrum of Na[B(pp)$_2$]·3DME (**1b**).

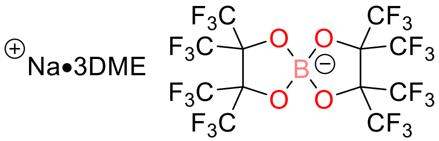

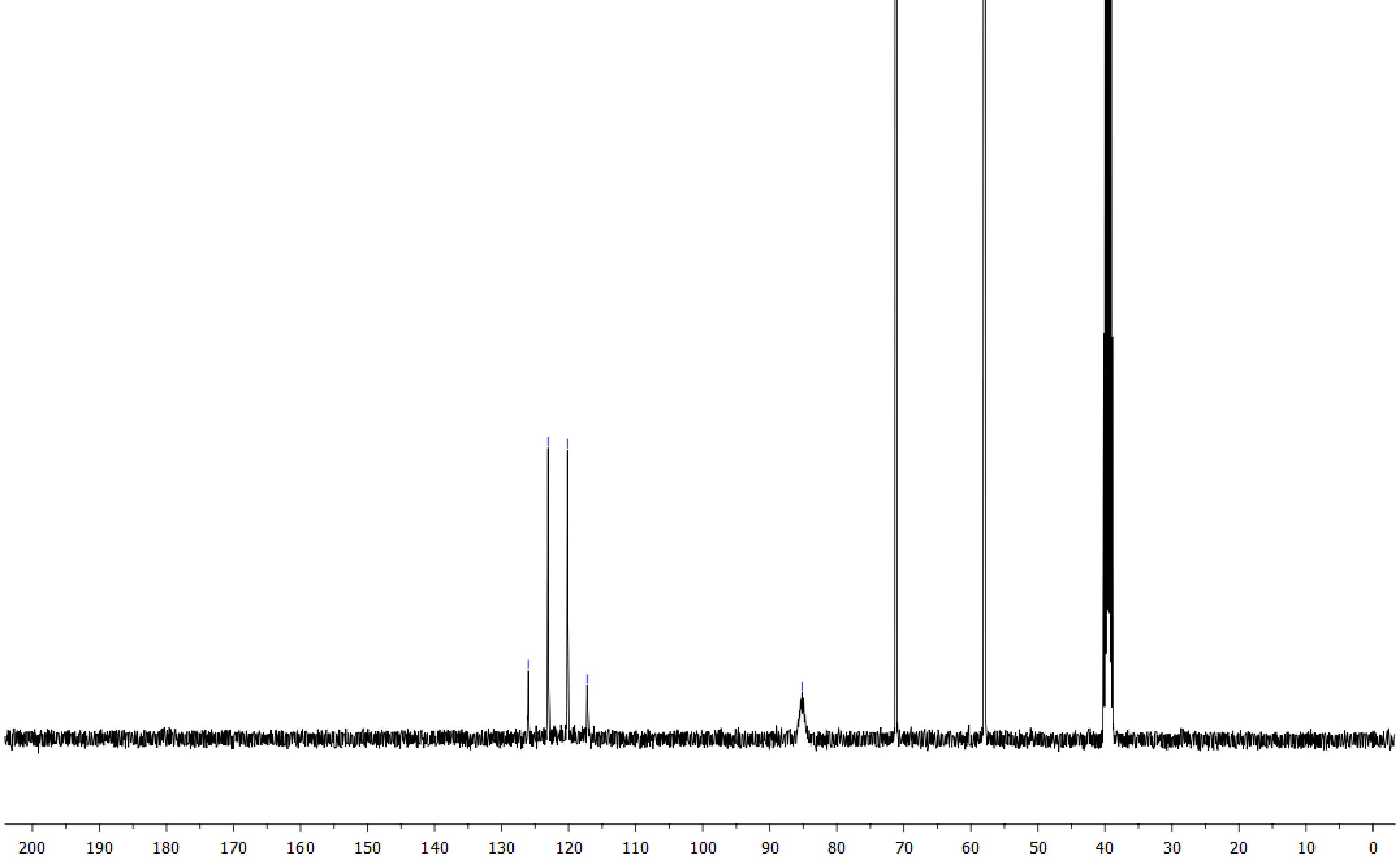

Figure S10.1.7 ^{11}B NMR (128 MHz, CD_3CN, 295 K) spectrum of $Na[B(pp)_2]\cdot 3DME$ (**1b**).

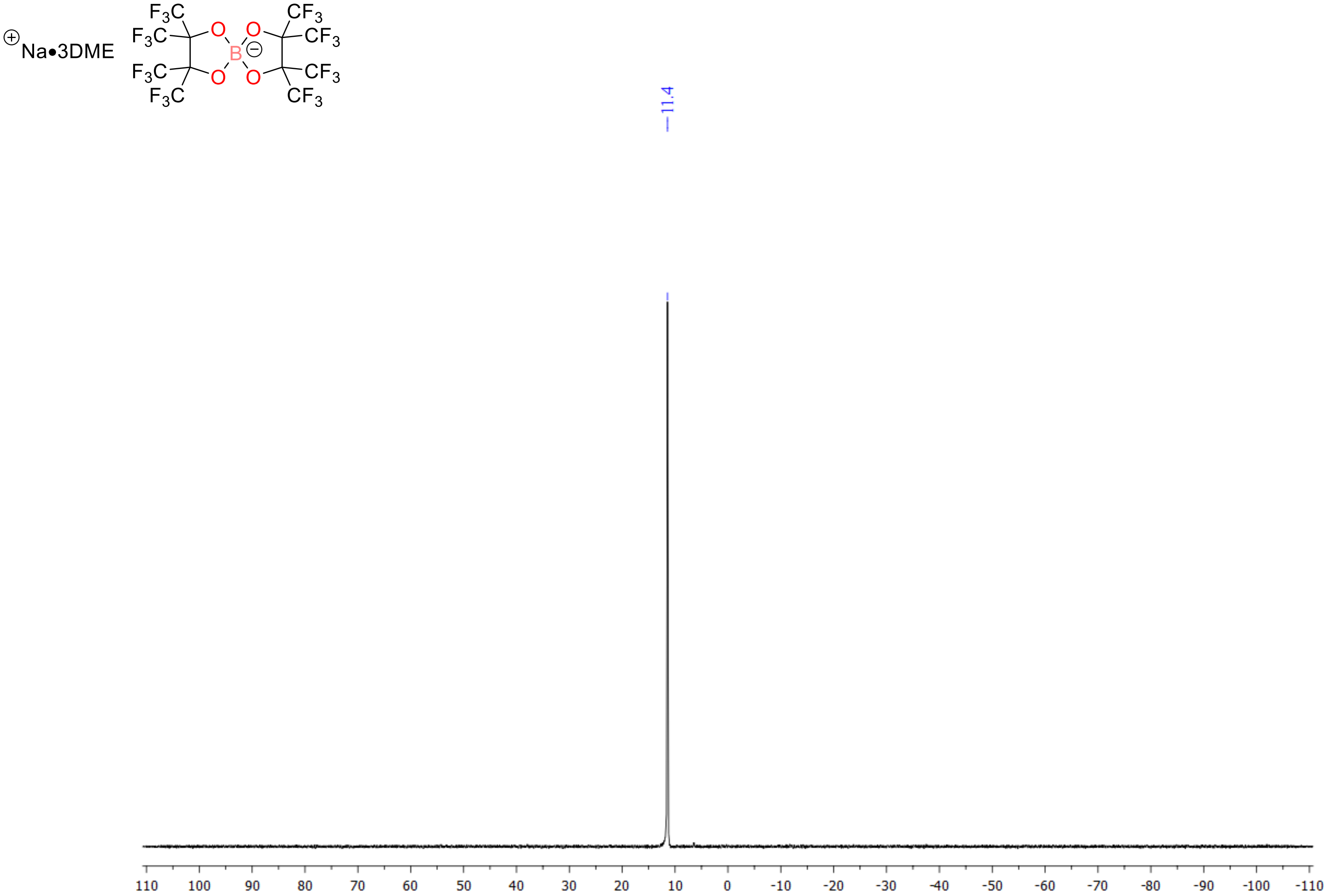

Figure S10.1.8 ^{19}F NMR (376 MHz, CD_3CN, 295 K) spectrum of Na[B(pp)$_2$]·3DME (**1b**).

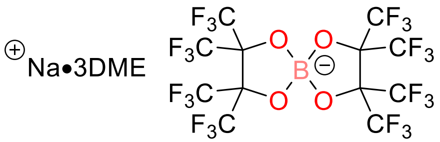

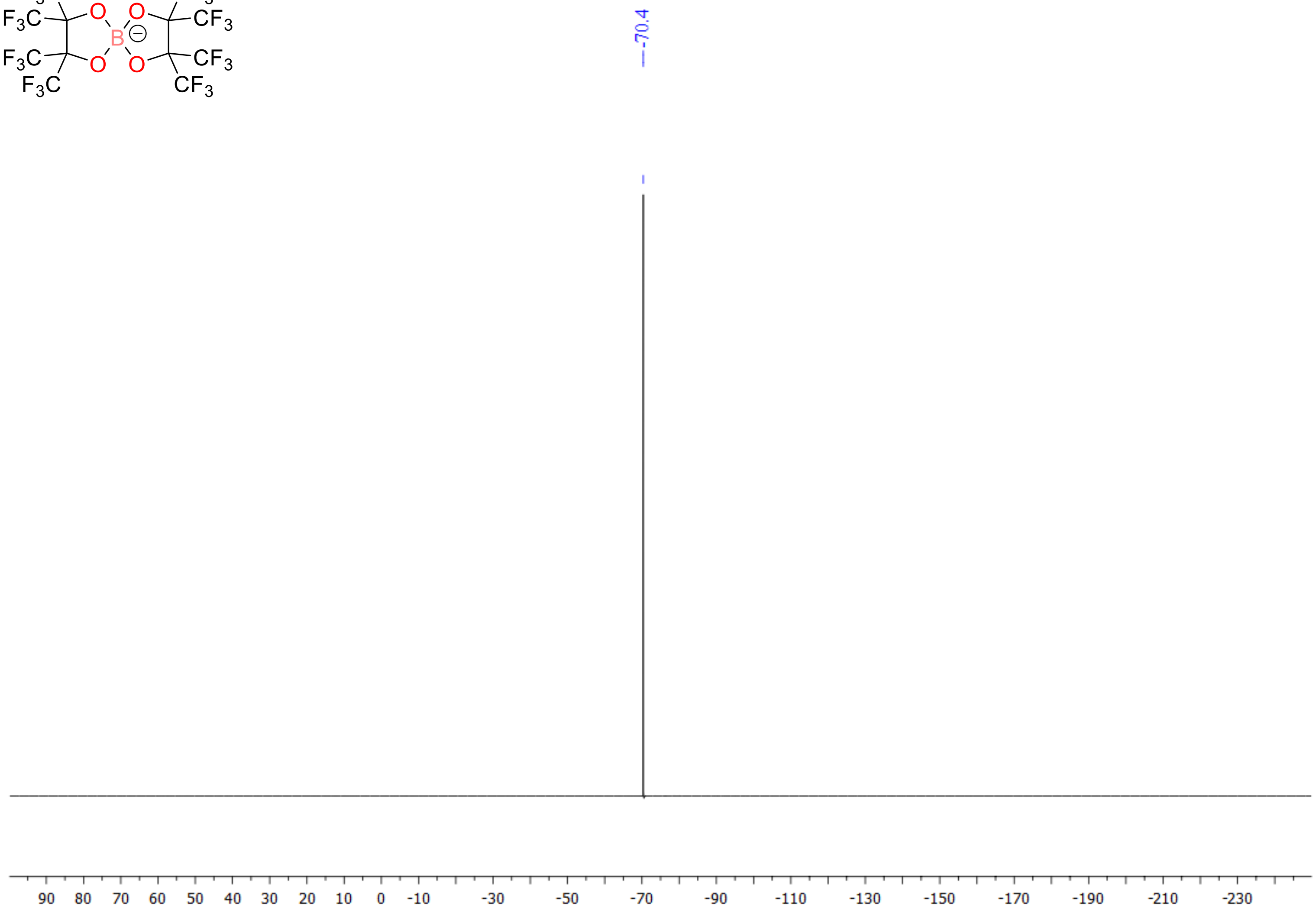

Figure S10.1.9 ^{1}H NMR (400 MHz, CD_3CN, 295 K) spectrum of Na[B(pp)$_2$] (**1b'**).

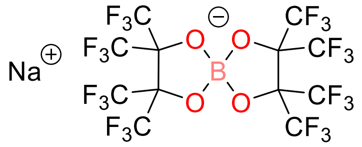

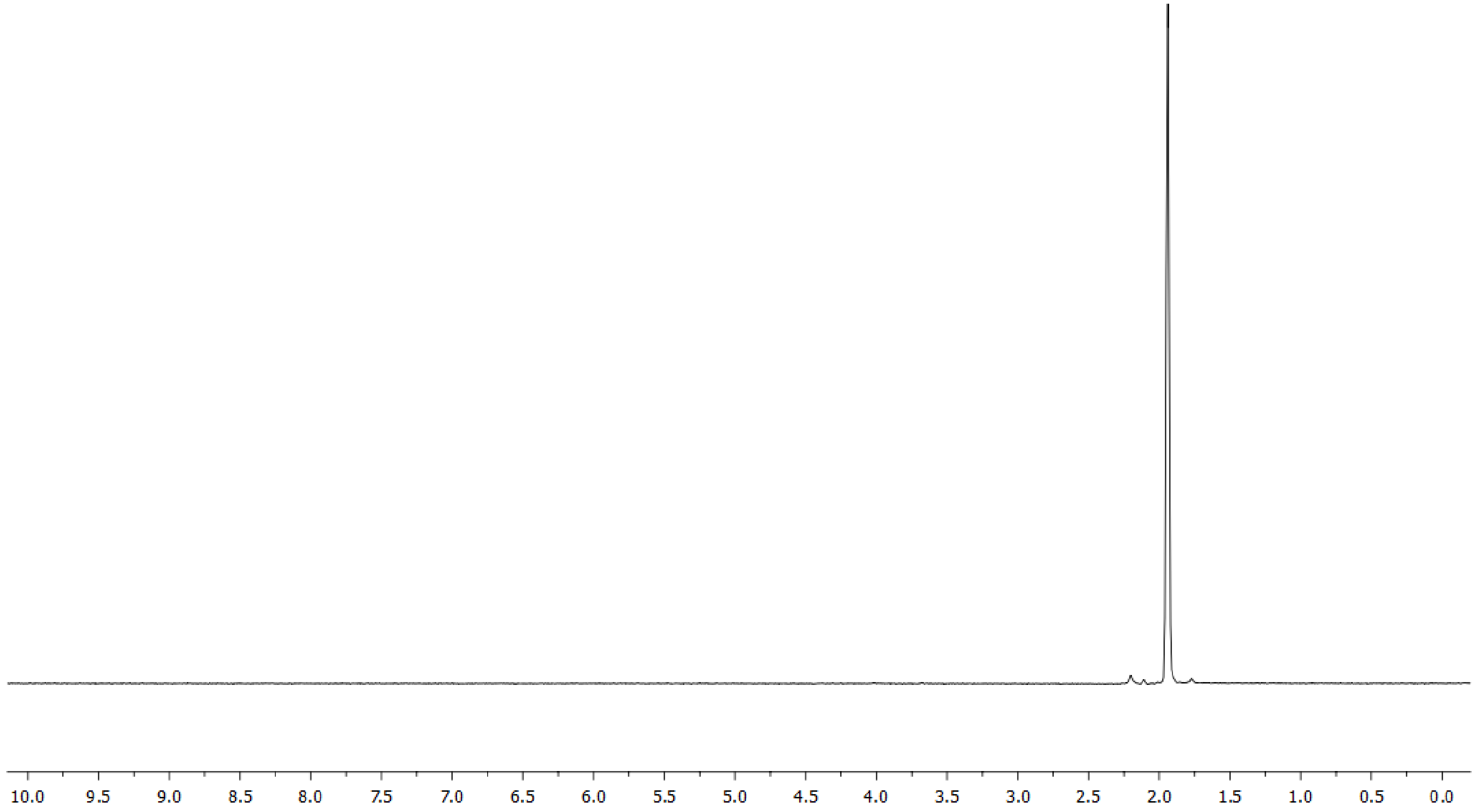

Figure S10.1.10 ^{11}B NMR (128 MHz, CD_3CN, 295 K) spectrum of Na[B(pp)$_2$] (**1b'**).

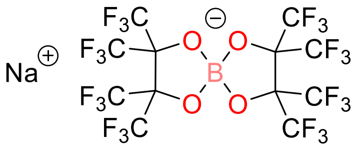

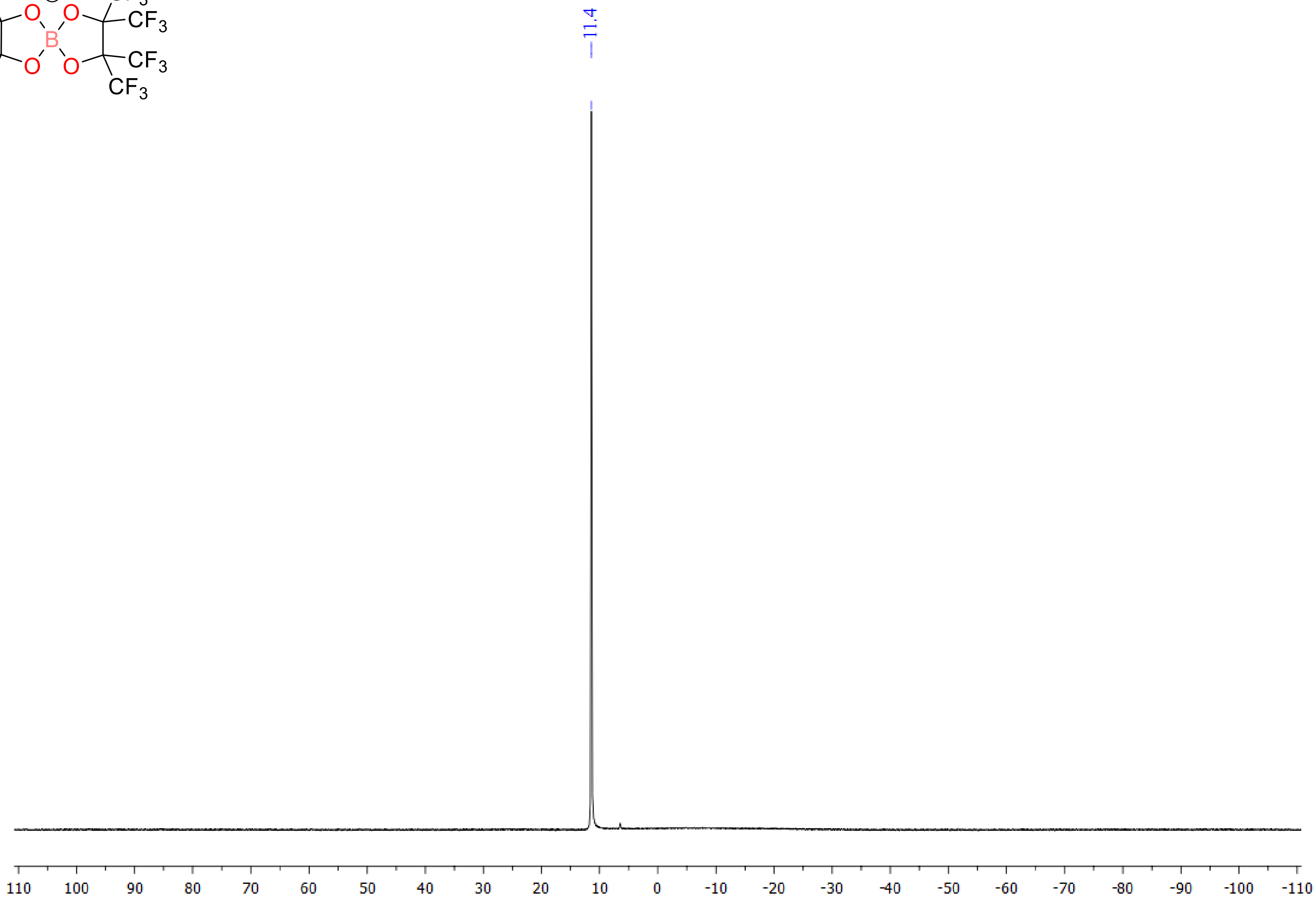

Figure S10.1.11 ^{19}F NMR (376 MHz, CD_3CN, 295 K) spectrum of Na[B(pp)$_2$] (**1b'**).

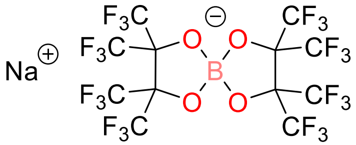

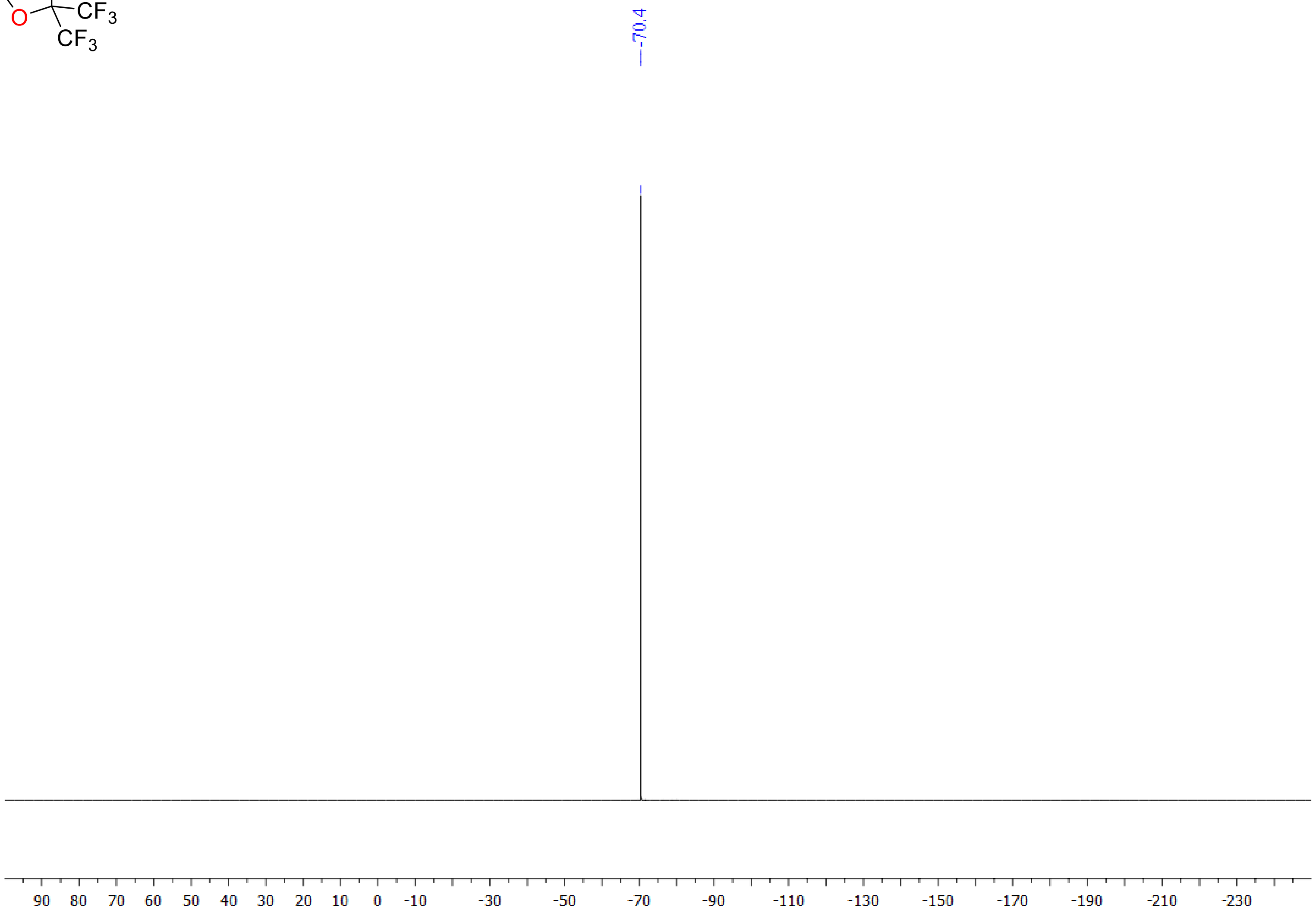

Figure S10.1.12 ^{1}H NMR (400 MHz, CD_3CN, 295 K) spectrum of $Na[B(OCH_2(CF_2)_2CF_3)_4]$ (**1c**).

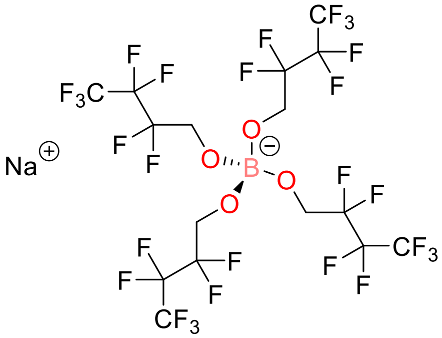

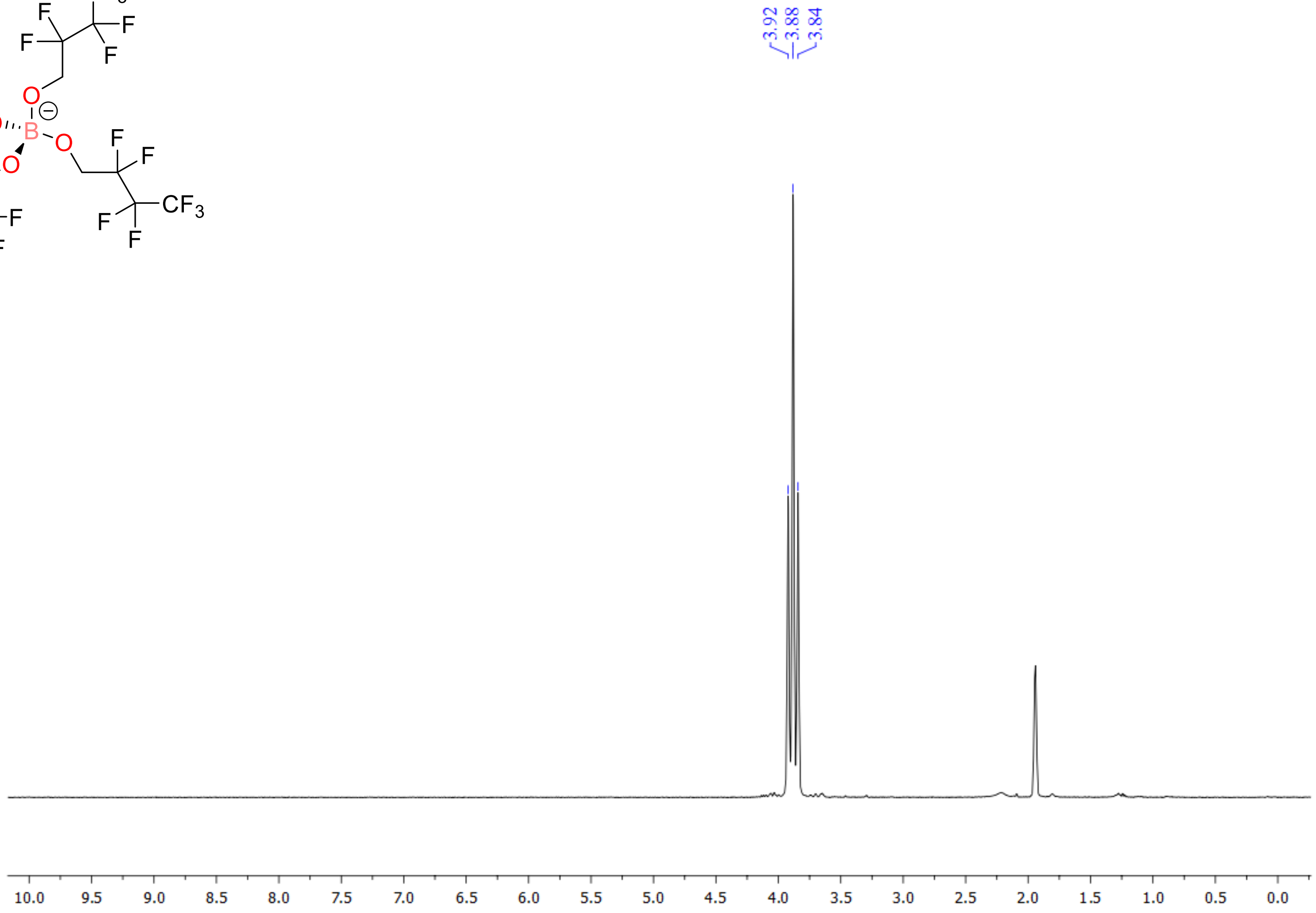

Figure S10.1.13 $^{13}C\{^{1}H\}$ NMR (101 MHz, $(CD_3)_2SO$, 295 K) spectrum of $Na[B(OCH_2(CF_2)_2CF_3)_4]$ (**1c**).

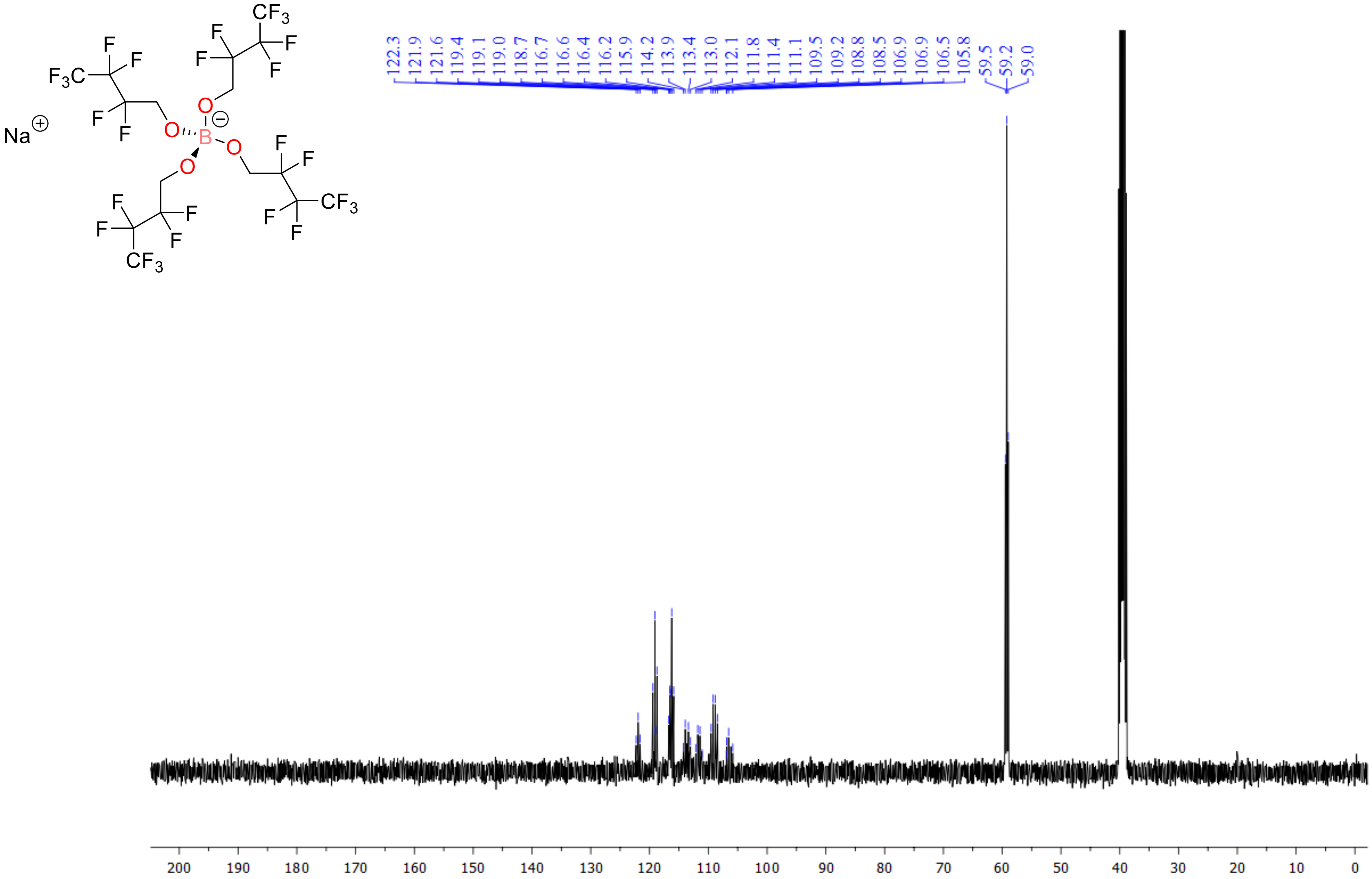

Figure S10.1.14 ^{11}B NMR (128 MHz, CD_3CN, 295 K) spectrum of $Na[B(OCH_2(CF_2)_2CF_3)_4]$ (**1c**).

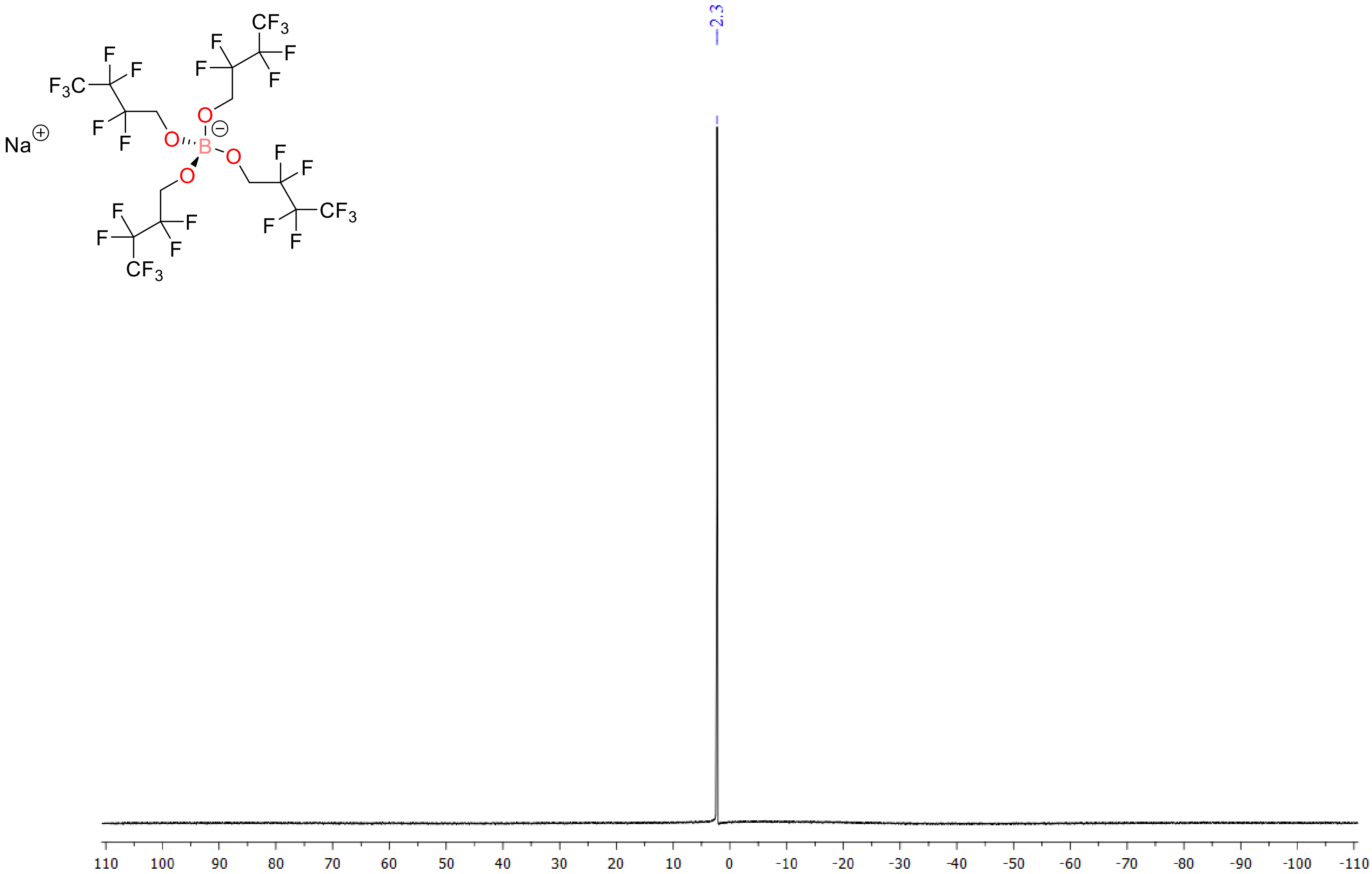

Figure S10.1.15 $^{19}F\{^1H\}$ NMR (376 MHz, CD_3CN, 295 K) spectrum of $Na[B(OCH_2(CF_2)_2CF_3)_4]$ (**1c**).

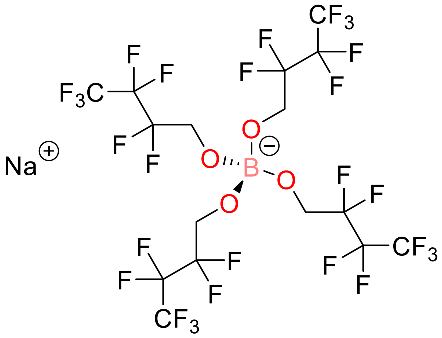

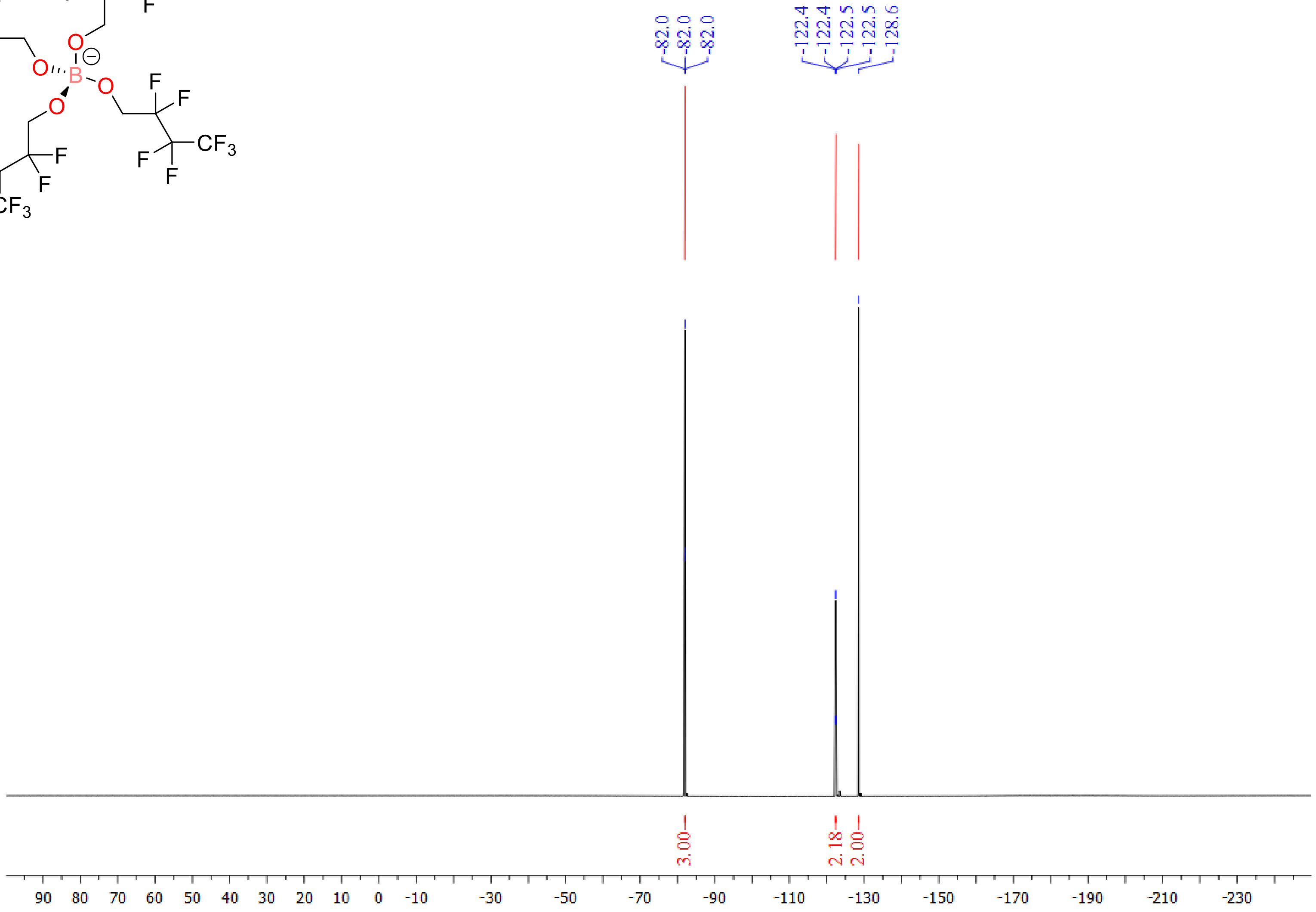

Figure S10.1.16 ^{1}H NMR (400 MHz, CD_3CN, 295 K) spectrum of $Na[B(OPh^F)_4]\cdot 3DME$ (**1d**).

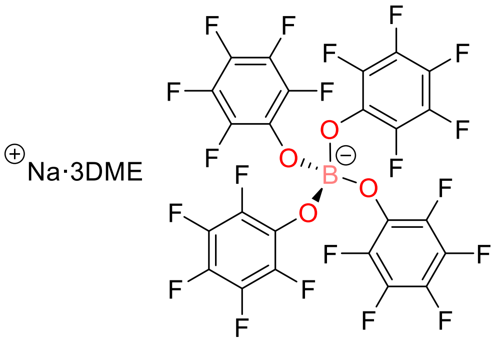

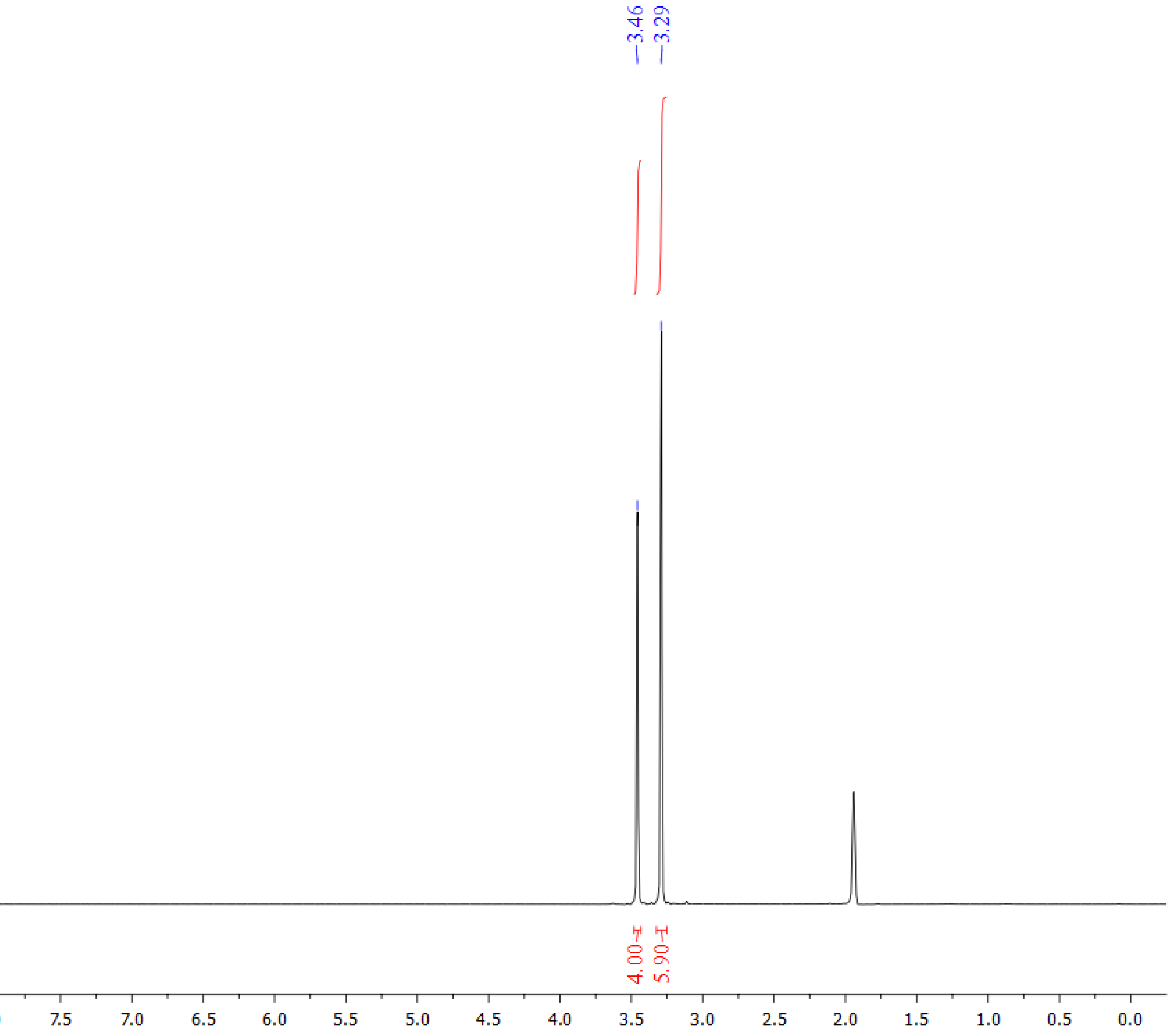

Figure S10.1.17 $^{13}C\{^{1}H\}$ NMR (101 MHz, $(CD_3)_2SO$, 295 K) spectrum of $Na[B(OPh^F)_4]\cdot 3DME$ (**1d**).

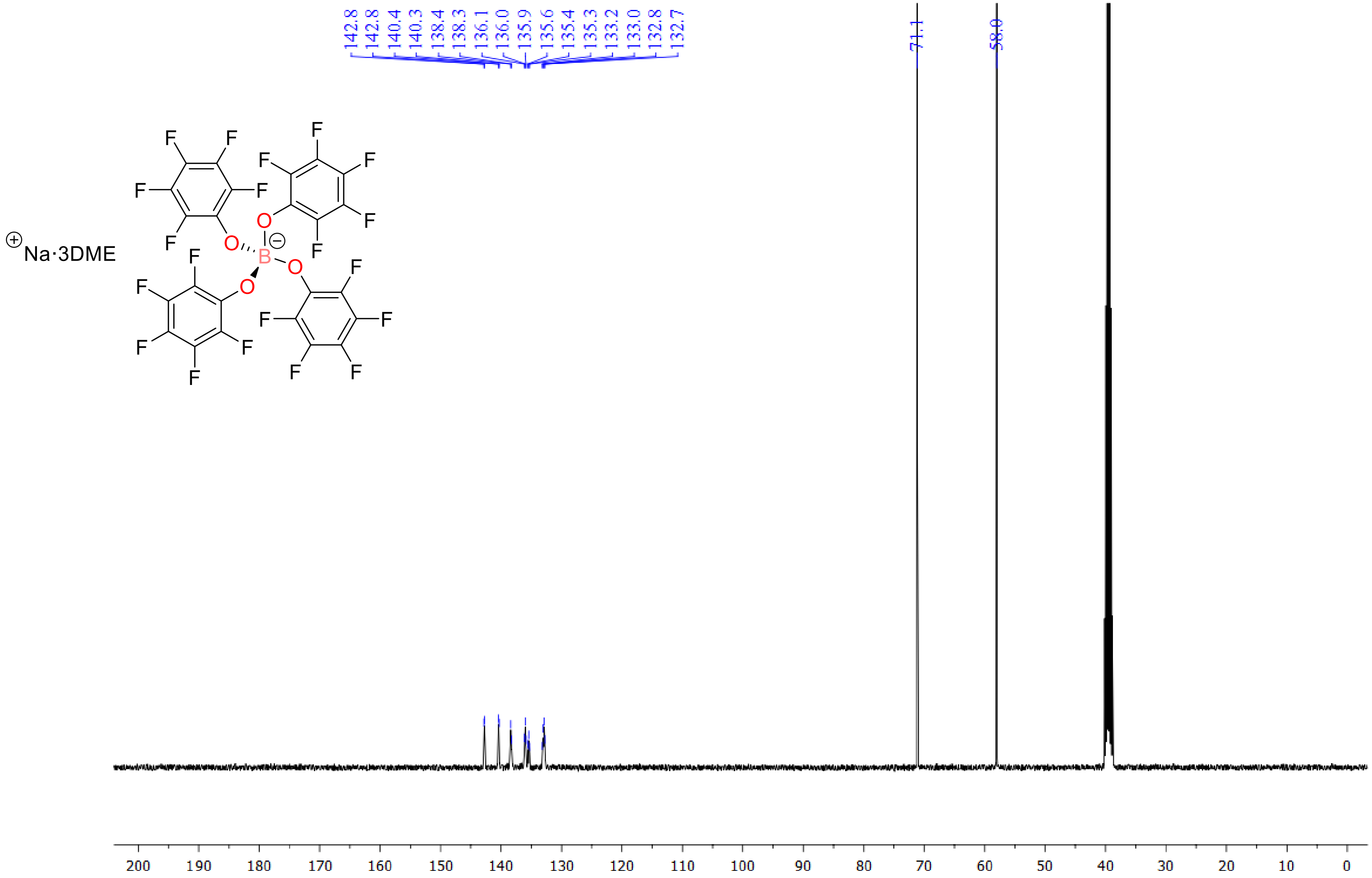

Figure S10.1.18 ^{11}B NMR (128 MHz, CD_3CN, 295 K) spectrum of Na[B(OPh^F)$_4$]·3DME (**1d**).

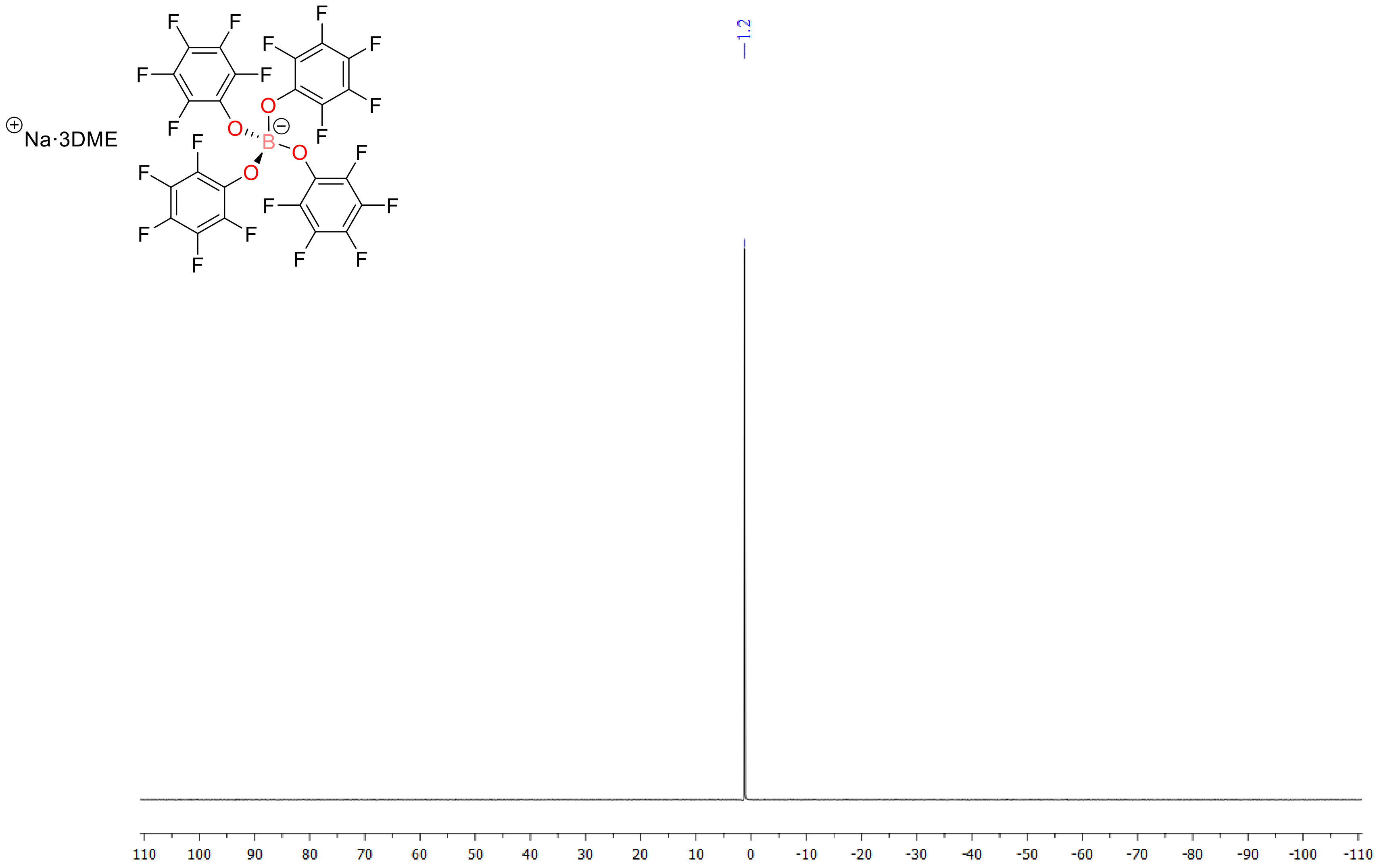

Figure S10.1.19 $^{19}F\{^{1}H\}$ NMR (376 MHz, CD_3CN, 295 K) spectrum of $Na[B(OPh^F)_4]\cdot 3DME$ (**1d**).

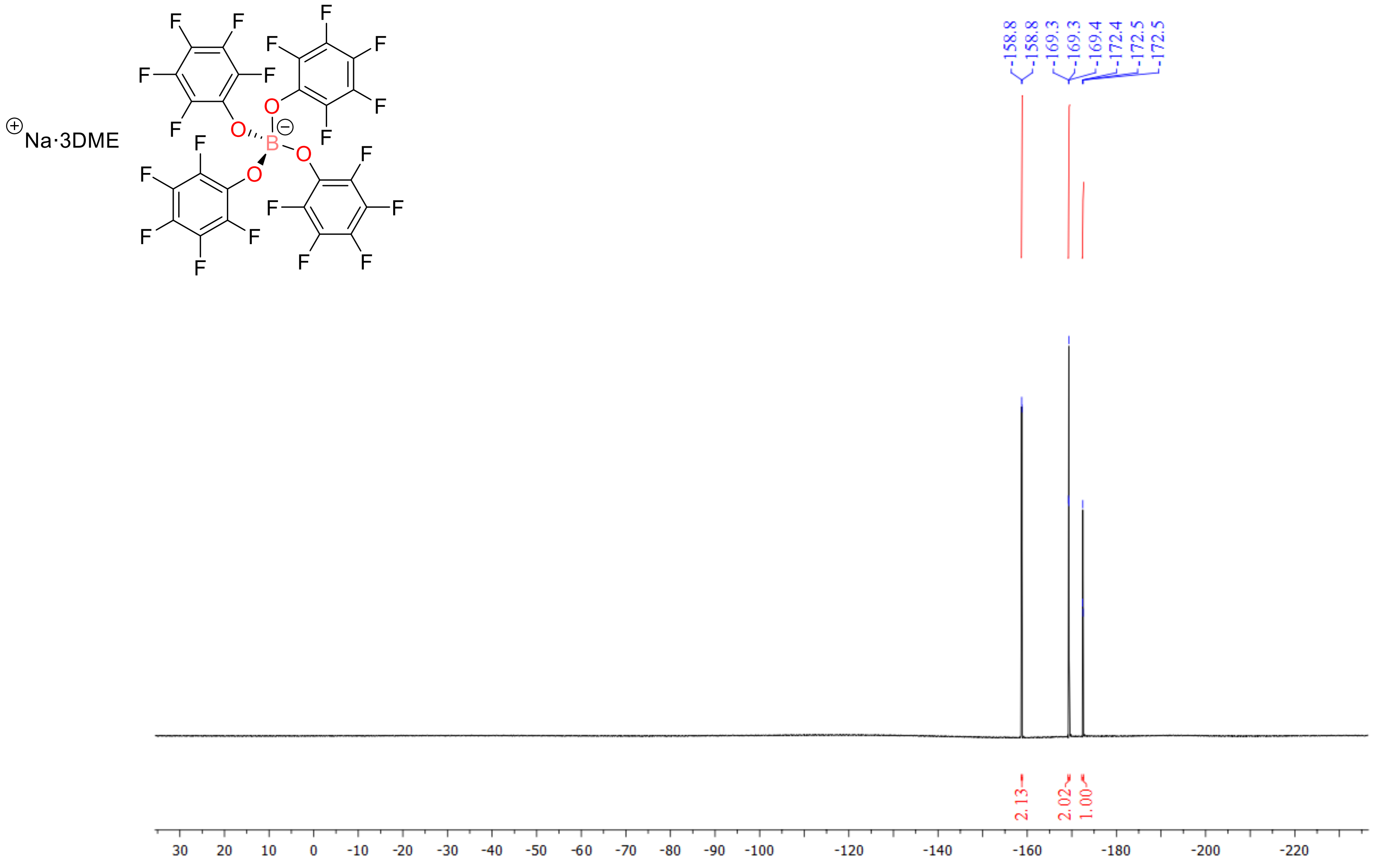

Figure S10.1.20 ^{1}H NMR (400 MHz, CD_3CN, 295 K) spectrum of $Na[B(OMe)_4]$ (**1e**).

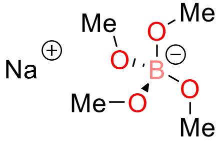

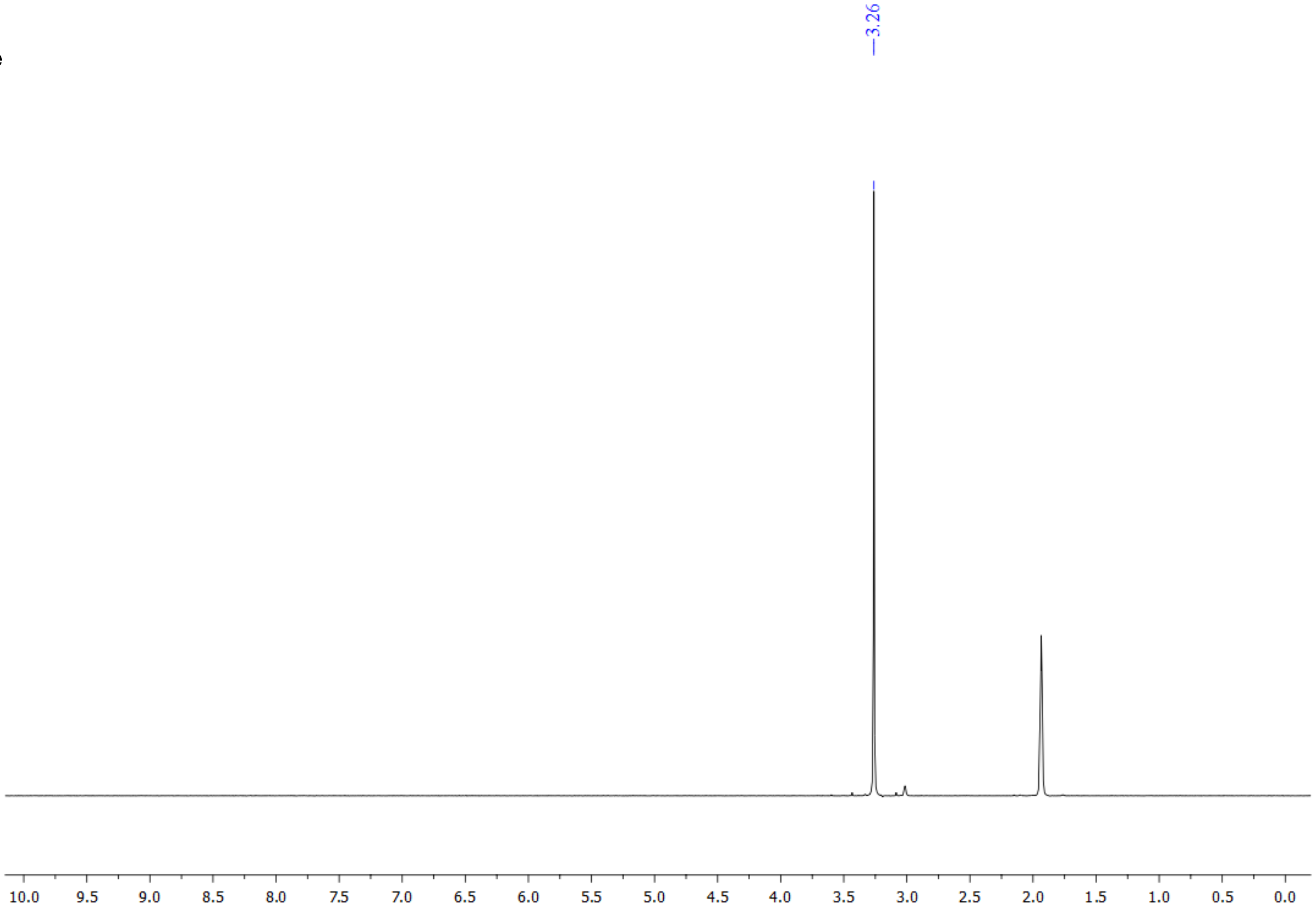

Figure S10.1.21 $^{13}C\{^{1}H\}$ NMR (101 MHz, CD_3CN, 295 K) spectrum of $Na[B(OMe)_4]$ (**1e**).

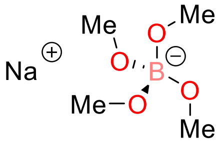

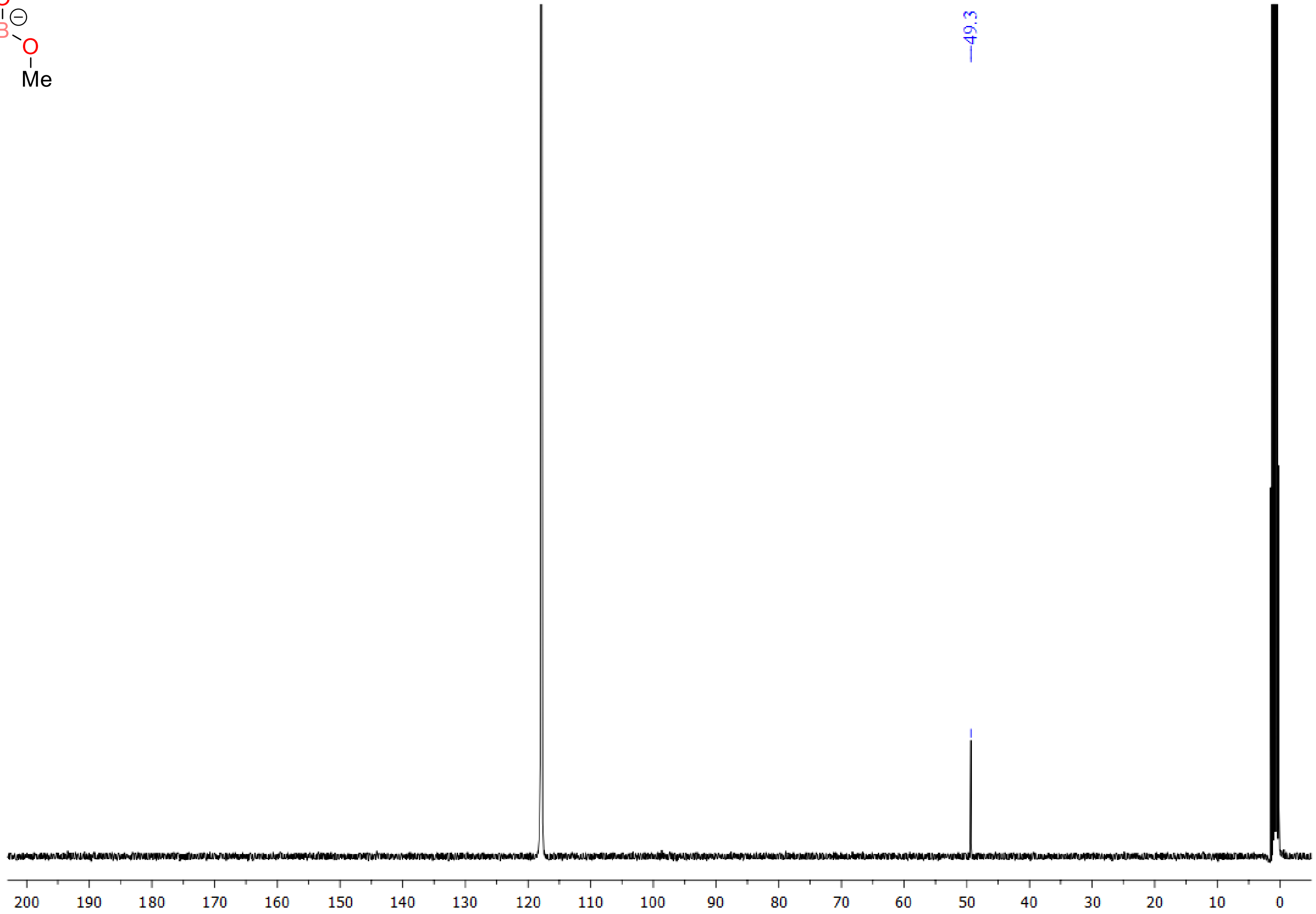

Figure S10.1.22 ^{11}B NMR (128 MHz, CD_3CN, 295 K) spectrum of $Na[B(OMe)_4]$ (**1e**).

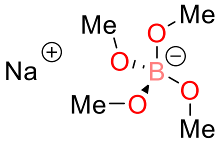

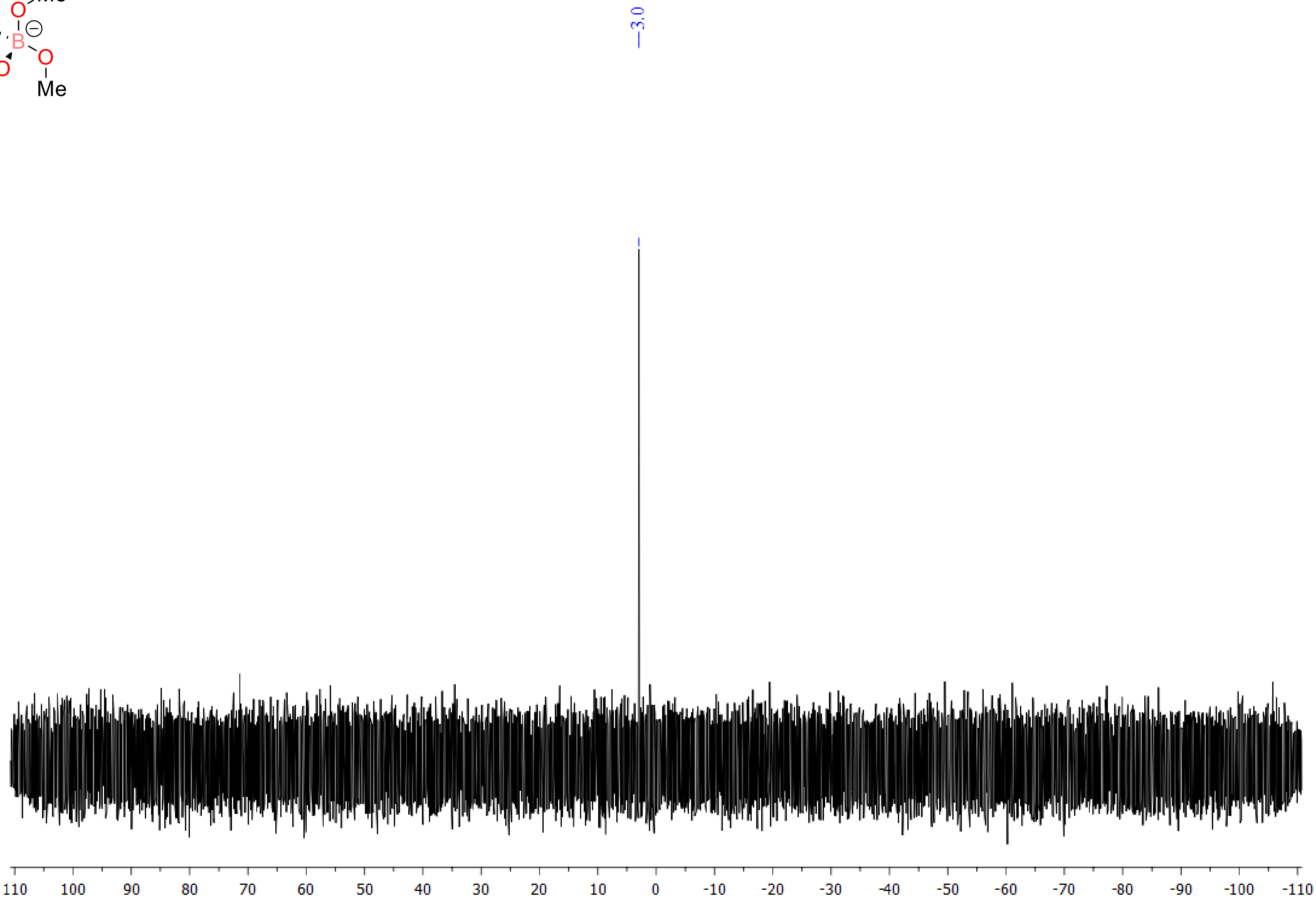

Figure S10.1.23 ^{1}H NMR (400 MHz, CD_3CN, 295 K) spectrum of Na[B(OPh)$_4$] (**1f**).

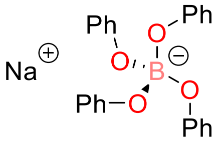

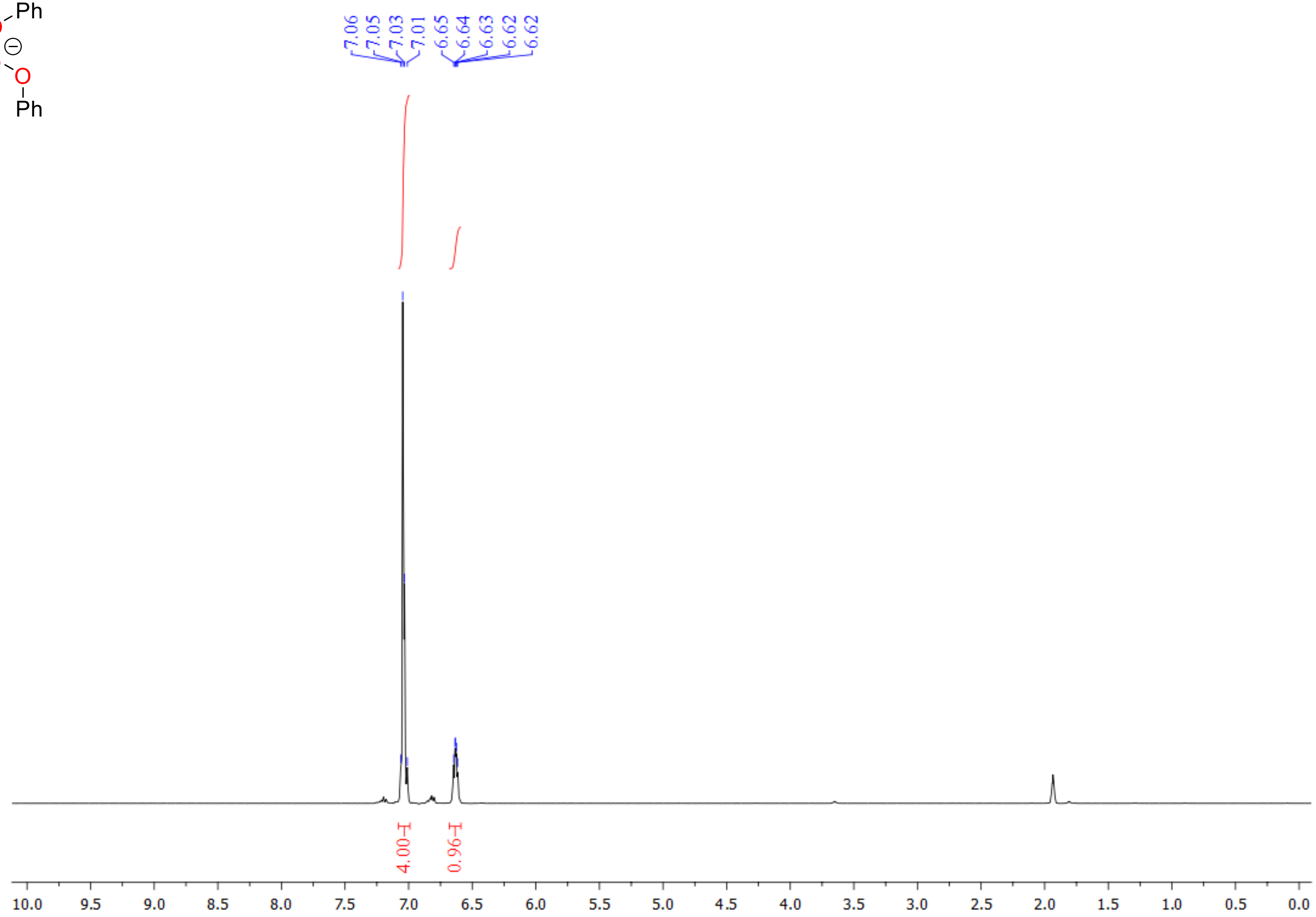

Figure S10.1.24 $^{13}C\{^1H\}$ NMR (101 MHz, CD_3CN, 295 K) spectrum of Na[B(OPh)$_4$] (**1f**).

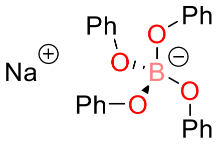

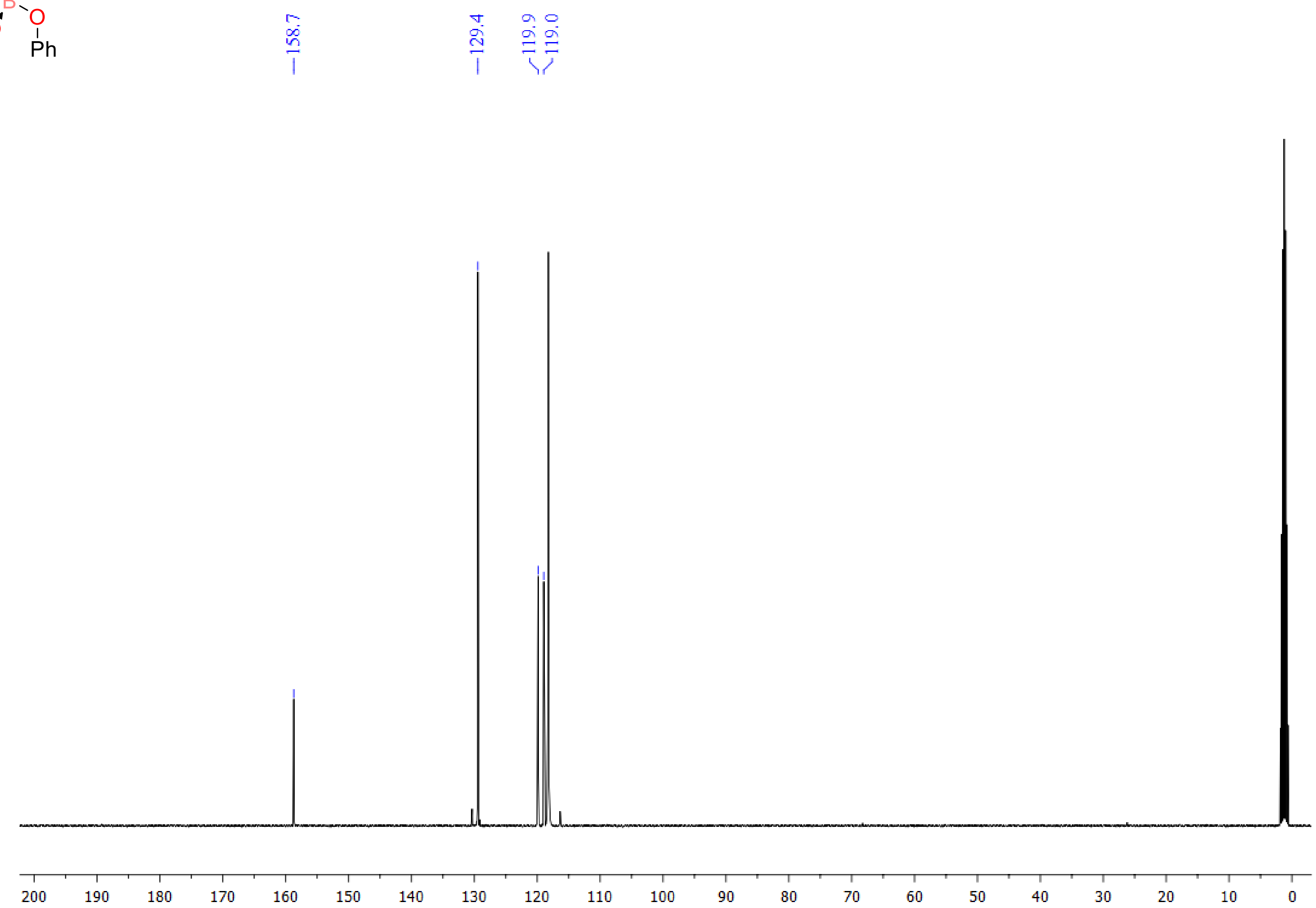

Figure S10.1.25 ^{11}B NMR (128 MHz, CD_3CN, 295 K) spectrum of Na[B(OPh)$_4$] (**1f**).

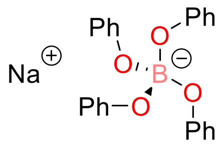

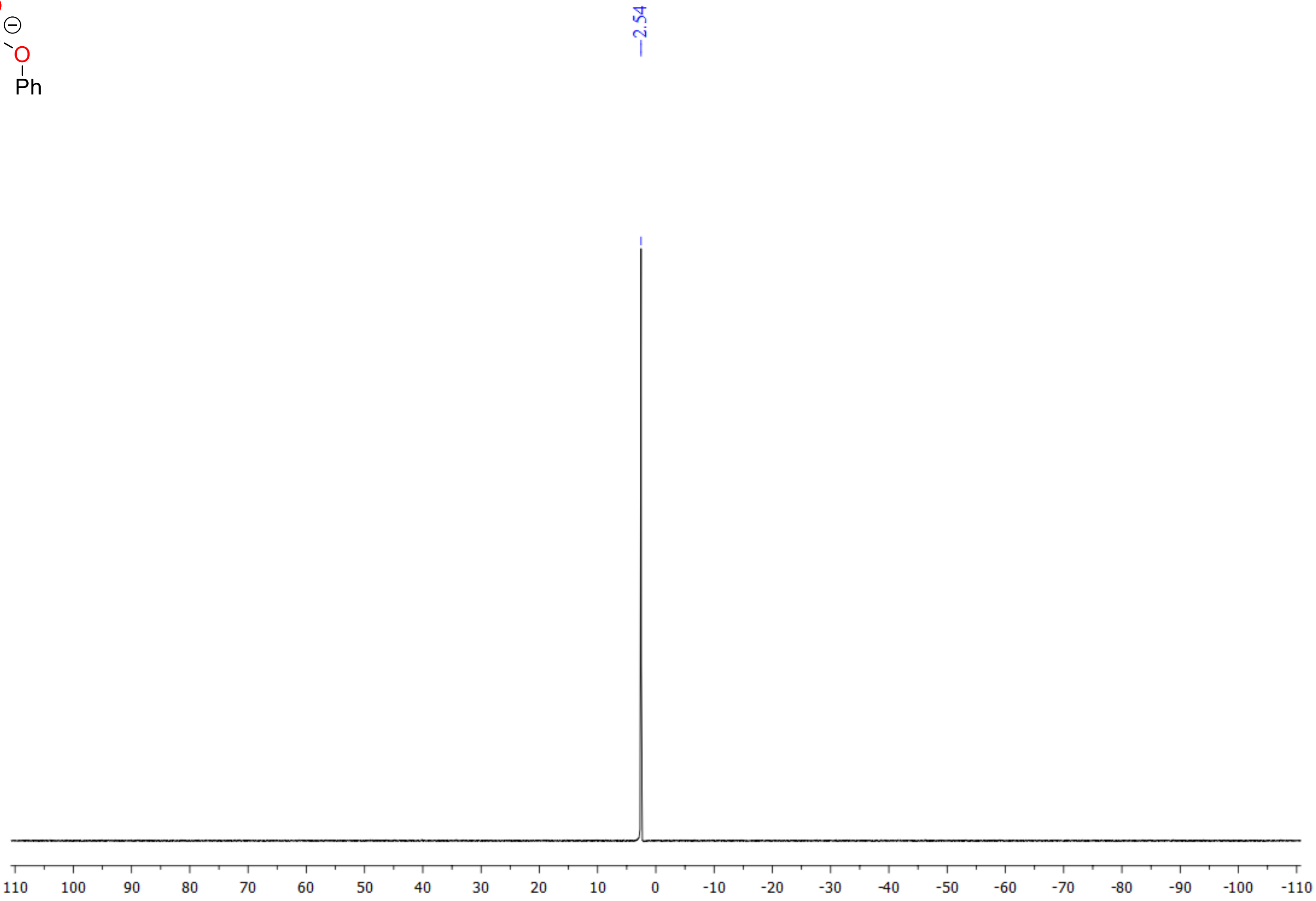

Figure S10.1.26 ^{1}H NMR (400 MHz, $(CD_3)_2SO$, 295 K) spectrum of $Na[B(O^{i}Pr)_4]$ (attempted synthesis).

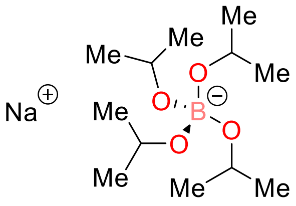

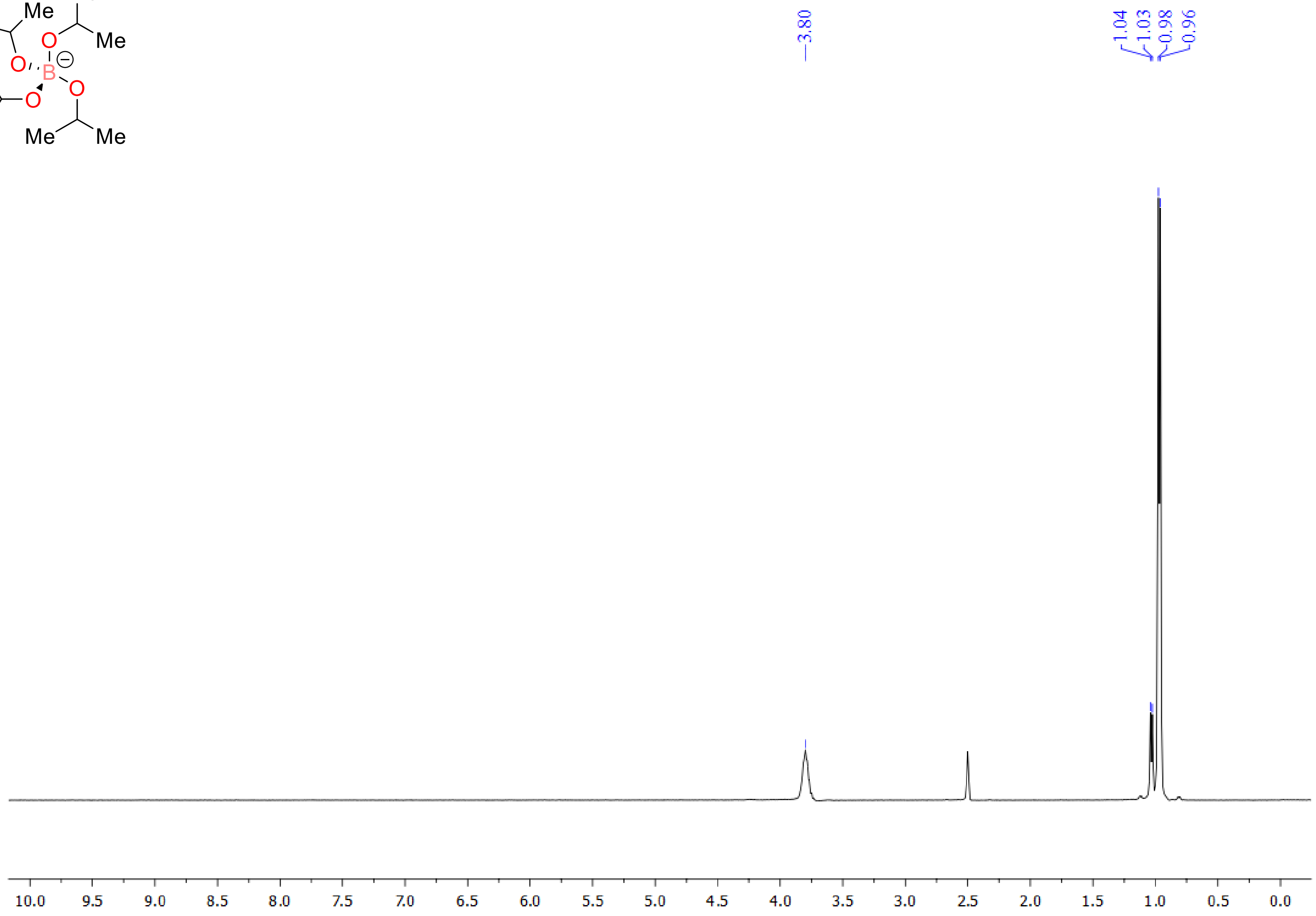

Figure S10.1.27 ^{11}B NMR (128 MHz, $(CD_3)_2SO$, 295 K) spectrum of $Na[B(O^iPr)_4]$ (attempted synthesis).

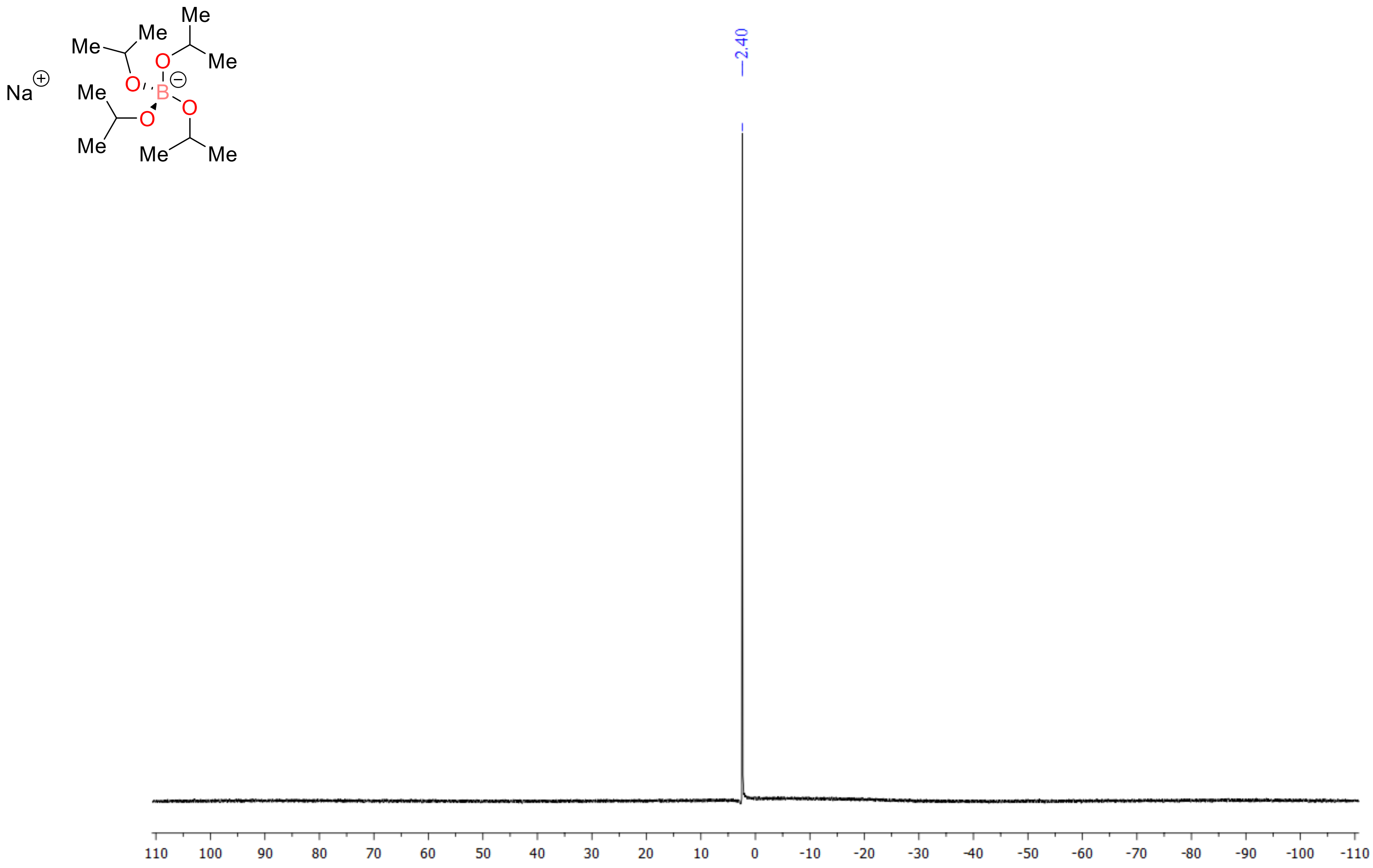

S10.2 NMR spectra of sodium borate complexes exposed to air.

Figure S10.2.1 ^{1}H NMR (400 MHz, CD_3CN, 295 K) spectrum of Na[B(hfip)$_4$]·DME (**1a**) after 24 hours (top) and 48 hours (bottom).

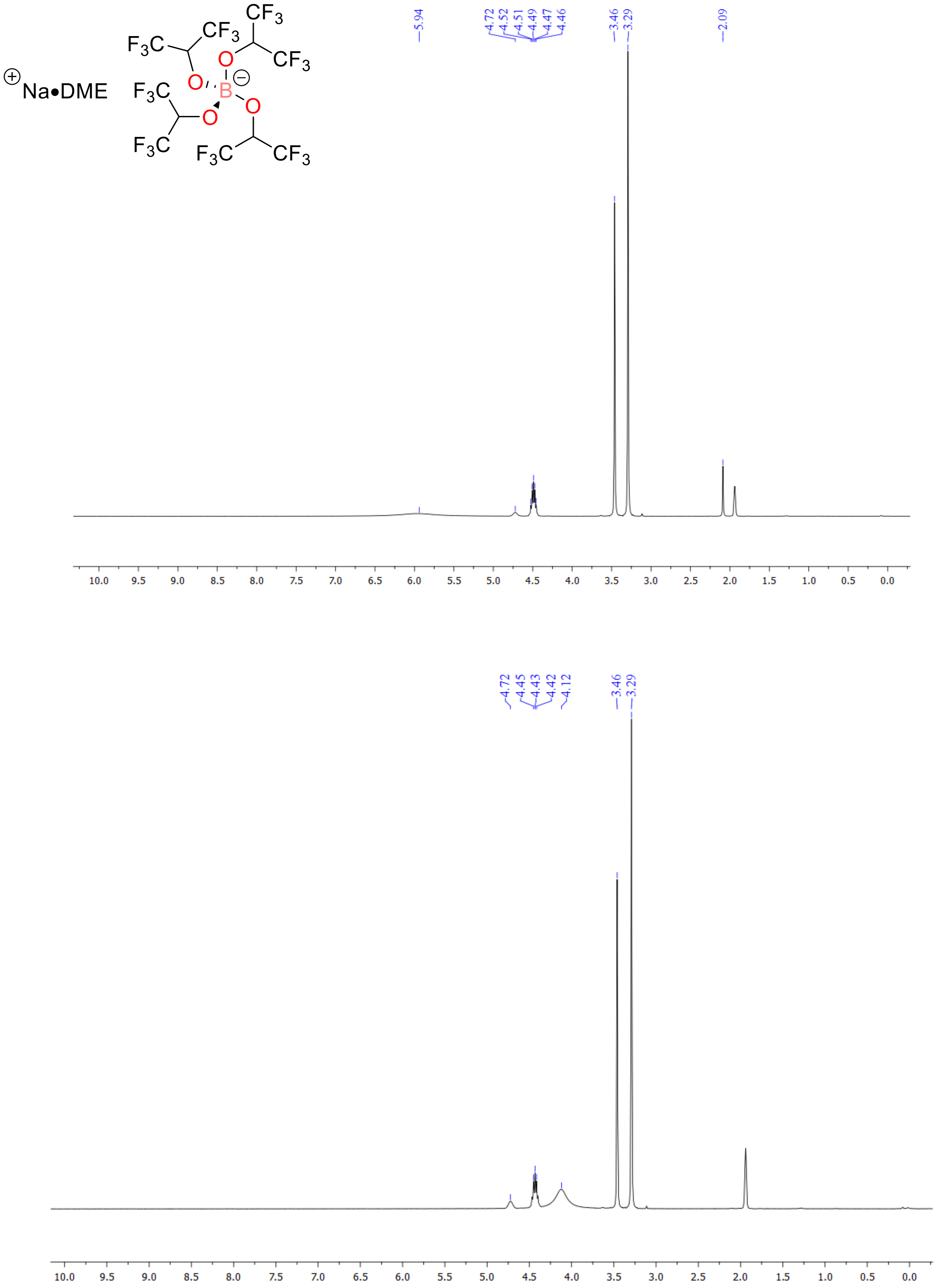

Figure S10.2.2 ^{11}B NMR (128 MHz, CD_3CN, 295 K) spectrum of Na[B(hfip)$_4$]·DME (**1a**) after 24 hours (top) and 48 hours (bottom).

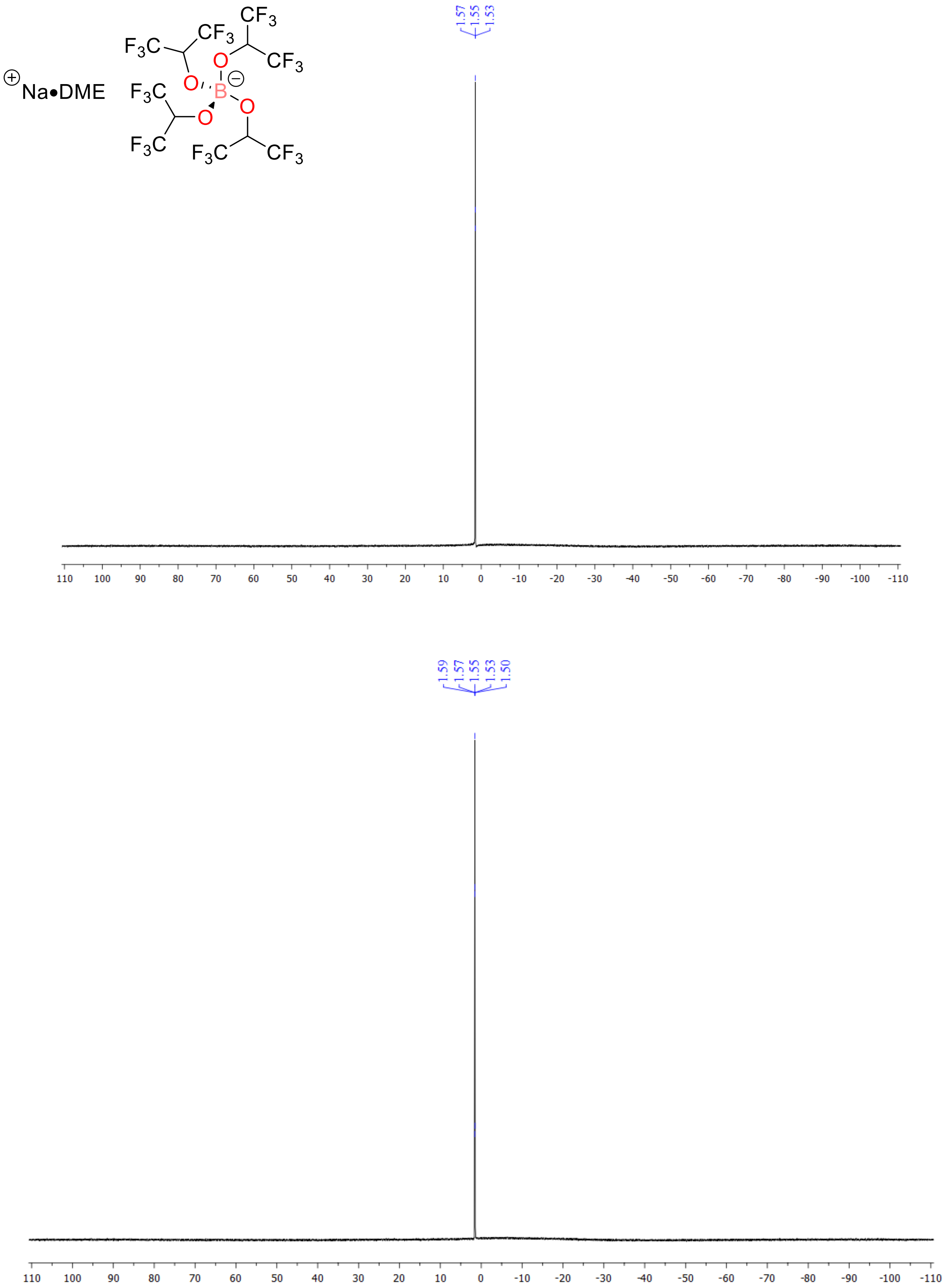

Figure S10.2.3 ^{19}F NMR (376 MHz, CD_3CN, 295 K) spectrum of Na[B(hfip)$_4$]·DME (**1a**) after 24 hours (top) and 48 hours (bottom).

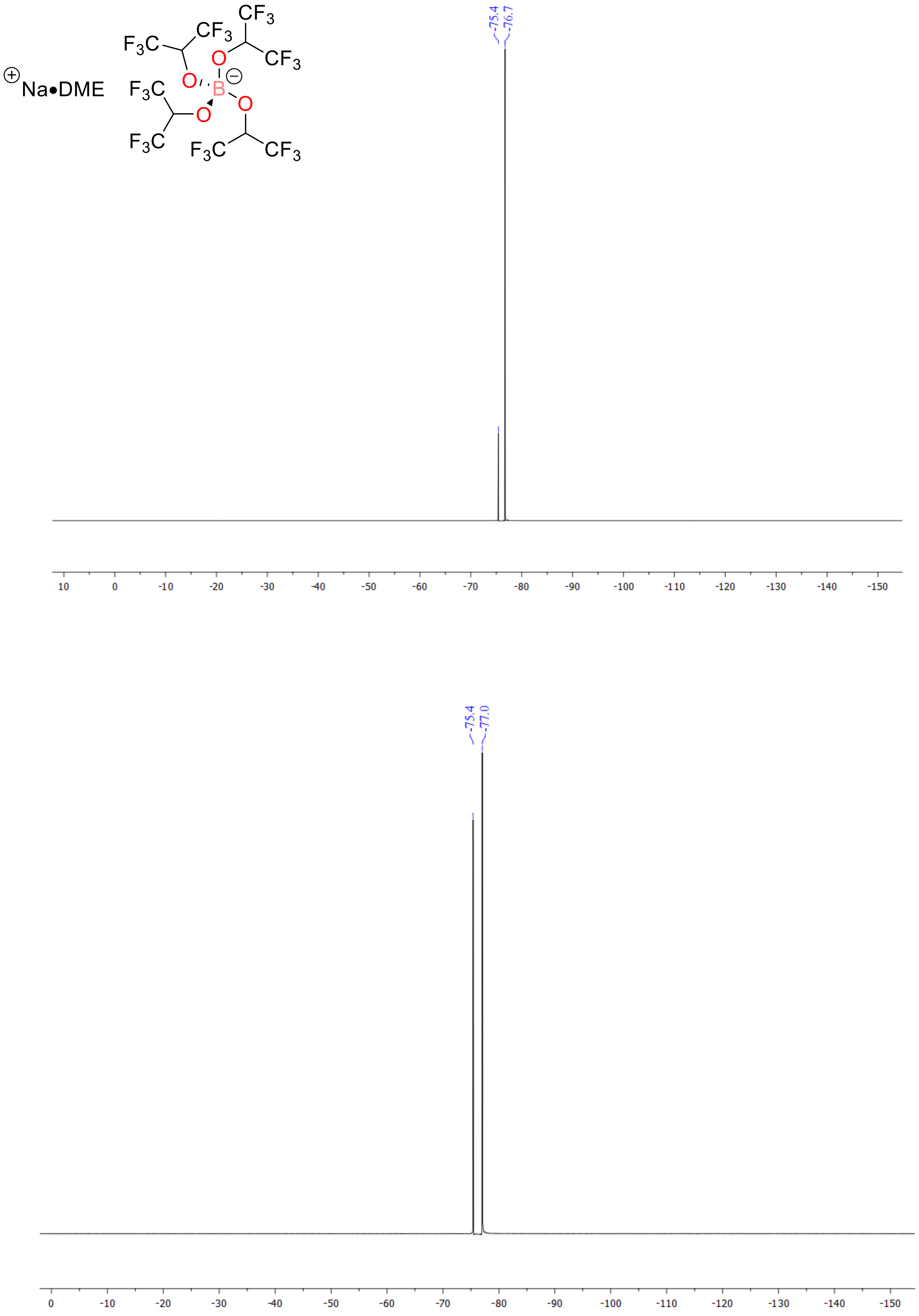

Figure S10.2.4 ^{1}H NMR (400 MHz, CD_3CN, 295 K) spectrum of $Na[B(pp)_2]\cdot 3DME$ (**1b**) after 24 hours (top) and 48 hours (bottom).

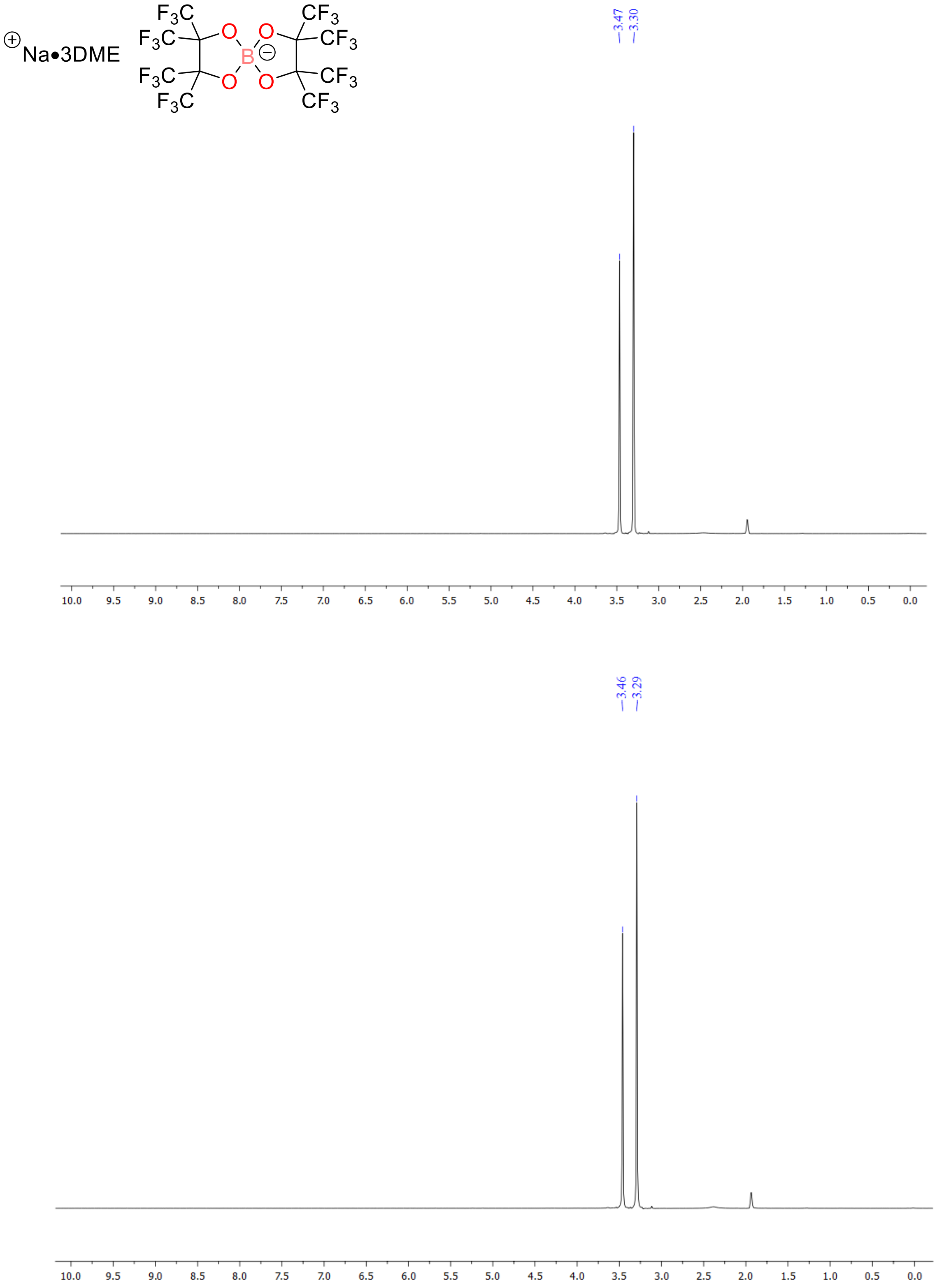

Figure S10.2.5 ^{11}B NMR (128 MHz, CD_3CN, 295 K) spectrum of Na[B(pp)$_2$]·3DME (**1b**) after 24 hours (top) and 48 hours (bottom).

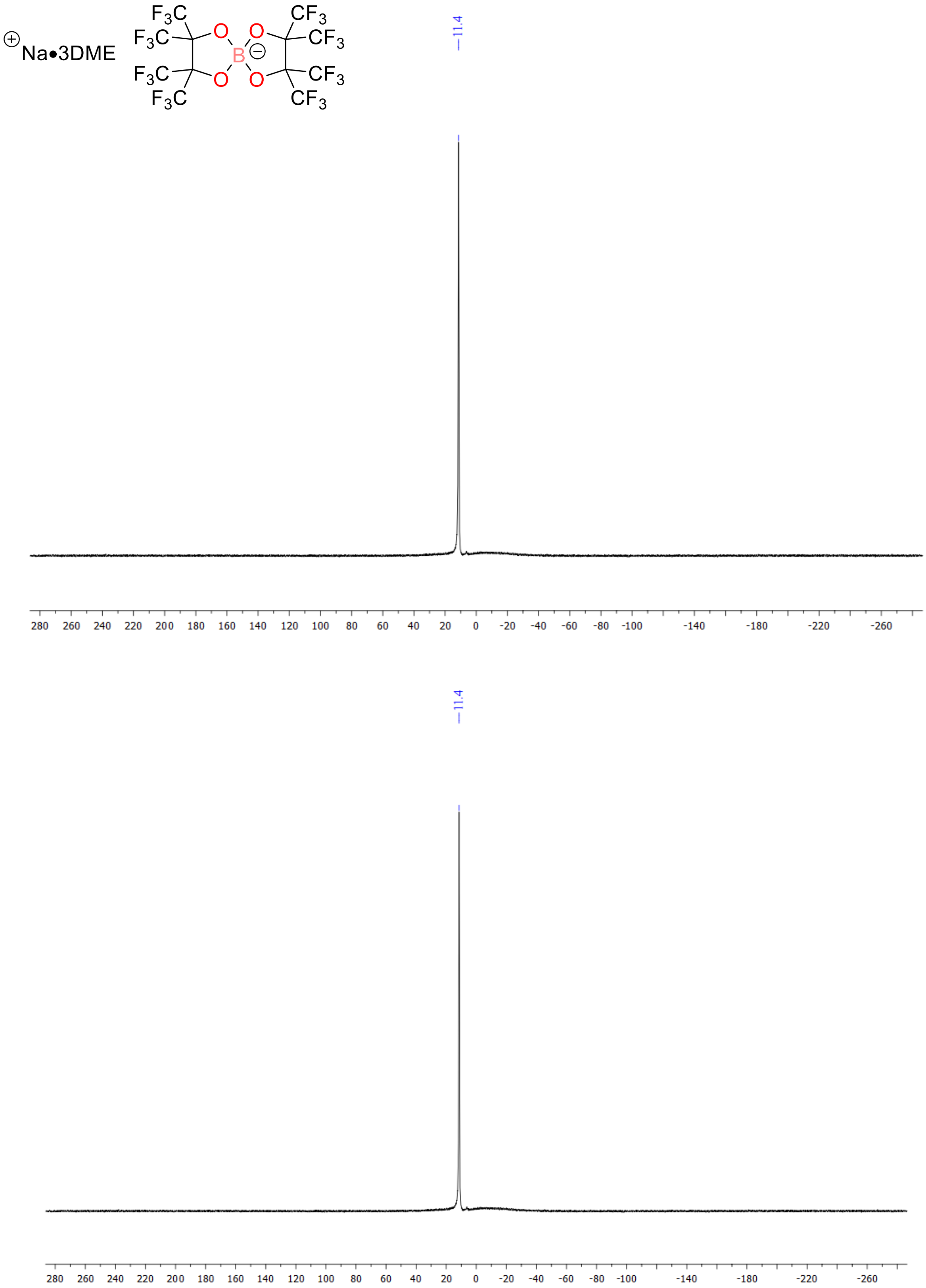

Figure S10.2.6 ^{19}F NMR (376 MHz, CD_3CN, 295 K) spectrum of Na[B(pp)$_2$]·3DME (**1b**) after 24 hours (top) and 48 hours (bottom).

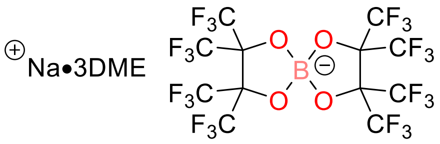

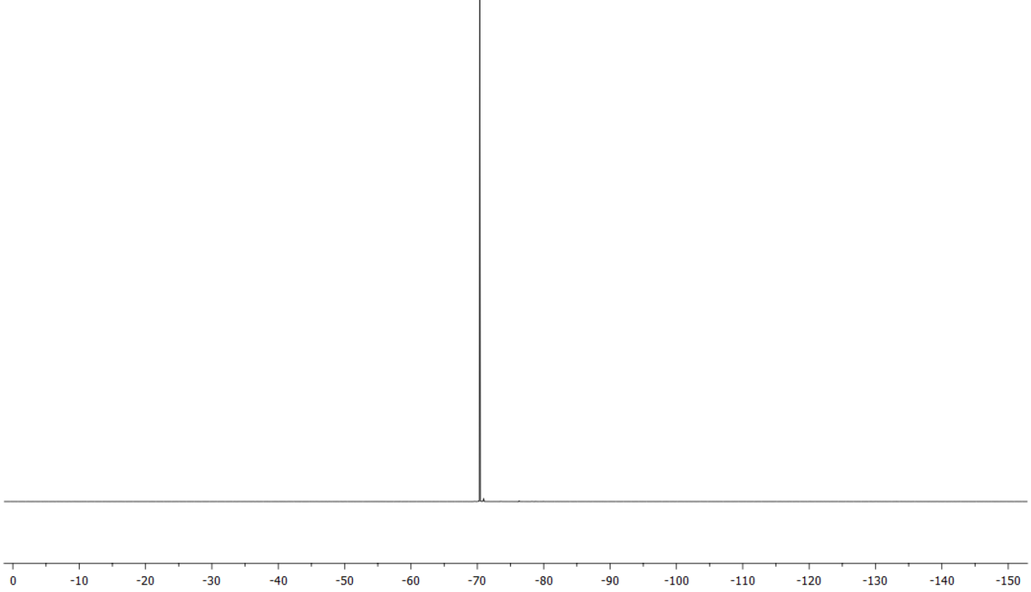

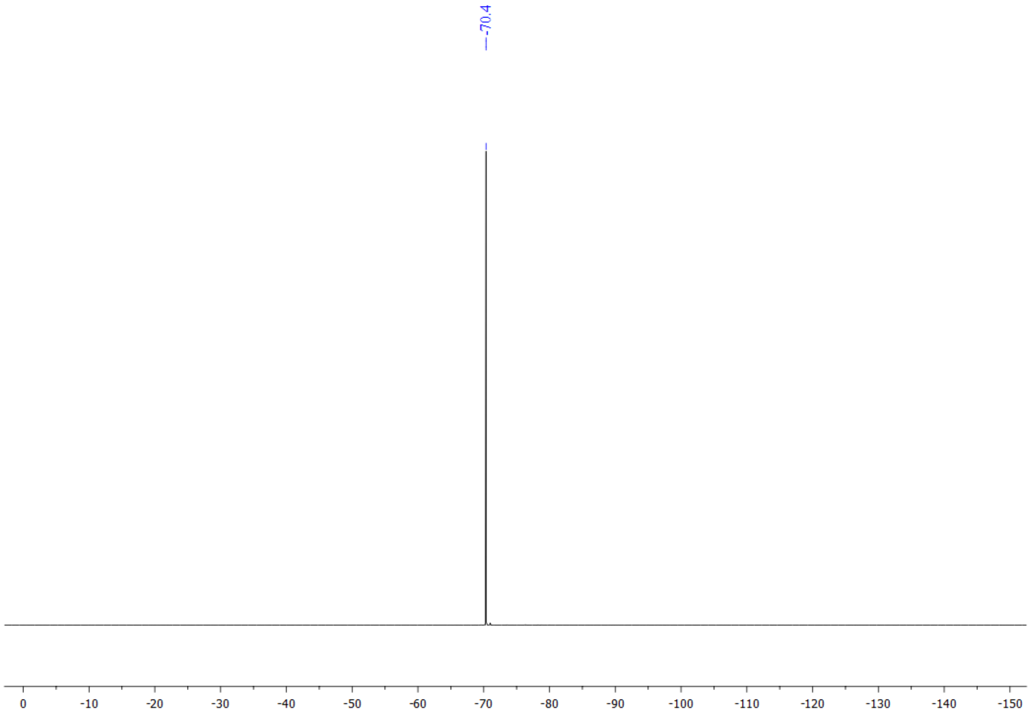

Figure S10.2.7 ^{1}H NMR (400 MHz, CD_3CN, 295 K) spectrum of $Na[B(pp)_2]$ (**1b'**) after 24 hours (top) and 48 hours (bottom).

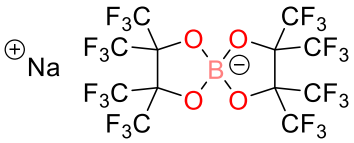

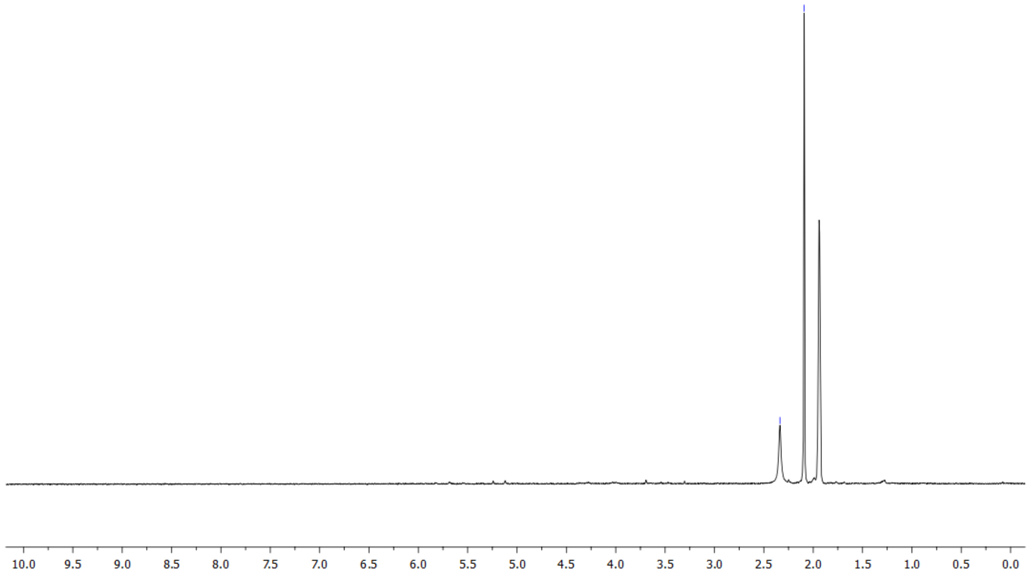

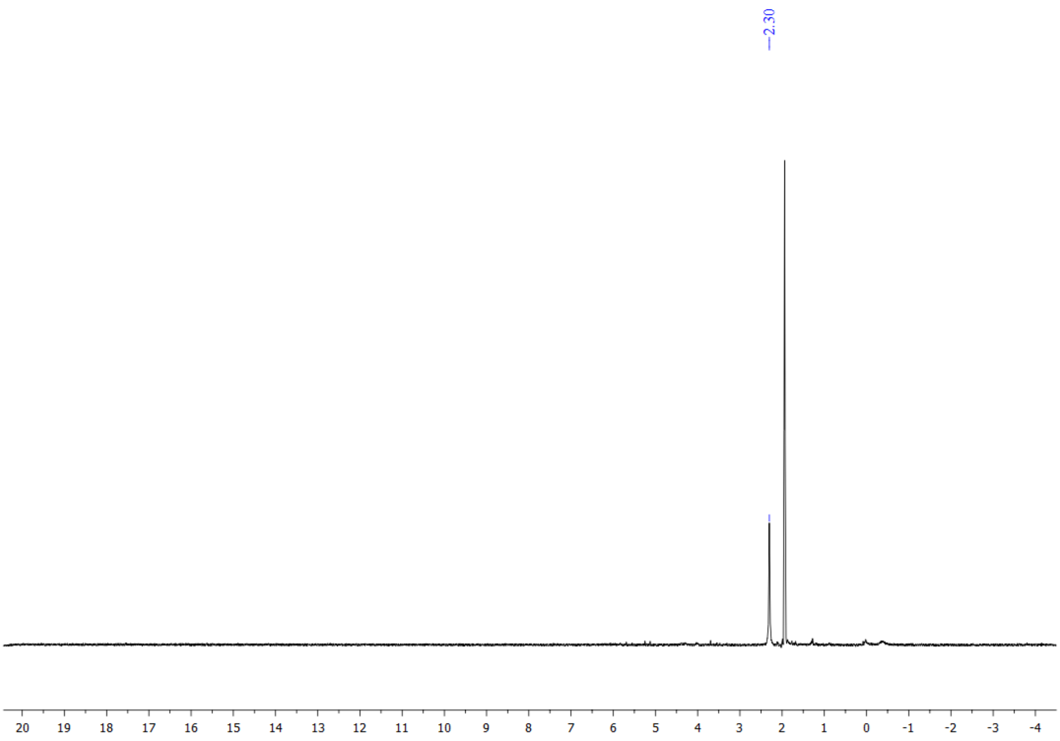

Figure S10.2.8 ^{11}B NMR (128 MHz, CD_3CN, 295 K) spectrum of Na[B(pp)$_2$] (**1b’**) after 24 hours (top) and 48 hours (bottom).

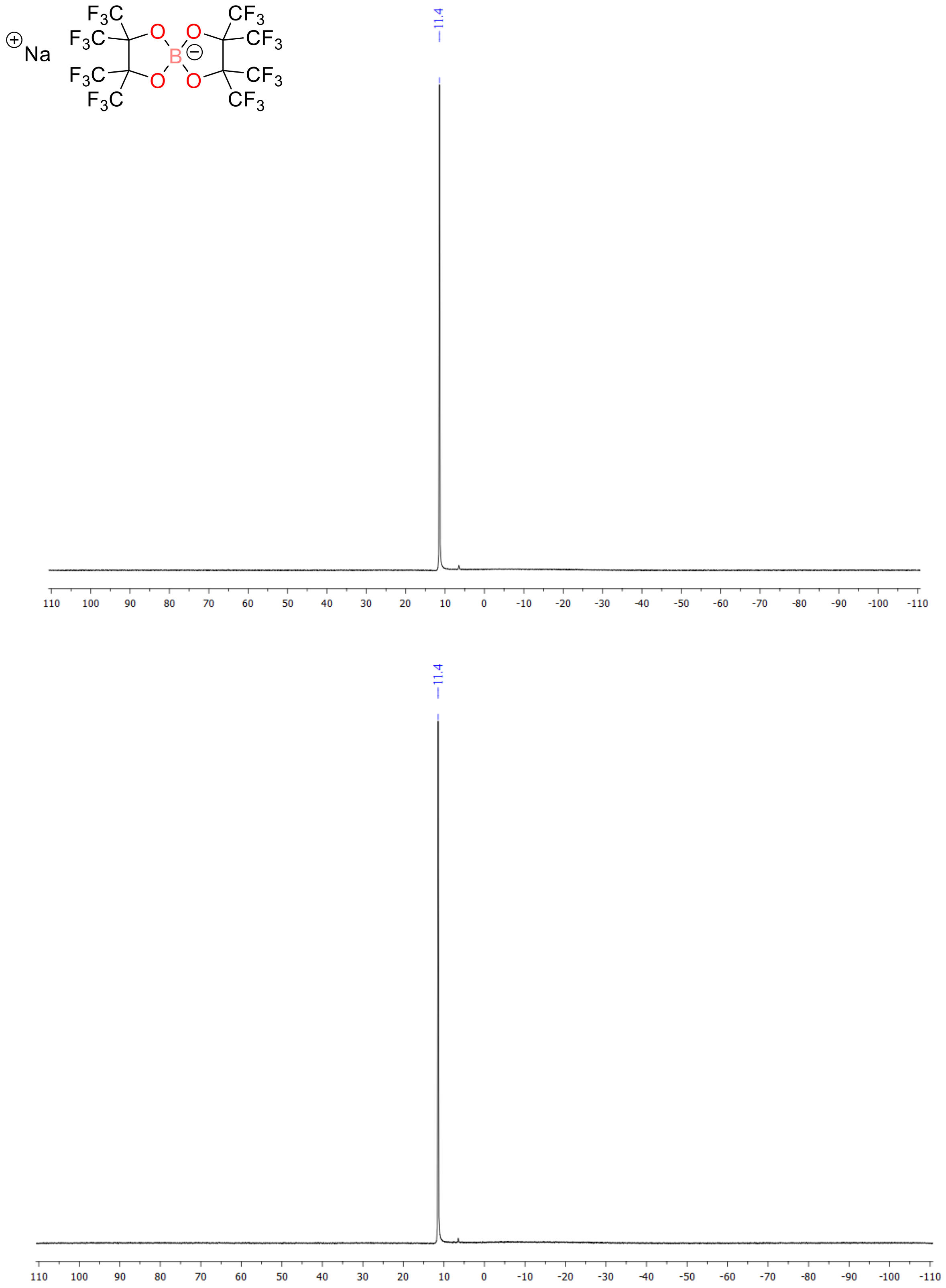

Figure S10.2.9 ^{19}F NMR (376 MHz, CD_3CN, 295 K) spectrum of Na[B(pp)$_2$] (**1b'**) after 24 hours (top) and 48 hours (bottom)

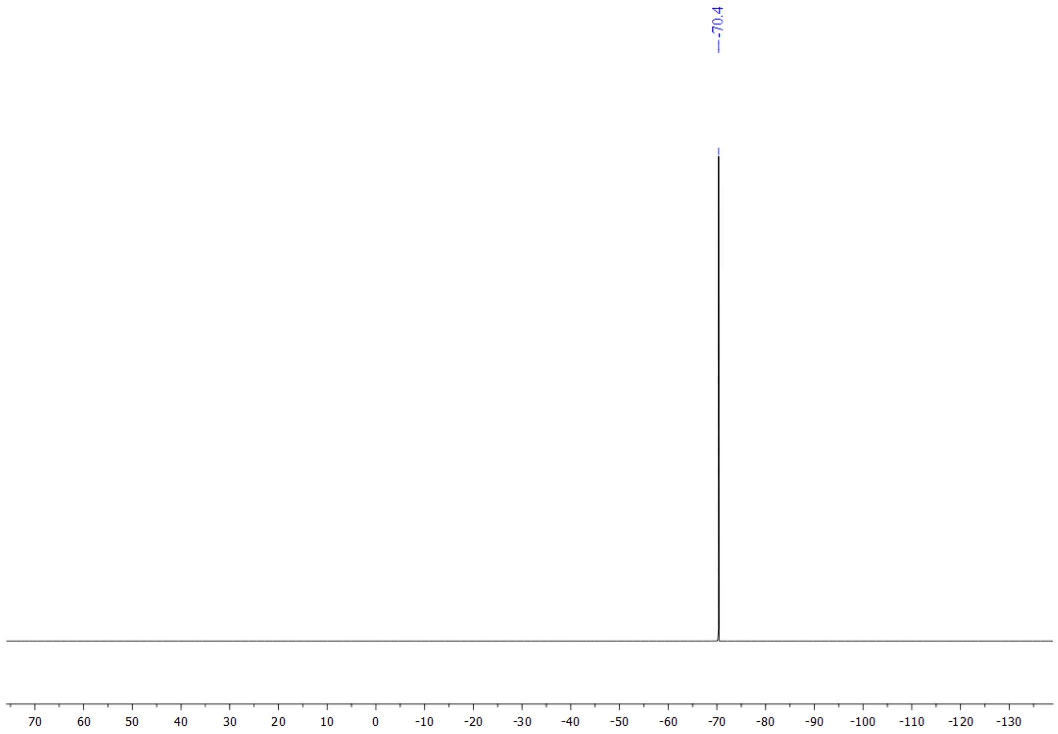

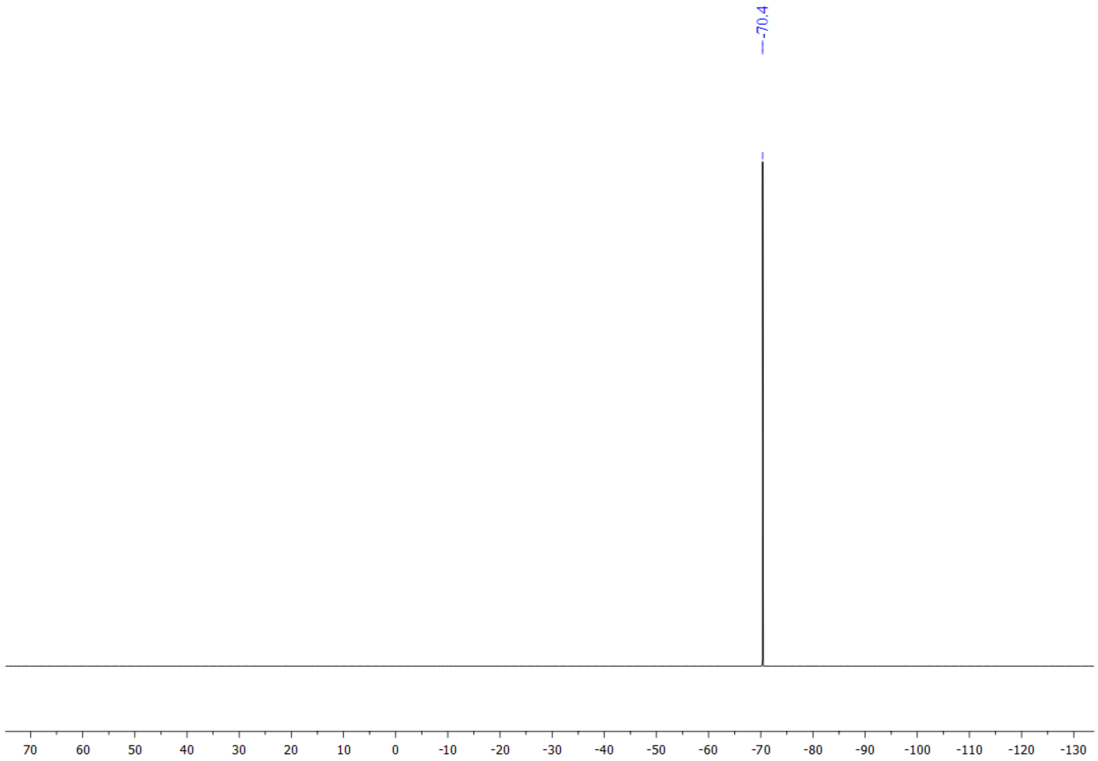

Figure S10.2.10 ^{1}H NMR (400 MHz, CD_3CN, 295 K) spectrum of $Na[B(OCH_2(CF_2)_2CF_3)_4]$ (**1c**) after 24 hours (top) and 48 hours (bottom).

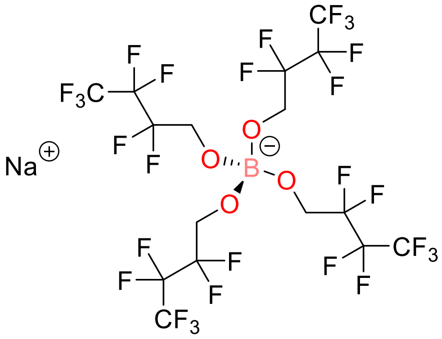

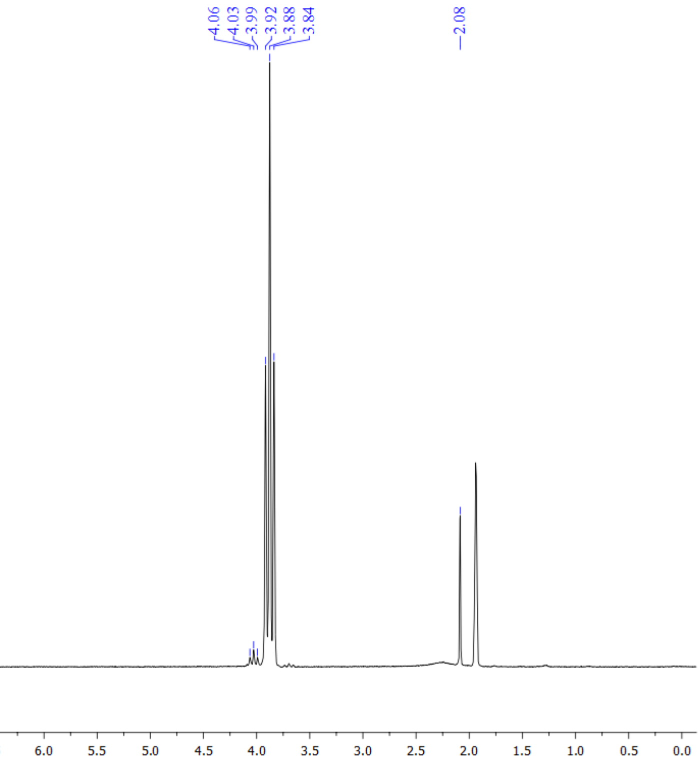

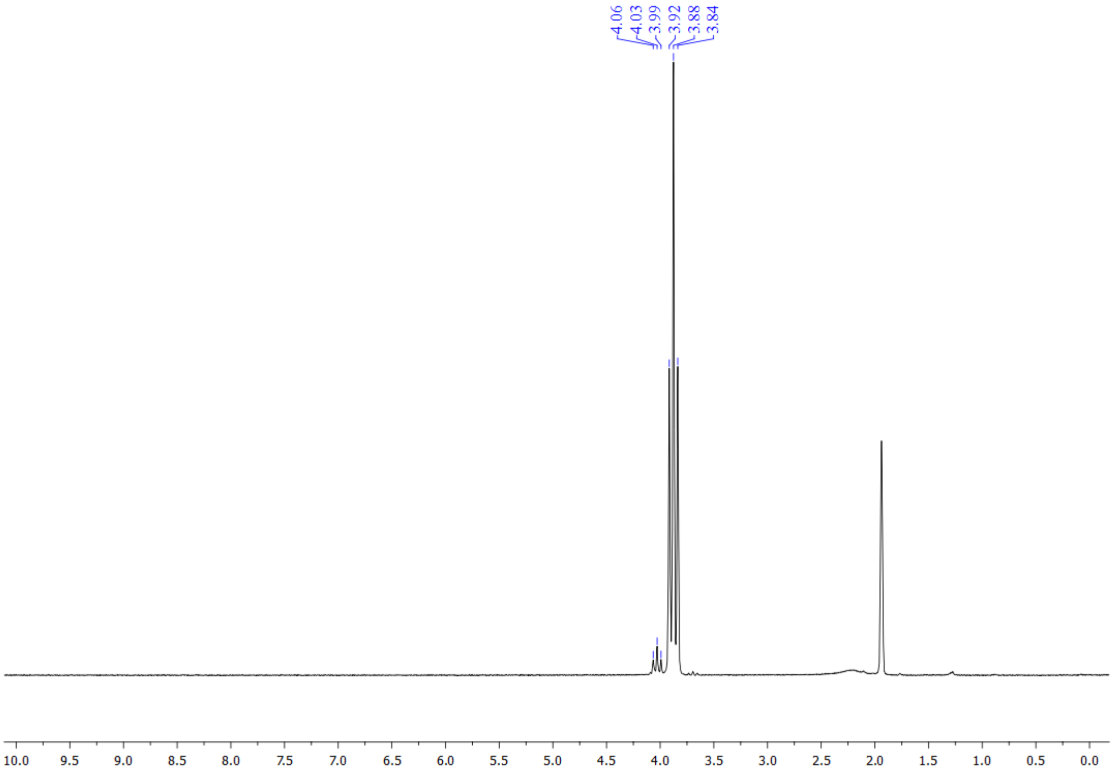

Figure S10.2.11 ^{11}B NMR (128 MHz, CD_3CN, 295 K) spectrum of $Na[B(OCH_2(CF_2)_2CF_3)_4]$ (**1c**) after 24 hours (top) and 48 hours (bottom).

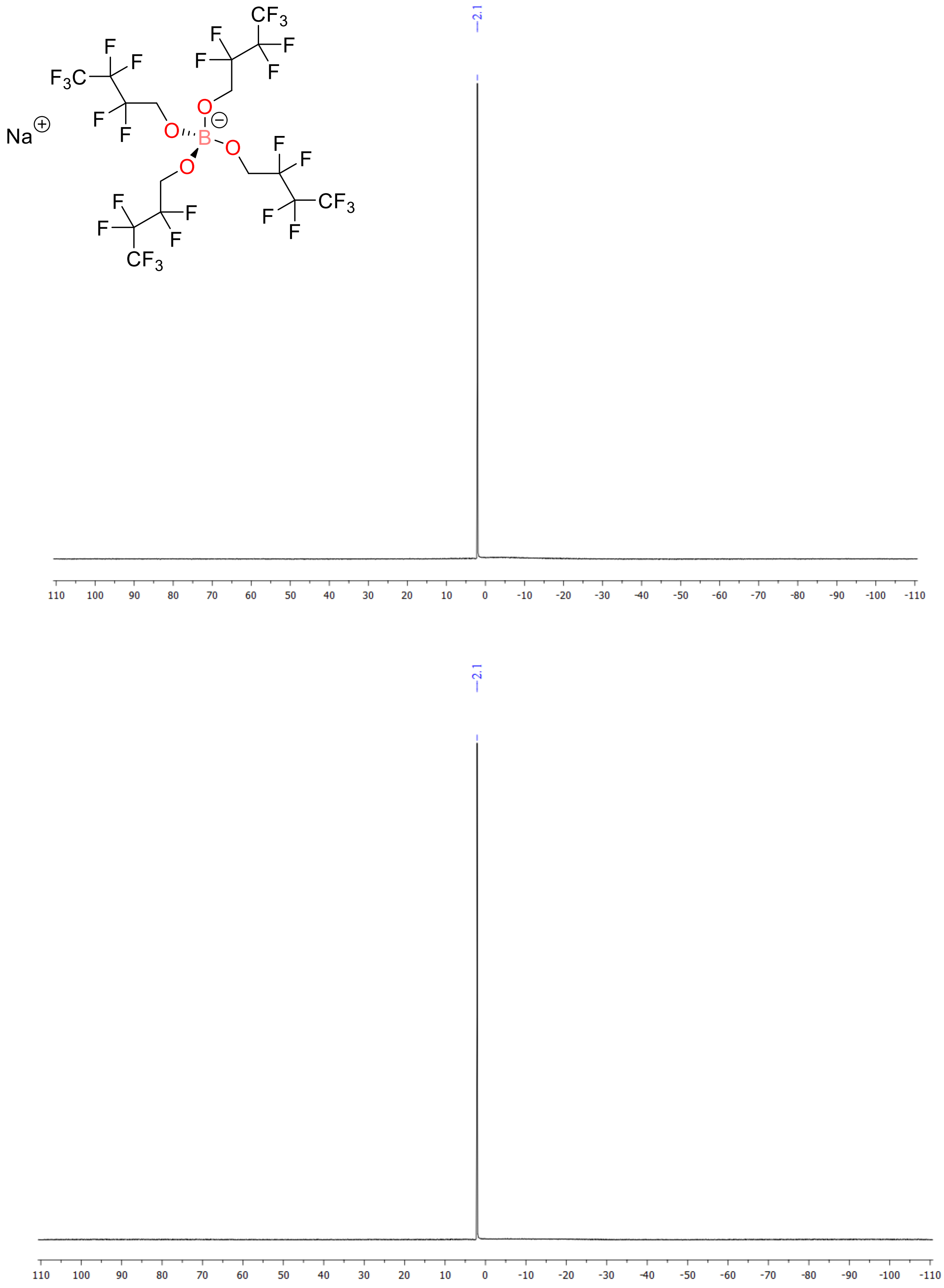

Figure S10.2.12 ^{19}F NMR (376 MHz, CD_3CN, 295 K) spectrum of $Na[B(OCH_2(CF_2)_2CF_3)_4]$ (**1c**) after 24 hours (top) and 48 hours (bottom).

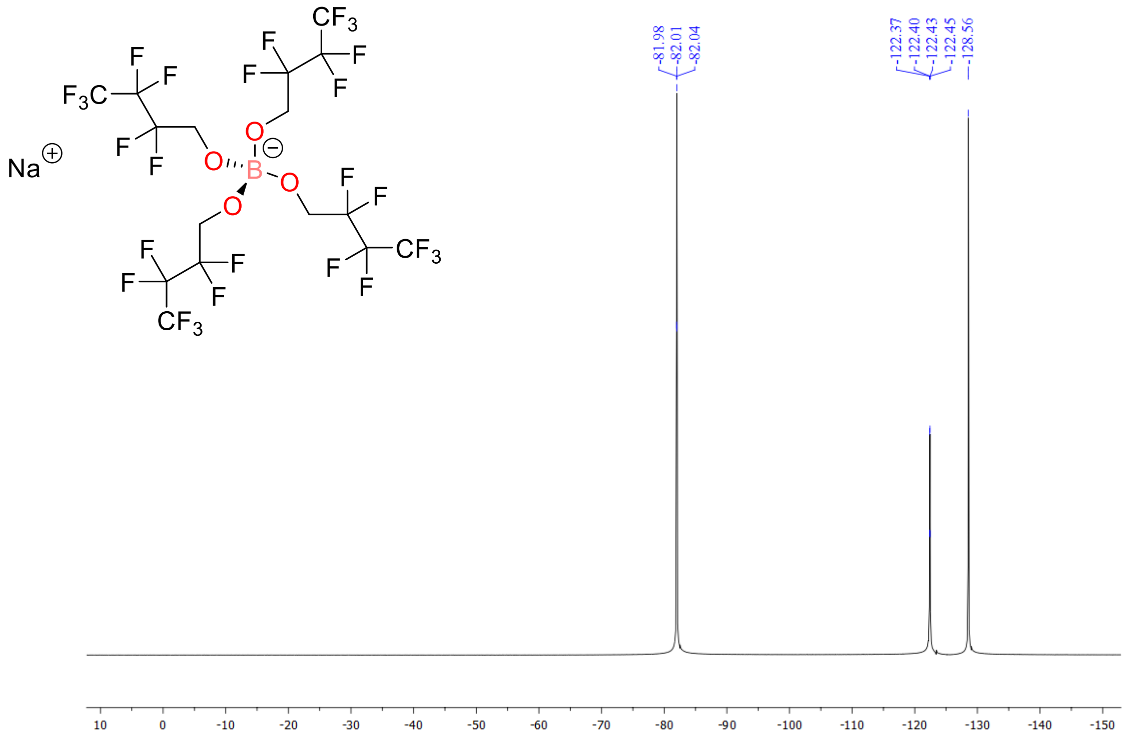

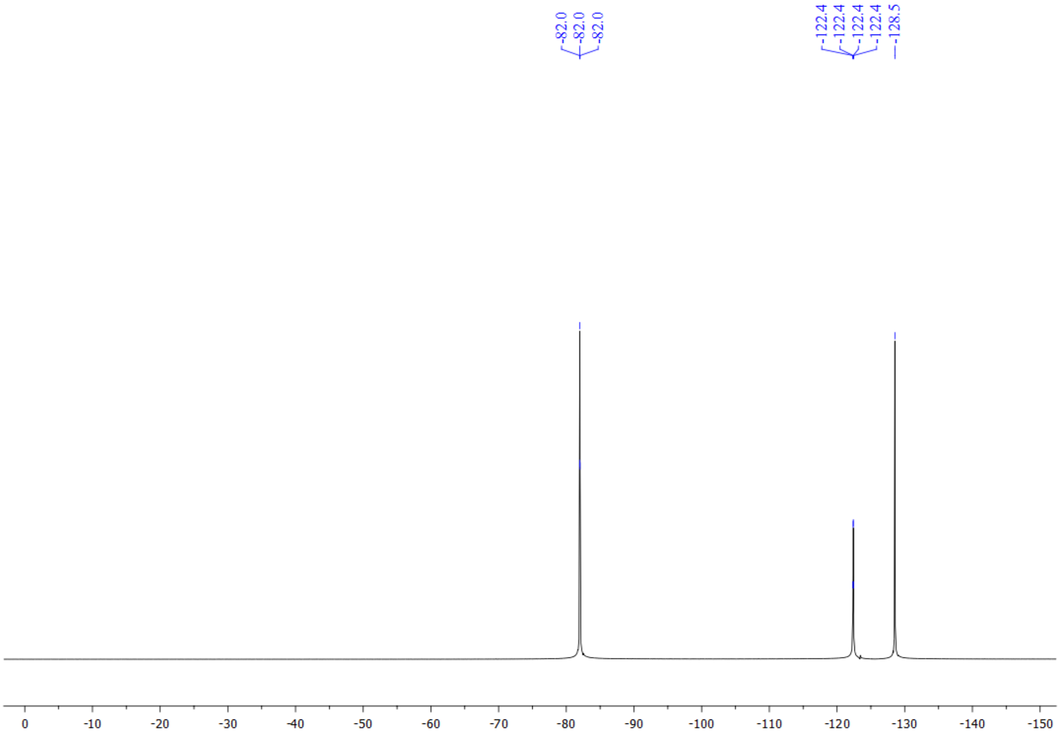

Figure S10.2.13 ^{1}H NMR (400 MHz, CD_3CN, 295 K) spectrum of $Na[B(OPh^F)_4]\cdot 3DME$ (**1d**) after 24 hours (top) and 48 hours (bottom).

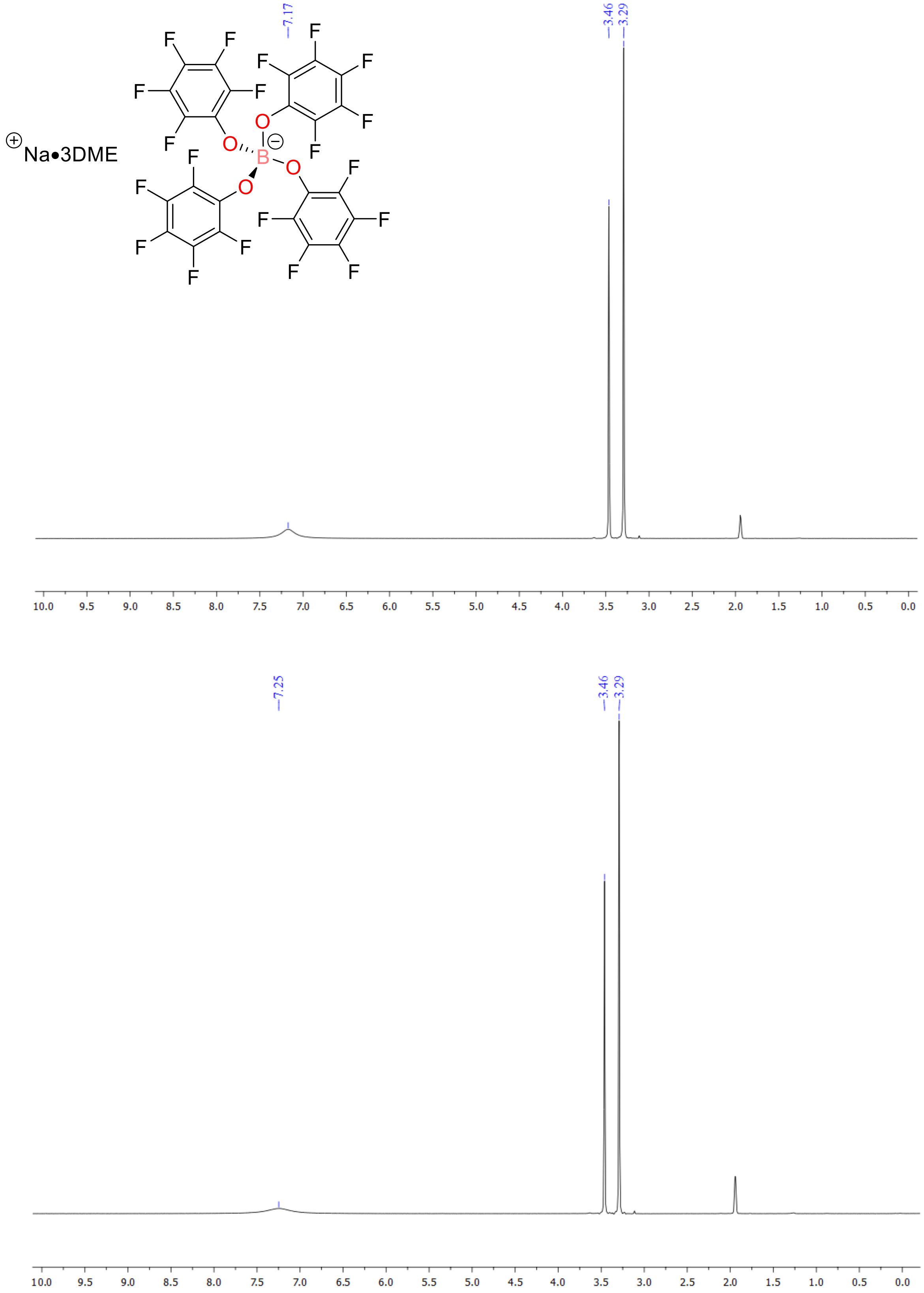

Figure S10.2.14 ^{11}B NMR (128 MHz, $(CD_3)_2SO$, 295 K) spectrum of Na[B(OPh^F)$_4$]·3DME (**1d**) after 24 hours (top) and 48 hours (bottom).

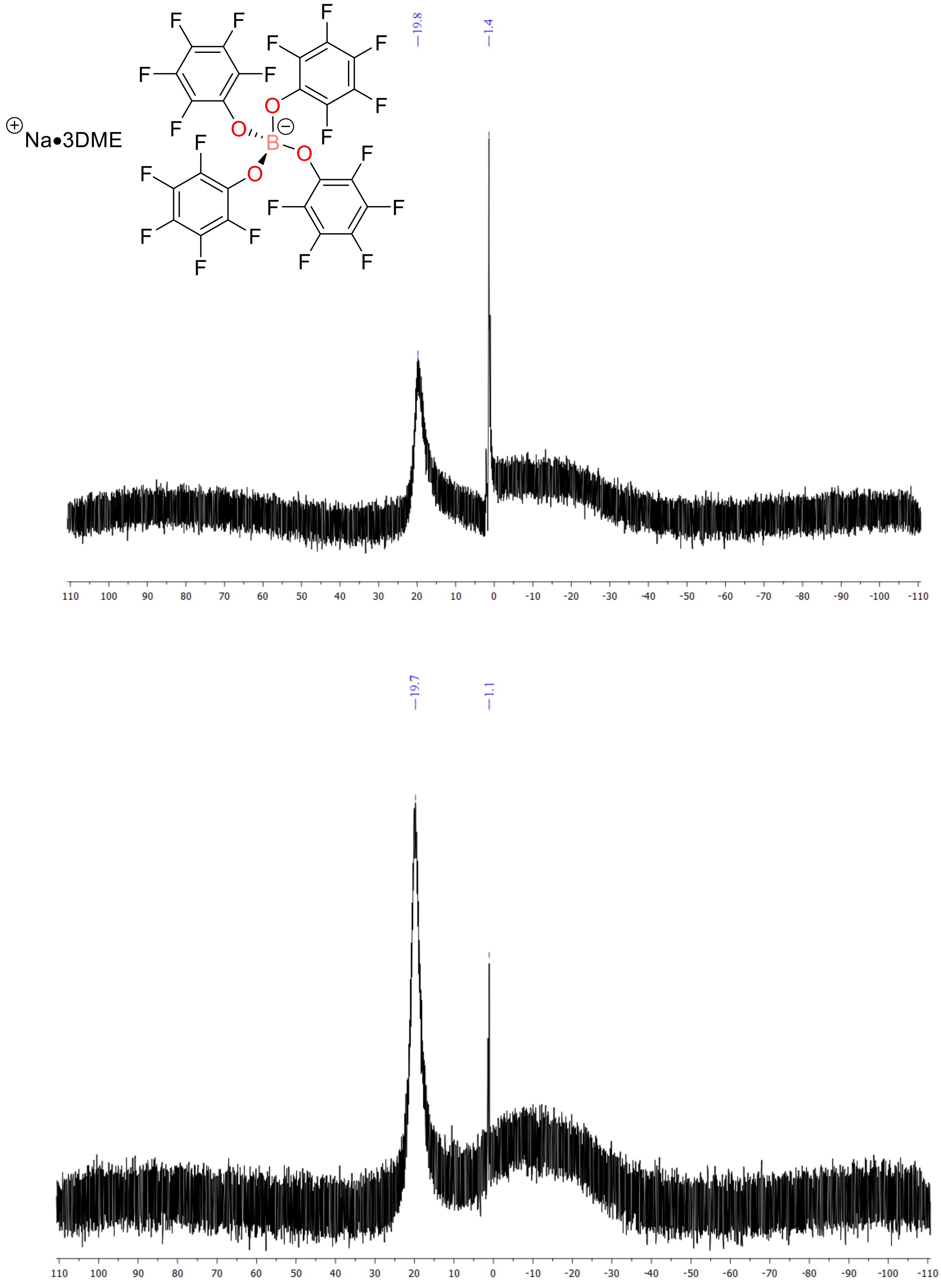

Figure S10.2.15 ^{19}F NMR (376 MHz, $(CD_3)_2SO$, 295 K) spectrum of $Na[B(OPh^F)_4]\cdot 3DME$ (**1d**) after 24 hours (top) and 48 hours (bottom).

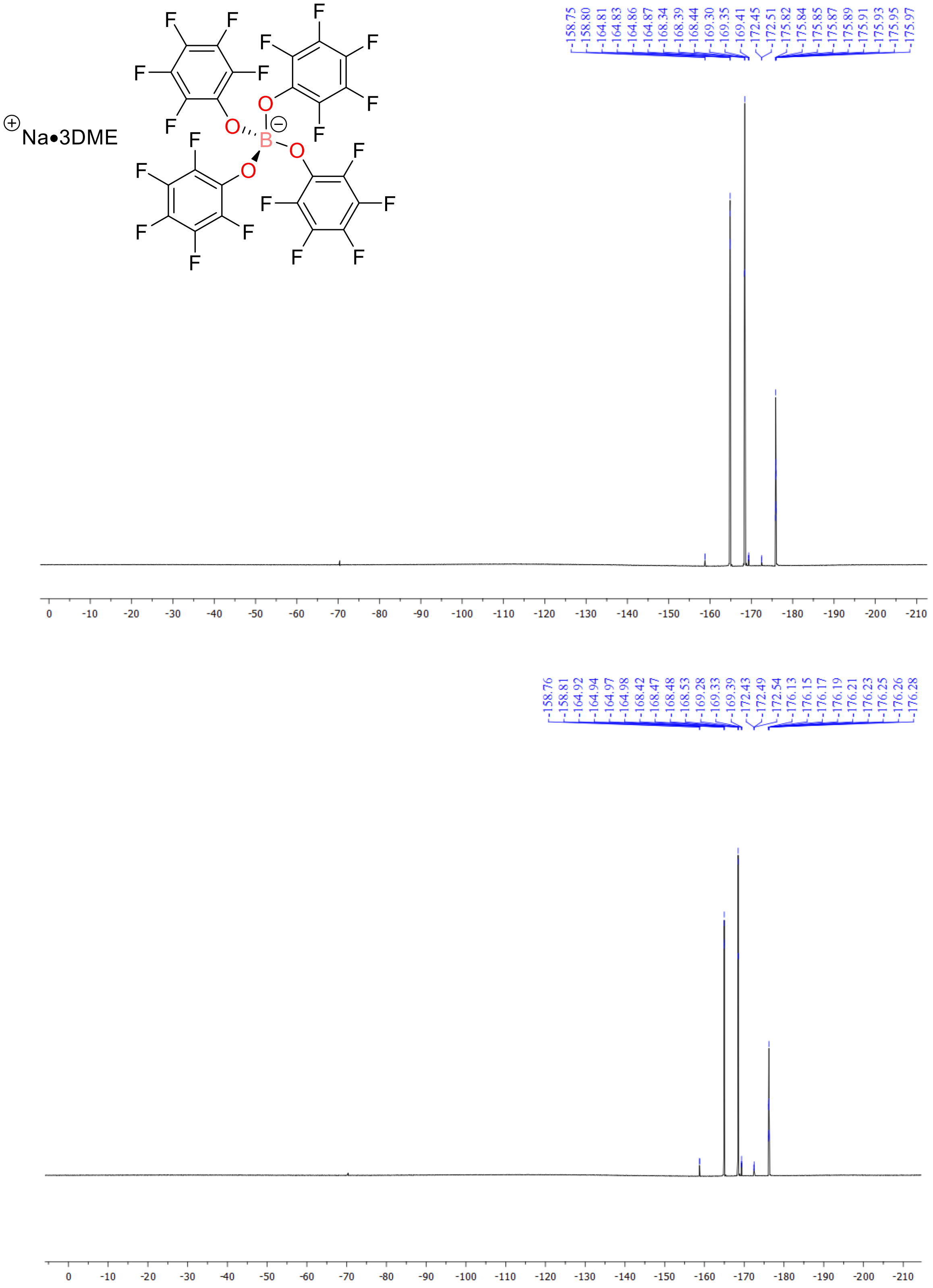

Figure S10.2.16 ^{1}H NMR (400 MHz, CD_3CN, 295 K) spectrum of Na[B(OMe)$_4$] (**1e**) after 24 hours (top) and 48 hours (bottom).

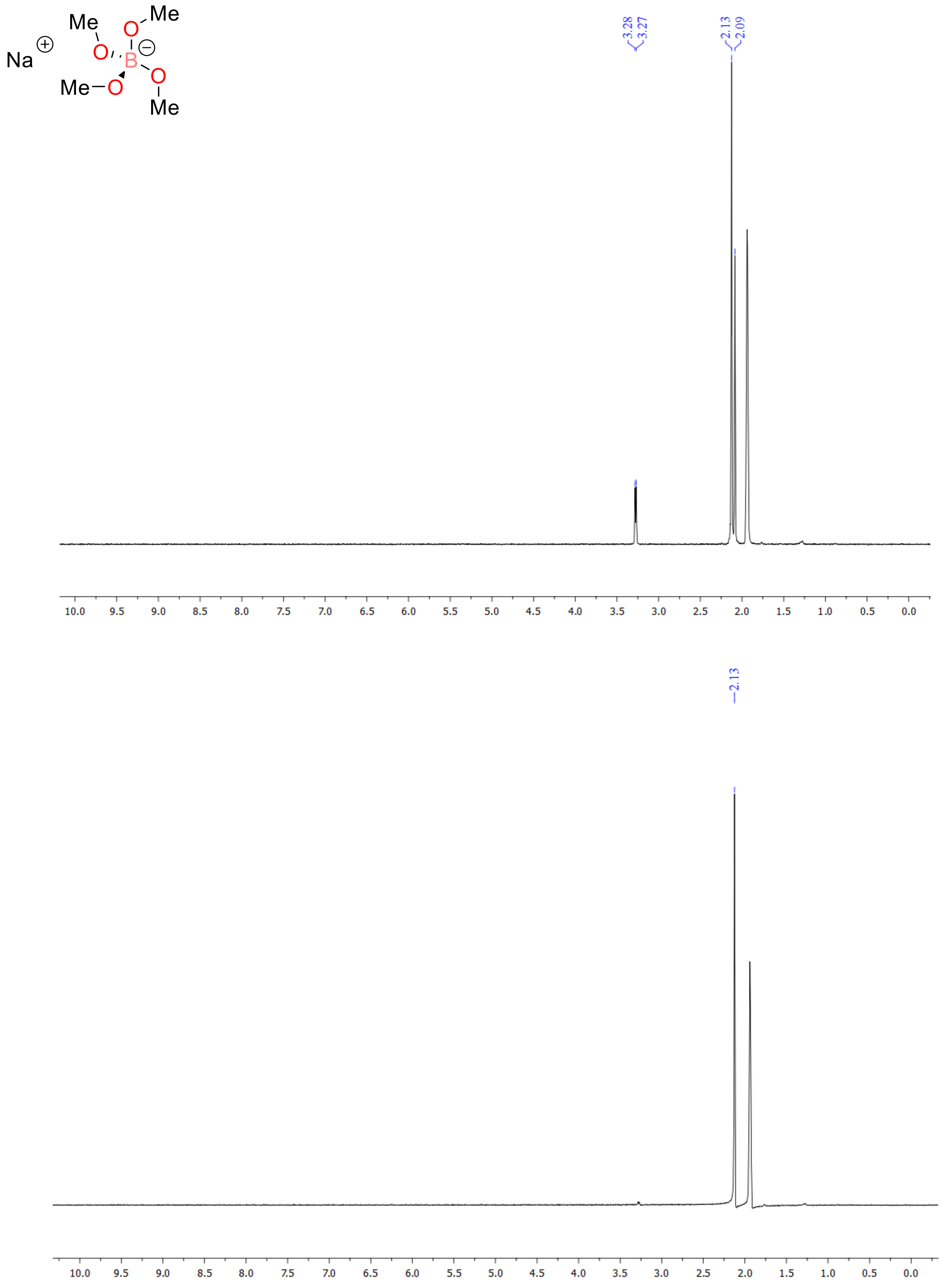

Figure S10.2.17 ^{1}H NMR (400 MHz, CD_3CN, 295 K) spectrum of $Na[B(OPh)_4]$ (**1f**) after 24 hours (top) and 48 hours (bottom).

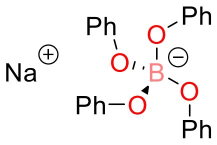

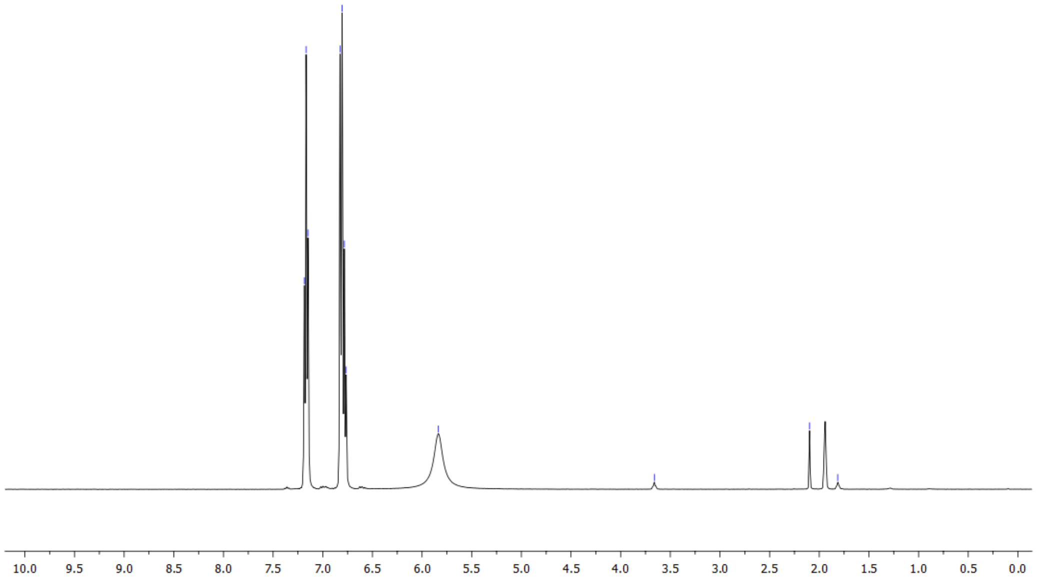

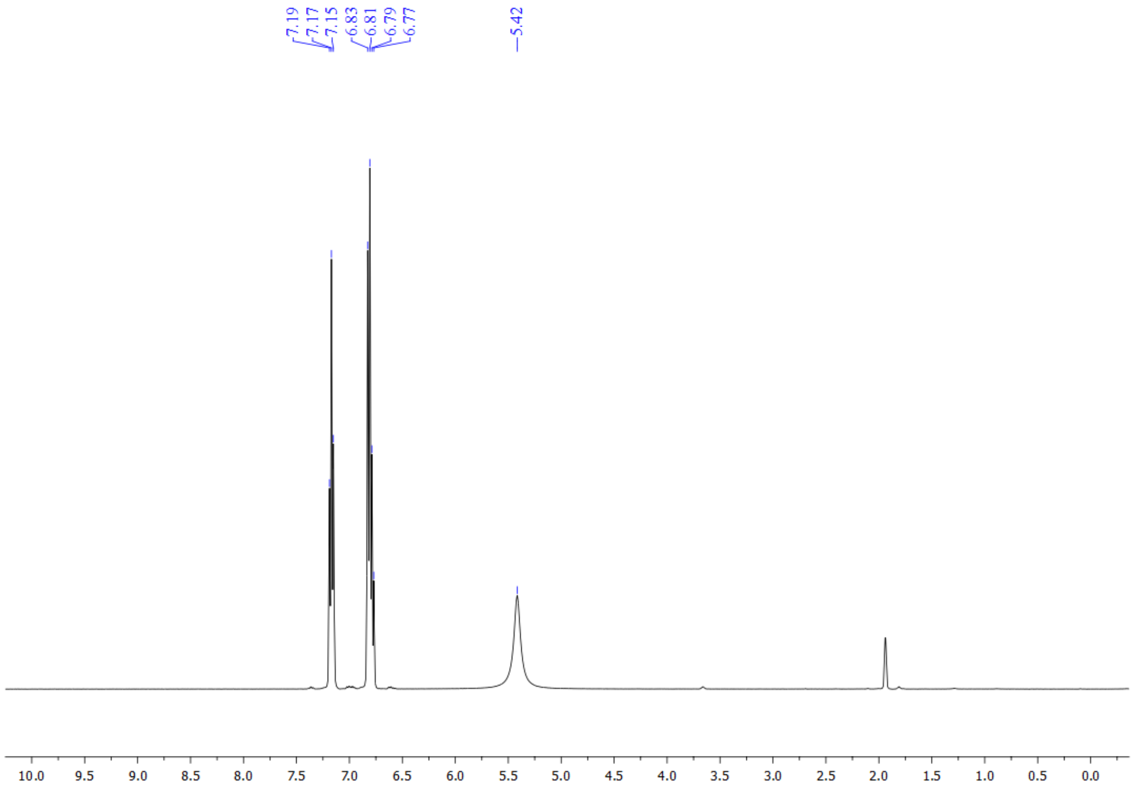

Figure S10.2.18 ^{11}B NMR (128 MHz, $(CD_3)_2SO$, 295 K) spectrum of $Na[B(OPh)_4]$ (**1f**) after 24 hours (top) and 48 hours (bottom).

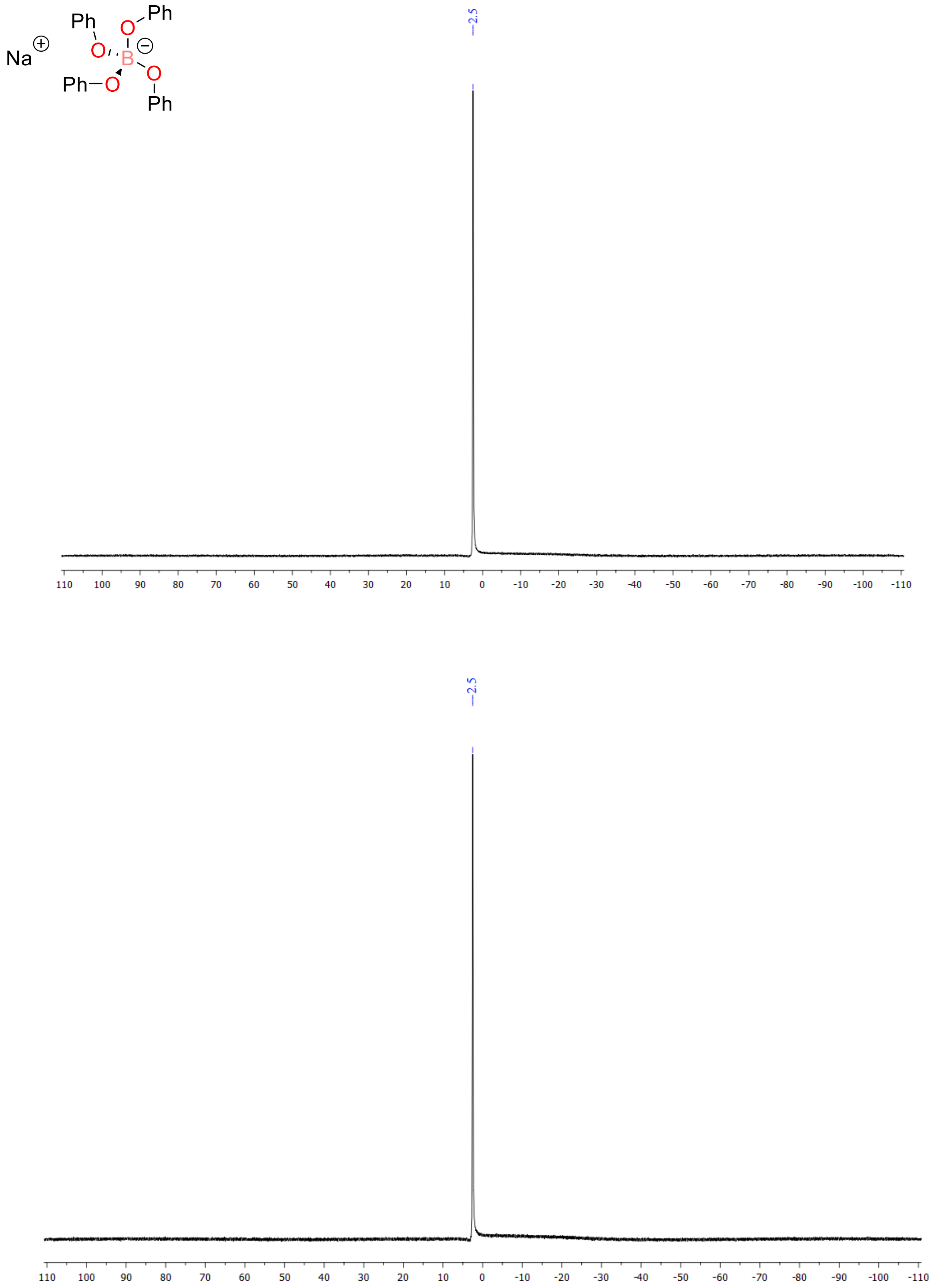

S10.3 NMR spectra of sodium borate complexes exposed to water.

Figure S10.3.1 ^{1}H NMR (400 MHz, CD_3CN, 295 K) spectrum $Na[B(hfip)_4]\cdot DME$ (**1a**) after 10 equiv of water added.

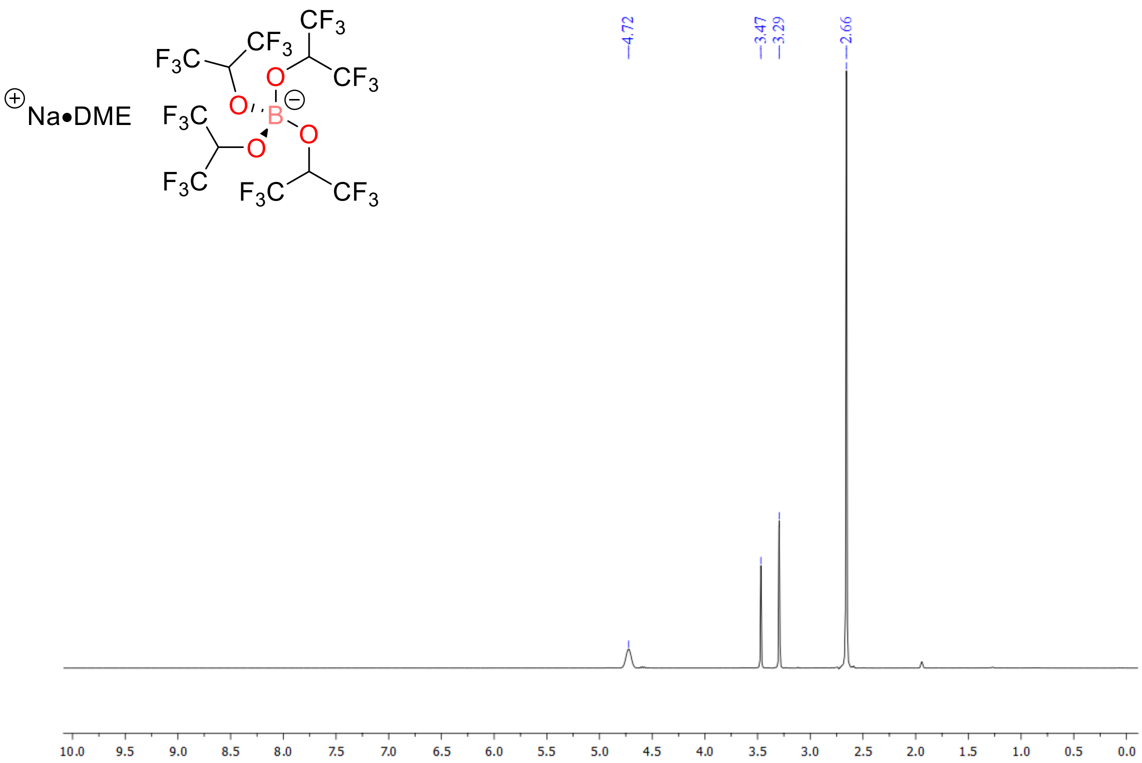

Figure S10.3.2 ^{11}B NMR (128 MHz, CD_3CN, 295 K) spectrum $Na[B(hfip)_4]\cdot DME$ (**1a**) after 10 equiv of water added.

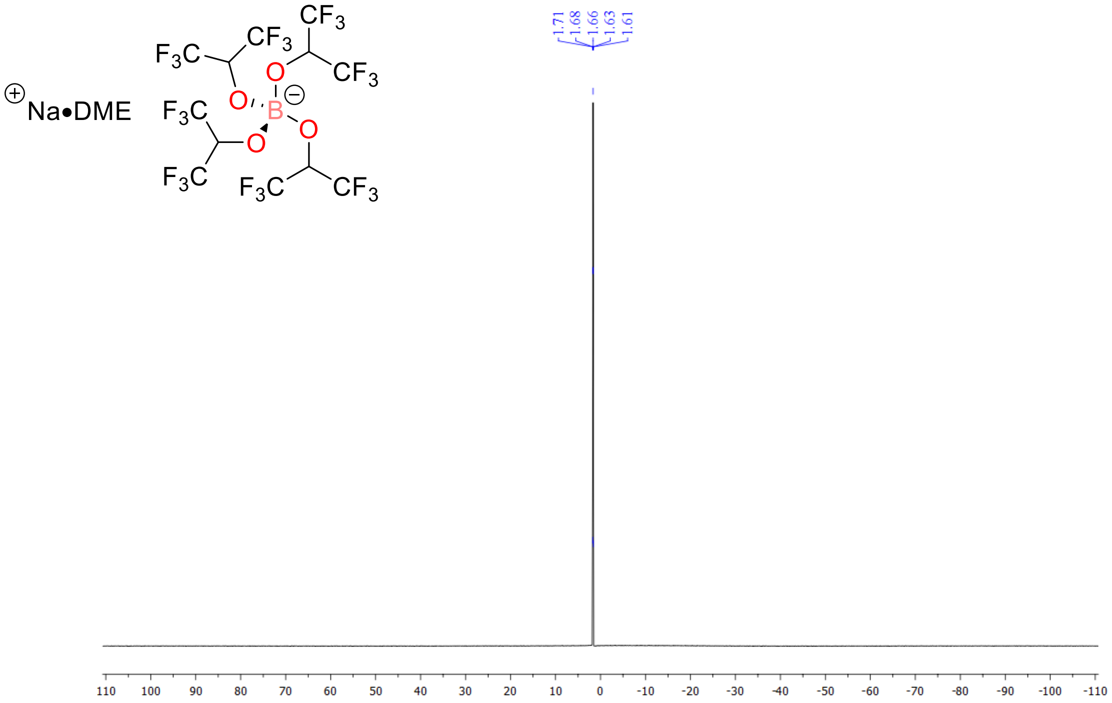

Figure S10.3.3 ^{19}F NMR (376 MHz, CD_3CN, 295 K) spectrum of $Na[B(hfip)_4]\cdot DME$ (**1a**) after 10 equiv of water added.

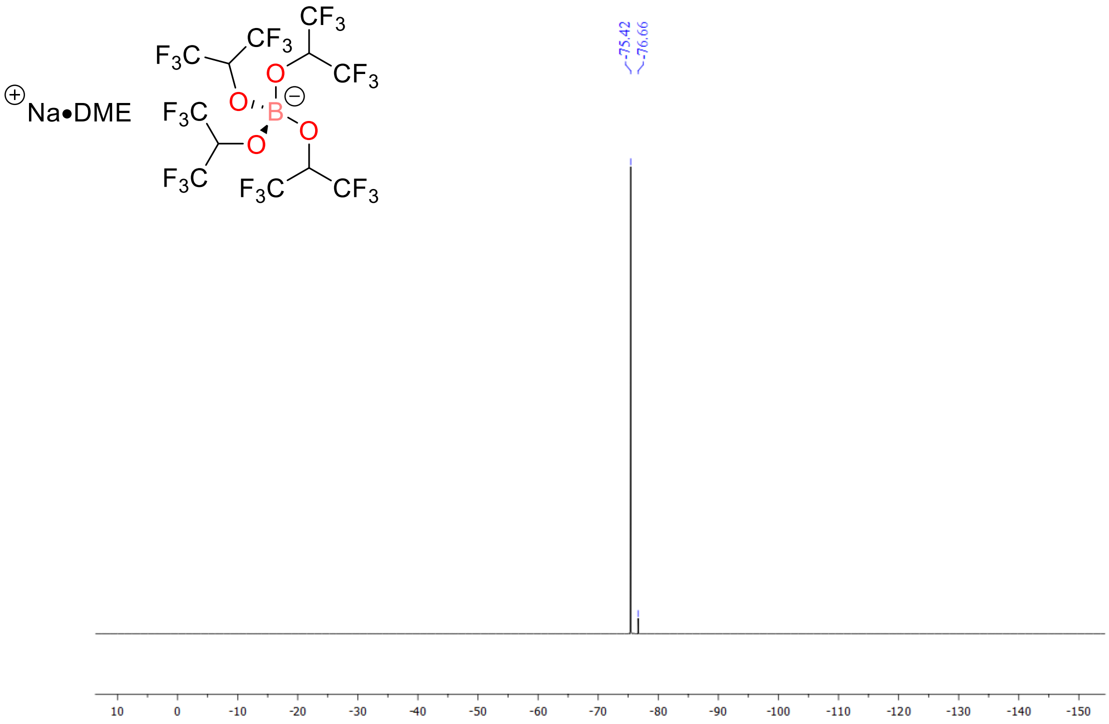

Figure S10.3.4 ^{1}H NMR (400 MHz, CD_3CN, 295 K) spectrum Na[B(pp)$_2$]·3DME (**1b**) after 10 equiv of water added.

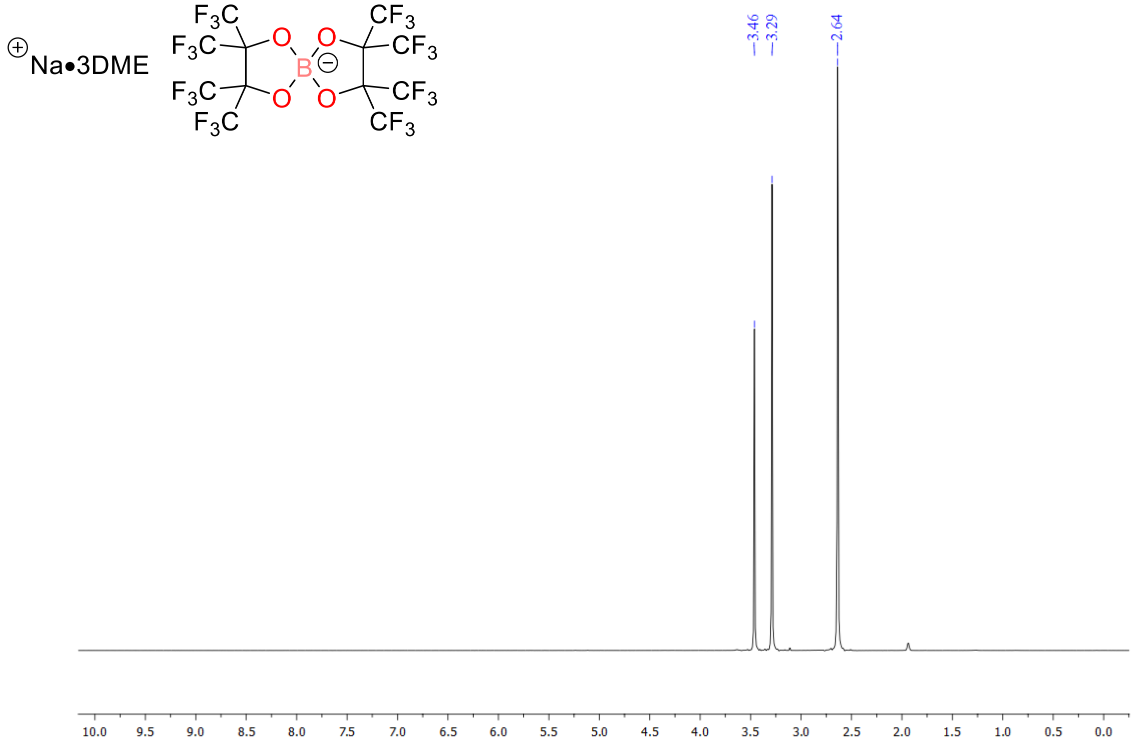

Figure S10.3.5 ^{11}B NMR (128 MHz, CD_3CN, 295 K) spectrum Na[B(pp)$_2$]·DME (**1b**) after 10 equiv of water added.

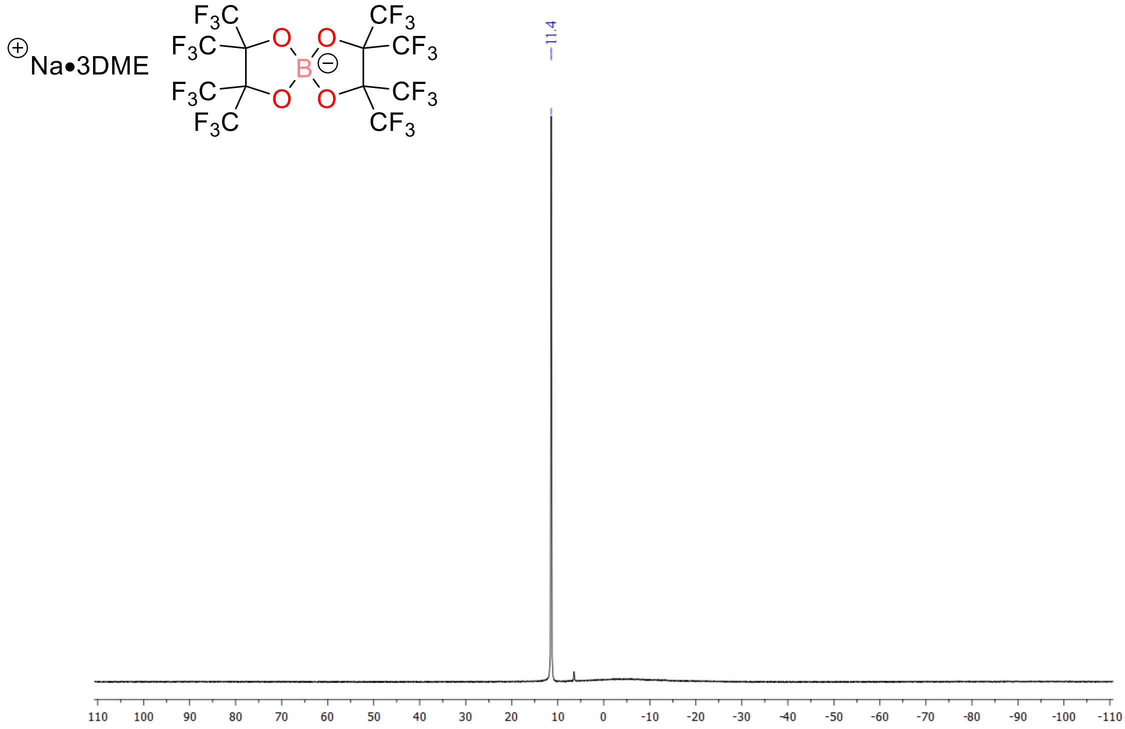

Figure S10.3.6 ^{19}F NMR (376 MHz, CD_3CN, 295 K) spectrum of $Na[B(pp)_2]\cdot 3DME$ (**1b**) after 10 equiv of water added.

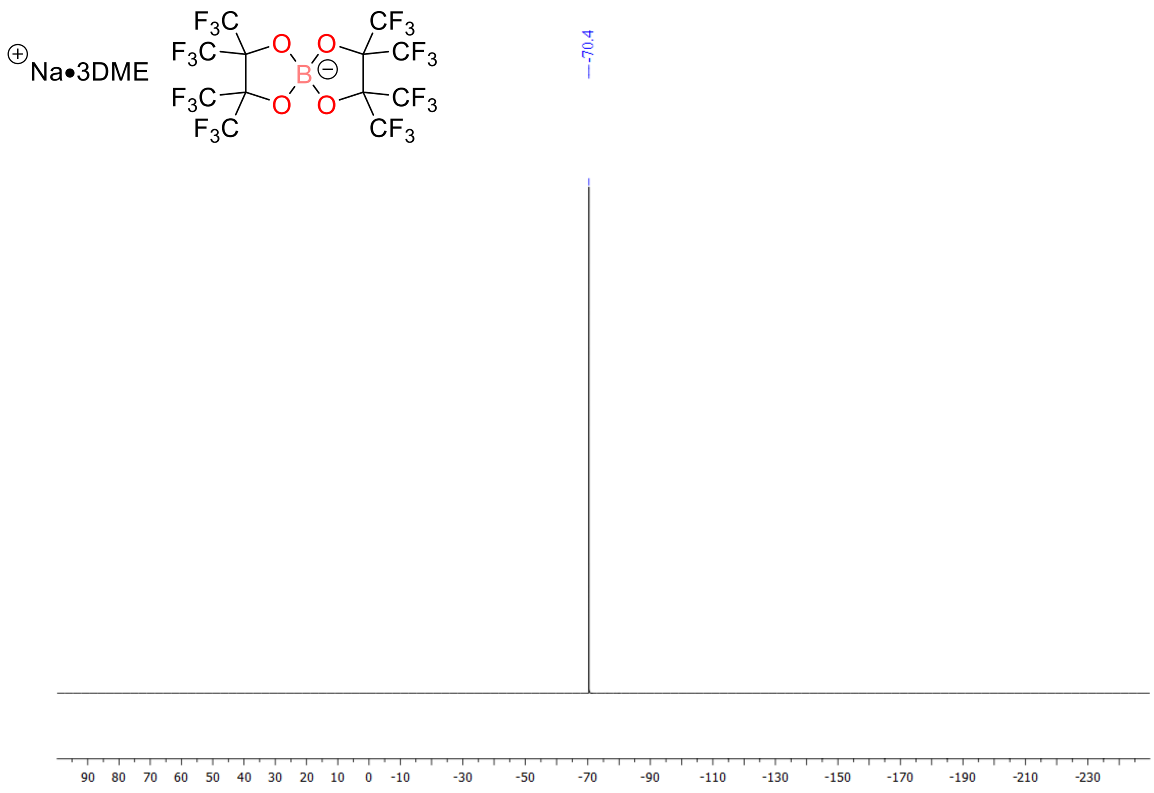

Figure S10.3.7 ^{1}H NMR (400 MHz, D_2O, 295 K) spectrum Na[B(pp)$_2$]·3DME (**1b**) in D_2O after 1 day.

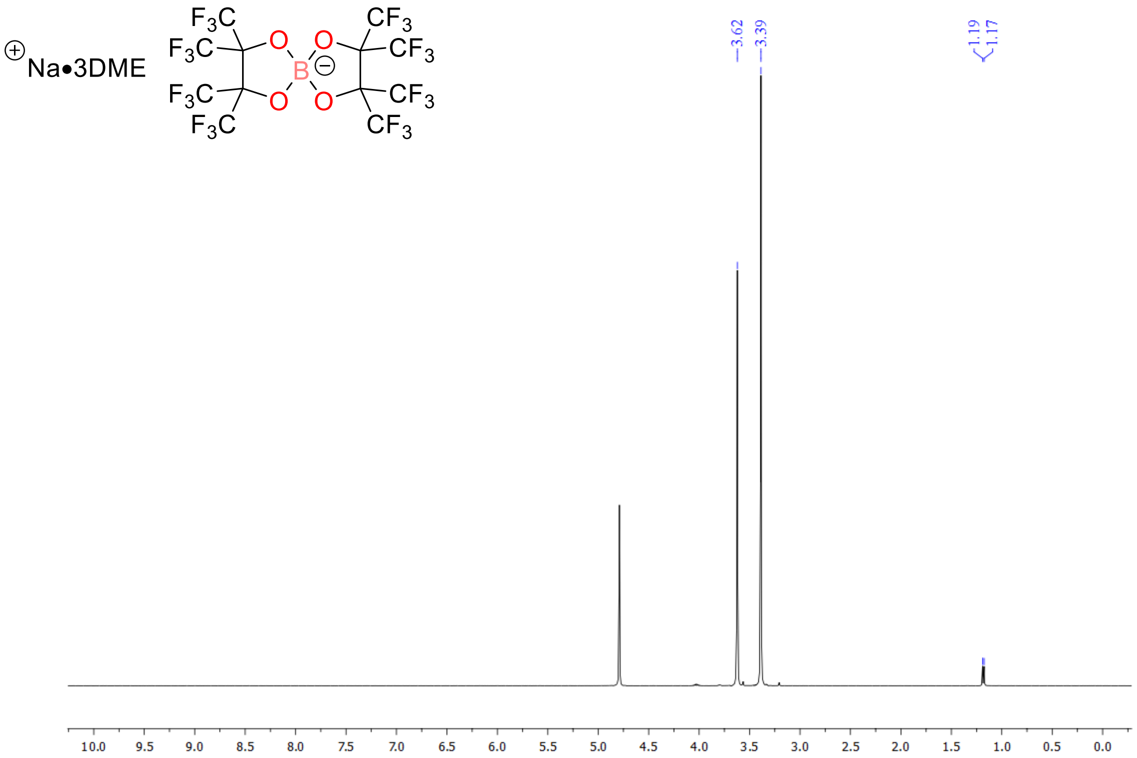

Figure S10.3.8 ^{11}B NMR (128 MHz, D_2O, 295 K) spectrum of Na[B(pp)$_2$]·3DME (**1b**) in D_2O after 1 day.

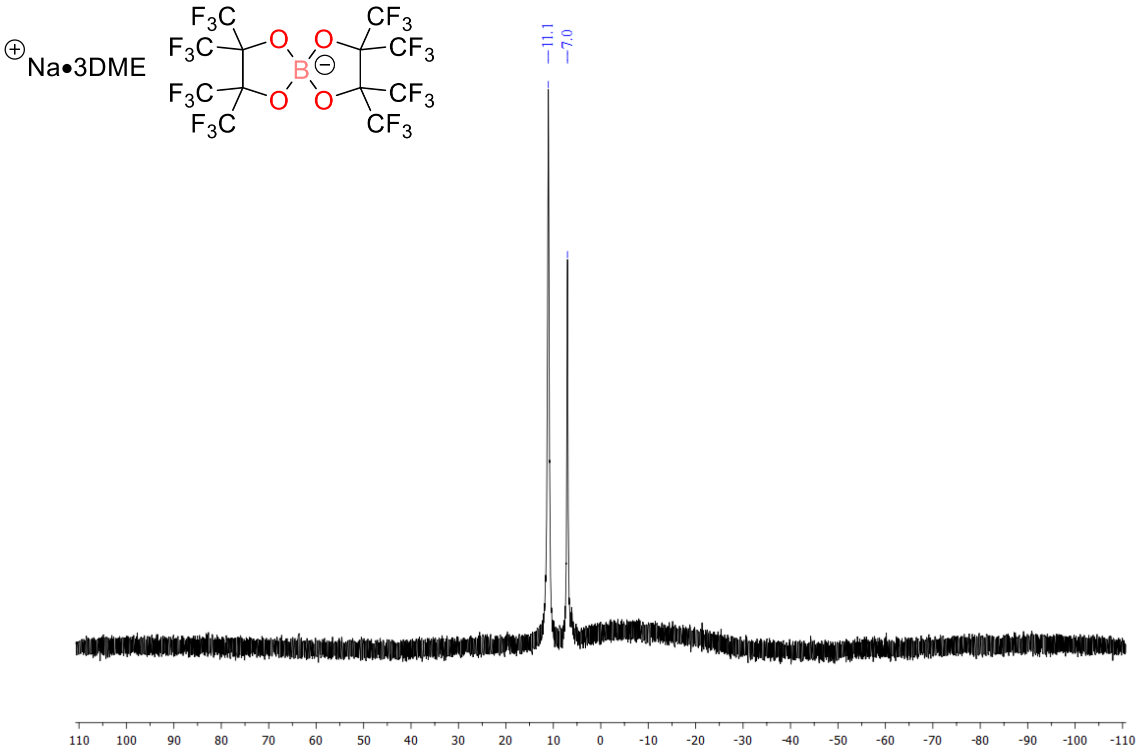

Figure S10.3.9 ^{19}F NMR (376 MHz, D_2O, 295 K) spectrum of Na[B(pp)$_2$]·3DME (**1b**) in D_2O after 1 day.

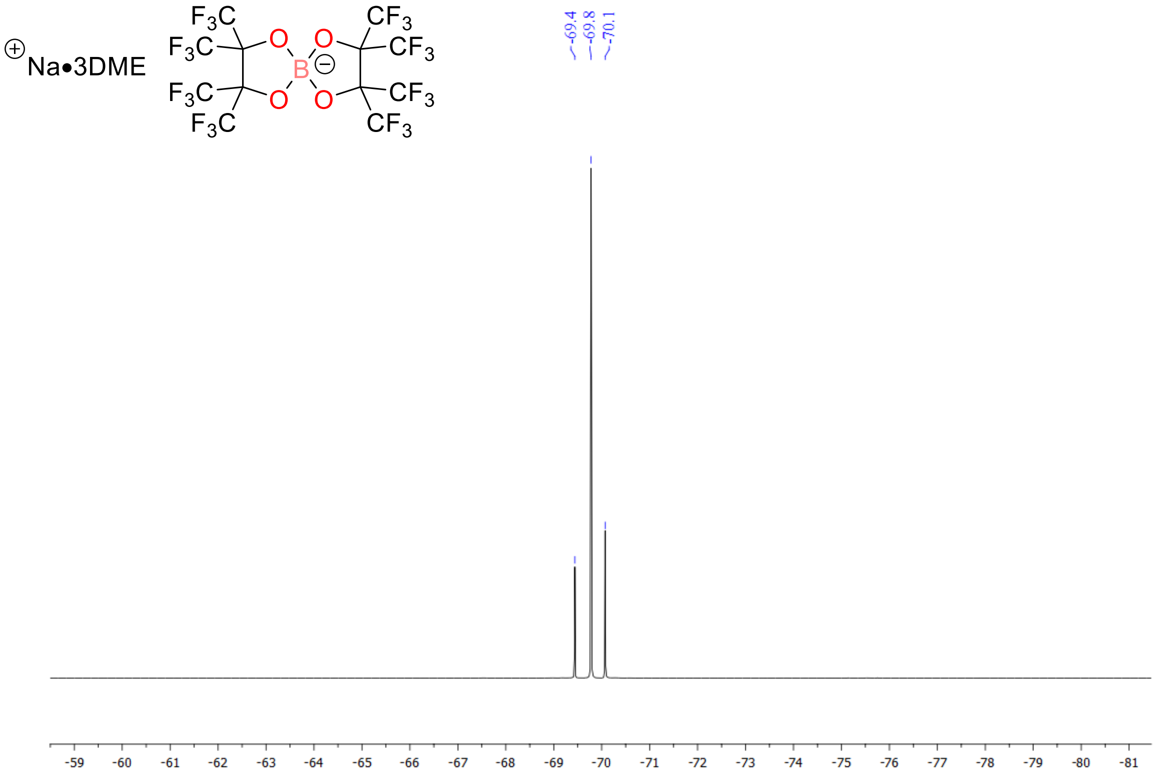

Figure S10.3.10 ^{1}H NMR (400 MHz, CD_3CN, 295 K) spectrum Na[B(pp)$_2$] (**1b'**) after 10 equiv of water added.

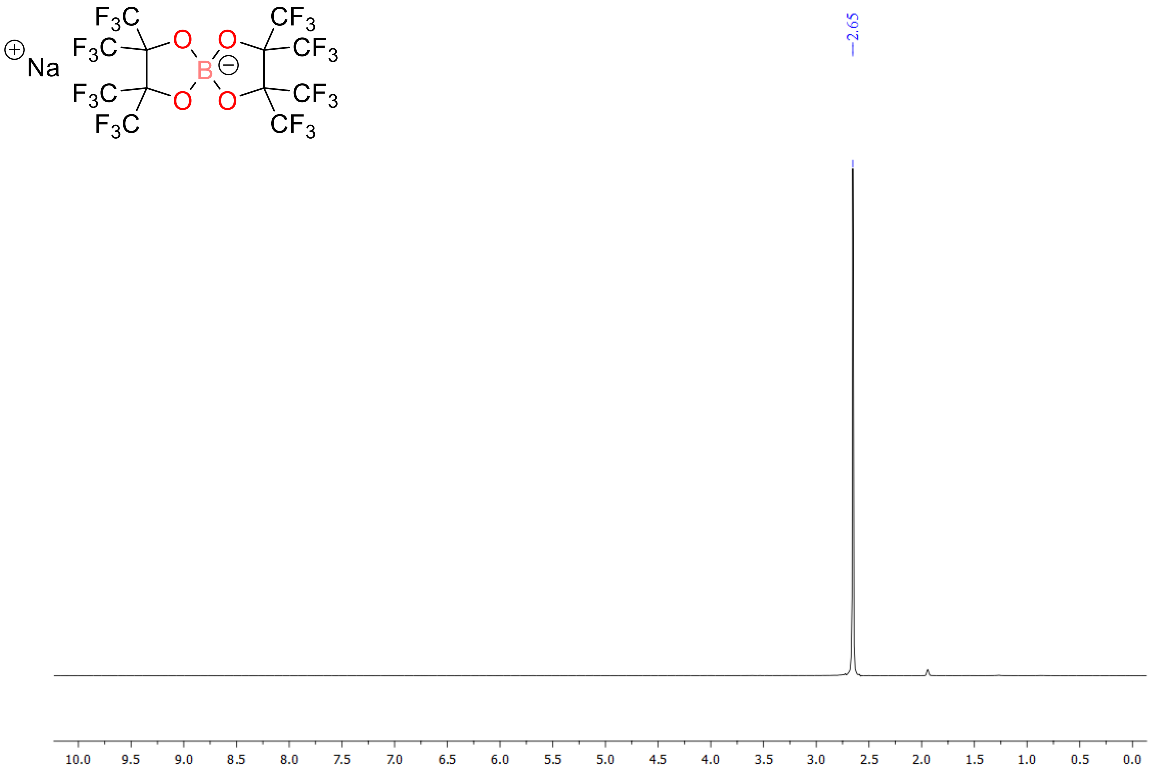

Figure S10.3.11 ^{11}B NMR (128 MHz, CD_3CN, 295 K) spectrum Na[B(pp)$_2$] (**1b'**) after 10 equiv of water added

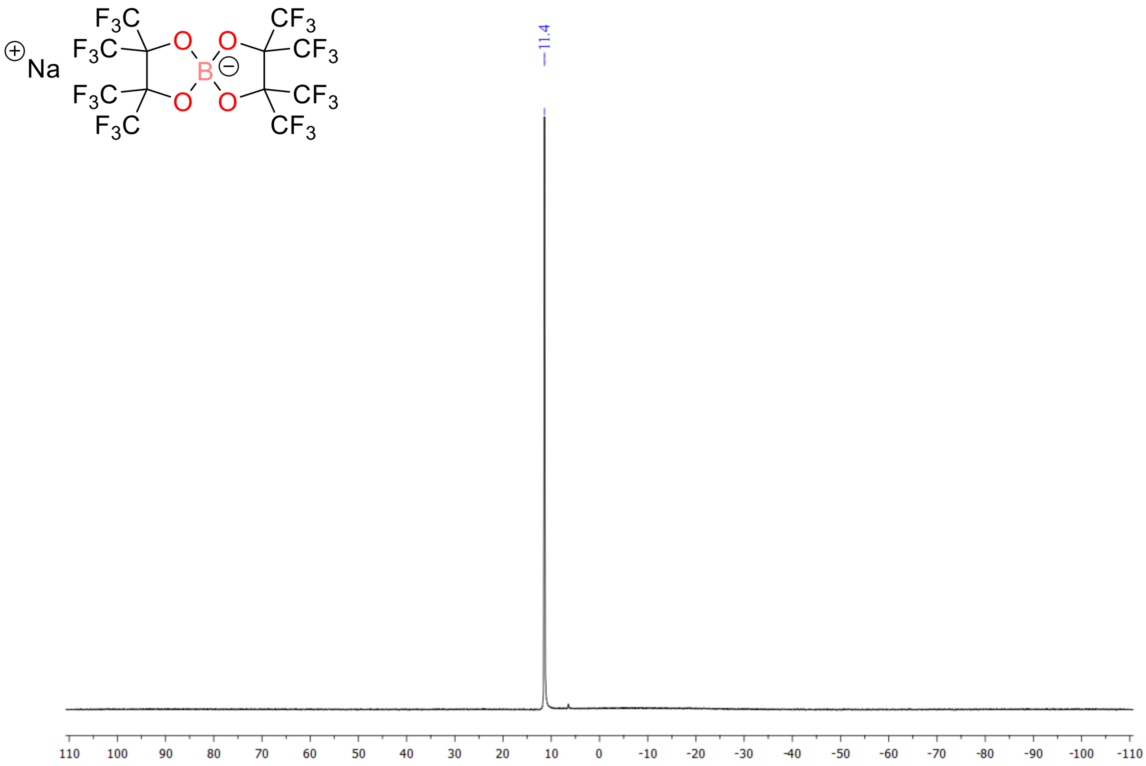

Figure S10.3.12 ^{19}F NMR (376 MHz, CD_3CN, 295 K) spectrum of Na[B(pp)$_2$] (**1b'**) after 10 equiv of water added.

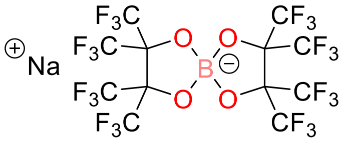

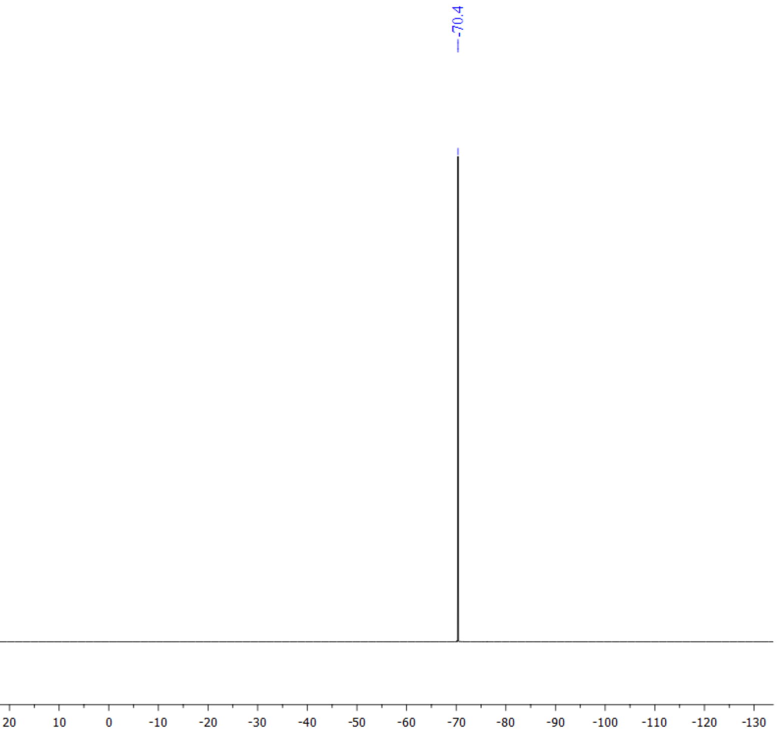

Figure S10.3.13 ^{1}H NMR (400 MHz, CD_3CN, 295 K) spectrum of $Na[B(OCH_2(CF_2)_2CF_3)_4]$ (**1c**) after 1 equiv of water added (24 hours).

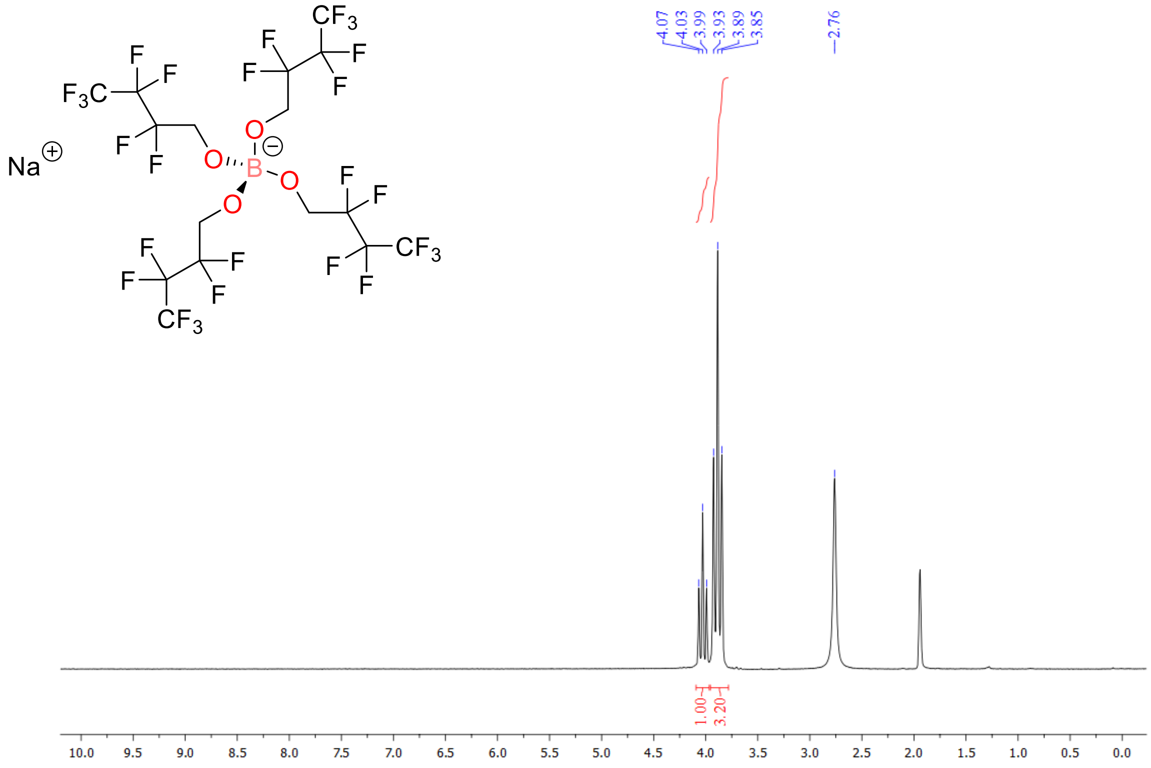

Figure S10.3.14 ^{1}H NMR (400 MHz, CD_3CN, 295 K) spectrum of $Na[B(OCH_2(CF_2)_2CF_3)_4]$ (**1c**) after 10 equiv of water added.

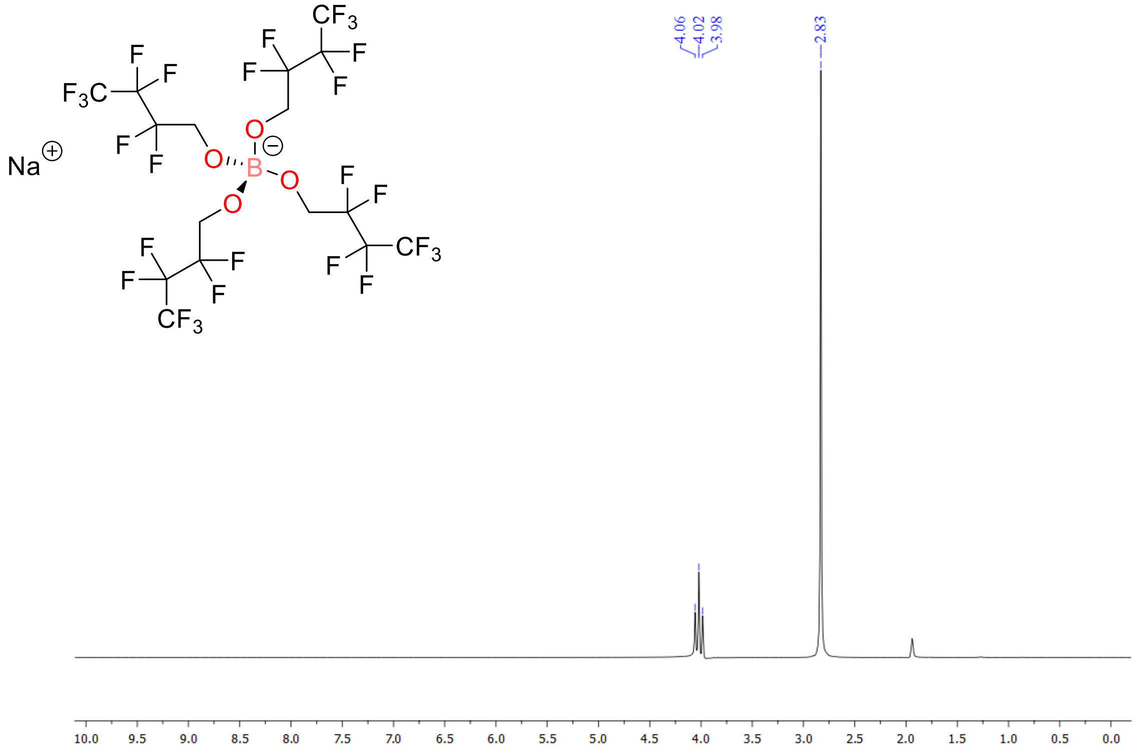

Figure S10.3.15 ^{11}B NMR (400 MHz, CD_3CN, 295 K) spectrum of $Na[B(OCH_2(CF_2)_2CF_3)_4]$ (**1c**) after 10 equiv of water added.

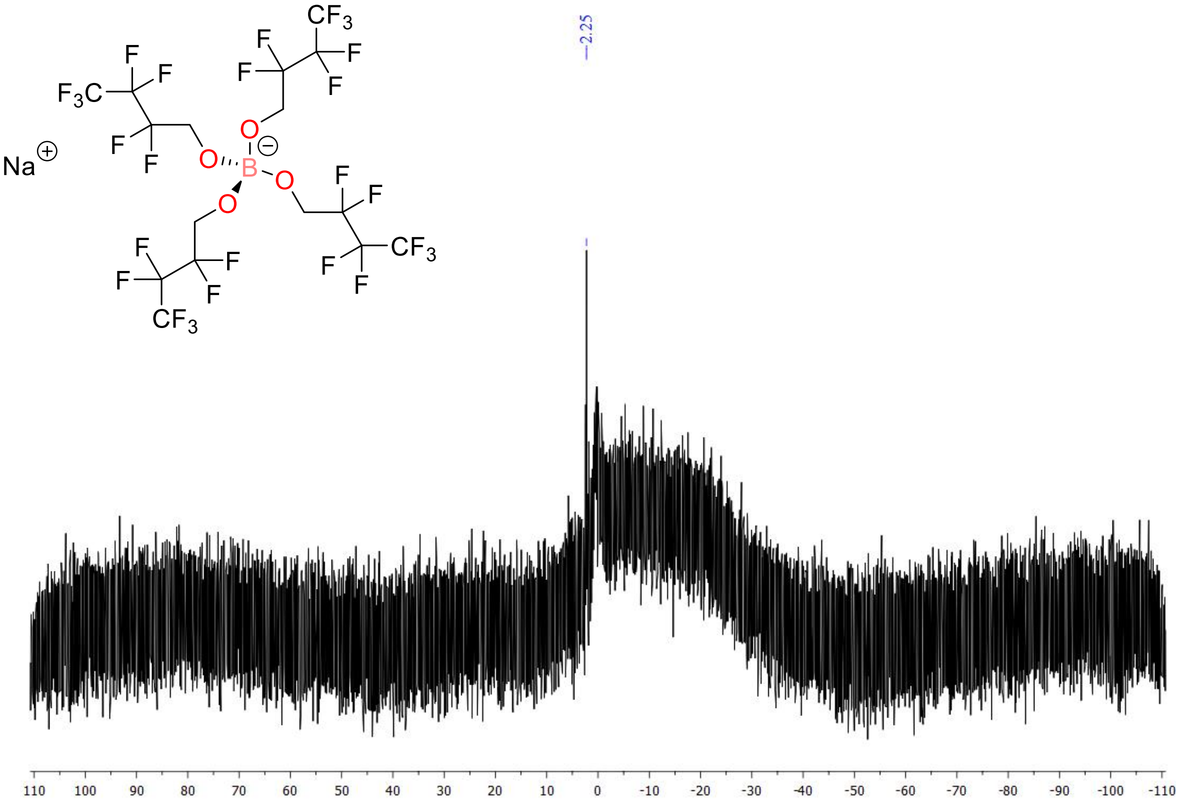

Figure S10.3.16 ^{19}F NMR (376 MHz, CD_3CN, 295 K) spectrum of $Na[B(OCH_2(CF_2)_2CF_3)_4]$ (**1c**) after 10 equiv of water added.

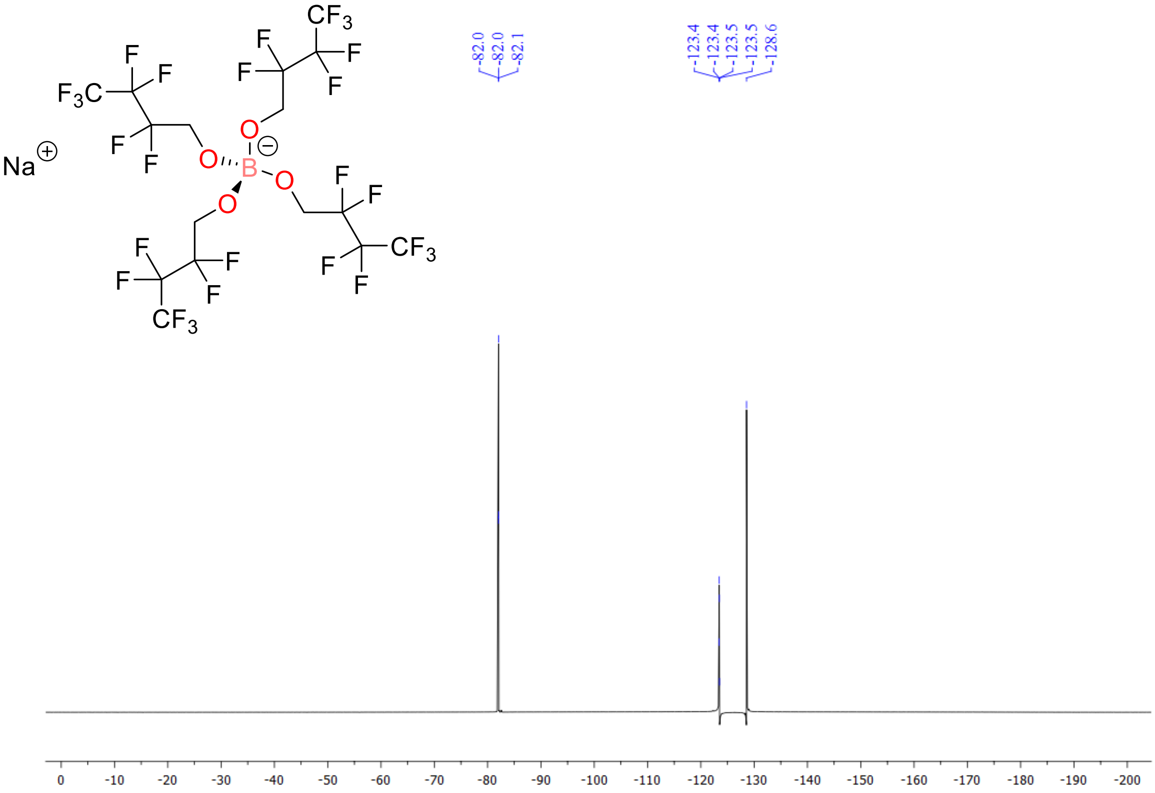

Figure S10.3.17 ^{1}H NMR (400 MHz, CD_3CN, 295 K) spectrum of Na[B(OPh^F)$_4$]·3DME (**1d**) after 1 equiv of water added (24 hours).

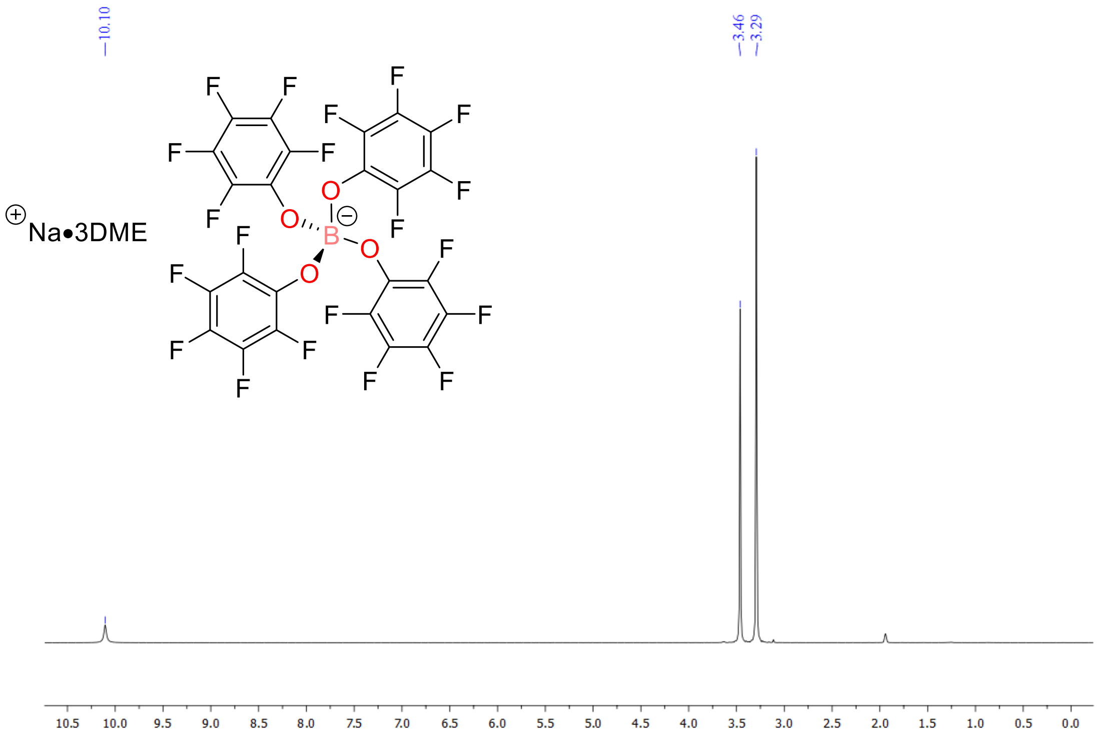

Figure S10.3.18 ^{1}H NMR (400 MHz, CD_3CN, 295 K) spectrum of Na[B(OPh^F)$_4$]·3DME (**1d**) after 10 equiv of water added.

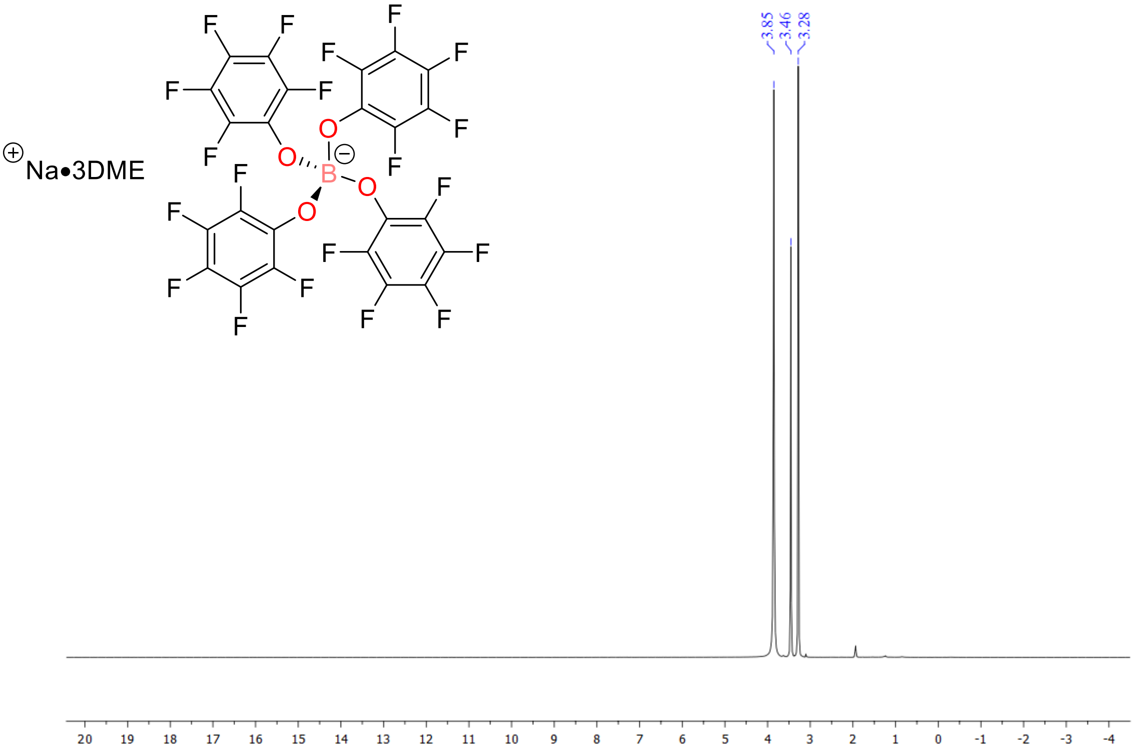

Figure S10.3.19 ^{11}B NMR (128 MHz, CD_3CN, 295 K) spectrum of Na[B(OPh^F)$_4$]·3DME (**1d**) after 10 equiv of water added.

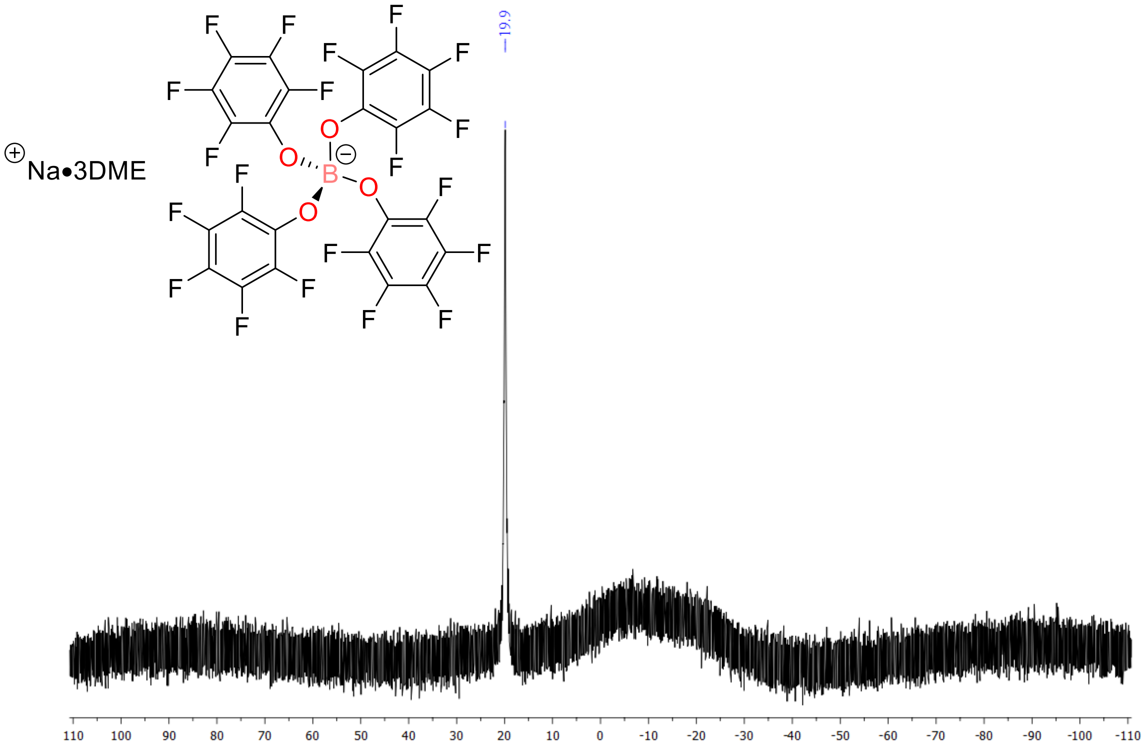

Figure S10.3.20 ^{19}F NMR (376 MHz, CD_3CN, 295 K) spectrum of Na[B(OPh^F)$_4$]·3DME (**1d**) after 10 equiv of water added.

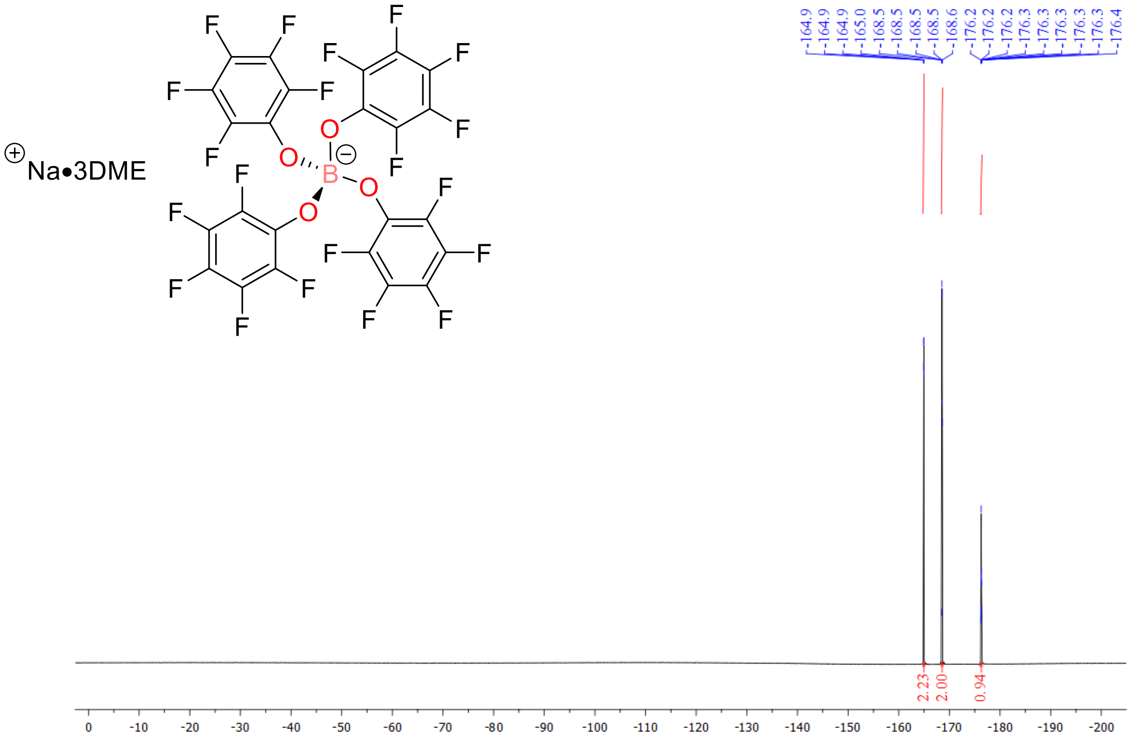

Figure S10.3.21 ^{1}H NMR (400 MHz, CD_3CN, 295 K) spectrum of Na[B(OMe)$_4$] (**1e**) after 5 equiv of water added.

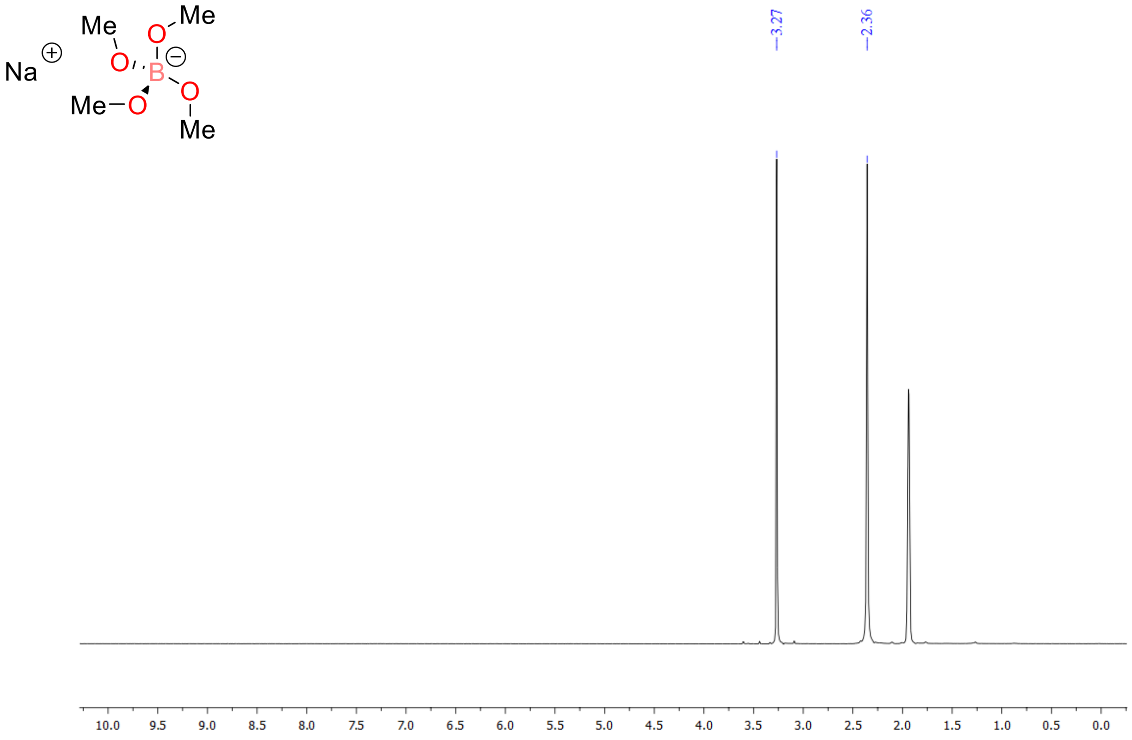

Figure S10.3.22 ^{1}H NMR (400 MHz, CD_3CN, 295 K) spectrum of Na[B(OMe)$_4$] (**1e**) after 10 equiv of water added.

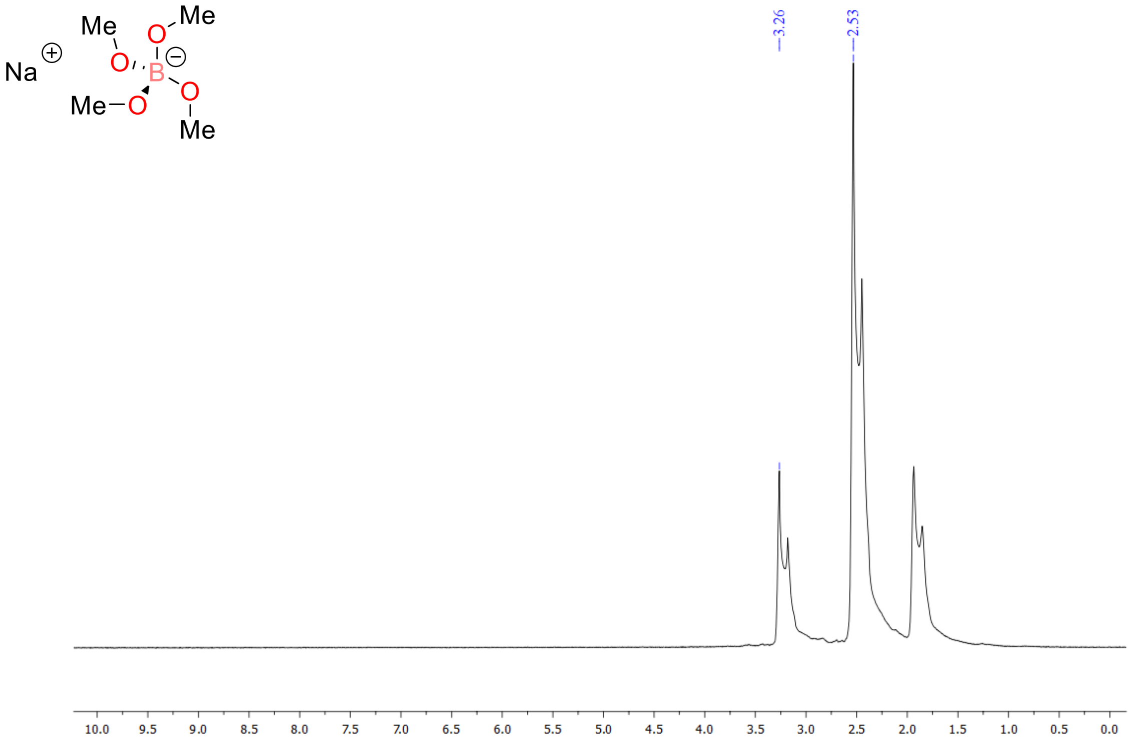

Figure S10.3.23 ^{11}B NMR (128 MHz, $(CD_3)_2SO$, 295 K) spectrum of Na[B(OMe)$_4$] (**1e**) after 1.0 equiv of water added (24 hours).

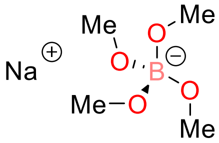

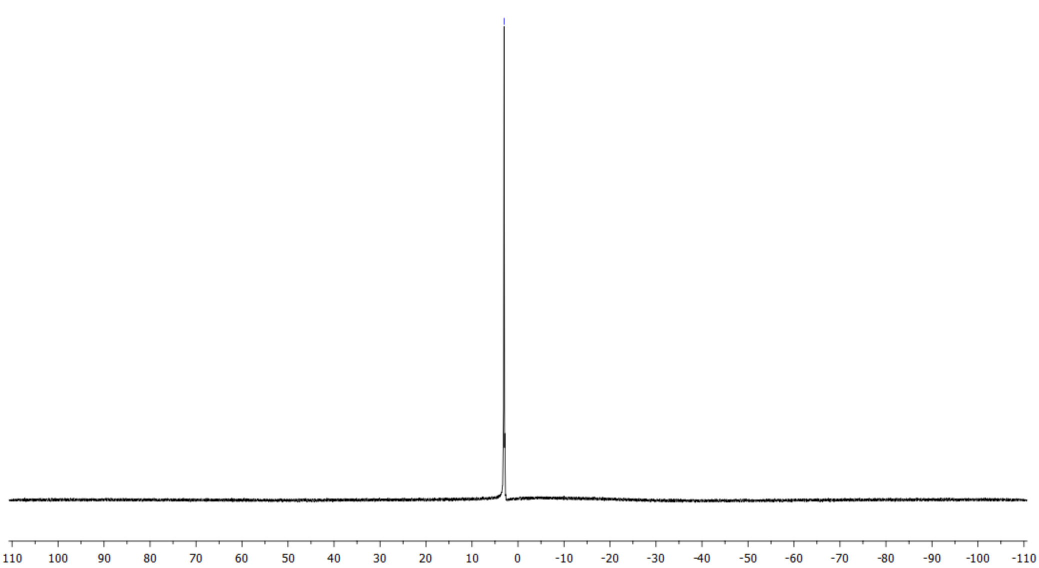

Figure S10.3.24 ^{11}B NMR (128 MHz, $(CD_3)_2SO$, 295 K) spectrum of Na[B(OMe)$_4$] (**1e**) after 10 equiv of water added.

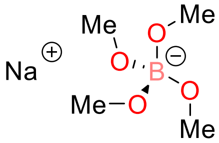

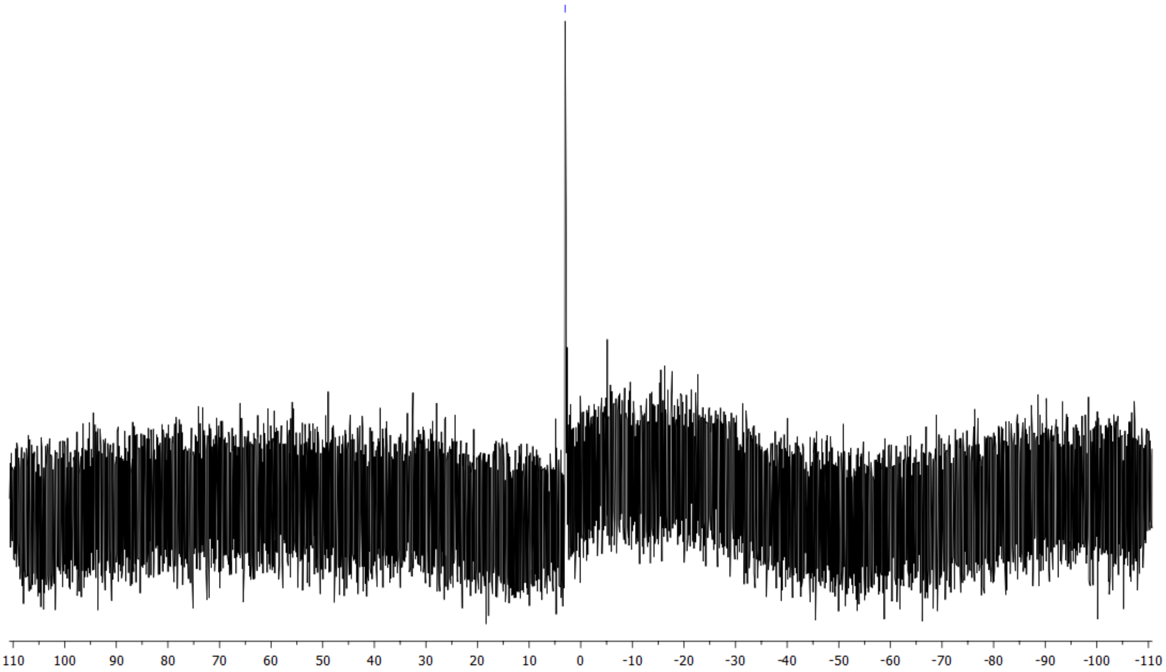

Figure S10.3.25 ^{1}H NMR (400 MHz, CD_3CN, 295 K) spectrum of $Na[B(OPh)_4]$ (**1f**) after 10 equiv of water added.

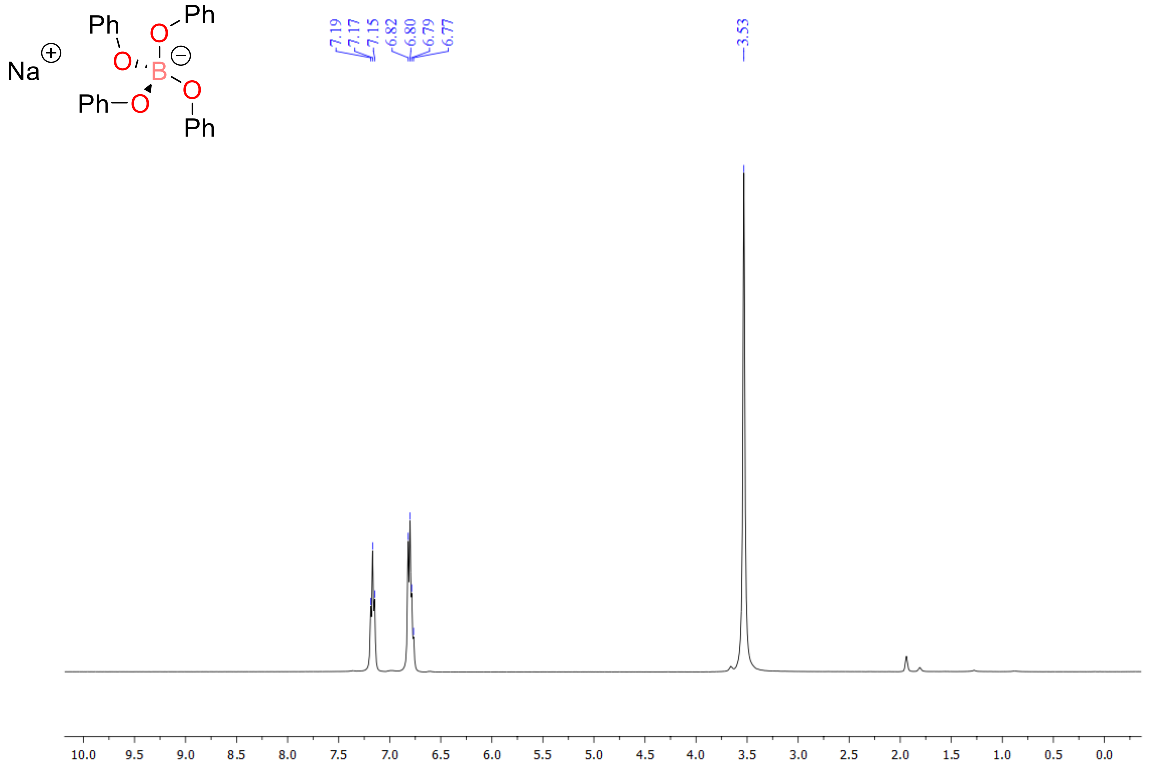

Figure S10.3.26 ^{11}B NMR (128 MHz, CD_3CN, 295 K) spectrum of $Na[B(OPh)_4]$ (**1f**) after 10 equiv of water added.

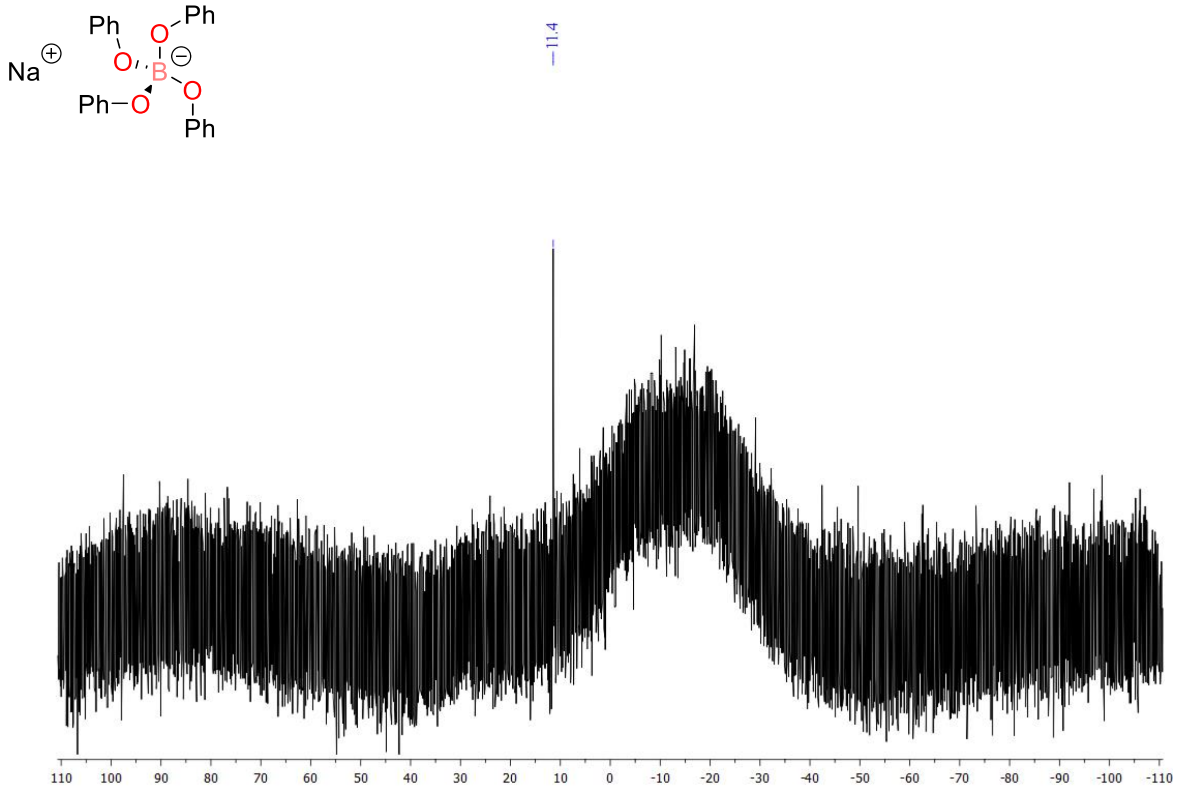

Supplement: Supplementary file 3 — Supporting Information [file ANIE-61-0-s002.pdf]
